# Design, Synthesis, and Antimicrobial Activity of Amide Derivatives Containing Cyclopropane

**DOI:** 10.3390/molecules29174124

**Published:** 2024-08-30

**Authors:** Dongdong Chen, Yu Cheng, Lele Shi, Xueting Gao, Yuhang Huang, Zhenting Du

**Affiliations:** 1 Department of Chemical and Material Engineering, Lyuliang University, Lvliang 033001, China; 2 College of Chemistry & Pharmacy, Northwest A&F University, Yangling 712100, China; cy2021056736@163.com (Y.C.); shilele23@163.com (L.S.); gxt05012022@163.com (X.G.); 15621485160@163.com (Y.H.)

**Keywords:** cyclopropane, antimicrobial activity, molecular docking, synthesis

## Abstract

As an important small organic molecule, cyclopropane is widely used in drug design. In this paper, fifty-three amide derivatives containing cyclopropane were designed and synthesized by introducing amide groups and aryl groups into cyclopropane through the active splicing method, and their antibacterial and antifungal activities were evaluated in vitro. Among them, thirty-five compounds were new compounds, and eighteen compounds were known compounds (**F14**, **F15**, **F18**, **F20**–**F26**, **F36**, and **F38**–**F44**). Bioassay results disclosed that four, three, and nine of the compounds showed moderate activity against *Staphylococcus aureus*, *Escherichia coli*, and *Candida albicans*, respectively. Three compounds were sensitive to *Candida albicans*, with excellent antifungal activity (MIC_80_ = 16 μg/mL). The molecular docking results show that compounds **F8**, **F24**, and **F42** have good affinity with the potential antifungal drug target CYP51 protein.

## 1. Introduction

Infectious diseases caused by bacterial pathogens seriously affect human health and have been a main public health problem in recent years [1]. The use of antibiotics has significantly improved the ability of humans to fight bacterial infections, which prolongs human life and improves the quality of human life [2]. However, the continuous use of antibiotics leads to the emergence of bacterial resistance. At present, one of the major challenges of healthcare worldwide is bacterial resistance to antibiotics [3]. Therefore, it is urgent to develop new, effective, and safe bactericides.

As an important small organic molecule, cyclopropane is widely found in natural products and drugs [4,5,6]. Cyclopropane is a structurally stable bioisostere, which can be used to replace carbon–carbon double bonds. It has the effects of improving the efficacy of drugs, enhancing metabolic stability, reducing the off-target effect of drugs, improving the dissociation degree of drugs, and enhancing the affinity to receptors [7,8,9,10]. Therefore, it is widely used in drug design. Compounds containing cyclopropane have certain drug properties and biological activities, including antibacterial [11,12], antifungal [13,14,15], antiviral [16,17], antitumor [18], antioxidant [19], and antidepressant activities [20,21,22]. Therefore, compounds with cyclopropane structures have attracted extensive research due to their biological activity and low toxicity.

**Figure 1:**
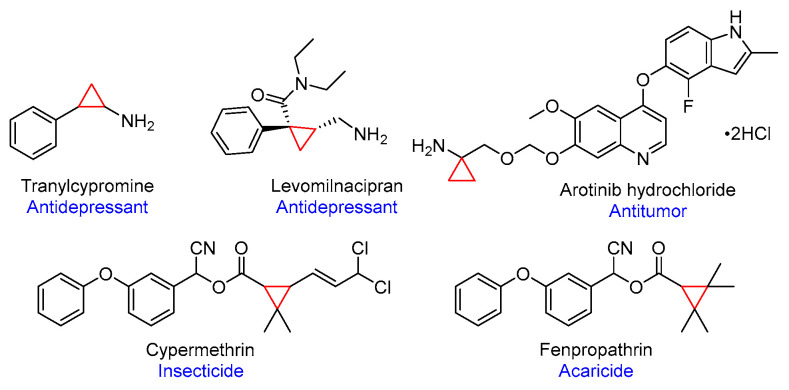
Structure of commercial drugs of cyclopropane.

At present, drugs containing cyclopropane structure have been applied to treat respiratory diseases, infectious diseases, mental disorders, endocrine and metabolic diseases, nervous system diseases, and cardiovascular and cerebrovascular diseases [23]. These include tranylcypromine, levomilnacipran, arotinib hydrochloride, cypermethrin, and fenpropathrin (Figure 1).

Phenyl, as a scaffold that could be substituted with various groups, can enhance the stability and biological activity of drugs [24]. The amide structure, as an important component of proteins, is widely present in antibacterial and antioxidant agents, and it is an important functional group in drug synthesis [25]. Therefore, we speculate that the introduction of substituted phenyl and cyclopropane with amide structure is likely to lead to new lead compounds with excellent biological activity.

**Figure 2:**
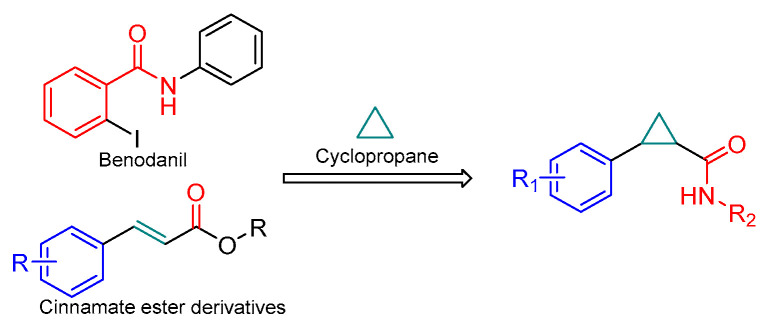
Design strategy of title compounds.

In this study, amide groups and aryl groups of benodanil [26] and cinnamate ester derivatives [27] with excellent antibacterial activity were introduced into the C_1_ and C_2_ positions of cyclopropane by the active splicing method, and fifty-three amide derivatives containing cyclopropane were synthesized (Figure 2). The antibacterial and antifungal activity of the target compounds in vitro was tested. Then, the mode of action and binding affinity of the ideal compounds with the highest antifungal activity and CYP51 protein were analyzed by molecular docking to preliminarily explore their antifungal mechanism.

## 2. Results

### 2.1. Synthesis

In order to explore the effect of different substituent groups on the activity and find more potent compounds, based on the consideration of molecular diversity, 2-phenylcyclopropane-1-carboxamide was used as a template to introduce different substituents into the benzene ring and the amide part, respectively.

**Scheme 1:**
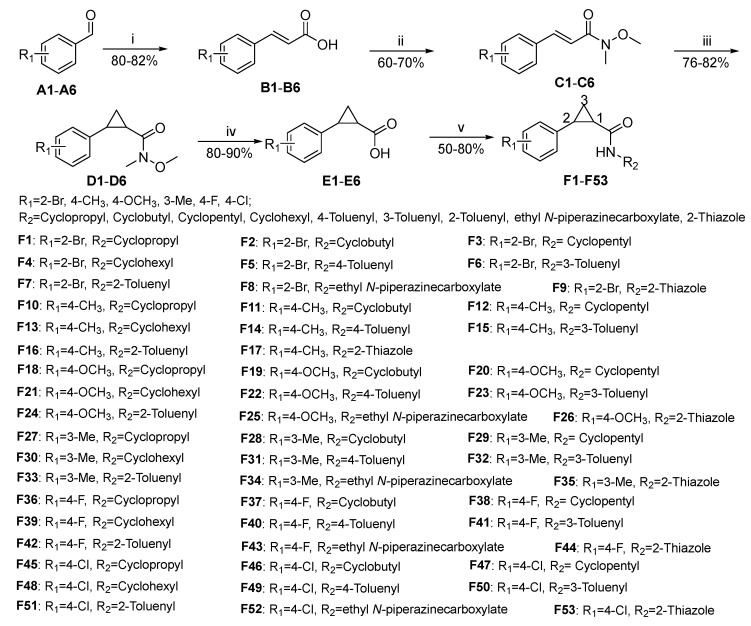
Synthetic reaction and structures of target compounds. Reagents and conditions: (i) Malonic acid, DMF, 90 °C, 6 h, yield: 80–82%; (ii) DMAP, N,O-dimethylhydroxylamine hydrochloride, EDCI, DCM, rt, 2 h, yield: 60–70%; (iii) NaH, Trimethylsulfonyl iodide, THF, 25 °C, yield: 76–82%; (iv) MeOH, NaOH, 25 °C, yield: 80–90%; (v) THF, EDCI, HOBT, amine, 37 °C, yield: 50–80%.

The synthetic rout is outlined in Scheme 1. Intermediate **B** was obtained by Knoevenagel condensation of substituted benzaldehyde **A** and malonic acid. Intermediate **B** was amidated with N,O-dimethylhydroxylamine hydrochloride to form intermediate **C**. Intermediate **D** was obtained from intermediate **C** and trimethylsulfonyl iodide by Corey-Chaykovsky cyclopropanation. Intermediate **D** was hydrolyzed to obtain intermediate **E**. A series of amide compounds containing cyclopropane **F1**–**F53** were obtained by amide reaction of intermediate **E** with aliphatic amine, methylaniline, ethyl N-piperazinecarboxylate, and 2-aminothiazole, respectively.

Among the fifty-three cyclopropane amide derivatives synthesized in this study, thirty-five compounds were new compounds, and eighteen compounds were known compounds (**F14**, **F15**, **F18**, **F20**–**F26**, **F36**, **F38**–**F44**). All of the synthesized compounds were characterized by ^1^H NMR, ^13^C NMR, and HRMS. The ^1^H and ^13^C NMR spectra and HRMS of the compounds above are shown in Supplementary Materials.

In ^1^H-NMR spectra, fatty amides (**F1**–**F4**, **F10**–**F13**, **F18**–**F21**, **F27**–**F30**, **F36**–**F39**, **F45**–**F48**) showed one doublet signal in the ranges of *δ*_H_ 7.95–8.39 due to H-N. Benzamides (**F5**–**F7**, **F14**–**F16**, **F22**–**F24**, **F31**–**F33**, **F40**–**F42**, **F49**–**F51**) and thiazole amides (**F9**, **F17**, **F2**6, **F35**, **F44**, **F53**) showed one single signals in the ranges of *δ*_H_ 9.52–10.43 and*δ*_H_ 12.34–12.43 due to H-N, respectively. The two hydrogens on C_1_ and C_2_ show two sets of multiple signals in the ranges of *δ*_H_ 2.70–1.65. The two hydrogens on C3 show two sets of multiple signals in the ranges of *δ*_H_ 1.75–1.21. In ^13^C-NMR spectra, the amides showed a signal of one carbonyl in the ranges of *δ*_C_ 169.43–172.48. In ^19^F-NMR spectra, the compounds (**F36**–**F44**) showed a signal of one F in the ranges of *δ*_F_ −116.54–−117.24.

### 2.2. Antimicrobial Activity In Vitro

Three strains of bacteria, including Gram-positive bacteria (*Staphylococcus aureus* (*S. aureus*) and *Escherichia coli* (*E. coli*)) and Gram-negative bacteria (*Pseudomonas aeruginosa* (*P. aeruginosa*)), and one strain of fungu (*Candida albicans* (*C. albicans*)), which causes great harm to human health, were selected to test the biological activity of the compounds in vitro. The antibacterial activity and antifungal activity against all synthesized amide derivatives containing cyclopropane (**F1**–**F53**) were evaluated in vitro. The in vitro antibacterial and antifungal activities of the prepared compounds were determined by the MIC_80_ value (minimum 80% inhibition concentration) using microdilution method with ciprofloxacin or fluconazole as positive controls, respectively. The results are listed in Table 1. Five compounds (**F7**, **F30**, **F36**, **F49**, and **F51**) showed some antibacterial activity against Staphylococcus aureus with the MIC_80_ of 128 μg/mL, and four compounds (**F5**, **F9**, **F29**, and **F53**) showed moderate inhibitory activity against Staphylococcus aureus with the MIC_80_ of 32 and 64 μg/mL. Two compounds (**F5** and **F53**) show antibacterial activity against Escherichia coli with the MIC_80_ of 128 μg/mL. Escherichia coli were relatively sensitive to three compounds (**F9**, **F31**, and **F45**) with the MIC_80_ of 32 and 64 μg/mL, respectively, but far lower than the antibacterial activity of the positive control ciprofloxacin (MIC_80_ = 2 μg/mL). Pseudomonas aeruginosa was not sensitive to all compounds with high MIC_80_ values (>128 μg/mL).

Six compounds (**F4**, **F10**, **F14**, **F33**, **F34**, and **F36**) showed some antifungal activity against Candida albicans with the MIC_80_ of 128 μg/mL, but the activity was far lower than that of fluconazole (MIC_80_ = 2 μg/mL). Candida albicans were more sensitive to nine compounds (**F5**, **F7**, **F9**, **F22**, **F23**, **F32**, **F49**, **F50**, and **F51**) which showed moderate activity (MIC_80_ = 32, 64 μg/mL). Three compounds (**F8**, **F24**, and **F42**) showed promising antifungal activity with the MIC_80_ of 16 μg/mL.

Although the activity of the synthesized compounds is inferior to ciprofloxacin and fluconazole, this class of compounds showed some attractive advantages, such as simple structure, easy synthesis, and low cost.

### 2.3. The Preliminary Structure–Activity Relationships

The comparison of the antimicrobial activity and structure of the compounds in Table 1 showed that the types and substitution sites of benzene ring and amide substituents significantly influence the antimicrobial activity of the compounds. The antibacterial activity of the target compounds showed that aryl amides showed higher activity than fatty amides (**F1**–**F4, F8** vs**. F5**–**F7**, **F9** (*S. aureus*, *E. coli*))). The cyclopropane derivatives substituted by piperazine amide did not show antibacterial activity (**F8**, **F25**, **F34**, **F43**, **F52** (*S. aureus*, *E. coli*)). Thiazole amide (**F9**) showed the best antibacterial activity (MIC_80_ = 32 μg/mL (*E. coli*)). The introduction of halogen at the 2-position of the benzene ring showed better antibacterial activity than the 4-position (**F9** vs. **F52**, **F44** (*S. aureus*, *E. coli*)). The introduction of halogens (F, Cl, Br) in the benzene ring was beneficial to the improvement of antibacterial activity compared with methoxy groups as an electron-donating (**F5**, **F49** vs. **F22**; **F7**, **F51** vs. **F24**; **F9**, **F53** vs. **F26** (*S. aureus*, *E. coli*)). Similar to the antibacterial activity, the presence of fatty amides cannot improve antifungal activity (**F1**–**F4**, **F10**–**F13**, **F18**–**F21**, **F27**–**F30**, **F36**–**F39**, **F45**–**F48** (*C. albicans*)) and the introduction of halogens in the benzene ring was beneficial to the improvement of antifungal activity (**F5**, **F49** vs. **F14**, **F31**; **F7**, **F42**, **F51** vs. **F16**, **F33** (*C. albicans*)). This is consistent with the performance of cinnamic acid ester derivatives with similar structures [28]. Unlike antibacterial activity, the introduction of methoxy groups in the benzene ring also contributed to the improvement of antifungal activity (**F22**–**F24** vs. **F14**–**F16**, **F31**–**F33** (*C. albicans*)). The introduction of benzamide was more conducive to the improvement of antifungal activity, and *o*-tolyl amide showed better antifungal activity than *m*-tolyl amide and *p*-tolyl amide. (**F7** vs. **F5**–**F6**; **F24** vs. **F22**–**F23**; **F42** vs. **F40**–**F41**; **F51** vs. **F49**–**F50** (*C. albicans*)).

### 2.4. Molecular Docking Studies

**Figure 3:**
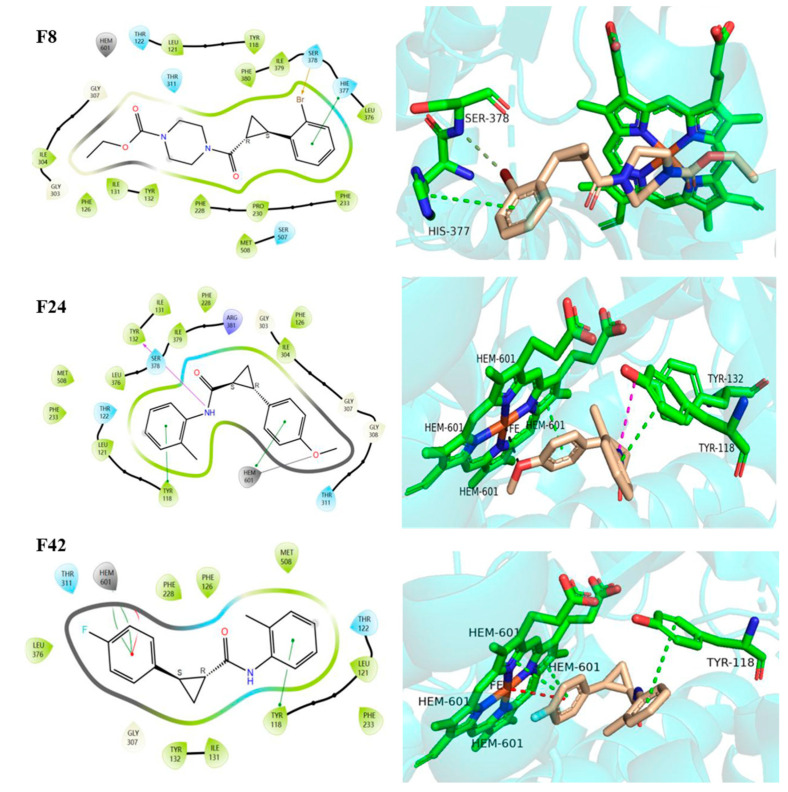
Binding interactions of compounds (**F8**, **F24**, and **F42**) with CYP51 protein (PDB code: 6AY6).

Sterol 14-α demethylase (CYP51), as a key enzyme in the synthesis of biosterols, is a potential target for antifungal drugs. It has the functions of regulating and distributing proteins and changing the fluidity and permeability of membranes, and it is an enzyme necessary for the growth and development of various eukaryotes [29,30]. In order to further explore the antifungal activity mechanism of the target compounds, molecular docking was used to predict the mode of action and binding affinity of excellent antifungal compounds (**F8**, **F24**, and **F42**) with CYP51 protein. The molecular docking results are shown in Figure 3. Compound **F8** is able to bind with CYP51 by interaction with two amino residues of SER378 and HIE377. The binding modes involved one halogen bond and one π…π interaction. Compared with **F8**, **F42** is mainly bound with CYP51 by interaction with TYR118 and HEM601 involved two π…π interaction. Compared with **F42**, **F24** also forms two additional interactions with CYP51 including two hydrogen bond ((CO)N…TYR132, (CH_3_)O…HEM601). The compounds **F8**, **F24**, and **F42** have good binding affinity with CYP51 and gave −6.087, −6.526, and −6.874 kcal/mol of docking scores, respectively. Compared with the reported compounds with excellent antifungal activity, the binding energies were slightly lower [13]. This may be the reason why the antibacterial activity of the above compounds was lower than that of fluconazole. The results further verified the potential bacteriostatic effect of derivatives **F8**, **F24** and **F42**.

## 3. Materials and Methods

### 3.1. Chemistry

All reactions were monitored by thin-layer silica chromatography (TLC) with GF_254_ under 254 nm UV light (Beijing Innochem Science&Technology Co.,Ltd, Beijing, China). The melting points (m.p.) of all synthesized chemicals were determined on an XT-4 micro-melting point apparatus (Hanon Technologies, Jinan, China). Compounds were purified by column chromatography with silica gel (200–300 mesh) (Qingdao Haiwan Chemical, Qingdao, China) using a mixture of ethyl acetate and petroleum ether as eluent. ^1^H nuclear magnetic resonance (^1^H NMR) and ^13^C NMR spectra were recorded on a Bruker AVANCE III operating at 400 and 100 MHz (Bruker, Karlsruhe, Germany) instrument in DMSO-*d*_6_ and using tetramethyl silane (TMS) as an internal standard. All chemical shifts (δ values) are given in ppm, and coupling constants (*J* values) are given in Hz. High-resolution mass spectra (HRMS) were obtained using a Bruker micrOTOF-Q II focus spectrometer (ESI) (AB SCIEX, Framingham, MA, USA). All starting materials were purchased from commercial suppliers and used without further purification unless otherwise stated.

### 3.2. General Synthesis Procedure for Intermediate B

Substituted benzaldehyde (**A1**–**A6**, 100 mmol), malonic acid (300 mmol), and pyridine (100 mmol) were added to a 500 mL flask containing *N*,*N*-dimethylformamide (100 mL) solution. The solution was reacted at 90 °C for 6 h. The reaction process was monitored by TLC. To the mixture was added water (60 mL) and concentrated HCl to pH = 1. Then, the solution was cooled to 0 °C. Filtrate and wash solid with ice water. The solid was collected and dried to give the intermediate B in 80–82% yield [31].

### 3.3. General Synthesis Procedure for Intermediate C

The intermediates **B** (70 mmol), *N*,*O*-dimethylhydroxylamine hydrochloride (105 mmol), 4-dimethylaminopyridine (DMAP, 14 mmol) and 1-(3-dimethylaminopropyl)-3-ethylcarbodiimide hydrochloride (EDCI, 105 mmol) were added to a 500 mL flask containing 100 mL dichloromethane at 0 °C. Triethylamine (105 mmol) was added slowly, and the reaction mixture was warmed slowly to room temperature and stirred for 2 h. The mixture was quenched with 1 mol/L HCl (10 mL) and extracted with ethyl acetate (50 mL × 3). The combined organic phase was washed with aqueous saturated NaHCO_3_ (100 mL × 3) and brine, dried over anhydrous Na_2_SO_4_, and concentrated to give the intermediate **C** in 60–70% yield [32].

### 3.4. General Synthesis Procedure for Intermediate D

To a solution of NaH (174 mmol) in anhydrous tetrahydrofuran (THF) (350 mL) was added intermediate **C** (60 mmol) dropwise under argon at 0 °C. The reaction mixture was allowed to be stirred at 0 °C for 0.5 h. Then trimethylsulfonyl iodide (79.8 mmol) was added. The reaction mixture was stirred at room temperature for 18 h, quenched with water, and extracted with ethyl acetate (50 mL × 3). The combined organic phase was washed with brine, dried over anhydrous Na_2_SO_4_, and concentrated to give the intermediate **D** in 76–82% yield [33].

### 3.5. General Synthesis Procedure for Intermediate E

The intermediate **D** (50 mmol) and sodium hydroxide (NaOH, 100 mmol) were dissolved in a mixed solution of methanol (75 mL) and water (10 mL) and stirred overnight at 25 °C. To the mixture was added diluted hydrochloric acid HCl to Ph = 3–4. The mixture was extracted with dichloromethane (DCM, 50 mL × 3). The combined organic phase was washed with brine, dried over anhydrous Na_2_SO_4_, and concentrated to give the intermediate **E** in 76–82% yield [34].

### 3.6. General Synthesis Procedure for ***F1**–**F53***

The intermediate **E** (2 mmol), *N*-(3-Dimethylaminopropyl)-*N*′-ethylcarbodiimide hydrochloride (EDCI, 2.4 mmol) and 1-Hydroxybenzotriazole (HOBT, 2.4 mmol) were dissolved in anhydrous tetrahydrofuran (THF, 10 mL). Then, the reaction mixture was stirred for 5 min at room temperature. The corresponding amine (3 mmol) was added and stired at 37 °C overnight. Water (20 mL) was added and extracted with ethyl acetate (20 mL × 3). The combined organic phase was washed with brine, dried over anhydrous Na_2_SO_4_, and concentrated. The residue was purified by column chromatography on silica gel with petroleum ether/ethyl acetate as eluent to give **F1**–**F53** in 50–80% yield [35].

*2-(2-Bromophenyl)-N-cyclopropylcyclopropane-1-carboxamide* (**F1**): White solid, Yield: 68.9%, m.p. 119.2–120.0 °C. ^1^H NMR (400 MHz, DMSO-*d*_6_) δ 8.20 (d, *J* = 4.2 Hz, 1H, H-N), 7.59 (dd, *J* = 8.0, 1.3 Hz, 1H), 7.33–7.29 (m, 1H), 7.17–7.13 (m, 1H), 7.08 (dd, *J* = 7.8, 1.7 Hz, 1H, Ar-H), 2.68–2.64 (m, 1H), 2.42–2.39 (m, 1H, H-2), 1.74–1.65 (m, 1H, H-1), 1.35–1.32 (m, 1H, H-3), 1.26–1.23 (m, 1H, H-3), 0.64–0.59 (m, 2H), 0.48–0.35 (m, 2H). ^13^C NMR (100 MHz, DMSO-*d*_6_) δ 172.33 (C=O), 139.75, 132.29, 128.23,127.91, 127.14, 125.11, 24.52, 24.47, 22.49, 13.97, 5.84, 5.78. HRMS (ESI) *m*/*z* [M + Na]^+^ calcd for C_13_H_14_BrNNaO: 302.0151, found: 302.0160.
*2-(2-Bromophenyl)-N-cyclobutylcyclopropane-1-carboxamide* (**F2**): White solid, Yield: 73.3%, m.p. 135.4–136.7 °C. ^1^H NMR (400 MHz, DMSO-*d*_6_) δ 8.39 (d, *J* = 7.8 Hz, 1H, H-N), 7.60 (dd, *J* = 8.0, 1.3 Hz, 1H), 7.38–7.26 (m, 1H),7.19–7.10 (m, 1H), 7.08 (dd, *J* = 7.8, 1.7 Hz, 1H), 4.28–4.17 (m, 1H), 2.44–2.34 (m, 1H, H-2), 2.20–2.12 (m, 2H), 1.94–1.80 (m, 2H), 1.75–1.71 (m, 1H, H-1), 1.65–1.56 (m, 2H), 1.36–1.27 (m, 1H, H-3), 1.25–1.21 (m, 1H, H-3). ^13^C NMR (100 MHz, DMSO-*d*_6_) δ 169.66 (C=O), 139.78, 132.29, 128.20, 127.92, 127.08, 125.06, 44.05, 30.49, 24.58, 24.35, 14.64, 14.05. HRMS (ESI) *m*/*z* [M + H]^+^ calcd for C_14_H_17_BrNO, 294.0488, found: 294.0499.
*2-(2-Bromophenyl)-N-cyclopentylcyclopropane-1-carboxamide* (**F3**): White solid, Yield: 70.7%, m.p. 164.0–165.3 °C. ^1^H NMR (400 MHz, DMSO-*d*_6_) δ 8.08 (d, *J* = 7.3 Hz, 1H, H-N), 7.60 (dd, *J* = 7.9, 1.3 Hz, 1H), 7.33–7.29 (m, 1H), 7.17–7.13 (m, 1H), 7.08 (dd, *J* = 7.7, 1.7 Hz, 1H), 4.08–3.99 (m, 1H), 2.41–2.36 (m, 1H, H-2), 1.86–1.73 (m, 3H), 1.67–1.58 (m, 2H), 1.55–1.44 (m, 2H), 1.43–1.30 (m, 3H), 1.24–1.20 (m, 1H, H-3). ^13^C NMR (100 MHz, DMSO-*d*_6_) δ 170.10 (C=O), 139.89, 132.26, 128.14, 127.88, 127.05, 125.10, 50.44, 32.48, 32.36, 24.61, 24.31, 23.43, 23.40, 13.85. HRMS (ESI) *m*/*z* [M + H]^+^ calcd for C_15_H_19_BrNO: 308.0645, found: 308.0654.
*2-(2-Bromophenyl)-N-cyclohexylcyclopropane-1-carboxamide* (**F4**): White solid, Yield: 76.0%, m.p. 166.0–167.2 °C. ^1^H NMR (400 MHz, DMSO-*d*_6_) δ 8.00 (d, *J* = 7.9 Hz, 1H, H-N), 7.60 (dd, *J* = 7.9, 1.3 Hz, 1H), 7.33–7.29 (m, 1H), 7.17–7.13 (m, 1H), 7.08 (dd, *J* = 7.8, 1.7 Hz, 1H), 3.62–3.51 (m, 1H), 2.41–2.36 (m, 1H, H-2), 1.87–1.66 (m, 5H), 1.56–1.52 (m, 1H), 1.34–1.30 (m, 1H, H-3),1.28–1.20 (m, 3H), 1.19–1.08 (m, 3H). ^13^C NMR (100 MHz, DMSO-*d*_6_) δ 169.71 (C=O), 139.90, 132.25, 128.14, 127.87, 127.07, 125.16, 47.59, 32.63, 32.56, 25.26, 24.65, 24.54, 24.39, 13.82. HRMS (ESI) *m*/*z* [M + H]^+^ calcd for C_16_H_21_BrNO: 322.0801, found: 322.0810.
*2-(2-Bromophenyl)-N-(p-tolyl)cyclopropane-1-carboxamide* (**F5**): White solid, Yield: 61.5%, m.p. 160.8–162.1 °C. ^1^H NMR (400 MHz, DMSO-*d*_6_) δ 10.43 (s, 1H, H-N), 7.88 (dd, *J* = 8.3, 1.2 Hz, 1H), 7.78–7.71 (m, 2H), 7.63–7.58 (m, 1H), 7.44 (t, *J* = 7.6 Hz, 2H), 7.39–7.32 (m, 2H), 2.82–2.79 (m, 1H, H-2), 2.78–2.75 (m, 3H), 2.28–2.19 (m, 1H, H-1), 1.74–1.63 (m, 2H, H-3). ^13^C NMR (100 MHz, DMSO-*d*_6_) δ169.54 (C=O), 139.48, 136.79, 132.31, 131.98, 129.15, 128.37, 127.94, 127.30, 125.20, 118.37, 25.48, 25.31, 20.45, 14.36. HRMS (ESI) *m*/*z* [M + H]^+^ calcd for C_17_H_17_BrNO: 330.0488, found: 330.0498.
*2-(2-Bromophenyl)-N-(m-tolyl)cyclopropane-1-carboxamide* (**F6**): White solid, Yield: 68.1%, m.p. 190.4–191.3 °C. ^1^H NMR (400 MHz, DMSO-*d*_6_) δ 9.59 (s, 1H, H-N), 7.64 (d, *J* = 7.9 Hz, 1H), 7.46 (d, *J* = 7.7 Hz, 1H), 7.36 (t, *J* = 7.4 Hz, 1H), 7.21–7.14 (m, 4H), 7.07 (t, *J* = 7.4 Hz, 1H), 2.23 (s, 3H), 2.13–2.07 (m, 1H, H-1), 1.50–1.38 (m, 2H, H-3). ^13^C NMR (100 MHz, DMSO-*d*_6_) δ 169.90 (C=O), 139.57, 136.40, 132.31, 131.31, 130.31, 128.40, 127.94, 127.46, 125.96, 125.39, 125.01, 124.79, 25.45, 24.91, 17.93, 13.98. HRMS (ESI) *m*/*z* [M + H]^+^ calcd for C_17_H_17_BrNO: 330.0488, found: 330.0497.
*2-(2-Bromophenyl)-N-(o-tolyl)cyclopropane-1-carboxamide* (**F7**): Pale yellow solid, Yield: 66.7%, m.p. 188.9–190.2 °C. ^1^H NMR (400 MHz, DMSO-*d*_6_) δ 9.60 (s, 1H, H-N), 7.63 (d, *J* = 7.9 Hz, 1H), 7.47 (d, *J* = 7.6 Hz, 1H), 7.35 (t, *J* = 7.6 Hz, 1H), 7.21–7.14 (m, 4H), 7.07 (t, *J* = 7.5 Hz, 1H), 2.23 (s, 3H), 2.16–2.04 (m, 1H, H-1), 1.49–1.41 (m, 2H, H-3). ^13^C NMR (100 MHz, DMSO-*d*_6_) δ 169.94 (C=O), 139.59, 136.42, 132.32, 131.33, 130.33, 128.40, 127.95, 127.48, 125.98, 125.42, 125.02, 124.82, 25.49, 24.93, 17.96, 14.01. HRMS (ESI) *m*/*z* [M + H]^+^ calcd for C_17_H_17_BrNO: 330.0488, found: 330.0498.
*Ethyl 4-(2-(2-bromophenyl)cyclopropane-1-carbonyl)piperazine-1-carboxylate* (**F8**): Pale yellow oil, Yield: 78.7%. ^1^H NMR (400 MHz, DMSO-*d*_6_) δ 7.60 (dd, *J* = 7.9, 1.3 Hz, 1H), 7.34–7.30 (m, 1H), 7.22–7.14 (m, 2H), 4.08–4.03 (m, 2H), 3.66 (d, *J* = 5.5 Hz, 2H), 3.56–3.46 (m, 2H), 3.42–3.40 (m, 2H), 3.36 (d, *J* = 4.0 Hz, 2H), 2.48–2.43 (m, 1H, H-2),2.25–2.14 (m, 1H, H-1), 1.49–1.38 (m, 1H, H-3), 1.45–1.40 (m, 1H, H-3), 1.19 (t, *J* = 7.1 Hz, 3H). ^13^C NMR (100 MHz, DMSO-*d*_6_) δ 169.70 (C=O), 154.62, 139.50, 132.23, 128.32, 127.83, 127.79, 125.34, 60.93, 44.78, 43.59, 43.25, 41.46, 25.86, 20.68, 15.01, 14.56. HRMS (ESI) *m*/*z* [M+Na]^+^ calcd for C_17_H_21_BrN_2_NaO_3_: 403.0628, found: 403.0636.
*2-(2-bromophenyl)-N-(thiazol-2-yl)cyclopropane-1-carboxamide* (**F9**): White solid, Yield: 71.3%, m.p. 161.7–163.3 °C. ^1^H NMR (400 MHz, DMSO-*d*_6_) δ 12.43 (s, 1H, NH, H-N), 7.63 (d, *J* = 6.3 Hz, 1H), 7.47 (s, 1H), 7.35 (d, *J* = 7.5 Hz, 1H), 7.29–7.12 (m, 3H), 2.67–2.54 (m, 1H, H-2), 2.19–2.12 (m, 1H, H-1), 1.56–1.52 (m, 2H, H-3). ^13^C NMR (100 MHz, DMSO-*d*_6_) δ 170.00 (C=O), 157.82, 138.87, 137.64, 132.33, 128.69, 127.96, 127.81, 125.45, 113.47, 26.64, 23.98, 14.99. HRMS (ESI) *m*/*z* [M + H]^+^ calcd for C_13_H_12_BrN_2_OS: 322.9848, found: 322.9857.
*N-cyclopropyl-2-(p-tolyl)cyclopropane-1-carboxamide* (**F10**): White solid, Yield: 51.6%, m.p. 153.7–155.3 °C. ^1^H NMR (400 MHz, DMSO-*d*_6_) δ 8.16 (d, *J* = 7.9 Hz, 1H, H-N), 7.06 (d, *J* = 7.7 Hz, 2H), 6.98 (d, *J* = 7.7 Hz, 2H), 2.67–2.60 (m, 1H, H-2), 2.24 (s, 3H), 2.19–2.17 (m, 1H, H-1), 1.75–1.64 (m, 1H, H-3), 1.34–1.21 (m, 1H, H-3), 1.17–1.05 (m, 1H), 0.64–0.55 (m, 2H), 0.42–0.35 (m, 2H). ^13^C NMR (100 MHz, DMSO-*d*_6_) δ 172.48 (C=O), 138.47, 135.36, 129.33, 126.15, 25.91, 24.00, 22.89, 21.04, 15.54, 6.18, 6.14. HRMS (ESI) *m*/*z* [M + H]^+^ calcd for C_14_H_18_NO: 216.1383, found: 216.1384.
*N-cyclobutyl-2-(p-tolyl)cyclopropane-1-carboxamide* (**F11**): White solid, Yield: 58.8%, m.p. 165.2–165.7 °C. ^1^H NMR (400 MHz, DMSO-*d*_6_) δ 8.34 (d, *J* = 7.8 Hz, 1H, H-N), 7.06 (d, *J* = 7.6 Hz, 2H), 6.98 (d, *J* = 7.7 Hz, 2H), 4.26–4.16 (m, 1H), 2.24 (s, 3H), 2.16–2.10 (m, 3H), 1.90–1.80 (m, 2H), 1.75–1.71 (m, 1H), 1.65–1.50 (m, 2H), 1.30–1.22 (m, 1H, H-3), 1.12 (t, *J* = 7.0 Hz, 1H). ^13^C NMR (100 MHz, DMSO-*d*_6_) δ 170.26 (C=O), 138.49, 135.35, 129.33, 126.15, 44.39, 31.00, 30.92, 25.97, 23.94, 21.04, 15.50, 15.04. HRMS (ESI) *m*/*z* [M+Na]^+^ calcd for C_15_H_19_NNaO: 252.1359, found: 252.1367.
*N-cyclopentyl-2-(p-tolyl)cyclopropane-1-carboxamide* (**F12**): Yellow solid, Yield: 50.6%, m.p. 176.1–176.8 °C. ^1^H NMR (400 MHz, DMSO-*d*_6_) δ 8.06 (d, *J* = 7.3 Hz, 1H, H-N), 7.06 (d, *J* = 7.2 Hz, 2H), 6.98 (d, *J* = 7.6 Hz, 2H), 4.05–3.94 (m, 1H), 2.24 (s, 3H), 2.18–2.13 (m, 1H, H-1), 1.85–1.71 (m, 3H), 1.66–1.55 (m, 2H), 1.48 (s, 2H), 1.39–1.24 (m, 3H), 1.14–1.04 (m, 1H). ^13^C NMR (100 MHz, DMSO-*d*_6_) δ 170.38 (C=O), 138.19, 134.90, 128.91, 125.71, 50.44, 32.54, 32.36, 25.57, 23.42, 20.63, 15.18. HRMS (ESI) *m*/*z* [M+Na]^+^ calcd for C_16_H_21_NNaO: 266.1515, found: 266.1523.
*N-cyclohexyl-2-(p-tolyl)cyclopropane-1-carboxamide* (**F13**): White solid, Yield: 63.4%, m.p. 186.7–188.5 °C. ^1^H NMR (400 MHz, DMSO-*d*_6_) δ 7.96 (d, *J* = 7.6 Hz, 1H, H-N), 7.07 (d, *J* = 7.5 Hz, 2H), 6.98 (d, *J* = 7.6 Hz, 2H), 3.62–3.45 (m, 1H), 2.24 (s, 3H), 2.17–2.09 (m, 1H, H-1), 1.90–1.59 (m, 5H), 1.53 (d, *J* = 11.3 Hz, 1H), 1.33–1.19 (m, 3H), 1.17–0.96 (m, 4H). ^13^C NMR (100 MHz, DMSO-*d*_6_) δ 169.88 (C=O), 138.17, 134.85, 128.86, 125.67, 47.57, 32.64, 32.54, 25.53, 25.24, 24.55, 23.36, 20.59, 15.18. HRMS (ESI) *m*/*z* [M + H]^+^ calcd for C_17_H_24_NO: 258.1852, found: 258.1860.
*N,2-di-p-tolylcyclopropane-1-carboxamide* (**F14**): White solid, Yield: 55.1%, m.p. 174.1–176.0 °C. ^1^H NMR (400 MHz, DMSO-*d*_6_) δ 10.13 (s, 1H, H-N), 7.47 (d, *J* = 7.9 Hz, 2H), 7.08 (dd, *J* = 11.0, 6.6 Hz, 6H), 2.35–2.28 (m, 1H, H-2), 2.26 (s, 3H), 2.24 (s, 3H), 2.04–1.97 (m, 1H, H-1), 1.46–1.43 (m, 1H, H-3), 1.31–1.26 (m, 1H, H-3). ^13^C NMR (100 MHz, DMSO-*d*_6_) δ 169.71 (C=O), 137.75, 136.82, 135.10, 131.87, 129.11, 128.92, 125.82, 118.89, 26.47, 24.48, 20.61, 20.44, 15.47. HRMS (ESI) *m*/*z* [M + H]^+^ calcd for C_18_H_20_NO: 266.1539, found: 266.1546.
*N-(m-tolyl)-2-(p-tolyl)cyclopropane-1-carboxamide* (**F15**): White solid, Yield: 56.4%, m.p. 163.9–165.9 °C. ^1^H NMR (400 MHz, DMSO-*d*_6_) δ 9.52 (s, 1H, H-N), 7.48 (d, *J* = 8.0 Hz, 1H), 7.19 (d, *J* = 7.4 Hz, 1H), 7.14–7.03 (m, 6H), 2.31 (d, *J* = 3.5 Hz, 1H, H-2), 2.27 (s, 3H), 2.18–2.11 (m, 4H), 1.44–1.40 (m, 1H, H-3), 1.32–1.21 (m, 1H, H-3). ^13^C NMR (100 MHz, DMSO-*d*_6_) δ 170.11 (C=O), 137.88, 136.47, 135.10, 131.02, 130.32, 128.95, 125.94, 125.86, 124.87, 124.59, 25.84, 24.37, 20.64, 17.92, 15.71. HRMS (ESI) *m*/*z* [M + H]^+^ calcd for C_18_H_20_NO: 266.1539, found: 266.1548.
*N-(o-tolyl)-2-(p-tolyl)cyclopropane-1-carboxamide* (**F16**): White solid, Yield: 58.8%, m.p. 165.1–166.2 °C. ^1^H NMR (400 MHz, DMSO-*d*_6_) δ 9.52 (s, 1H, H-N), 7.48 (d, *J* = 7.9 Hz, 1H), 7.19 (d, *J* = 7.4 Hz, 1H), 7.14–7.03 (m, 6H), 2.35–2.29 (m, 1H, H-2), 2.27 (s, 3H), 2.20 (s, 4H), 1.45–1.40 (m, 1H, H-3), 1.31–1.22 (m, 1H, H-3). ^13^C NMR (100 MHz, DMSO-*d*_6_) δ 169.77 (C=O), 137.86, 136.86, 135.08, 131.00, 130.30, 128.93, 125.92, 125.84, 124.85, 124.56, 25.81, 24.36, 20.62, 17.90, 15.68. HRMS (ESI) *m*/*z* [M + H]^+^ calcd for C_18_H_20_NO: 266.1539, found: 266.1548.
*N-(thiazol-2-yl)-2-(p-tolyl)cyclopropane-1-carboxamide* (**F17**): Pale yellow solid, Yield: 55.3%, m.p. 188.9–190.8 °C. ^1^H NMR (400 MHz, DMSO-*d*_6_) δ 12.36 (s, 1H, H-N), 7.45 (d, *J* = 3.5 Hz, 1H), 7.18 (d, *J* = 3.6 Hz, 1H), 7.11–7.01 (m, 4H), 2.45–2.40 (m, 1H, H-2), 2.26 (s, 3H), 2.21–2.16 (m, 1H), 1.54–1.49 (m, 1H, H-3), 1.46–1.38 (m, 1H, H-3). ^13^C NMR (100 MHz, DMSO-*d*_6_) δ 170.12 (C=O), 158.00, 137.64, 137.08, 135.44, 129.02, 126.03, 113.41, 25.72, 25.11, 20.64, 16.23. HRMS (ESI) *m*/*z* [M + H]^+^ calcd for C_14_H_15_N_2_OS: 259.0900, found: 259.0908.
*N-cyclopropyl-2-(4-methoxyphenyl)cyclopropane-1-carboxamide* (**F18**): White solid, Yield: 54.8%, m.p. 158.1–160.0 °C. ^1^H NMR (400 MHz, DMSO-*d*_6_) δ 8.15 (d, *J* = 4.2 Hz, 1H, H-N), 7.08–6.94 (m, 2H), 6.82 (d, *J* = 8.4 Hz, 2H), 3.70 (s, 3H), 2.68–2.54 (m, 1H, H-2), 2.23–2.15 (m, 1H, H-1), 1.69–1.65 (m, 1H, H-3), 1.30–1.20 (m, 1H, H-3), 1.15–0.98 (m, 1H), 0.62–0.53 (m, 2H), 0.43–0.25 (m, 2H). ^13^C NMR (100 MHz, DMSO-*d*_6_) δ 172.11 (C=O), 157.64, 132.87, 126.90, 113.78, 55.06, 25.31, 23.22, 22.44, 14.88, 5.74, 5.69. HRMS (ESI) *m*/*z* [M + H]^+^ calcd for C_14_H_18_NO_2_: 232.1332, found: 232.1339.
*N-cyclobutyl-2-(4-methoxyphenyl)cyclopropane-1-carboxamide* (**F19**): White solid, Yield: 72.2%, m.p. 188.3–189.8 °C. ^1^H NMR (400 MHz, DMSO-*d*_6_) δ 8.32 (d, *J* = 7.9 Hz, 1H, H-N), 7.03 (d, *J* = 8.4 Hz, 2H), 6.92–6.74 (m, 2H), 4.26–4.12 (m, 1H), 3.71 (s, 3H), 2.21–2.06 (m, 3H), 1.90–1.80 (m, 2H), 1.71–1.65 (m, 1H), 1.63–1.54 (m, 2H), 1.28–1.24 (m, 1H, H-3), 1.11–1.07 (m, 1H). ^13^C NMR (100 MHz, DMSO-*d*_6_) δ 169.89 (C=O), 157.64, 132.89, 126.89, 113.78, 55.06, 43.94, 30.56, 30.48, 25.36, 23.16, 14.84, 14.58. HRMS (ESI) *m*/*z* [M + H]^+^ calcd for C_15_H_20_NO_2_): 246.1489, found: 246.1495.
*N-cyclopentyl-2-(4-methoxyphenyl)cyclopropane-1-carboxamide* (**F20**): White solid, Yield: 69.6%, m.p. 194.6–196.0 °C. ^1^H NMR (400 MHz, DMSO-*d*_6_) δ 8.10–7.93 (m, 1H, H-N), 7.16–7.01 (m, 2H), 6.85–6.81 (m, 2H), 4.08–3.93 (m, 1H), 3.78–3.65 (m, 3H), 2.17–2.12 (m, 1H, H-2), 1.86–1.69 (m, 3H), 1.67–1.56 (m, 2H), 1.54–1.42 (m, 2H), 1.38–1.32 (m, 2H), 1.26 (dd, *J* = 9.2, 3.8 Hz, 1H), 1.09–1.04 (m, 1H). ^13^C NMR (100 MHz, DMSO-*d*_6_) δ 170.39 (C=O), 157.62, 133.02, 126.86, 113.78, 55.06, 50.39, 32.52, 32.33, 25.37, 23.39, 23.02, 14.92. HRMS (ESI) *m*/*z* [M+Na]^+^ calcd for C_16_H_21_NNaO_2_: 282.1465, found: 282.1474.
*N-cyclohexyl-2-(4-methoxyphenyl)cyclopropane-1-carboxamide* (**F21**): White solid, Yield: 68.3%, m.p. 142.8–144.0 °C. ^1^H NMR (400 MHz, DMSO-*d*_6_) δ 7.95 (d, *J* = 7.9 Hz, 1H, H-N), 7.02 (d, *J* = 8.3 Hz, 2H), 6.83 (d, *J* = 8.3 Hz, 2H), 3.70 (s, 3H), 3.58–3.47 (m, 1H), 2.22–2.08 (m, 1H, H-2), 1.81–1.60 (m, 5H), 1.58–1.48 (m,1H, H-3), 1.31–1.18 (m,3H), 1.18–1.02 (m, 4H). ^13^C NMR (100 MHz, DMSO-*d*_6_) δ 170.00 (C=O), 157.63, 133.03, 126.87, 113.79, 55.08, 47.59, 32.66, 32.56, 25.38, 25.26, 24.57, 23.05, 14.99. HRMS (ESI) *m*/*z* [M + H]^+^ calcd for C_17_H_24_NO_2_: 274.1802, found: 274.1809.
*2-(4-Methoxyphenyl)-N-(p-tolyl)cyclopropane-1-carboxamide* (**F22**): White solid, Yield: 74.4%, m.p. 171.4–173.3 °C. ^1^H NMR (400 MHz, DMSO-*d*_6_) δ 10.11 (s, 1H, H-N), 7.47 (d, *J* = 8.4 Hz, 2H), 7.16–7.01 (m, 4H), 6.85 (d, *J* = 8.7 Hz, 2H), 3.72 (s, 3H), 2.33–2.26 (m, 1H, H-2), 2.23 (s, 3H), 2.02–1.91 (m,1H, H-1), 1.43–1.39 (m, 1H, H-3), 1.28–1.23 (m, 1H, H-3). ^13^C NMR (100 MHz, DMSO-*d*_6_) δ 169.80 (C=O), 157.77, 136.84, 132.61, 131.86, 129.12, 127.04, 118.89, 113.84, 55.09, 26.34, 24.18, 20.45, 15.29. HRMS (ESI) *m*/*z* [M + H]^+^ calcd for C_18_H_20_NO_2_: 282.1489, found: 282.1496.
*2-(4-Methoxyphenyl)-N-(m-tolyl)cyclopropane-1-carboxamide* (**F23**): White solid, Yield: 80.5%, m.p. 134.4–135.8 °C. ^1^H NMR (400 MHz, DMSO-*d*_6_) δ 9.51 (s, 1H, H-N), 7.47 (d, *J* = 7.7 Hz, 1H), 7.22–7.17 (m, 1H), 7.15 (dd, *J* = 7.6, 1.7 Hz, 1H), 7.13–7.08 (m, 2H), 7.08–7.01 (m, 1H), 6.90–6.83 (m, 2H), 3.72 (s, 3H), 2.33–2.28 (m, 1H, H-2), 2.20 (s, 3H), 2.17–2.12 (m, 1H, H-1), 1.44–1.35 (m, 1H, H-3), 1.28–1.20 (m, 1H, H-3). ^13^C NMR (100 MHz, DMSO-*d*_6_) δ 170.16 (C=O), 157.76, 136.48, 132.71, 131.00, 130.30, 127.05, 125.92, 124.83, 124.57, 113.85, 55.10, 25.68, 24.05, 17.90, 15.48. HRMS (ESI) *m*/*z* [M + H]^+^ calcd for C_18_H_20_NO_2_: 282.1489, found: 282.1499.
*2-(4-Methoxyphenyl)-N-(o-tolyl)cyclopropane-1-carboxamide* (**F24**): White solid, Yield: 75.4%, m.p. 135.1–136.2 °C. ^1^H NMR (400 MHz, DMSO-*d*_6_) δ 9.51 (s, 1H, H-N), 7.49 (d, *J* = 7.7 Hz, 1H), 7.19 (d, *J* = 7.3 Hz, 1H), 7.11 (d, *J* = 8.7 Hz, 3H), 7.05 (s, 1H), 6.87 (d, *J* = 8.2 Hz, 2H), 3.72 (s, 3H), 2.36–2.29 (m, 1H, H-2), 2.3 (s, 3H), 2.18–2.12 (m, 1H, H-1), 1.43–1.38 (m, 1H, H-3), 1.28–1.23 (m, 1H, H-3). ^13^C NMR (100 MHz, DMSO-*d*_6_) δ 170.16 (C=O), 157.77, 136.49, 132.73, 131.00, 130.30, 127.05, 125.93, 124.84, 124.57, 113.85, 55.10, 25.67, 24.05, 17.91, 15.49. HRMS (ESI) *m*/*z* [M + H]^+^ calcd for C_18_H_20_NO_2_: 282.1489, found: 282.1498.
*Ethyl 4-(2-(4-methoxyphenyl)cyclopropane-1-carbonyl)piperazine-1-carboxylate* (**F25**): Yellow oil, Yield: 54.9%. ^1^H NMR (400 MHz, DMSO-*d*_6_) δ 7.16–7.02 (m, 2H), 6.89–6.76 (m, 2H), 4.08 –4.01 (m, 2H), 3.72 (s, 3H), 3.63 (d, *J* = 16.3 Hz, 2H), 3.50 (s, 2H), 3.37 (d, *J* = 10.1 Hz, 4H), 2.34–2.12 (m, 2H), 1.39 –1.32 (m, 1H, H-3), 1.25–1.11 (m, 4H). ^13^C NMR (100 MHz, DMSO-*d*_6_) δ 169.95 (C=O), 157.74, 154.63, 132.59, 127.17, 113.79, 60.92, 55.06, 44.65, 43.64, 43.12, 41.37, 24.18, 22.29, 15.96, 14.56. HRMS (ESI) *m*/*z* [M + H]^+^ calcd for C_18_H_24_N_2_NaO_4_: 355.1628, found: 355.1638.
*2-(4-Methoxyphenyl)-N-(thiazol-2-yl)cyclopropane-1-carboxamide* (**F26**): White solid, Yield: 74.7%, m.p. 172.9–174.5 °C. ^1^H NMR (400 MHz, DMSO-*d*_6_) δ 12.35 (s, 1H, H-N), 7.45 (d, *J* = 3.6 Hz, 1H), 7.18 (d, *J* = 3.6 Hz, 1H), 7.14–7.08 (m, 2H), 6.90–6.82 (m, 2H), 3.72 (s, 3H), 2.47–2.39 (m, 1H, H-2), 2.17–2.13 (m, 1H, H-1), 1.51–1.47 (m, 1H, H-3), 1.43–1.38 (m, 1H, H-3). ^13^C NMR (100 MHz, DMSO-*d*_6_) δ 170.17 (C=O), 158.99, 158.01, 157.95, 137.63, 131.92, 127.29, 113.89, 113.37, 55.11, 25.44, 24.98, 16.04. HRMS (ESI) *m*/*z* [M + H]^+^ calcd for C_14_H_15_N_2_O_2_S: 275.0849, found: 275.0857.
*N-cyclopropyl-2-(m-tolyl)cyclopropane-1-carboxamide* (**F27**): Yellow oil, Yield: 66.0%. ^1^H NMR (400 MHz, DMSO-*d*_6_) δ 8.17 (d, *J* = 3.9 Hz, 1H, H-N), 7.13 (t, *J* = 7.5 Hz, 1H), 6.97 (d, *J* = 7.5 Hz, 1H), 6.92–6.84 (m, 2H), 2.70–2.61 (m, 1H, H-2), 2.25 (s, 3H), 2.20–2.13 (m, 1H, H-1), 1.76–1.71 (m, 1H, H-3), 1.37–1.27 (m, 1H, H-3), 1.19–1.10 (m, 1H), 0.64–0.55 (m, 2H), 0.41–0.34 (m, 2H). ^13^C NMR (100 MHz, DMSO-*d*_6_) δ 172.00 (C=O), 141.04, 137.41, 128.22, 126.60, 126.40, 122.94, 25.58, 23.81, 22.45, 20.99, 15.10, 5.72. HRMS (ESI) *m*/*z* [M + H]^+^ calcd for C_14_H_18_NO: 216.1383, found: 216.1391.
*N-cyclobutyl-2-(m-tolyl)cyclopropane-1-carboxamide* (**F28**): White solid, Yield: 56.9%, m.p. 146.6–148.3 °C. ^1^H NMR (400 MHz, DMSO-*d*_6_) δ 8.34 (d, *J* = 7.9 Hz, 1H, H-N), 7.14 (t, *J* = 7.5 Hz, 1H), 6.97 (d, *J* = 7.4 Hz, 1H), 6.93–6.84 (m, 2H), 4.26–4.16 (m, 1H), 2.25 (s, 3H), 2.20–2.07 (m, 3H), 1.91–1.79 (m, 2H), 1.79–1.72 (m, 1H, H-1), 1.63–1.54 (m, 2H), 1.33–1.25 (m, 1H), 1.19–1.09 (m, 1H). ^13^C NMR (100 MHz, DMSO-*d*_6_) δ 169.77 (C=O), 141.06, 137.41, 128.22, 126.59, 126.38, 122.94, 43.95, 30.54, 30.48, 25.64, 23.75, 20.99, 15.05, 14.59. HRMS (ESI) *m*/*z* [M + H]^+^ calcd for C_15_H_20_NO: 230.1539, found: 230.1548.
*N-cyclopentyl-2-(m-tolyl)cyclopropane-1-carboxamide* (**F29**): White solid, Yield: 58.7%, m.p. 140.9–142.2 °C. ^1^H NMR (400 MHz, DMSO-*d*_6_) δ 8.06 (d, *J* = 7.3 Hz, 1H, H-N), 7.14 (t, *J* = 7.5 Hz, 1H), 6.97 (d, *J* = 7.5 Hz, 1H), 6.89 (d, *J* = 8.5 Hz, 2H), 4.05–3.93 (m, 1H), 2.25 (s, 3H), 2.18–2.13 (m, 1H, H-2), 1.86–1.71 (m, 3H), 1.67–1.57 (m, 2H), 1.55–1.44 (m, 2H), 1.39–1.24 (m, 3H), 1.15–1.10 (m, 1H). ^13^C NMR (100 MHz, DMSO-*d*_6_) δ 170.27 (C=O), 141.21, 137.41, 128.22, 126.56, 126.34, 122.93, 50.41, 32.51, 32.35, 25.68, 23.62, 23.39, 21.01, 15.17. HRMS (ESI) *m*/*z* [M + H]^+^ calcd for C_16_H_22_NO: 244.1696, found: 244.1711.
*N-cyclohexyl-2-(m-tolyl)cyclopropane-1-carboxamide* (**F30**): White solid, Yield: 63.7%, m.p. 159.8–162.4 °C. ^1^H NMR (400 MHz, DMSO-*d*_6_) δ 7.97 (d, *J* = 7.9 Hz, 1H, H-N), 7.14 (s, 1H), 6.97 (d, *J* = 7.5 Hz, 1H), 6.89 (d, *J* = 9.2 Hz, 2H), 3.60–3.49 (m, 1H), 2.25 (s, 3H), 2.18–2.13 (m, 1H, H-2), 1.87–1.78 (m, 1H, H-1), 1.76–1.59 (m, 4H), 1.58–1.48 (m, 1H, H-3), 1.33–1.18 (m, 3H), 1.17–1.03 (m, 4H). ^13^C NMR (100 MHz, DMSO-*d*_6_) δ 169.86 (C=O), 141.21, 137.41, 128.23, 126.56, 126.36, 122.92, 47.59, 32.65, 32.55, 25.69, 25.26, 24.56, 23.64, 21.02, 15.24. HRMS (ESI) *m*/*z* [M + H]^+^ calcd for C_17_H_24_NO: 258.1852, found: 258.1858.
*2-(m-Tolyl)-N-(p-tolyl)cyclopropane-1-carboxamide* (**F31**): White solid, Yield: 57.1%, m.p. 132.0–133.4 °C. ^1^H NMR (400 MHz, DMSO-*d*_6_) δ 10.14 (s, 1H, H-N), 7.52–7.43 (m, 2H), 7.16 (d, *J* = 7.5 Hz, 1H), 7.09 (d, *J* = 8.3 Hz, 2H), 7.01–6.90 (m, 3H), 2.33–2.28 (m, 1H, H-2), 2.27 (s, 3H), 2.24 (s, 3H), 2.07–1.99 (m, 1H, H-1), 1.48–1.44 (m, 1H, H-3), 1.44–1.27 (m, 1H, H-3). ^13^C NMR (100 MHz, DMSO-*d*_6_) δ 169.69 (C=O), 140.80, 137.50, 136.83, 131.91, 129.15, 128.29, 126.78, 126.46, 123.10, 118.90, 26.63, 24.75, 21.02, 20.47, 15.52. HRMS (ESI) *m*/*z* [M + H]^+^ calcd for C_18_H_20_NO: 266.1539, found: 266.1547.
*N,2-Di-m-tolylcyclopropane-1-carboxamide* (**F32**): White solid, Yield: 59.8%, m.p. 150.8–151.9 °C. ^1^H NMR (400 MHz, DMSO-*d*_6_) δ 9.52 (s, 1H, H-N), 7.48 (d, *J* = 7.7 Hz, 1H), 7.17 (dd, *J* = 16.1, 7.5 Hz, 3H), 7.05 (s, 1H), 7.02–6.92 (m, 3H), 2.36–2.28 (m, 4H), 2.25–2.20 (m, 4H), 1.46–1.41 (m, 1H, H-3), 1.36–1.23 (m, 1H, H-3). ^13^C NMR (100 MHz, DMSO-*d*_6_) δ 170.07 (C=O), 140.91, 137.50, 136.46, 130.98, 130.32, 128.30, 126.76, 126.47, 125.95, 124.87, 124.55, 123.11, 25.96, 24.61, 21.05, 17.93, 15.78. HRMS (ESI) *m*/*z* [M + H]^+^ calcd for C_18_H_20_NO: 266.1539, found: 266.1546.
*2-(m-Tolyl)-N-(o-tolyl)cyclopropane-1-carboxamide* (**F33**): White solid, Yield: 60.5%, m.p. 150.5–151.5 °C. ^1^H NMR (400 MHz, DMSO-*d*_6_) δ 9.52 (s, 1H, H-N), 7.48 (d, *J* = 8.0 Hz, 1H), 7.21–7.12 (m, 3H), 7.02 (d, *J* = 28.5 Hz, 4H), 2.29 (d, *J* = 9.4 Hz, 4H), 2.20 (s, 4H), 1.48–1.39 (m, 1H, H-3), 1.35–1.24 (m, 1H, H-3). ^13^C NMR (100 MHz, DMSO-*d*_6_) δ 170.07 (C=O), 140.92, 137.50, 136.48, 130.99, 130.33, 128.30, 126.76, 126.48, 125.95, 124.87, 124.55, 123.11, 25.97, 24.62, 21.05, 17.93, 15.79. HRMS (ESI) *m*/*z* [M+Na]^+^ calcd for C_18_H_19_NNaO: 288.1359, found: 288.1367.
*Ethyl 4-(2-(m-tolyl)cyclopropane-1-carbonyl)piperazine-1-carboxylate* (**F34**): Yellow oil, Yield: 67.2%. ^1^H NMR (400 MHz, DMSO-*d*_6_) δ 7.17–7.12 (m, 1H), 7.02–6.92 (m, 3H), 4.10–3.98 (m, 2H), 3.72–3.57 (m, 2H), 3.56–3.40 (m, 4H), 2.29–2.24 (m, 5H), 1.45–1.33 (m, 1H, H-3), 1.27–1.12 (m, 4H). ^13^C NMR (100 MHz, DMSO-*d*_6_) δ 169.85 (C=O), 154.63, 140.76, 137.45, 128.21, 126.72, 126.59, 123.23, 60.94, 44.67, 43.59, 43.05, 41.39, 24.74, 22.09, 21.03, 14.57. HRMS (ESI) *m*/*z* [M+Na]^+^ calcd for C_18_H_24_N_2_NaO_3_: 339.1679, found: 339.1688.
*N-(thiazol-2-yl)-2-(m-tolyl)cyclopropane-1-carboxamide* (**F35**): White solid, Yield: 50.8%, m.p. 152.8–154.9 °C. ^1^H NMR (400 MHz, DMSO-*d*_6_) δ 12.34 (s, 1H, H-N), 7.45 (d, *J* = 3.6 Hz, 1H), 7.22–7.10 (m, 2H), 7.01 (d, *J* = 9.0 Hz, 2H), 6.97 (s, 1H), 2.45–2.37 (m, 1H, H-2), 2.27 (s, 3H), 2.24–2.17 (m, 1H, H-1), 1.55–1.50 (m, 1H, H-3), 1.48–1.43 (m, 1H, H-3). ^13^C NMR (100 MHz, DMSO-*d*_6_) δ 170.07 (C=O), 157.98, 140.07, 137.65, 137.27, 128.37, 127.05, 126.79, 123.22, 113.43, 25.94, 25.17, 21.02, 16.19. HRMS (ESI) *m*/*z* [M + H]^+^ calcd for C_14_H_15_N_2_OS: 259.0900, found: 259.0909.
*N-cyclopropyl-2-(4-fluorophenyl)cyclopropane-1-carboxamide* (**F36**): White solid, Yield: 51.4%, m.p. 152.5–153.5 °C. ^1^H NMR (400 MHz, DMSO-*d*_6_) δ 8.19 (d, *J* = 7.9 Hz, 1H, H-N), 7.15 (dd, *J* = 8.7, 5.6 Hz, 2H), 7.11–7.04 (m, 2H), 2.67–2.60 (m, 1H, H-2), 2.247–2.22 (m, 1H, H-1), 1.76–1.67 (m, 1H, H-3), 1.37–1.28 (m, 1H, H-3), 1.18–1.13 (m, 1H), 0.62–0.55 (m, 2H), 0.41–0.35 (m, 2H). ^13^C NMR (100 MHz, DMSO-*d*_6_) δ 171.89 (C=O), 160.71 (*J*_C-F_= 241.5 Hz), 137.26, 127.63 (*J*_C-F_ = 8.0 Hz, 2C), 115.06 (*J*_C-F_ = 21.2 Hz, 2C), 25.68, 23.13, 22.47, 15.16, 5.75, 5.72. ^19^F NMR (376 MHz, DMSO-*d*_6_) δ-117.09. HRMS (ESI) *m*/*z* [M+Na]^+^ calcd for C_13_H_14_FNNaO: 242.0952, found: 242.0960.
*N-cyclobutyl-2-(4-fluorophenyl)cyclopropane-1-carboxamide* (**F37**): White solid, Yield: 62.9%, m.p. 170.2–172.0 °C. ^1^H NMR (400 MHz, DMSO-*d*_6_) δ 8.36 (d, *J* = 7.9 Hz, 1H, H-N), 7.15 (dd, *J* = 8.6, 5.6 Hz, 2H), 7.08 (t, *J* = 8.9 Hz, 2H), 4.26–4.16 (m, 1H), 2.25–2.20 (m, 1H, H-2), 2.17–2.11 (m, 2H), 1.90–1.80 (m, 2H), 1.76–1.72 (m, 1H, H-3), 1.67–1.51 (m, 2H), 1.32–1.28 (m, 1H, H-3), 1.22–1.12 (m, 1H). ^13^C NMR (100 MHz, DMSO-*d*_6_) δ 169.67 (C=O), 160.71 (*J*_C-F_ = 240.5 Hz), 137.31, 127.62 (*J*_C-F_ = 8.1 Hz, 2C), 115.06 (*J*_C-F_ = 21.3 Hz, 2C), 43.98, 30.56, 30.48, 25.74, 23.07, 15.12, 14.61. ^19^F NMR (376 MHz, DMSO-*d*_6_) δ-117.17. HRMS (ESI) *m*/*z* [M + H]^+^ calcd for C_14_H_17_FNO: 234.1289, found: 234.1296.
*N-cyclopentyl-2-(4-fluorophenyl)cyclopropane-1-carboxamide* (**F38**): White solid, Yield: 51.8%, m.p. 166.1–167.6 °C. ^1^H NMR (400 MHz, DMSO-*d*_6_) δ 8.06 (d, *J* = 7.3 Hz, 1H, H-N), 7.15 (dd, *J* = 8.6, 5.6 Hz, 2H), 7.08 (t, *J* = 8.9 Hz, 2H), 4.05–3.97 (m, 1H), 2.25–2.20 (m, 1H, H-2), 1.88–1.72 (m, 3H), 1.66–1.57 (m, 2H), 1.53–1.46 (m, 2H), 1.41–1.26 (m, 3H), 1.15–1.11 (m, 1H). ^13^C NMR (100 MHz, DMSO-*d*_6_) δ 170.16 (C=O), 160.69 (*J*_C-F_ = 241.4 Hz), 137.43, 127.58 (*J*_C-F_ = 7.9 Hz, 2C), 115.04 (*J*_C-F_ = 21.1 Hz, 2C), 50.44, 32.53, 32.33, 25.75, 23.41, 22.94, 15.20. ^19^F NMR (376 MHz, DMSO-*d*_6_) δ-117.21. HRMS (ESI) *m*/*z* [M + H]^+^ calcd for C_15_H_19_FNO: 248.1445, found: 248.1455.
*N-cyclohexyl-2-(4-fluorophenyl)cyclopropane-1-carboxamide* (**F39**): White solid, Yield: 72.4%, m.p. 188.2–190.1 °C. ^1^H NMR (400 MHz, DMSO-*d*_6_) δ 7.97 (d, *J* = 7.8 Hz, 1H, H-N), 7.14 (dd, *J* = 8.4, 5.5 Hz, 2H), 7.08 (t, *J* = 8.7 Hz, 2H), 3.54 (d, *J* = 7.6 Hz, 1H), 2.27–2.18 (m, 1H, H-2), 1.84–1.80 (m, 1H, H-2), 1.77–1.61 (m, 4H), 1.53 (d, *J* = 12.9 Hz, 1H), 1.36–1.19 (m, 3H), 1.17–1.07 (m, 4H). ^13^C NMR (100 MHz, DMSO-*d*_6_) δ 169.75 (C=O), 160.70 (*J*_C-F_ = 241.5 Hz), 137.43, 127.58 (*J*_C-F_ = 8.0 Hz, 2C), 115.04 (*J*_C-F_ = 21.1 Hz, 2C), 47.63, 32.66, 32.54, 25.74, 25.26, 24.58, 24.55, 22.95, 15.27. ^19^F NMR (376 MHz, DMSO-*d*_6_) δ-117.24. HRMS (ESI) *m*/*z* [M + H]^+^ calcd for C_16_H_21_FNO: 262.1602, found: 262.1611.
*2-(4-Fluorophenyl)-N-(p-tolyl)cyclopropane-1-carboxamide* (**F40**): White solid, Yield: 65.9%, m.p. 181.9–183.4 °C. ^1^H NMR (400 MHz, DMSO-*d*_6_) δ 10.13 (s, 1H, H-N), 7.47 (d, *J* = 8.0 Hz, 2H), 7.22 (dd, *J* = 8.5, 5.4 Hz, 2H), 7.11 (q, *J* = 8.4 Hz, 4H), 2.41–2.32 (m, 1H, H-2), 2.24 (s, 3H), 2.04–1.98 (m, 1H, H-2), 1.48–1.43 (m, 1H, H-3), 1.34–1.30 (m, 1H, H-3). ^13^C NMR (100 MHz, DMSO-*d*_6_) δ 169.57 (C=O), 160.82 (*J*_C-F_ = 241.7 Hz), 137.04, 136.80, 131.97, 129.16 (2C), 127.79 (*J*_C-F_ = 8.0 Hz, 2C), 118.94, 115.12 (*J*_C-F_ = 21.3 Hz, 2C), 26.67, 24.04, 20.47, 15.54. ^19^F NMR (376 MHz, DMSO-*d*_6_) δ-116.92. HRMS (ESI) *m*/*z* [M + H]^+^ calcd for C_17_H_17_FNO: 270.1289, found: 270.1296.
*2-(4-Fluorophenyl)-N-(m-tolyl)cyclopropane-1-carboxamide* (**F41**): White solid, Yield: 73.2%, m.p. 157.4–159.0 °C. ^1^H NMR (400 MHz, DMSO-*d*_6_) δ 9.54 (s, 1H, H-N), 7.48 (d, *J* = 7.9 Hz, 1H), 7.23–7.01 (m, 7H), 2.37 (s, 1H, H-2), 2.20 (s, 4H), 1.45–1.31 (m, 2H, H-3). ^13^C NMR (100 MHz, DMSO-*d*_6_) δ 169.97 (C=O), 160.84 (*J*_C-F_ = 242.7 Hz), 137.13, 136.44, 131.06, 130.35, 127.81 (*J*_C-F_ = 8.0 Hz, 2C), 125.98, 124.94, 124.61, 115.14 (*J*_C-F_ = 21.3 Hz, 2C), 25.99, 23.91, 17.93, 15.77. ^19^F NMR (376 MHz, DMSO-*d*_6_) δ-116.97. HRMS (ESI) *m*/*z* [M + H]^+^ calcd for C_17_H_17_FNO: 270.1289, found: 270.1297.
*2-(4-Fluorophenyl)-N-(o-tolyl)cyclopropane-1-carboxamide* (**F42**): White solid, Yield: 74.2%, m.p. 158.0–159.9 °C. ^1^H NMR (400 MHz, DMSO-*d*_6_) δ 9.54 (s, 1H, H-N), 7.48 (d, *J* = 7.6 Hz, 1H), 7.34–6.83 (m, 7H), 2.40–2.36 (m, 1H, H-2), 2.20 (s, 4H), 1.47–1.23 (m, 2H, H-3). ^13^C NMR (100 MHz, DMSO-*d*_6_) δ 169.94 (C=O), 160.82 (*J*_C-F_ = 241.3 Hz), 137.13, 136.44, 131.03, 130.33, 127.80 (*J*_C-F_= 8.0 Hz, 2C), 125.96, 124.92, 124.58, 115.13 (*J*_C-F_ = 21.2 Hz, 2C), 25.98, 23.90, 17.92, 15.75. ^19^F NMR (376 MHz, DMSO-*d*_6_) δ-116.97. HRMS (ESI) *m*/*z* [M+Na]^+^ calcd for C_17_H_16_FNNaO: 292.1108, found: 292.1118.
*Ethyl 4-(2-(4-fluorophenyl)cyclopropane-1-carbonyl)piperazine-1-carboxylate* (**F43**): Yellow oil, Yield: 73.3%. ^1^H NMR (400 MHz, DMSO-*d*_6_) δ 7.22 (dd, *J* = 8.6, 5.6 Hz, 2H), 7.09 (t, *J* = 8.8 Hz, 2H), 4.04 (q, *J* = 7.1 Hz, 2H), 3.67–3.56 (m, 2H), 3.52–3.43 (m, 4H), 3.35–3.27 (m, 2H), 2.38–2.24 (m, 2H), 1.41–1.35 (m, 1H, H-3), 1.22–1.16 (m, 4H). ^13^C NMR (100 MHz, DMSO-*d*_6_) δ 169.77 (C=O), 160.81 (*J*_C-F_ = 241.6 Hz), 154.66, 136.99, 127.94 (*J*_C-F_ = 8.0 Hz, 2C), 115.05 (*J*_C-F_ = 21.2 Hz, 2C), 60.96, 44.67, 43.56, 43.07, 41.42, 24.01, 22.07, 16.40, 14.59. ^19^F NMR (376 MHz, DMSO-*d*_6_) δ-117.08. HRMS (ESI) *m*/*z* [M+Na]^+^ calcd for C_17_H_22_FN_2_O_3_: 343.1428, found: 343.1438.
*2-(4-Fluorophenyl)-N-(thiazol-2-yl)cyclopropane-1-carboxamide* (**F44**): Pale yellow solid, Yield: 70.4%, m.p. 187.0–189.3 °C. ^1^H NMR (400 MHz, DMSO-*d*_6_) δ 12.38 (s, 1H, H-N), 7.45 (s, 1H), 7.36–6.97 (m, 5H), 2.20 (s, 1H, H-1), 1.53–1.46 (m, 2H, H-3). ^13^C NMR (100 MHz, DMSO-*d*_6_) δ 169.95 (C=O), 160.96 (*J*_C-F_ = 242.4 Hz), 157.98, 137.66, 136.34, 128.05 (*J*_C-F_ = 8.0 Hz, 2C), 115.20 (d, *J* = 21.2 Hz), 113.45, 25.20, 16.24. ^19^F NMR (376 MHz, DMSO-*d*_6_) δ-116.54. HRMS (ESI) *m*/*z* [M + H]^+^ calcd for C_13_H_12_FN_2_OS: 263.0649, found: 263.0657.
*2-(4-Chlorophenyl)-N-cyclopropylcyclopropane-1-carboxamide* (**F45**): White solid, Yield: 67.5%, m.p. 160.0–161.5 °C. ^1^H NMR (400 MHz, DMSO-*d*_6_) δ 8.07 (d, *J* = 7.3 Hz, 1H, H-N), 7.18 (d, *J* = 7.7 Hz, 2H), 7.01 (d, *J* = 7.8 Hz, 2H), 2.37–2.23 (m, 1H, H-2), 2.14–2.07 (m, 1H, H-2), 1.61 (d, *J* = 4.2 Hz, 1H, H-3), 1.25–1.19 (m, 1H, H-3), 1.05 (d, *J* = 4.3 Hz, 1H), 0.50–0.42 (m, 2H), 0.30–0.24 (m, 2H). ^13^C NMR (100 MHz, DMSO-*d*_6_) δ 171.75 (C=O), 140.32, 130.41, 128.24, 127.68, 25.84, 23.23, 22.47, 15.35, 5.75, 5.71. HRMS (ESI) *m*/*z* [M + H]^+^ calcd for C_13_H_15_ClNO: 236.0837, found: 236.0845.
*2-(4-Chlorophenyl)-N-cyclobutylcyclopropane-1-carboxamide* (**F46**): White solid, Yield: 72.8%, m.p. 194.5–195.7 °C. ^1^H NMR (400 MHz, DMSO-*d*_6_) δ 8.37 (d, *J* = 7.8 Hz, 1H, H-N), 7.36–7.26 (m, 2H), 7.16–7.11 (m, 2H), 4.26–4.13 (m, 1H), 2.26–2.18 (m, 1H, H-2), 2.17–2.07 (m, 2H), 1.90–1.80 (m, 2H), 1.80–1.74 (m, 1H), 1.65–1.56 (m, 2H), 1.35–1.28 (m, 1H), 1.21–1.13 (m, 1H). ^13^C NMR (100 MHz, DMSO-*d*_6_) δ 169.53 (C=O), 140.36, 130.42, 128.25, 127.68, 43.99, 30.54, 30.46, 25.90, 23.17, 15.31, 14.62. HRMS (ESI) *m*/*z* [M + H]^+^ calcd for C_14_H_17_ClNO: 250.0993, found: 250.1002.
*2-(4-Chlorophenyl)-N-cyclopentylcyclopropane-1-carboxamide* (**F47**): White solid, Yield: 74.6%, m.p. 197.9–199.0 °C. ^1^H NMR (400 MHz, DMSO-*d*_6_) δ 8.09 (d, *J* = 7.2 Hz, 1H, H-N), 7.31 (d, *J* = 7.8 Hz, 2H), 7.13 (d, *J* = 7.9 Hz, 2H), 4.07–3.95 (m, 1H), 2.32–2.18 (m, 1H, H-2), 1.89–1.70 (m, 3H), 1.67–1.55 (m, 2H), 1.53–1.42 (m, 2H), 1.39–1.25 (m, 3H), 1.16 (d, *J* = 4.2 Hz, 1H). ^13^C NMR (100 MHz, DMSO-*d*_6_) δ 170.03 (C=O), 140.48, 130.37, 128.24, 127.65, 50.46, 32.53, 32.33, 25.92, 23.41, 23.05, 15.39. HRMS (ESI) *m*/*z* [M+Na]^+^ calcd for C_15_H_18_ClNNaO: 286.0969, found: 286.0980.
*2-(4-Chlorophenyl)-N-cyclohexylcyclopropane-1-carboxamide* (**F48**): White solid, Yield: 74.5%, m.p. 181.3–183.3 °C. ^1^H NMR (400 MHz, DMSO-*d*_6_) δ 8.00 (d, *J* = 7.7 Hz, 1H, H-N), 7.31 (d, *J* = 8.0 Hz, 2H), 7.13 (d, *J* = 7.6 Hz, 2H), 3.57–3.50 (m, 1H), 2.26–2.16 (m, 1H, H-2), 1.88–1.80 (m, 1H, H-1), 1.77–1.60 (m, 4H), 1.52 (s, 1H), 1.36–1.29 (m, 1H), 1.27–1.19 (m, 2H), 1.18–1.05 (m, 4H). ^13^C NMR (100 MHz, DMSO-*d*_6_) δ 169.61 (C=O), 140.48, 130.37, 128.24, 127.65, 47.65, 32.64, 32.52, 25.92, 25.25, 24.55, 23.06, 15.45. HRMS (ESI) *m*/*z* [M + H]^+^ calcd for C_16_H_21_ClNO: 278.1306, found: 278.1313.
*2-(4-Chlorophenyl)-N-(p-tolyl)cyclopropane-1-carboxamide* (**F49**): White solid, Yield: 62.5%, m.p. 192.3–194.2 °C. ^1^H NMR (400 MHz, DMSO-*d*_6_) δ 10.16 (s, 1H, H-N), 7.47 (d, *J* = 7.8 Hz, 2H), 7.34 (d, *J* = 7.8 Hz, 2H), 7.21 (d, *J* = 8.0 Hz, 2H), 7.09 (d, *J* = 7.8 Hz, 2H), 2.41–2.34 (m, 1H, H-2), 2.23 (s, 3H), 2.07–2.03 (m, 1H, H-1), 1.55–1.44 (m, 1H, H-3), 1.36–1.31 (m, 1H, H-3). ^13^C NMR (100 MHz, DMSO-*d*_6_) δ 169.43 (C=O), 140.07, 136.76, 132.01, 130.60, 129.17, 128.30, 127.82, 118.96, 26.82, 24.12, 20.47, 15.72. HRMS (ESI) *m*/*z* [M + H]^+^ calcd for C_17_H_17_ClNO: 286.0993, found: 286.0999.
*2-(4-Chlorophenyl)-N-(m-tolyl)cyclopropane-1-carboxamide* (**F50**): White solid, Yield: 69.4%, m.p. 163.5–165.0 °C. ^1^H NMR (400 MHz, DMSO-*d*_6_) δ 9.54 (s, 1H, H-N), 7.49 (d, *J* = 7.5 Hz, 1H), 7.35 (d, *J* = 7.8 Hz, 2H), 7.21 (t, *J* = 8.9 Hz, 3H), 7.13 (d, *J* = 7.5 Hz, 1H), 7.06 (d, *J* = 7.1 Hz, 1H), 2.30–2.34 (m, 1H, H-2), 2.21 (s, 4H), 1.49–1.45 (m, 1H, H-3), 1.38–1.27 (m, 1H, H-3). ^13^C NMR (100 MHz, DMSO-*d*_6_) δ 169.87 (C=O), 140.18, 136.44, 131.08, 130.63, 130.37, 128.34, 127.85, 126.00, 124.98, 124.63, 26.17, 24.04, 17.95, 15.98. HRMS (ESI) *m*/*z* [M + H]^+^ calcd for C_17_H_17_ClNO: 286.0993, found: 286.1000.
*2-(4-Chlorophenyl)-N-(o-tolyl)cyclopropane-1-carboxamide* (**F51**): White solid, Yield: 67.7%, m.p. 157.9–159.3 °C. ^1^H NMR (400 MHz, DMSO-*d*_6_) δ 9.55 (s, 1H, H-N), 7.48 (d, *J* = 7.9 Hz, 1H), 7.35 (d, *J* = 8.1 Hz, 2H), 7.24–7.18 (m, 3H), 7.14 (d, *J* = 1.7 Hz, 1H), 7.08–7.03 (m, 1H), 2.40–2.36 (m, 1H, H-2), 2.26–2.22 (m, 1H, H-2), 2.20 (s, 3H), 1.49–1.43 (m, 1H, H-3), 1.36–1.29 (m, 1H, H-3). ^13^C NMR (100 MHz, DMSO-*d*_6_) δ 169.80 (C=O), 140.17, 136.42, 131.02, 130.59, 130.33, 128.30, 127.82, 125.96, 124.92, 124.57, 26.16, 24.00, 17.93, 15.93. HRMS (ESI) *m*/*z* [M + Na]^+^ calcd for C_17_H_16_ClNNaO: 308.0813, found: 308.0821.
*Ethyl 4-(2-(4-Chlorophenyl)cyclopropane-1-carbonyl)piperazine-1-carboxylate* (**F52**): Yellow oil, Yield: 76.0%. ^1^H NMR (400 MHz, DMSO-*d*_6_) δ 7.33–7.25 (m, 2H), 7.23–7.15 (m, 2H), 4.09–4.00 (m, 2H), 3.73–3.56 (m, 2H), 3.55–3.44 (m, 2H), 3.35 (d, *J* = 5.5 Hz, 2H), 2.38–2.24 (m, 2H), 1.50–1.38 (m, 1H, H-3), 1.18 (t, *J* = 7.1 Hz, 4H). ^13^C NMR (100 MHz, DMSO-*d*_6_) δ 169.58 (C=O), 154.60, 139.98, 130.60, 128.17, 127.87, 60.92, 44.67, 43.55, 43.07, 41.41, 24.11, 22.31, 16.48, 14.50. HRMS (ESI) *m*/*z* [M + H]^+^ calcd for C_17_H_22_ClN_2_O_3_: 337.1313, found: 337.1317.
*2-(4-Chlorophenyl)-N-(thiazol-2-yl)cyclopropane-1-carboxamide* (**F53**): White solid, Yield: 59.0%, m.p. 218.3–221.0 °C. ^1^H NMR (400 MHz, DMSO-*d*_6_) δ 12.39 (s, 1H, H-N), 7.45 (d, *J* = 3.5 Hz, 1H), 7.35 (d, *J* = 8.0 Hz, 2H), 7.28–7.15 (m, 3H), 2.30–2.16 (m, 1H, H-1), 1.63–1.41 (m, 2H, H-3). ^13^C NMR (100 MHz, DMSO-*d*_6_) δ 169.81 (C=O), 157.99, 139.35, 137.66, 130.91, 128.36, 127.99, 113.44, 25.38, 25.26, 16.38. HRMS (ESI) *m*/*z* [M + H]^+^ calcd for C_13_H_12_ClN_2_OS: 279.0353, found: 279.0363.

### 3.7. The Antimicrobial Activity In Vitro

Three strains of bacteria (*S. aureus*, *E. coli*, and *P. aeruginosa*) and one strain of fungal (*C. albicans*) were selected to determine the antimicrobial activity in vitro by the broth microdilution method. The fungal and bacterial strains were obtained from China General Microbiological Culture Collection Center. The bacteria and fungi were cultured to 1–5 × 10^3^ CFU/mL in Mueller Hinton Broth medium and RPMI1640 medium, respectively, before use. The commercial ciprofloxacin or fluconazole was selected as the positive control. All compounds were dissolved in DMSO and serially diluted, and the concentrations of all tested compounds were 1, 2, 4, 8, 16, 32, 64, and 128 μg/mL. Then, the plates were continuously incubated at 37 °C for 24 h. The OD values were read on a Microplate reader at a wavelength of 600 nm. The plates without drugs acted as the growth control, while the plates without cells and drugs acted as blank control. The MIC_80_ value was the lowest concentration; the inhibition rate was ≥80%. The test was performed in triplicates.

$$Inhibition\;rate\left(\%\right)=\frac{{OD}_{control}-{OD}_{sample}}{{OD}_{control}-{OD}_{blank}}\times 100\%$$

### 3.8. Molecular Docking Analysis

The 3D crystal structure of CYP51 (PDB code: 6AY6) was acquired from the RCSB Protein Date Bank (https://www.rcsb.org/structure/6AY6 (accessed on 7 September 2017)). The molecular docking process was conducted for the investigation of the binding mode of compounds **F8**, **F24**, and **F42** with CYP51 using Discovery Studio 2016 software (Accelrys, San Diego, CA, USA) [32]. ChemBio 3D Ultra 14.0 software was used to generate the 3D structure of the above compounds and treated with energy minimization [36]. Water molecules and the co-crystallized ligand of the 3D crystal structure of CYP51 were removed, and the crystal structure was hydrotreated before molecular docking. The parameters of docking were in accordance with the previously reported method [13].

## 4. Conclusions

In summary, a series of amide derivatives containing cyclopropane were designed and synthesized, and their antimicrobial activity in vitro was evaluated. The results showed that the four and three of the compounds exhibited moderate activity against *Staphylococcus aureus* and *Escherichia coli*, respectively. Three compounds were sensitive to *Candida albicans* and exhibited excellent antifungal activity, with great potential for further development. SAR analysis showed that introducing halogens into the benzene ring was beneficial for improving antibacterial and antifungal activity. The presence of thiazolamide contributed to the improvement of antibacterial activity, while benzamides were beneficial to the improvement of antifungal activity. Compounds **F8**, **F24**, and **F42** showed the highest activity against *C. albicans* (MIC_80_ = 16 μg/mL). The molecular docking results showed that the above compounds had a good binding affinity with the CYP51 protein. Thus, amide compounds containing cyclopropane can be considered promising antimicrobial lead compounds. It is necessary to further study the in vivo activity and mechanism of action of these compounds.

## Figures and Tables

**Table 1:** The results of antimicrobial and antifungal activity of tested compounds (MIC_80_, μg/mL).

Compound		Gram-Negative Bacteria		Gram-Positive Bacteria	Fungus	Compound		Gram-Negative Bacteria		Gram-Positive Bacteria	Fungus
No.	R	*S. aureus*	*E. coli*	*P. aeruginosa*	*C. albicans*	No.	R	*S. aureus*	*E. coli*	*P. aeruginosa*	*C. albicans*
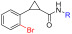 **(F1–F9)**						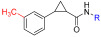 **(F27–F35)**					
**F1**	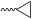	>128	>128	>128	>128	**F27**	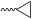	>128	>128	>128	>128
**F2**	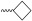	>128	>128	>128	>128	**F28**	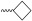	>128	>128	>128	>128
**F3**	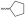	>128	>128	>128	>128	**F29**	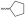	64	>128	>128	>128
**F4**	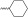	>128	>128	>128	128	**F30**	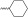	128	>128	>128	>128
**F5**	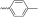	64	128	>128	64	**F31**	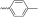	>128	64	>128	>128
**F6**	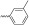	>128	>128	>128	>128	**F32**	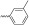	>128	>128	>128	64
**F7**	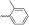	128	>128	>128	32	**F33**	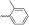	>128	>128	>128	128
**F8**	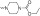	>128	>128	>128	16	**F34**	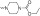	>128	>128	>128	128
**F9**	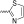	64	32	>128	64	**F35**	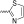	>128	>128	>128	>128
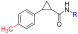 **(F10–F17)**						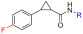 **(F36–F44)**					
**F10**	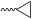	>128	>128	>128	128	**F36**	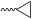	128	>128	>128	128
**F11**	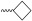	>128	>128	>128	>128	**F37**	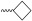	>128	>128	>128	>128
**F12**	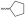	>128	>128	>128	>128	**F38**	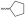	>128	>128	>128	>128
**F13**	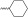	>128	>128	>128	>128	**F39**	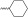	>128	>128	>128	>128
**F14**	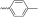	>128	>128	>128	128	**F40**	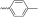	>128	>128	>128	>128
**F15**	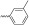	>128	>128	>128	>128	**F41**	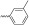	>128	>128	>128	>128
**F16**	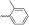	>128	>128	>128	>128	**F42**	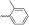	>128	>128	>128	16
**F17**	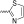	>128	>128	>128	>128	**F43**	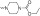	>128	>128	>128	>128
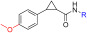 **(F18–F26)**						**F44**	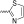	>128	>128	>128	>128
**F18**	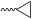	>128	>128	>128	>128	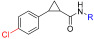 **(F45–F53)**					
**F19**	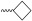	>128	>128	>128	>128	**F45**	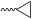	>128	64	>128	>128
**F20**	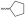	>128	>128	>128	>128	**F46**	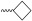	>128	>128	>128	>128
**F21**	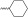	>128	>128	>128	>128	**F47**	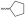	>128	>128	>128	>128
**F22**	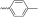	>128	>128	>128	32	**F48**	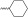	>128	>128	>128	>128
**F23**	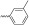	>128	>128	>128	64	**F49**	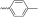	128	>128	>128	64
**F24**	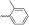	>128	>128	>128	16	**F50**	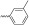	>128	>128	>128	64
**F25**	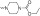	>128	>128	>128	>128	**F51**	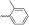	128	>128	>128	32
**F26**	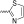	>128	>128	>128	>128	**F52**	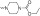	>128	>128	>128	>128
						**F53**	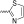	64	128	>128	>128
**Ciprofloxacin**		2	2	2	-	**Ciprofloxacin**		2	2	2	-
**Fluconazole**		-	-	-	2	**Fluconazole**		-	-	-	2

## Data Availability

The data are contained within the article.

## Supplementary Materials

The following supporting information can be downloaded at https://www.mdpi.com/article/10.3390/molecules29174124/s1, Compounds characterization—Pages S2–S86: ^1^H NMR, ^13^C NMR and HRMS spectrum of all the target compounds.
