# Supplementary material for: Design, Synthesis, and Antimicrobial Activity of Amide Derivatives Containing Cyclopropane
Source: Molecules. 2024 Aug 30;29(17):4124. doi: 10.3390/molecules29174124 (PMC11397633; doi:10.3390/molecules29174124)
Supplement: Supplementary file 1 [file molecules-29-04124-s001.zip › molecules-3155158-supplementary.pdf]

Supporting Information For

Design, Synthesis and Antibacterial Activity of Amide Deriva-tives Containing Cyclopropane

Dongdong Chen[1,*], Yu Cheng[2], Lele Shi[2], Xueting Gao[2], Yuhang Huang[2] and Zhenting Du[2,*]

1 Department of Chemical and Material Engineering, Lyuliang University, Lvliang 033001, China College of

2 Chemistry & Pharmacy, Northwest A&F University, Yangling 712100, China; cy2021056736@163.com (Y.C.); shilele23@163.com (L.S.); gxt05012022@163.com (X.G.); 15621485160@163.com (Y.H.)

* Correspondence: chenddxy@163.com (D.C.); duzt@nwsuaf.edu.cn (Z.D.)

CATALOG

1. ^{1}H, ^{13}C and ^{19}F NMR spectra of all compounds……………………………………………………………S2

2. HRMS of all compounds…………………………………………………………………………S60

1. ^{1}H and ^{13}C NMR spectra of all compounds

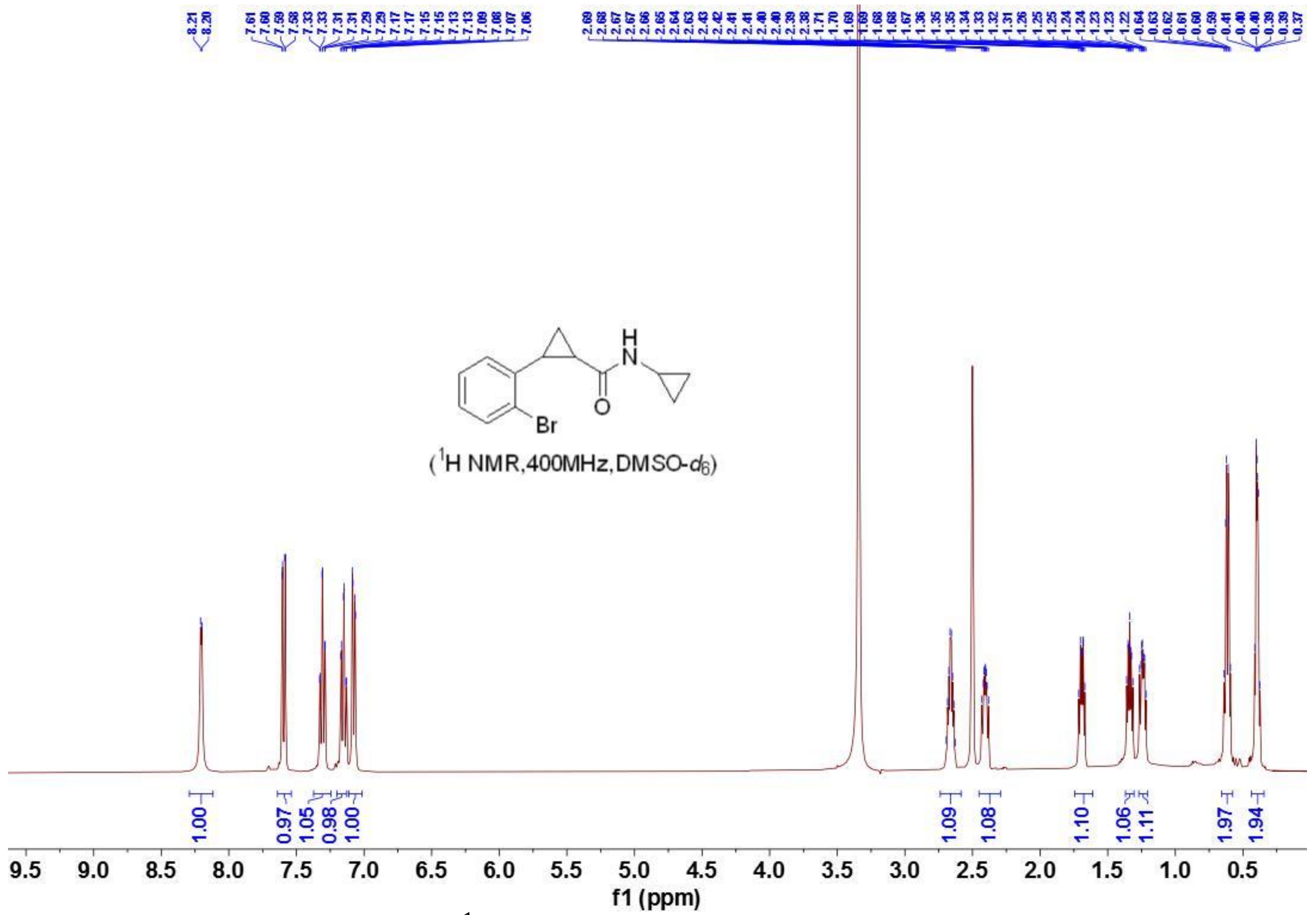

^{1}H NMR of compound **F1**

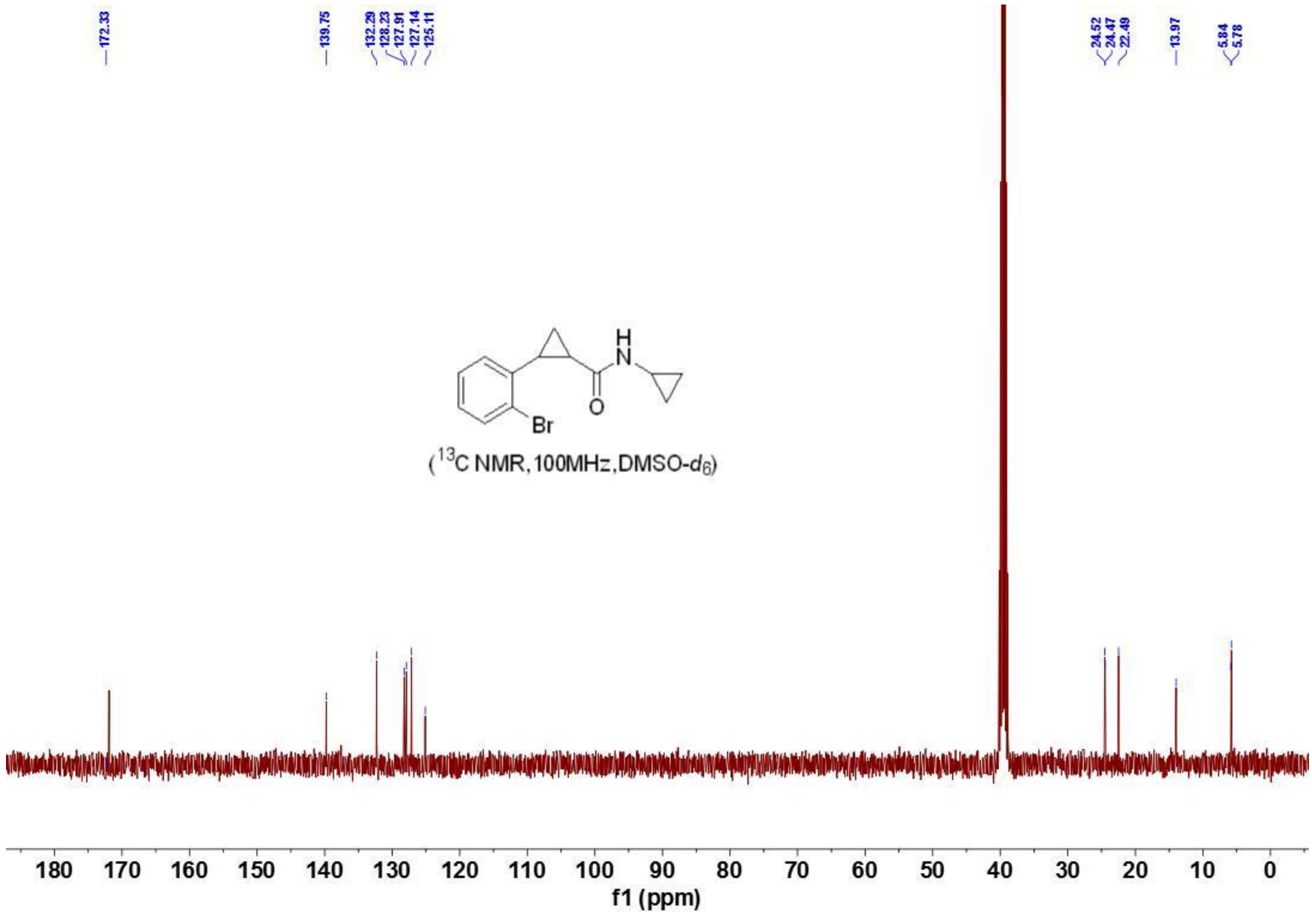

^{13}C NMR of compound **F1**

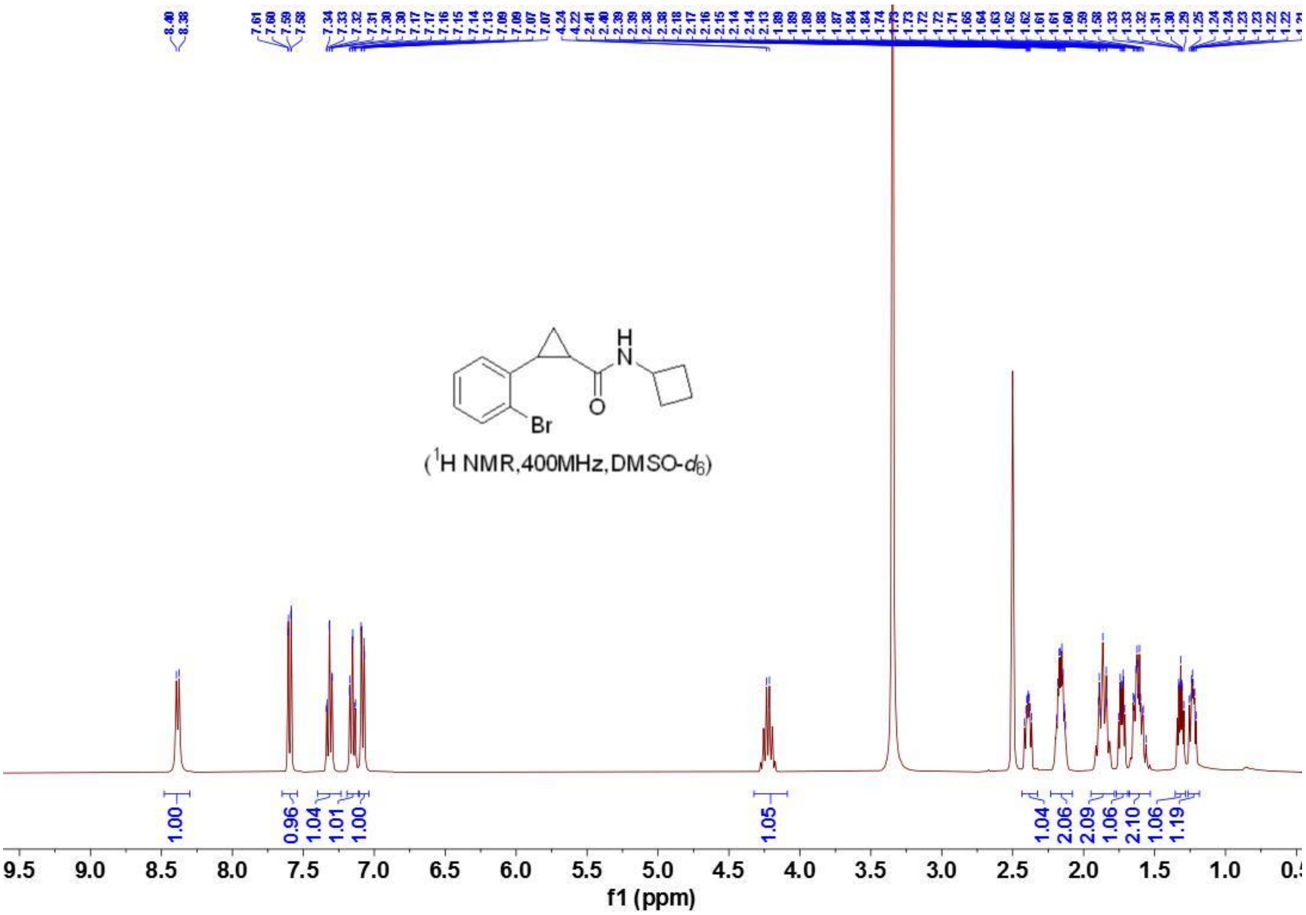

^{1}H NMR of compound **F2**

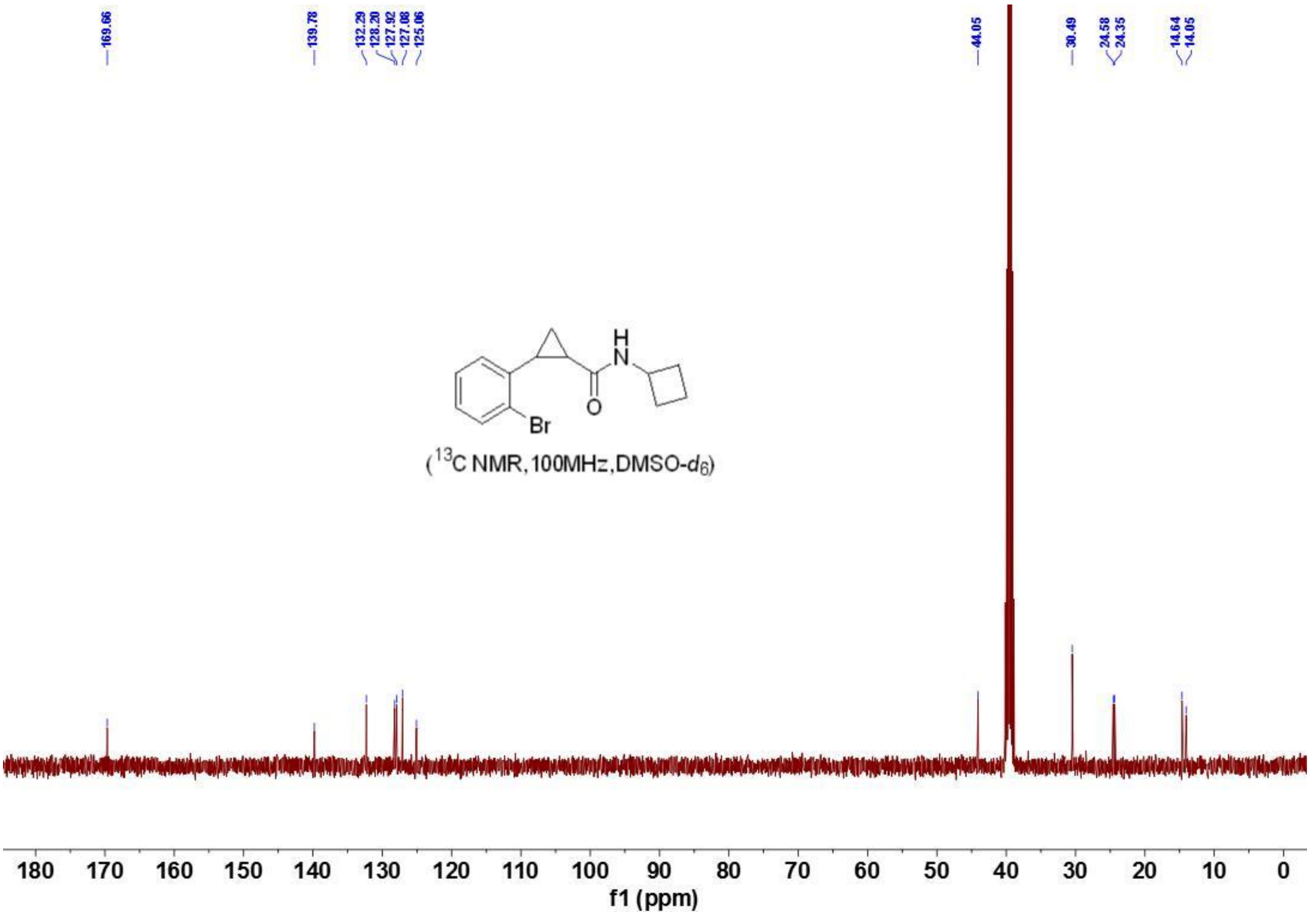

^{13}C NMR of compound **F2**

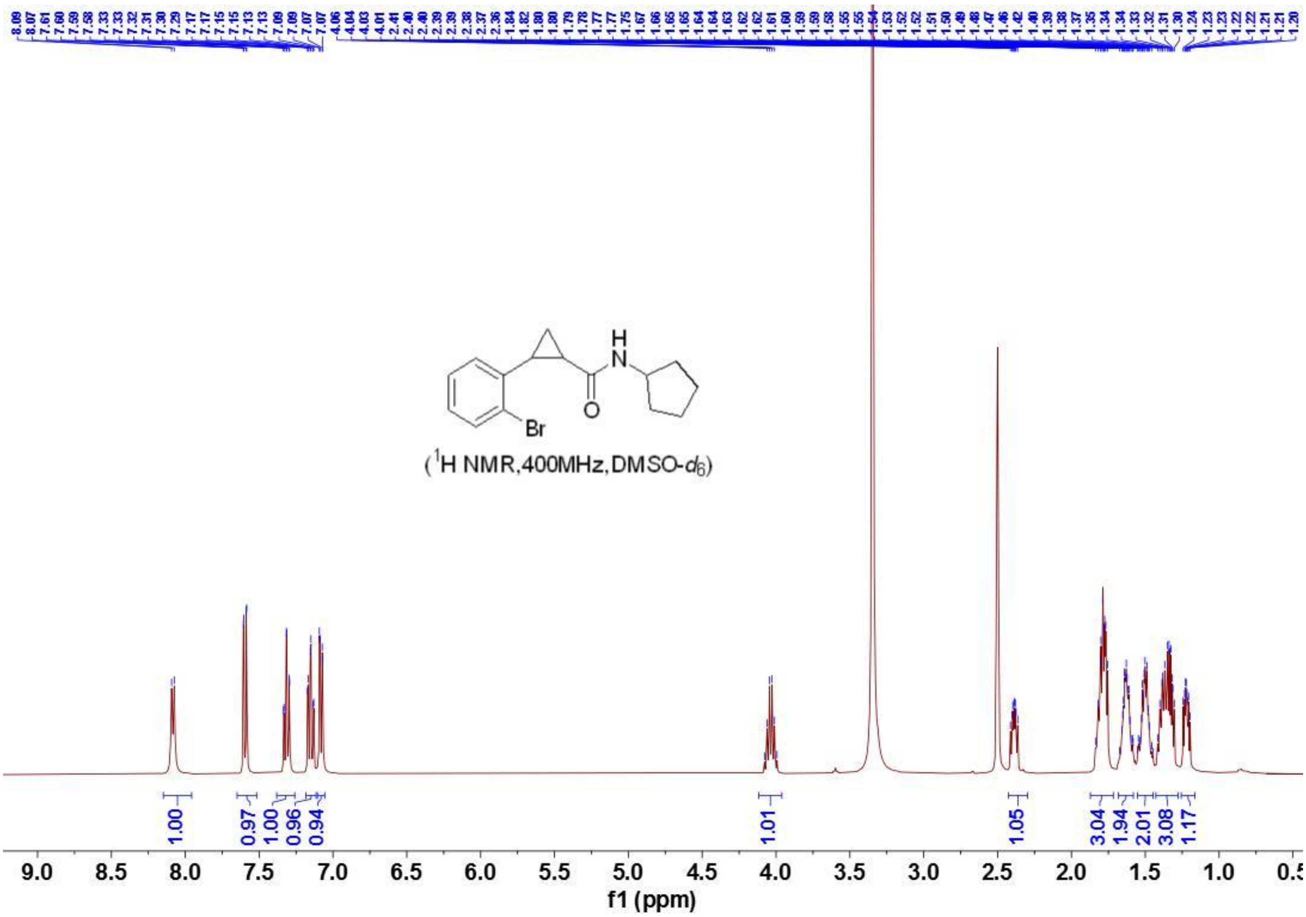

^{1}H NMR of compound **F3**

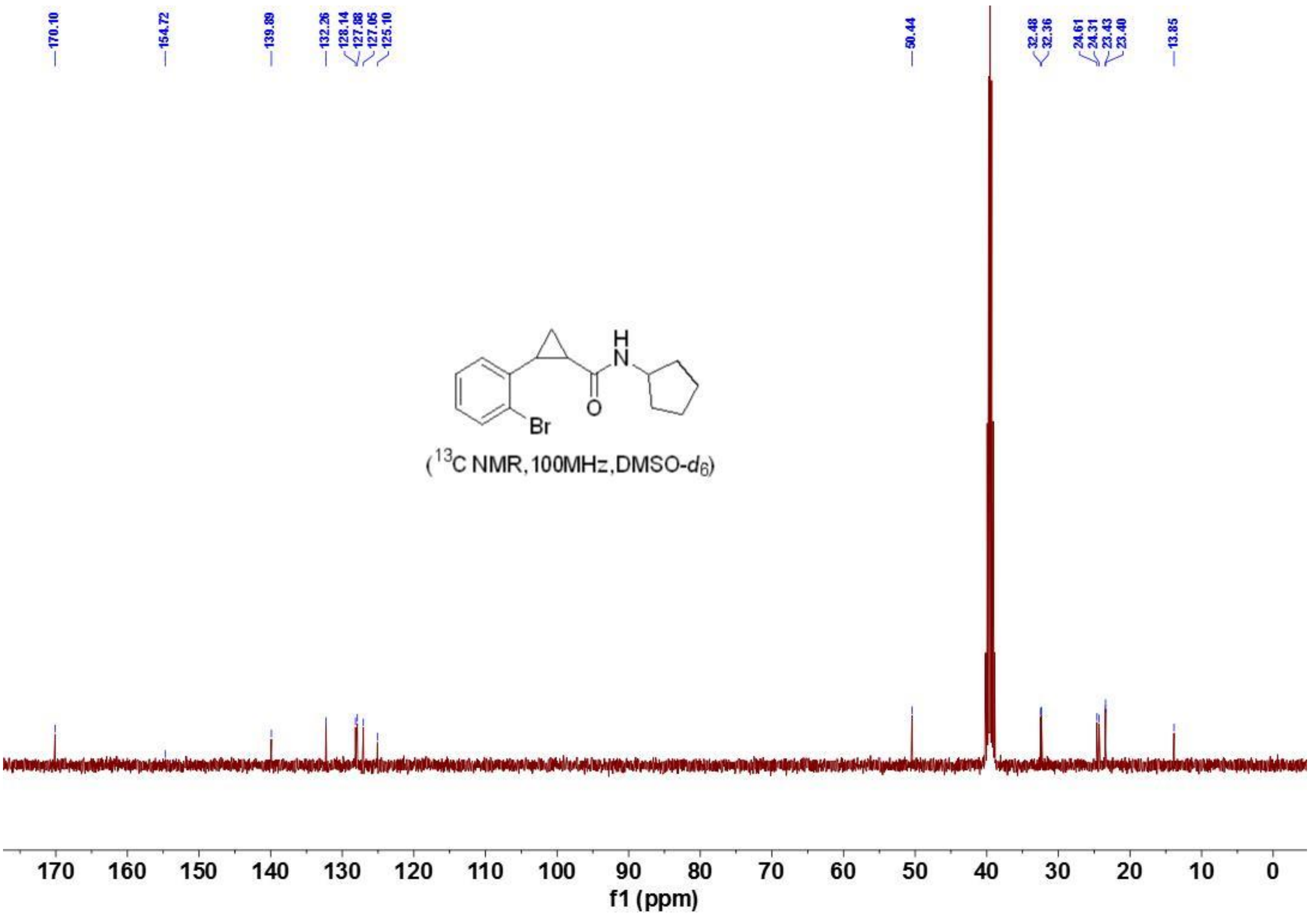

^{13}C NMR of compound **F3**

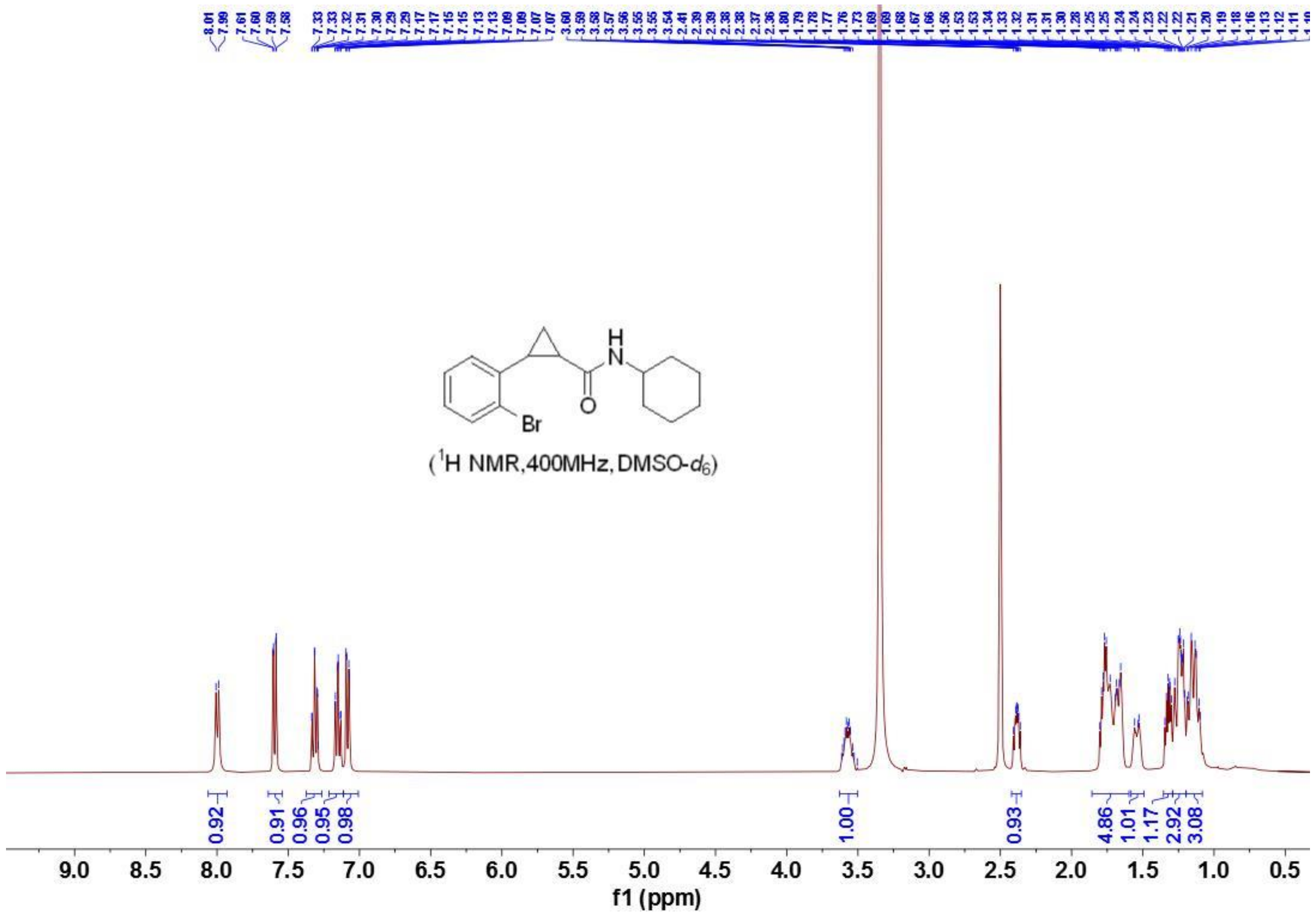

^{1}H NMR of compound **F4**

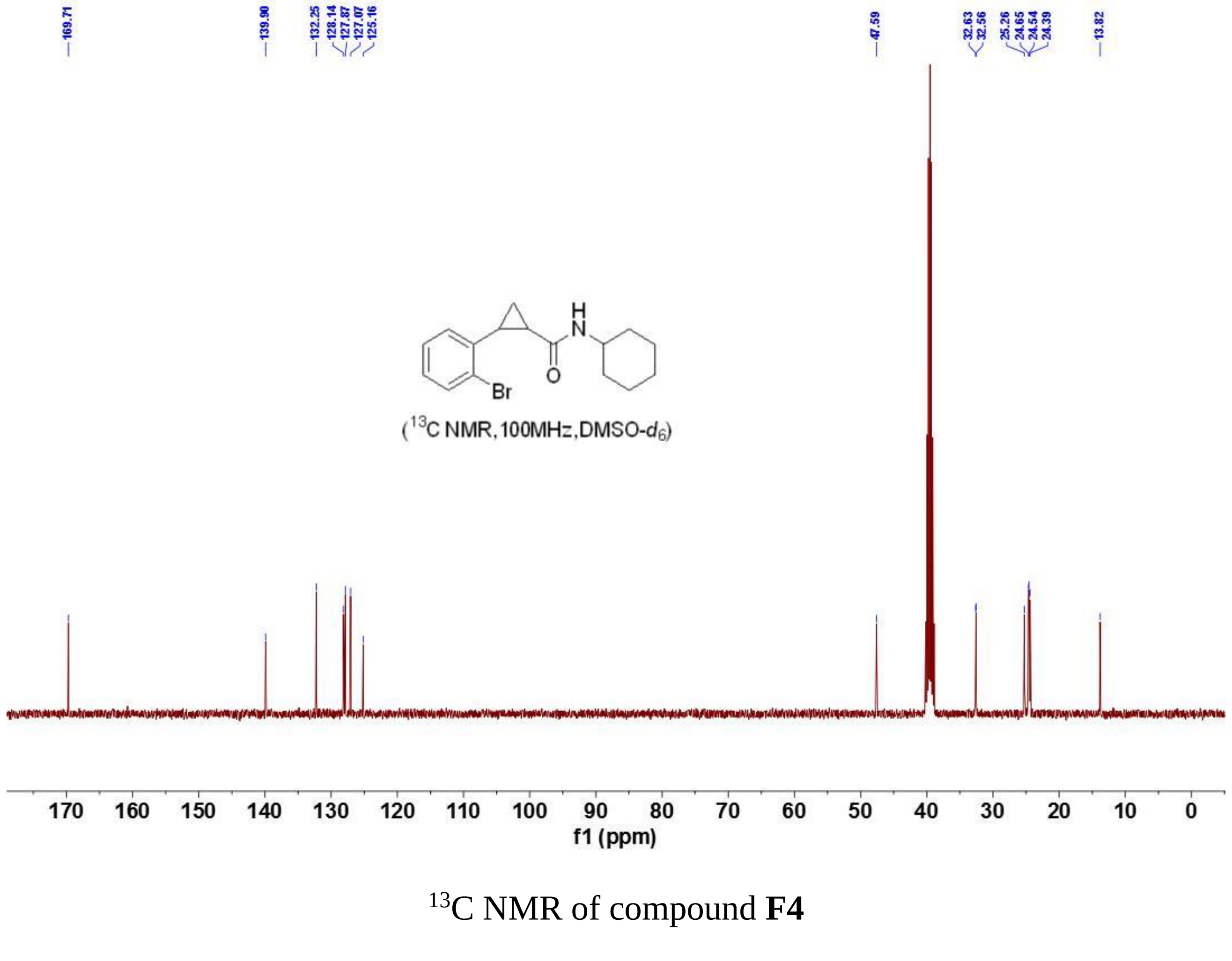

^{13}C NMR of compound **F4**

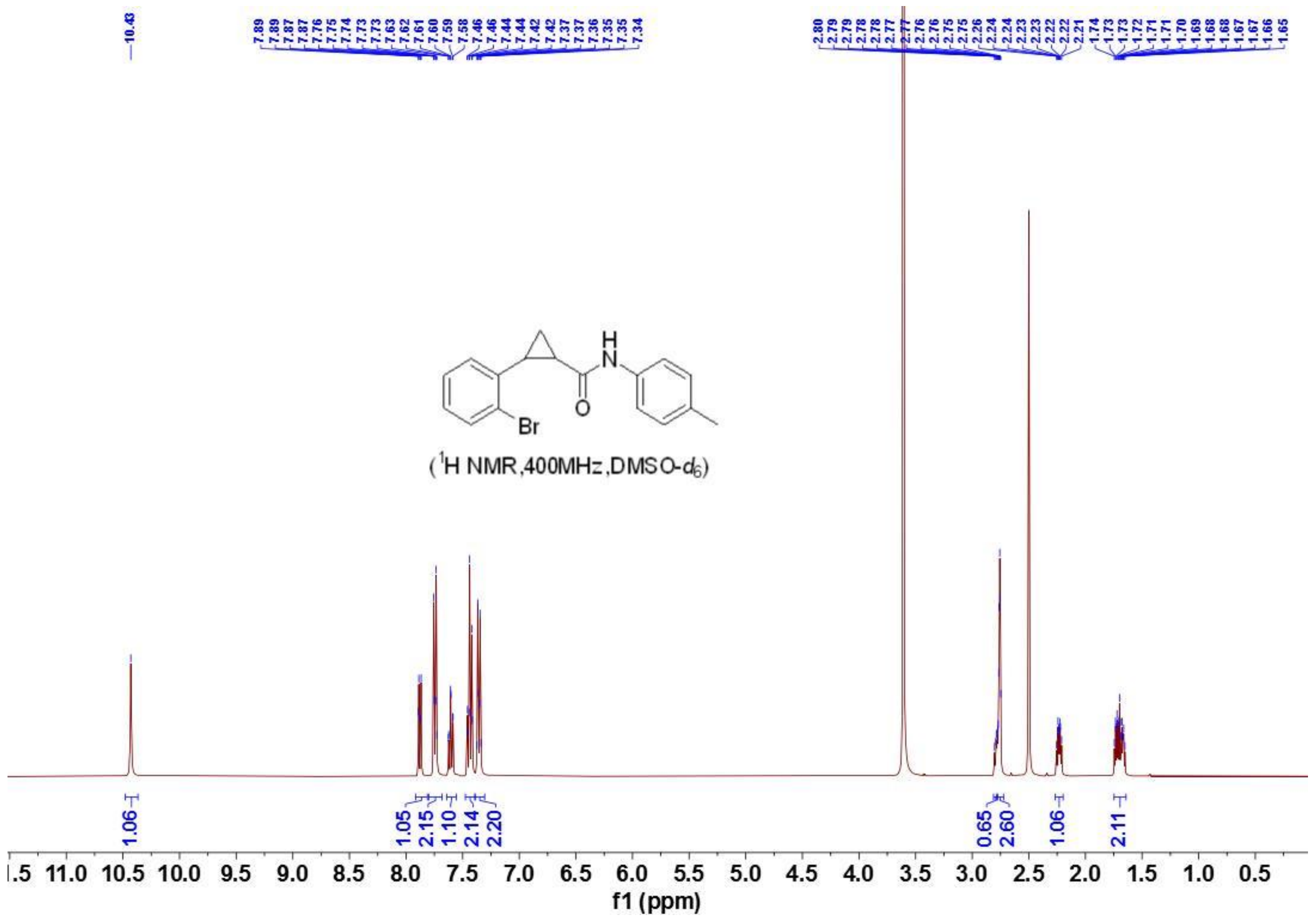

^{1}H NMR of compound **F5**

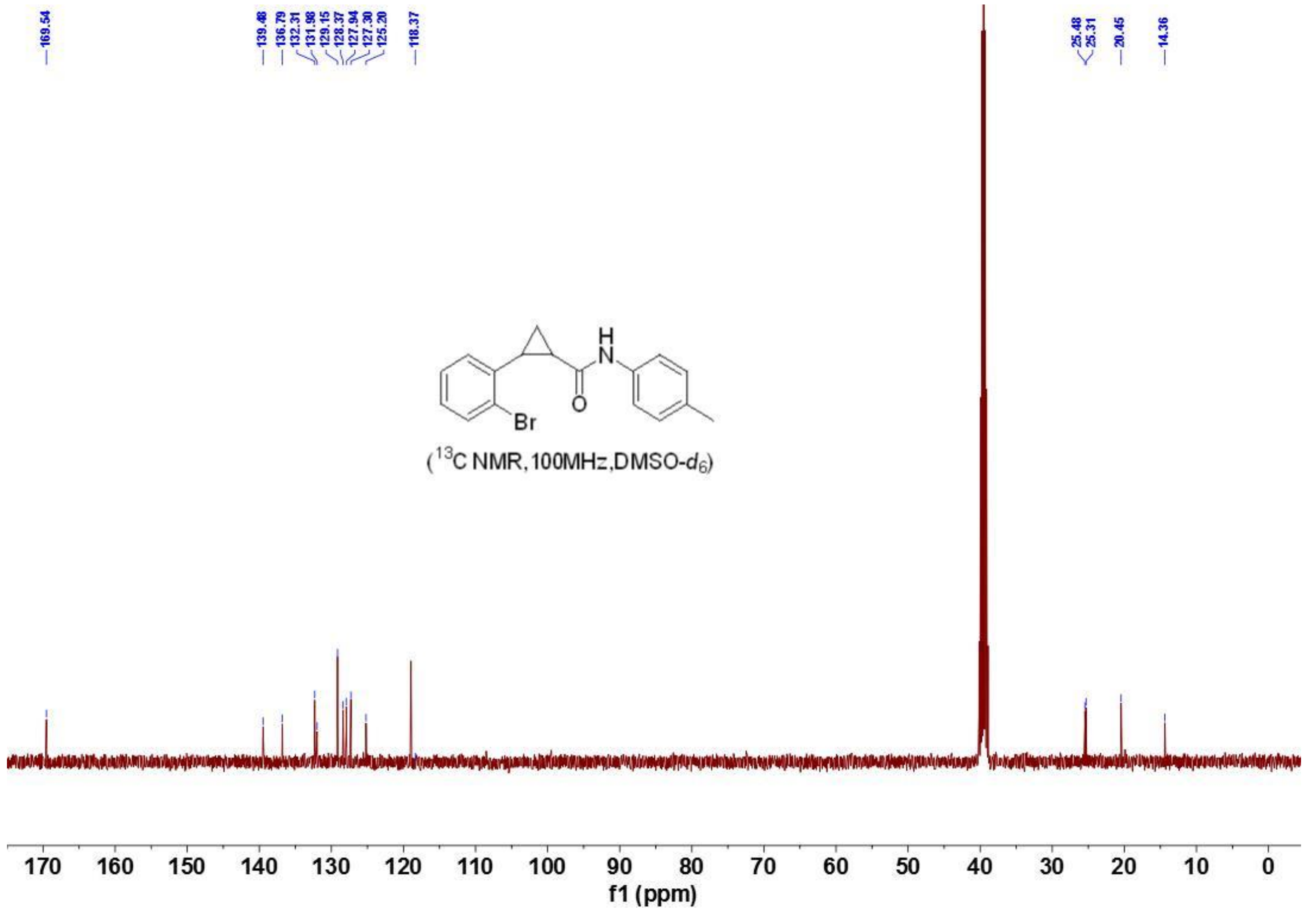

^{13}C NMR of compound **F5**

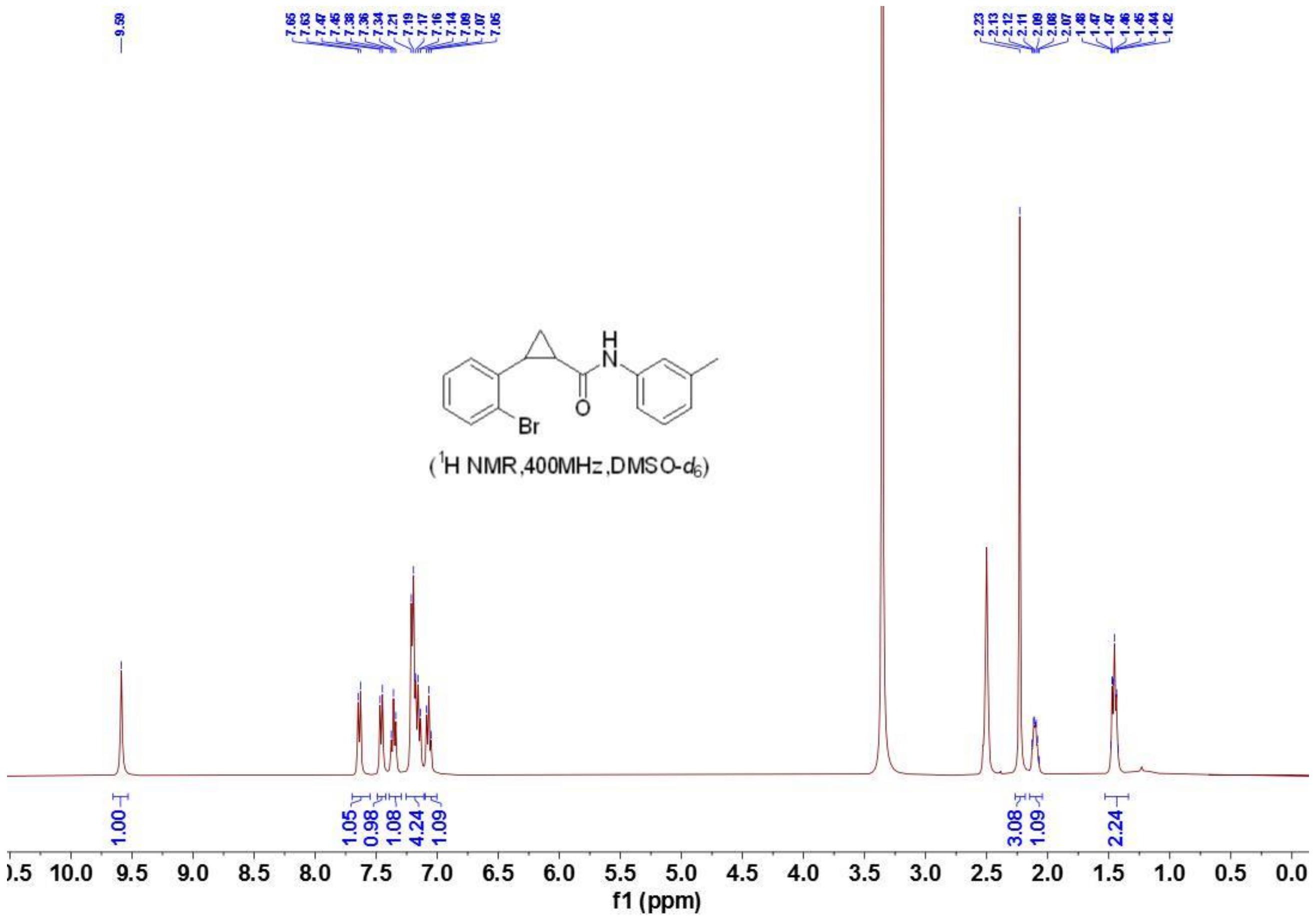

^{1}H NMR of compound **F6**

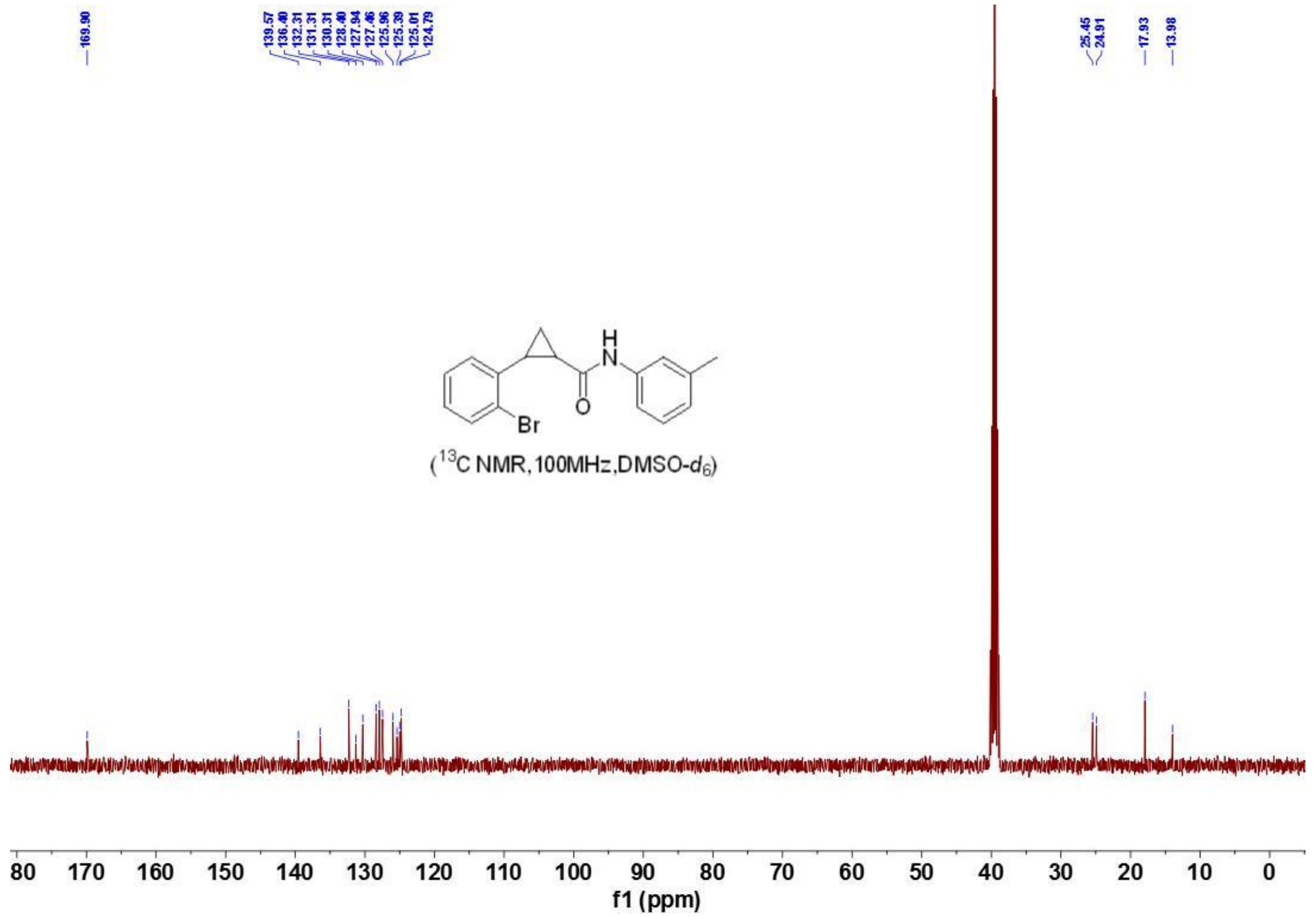

^{13}C NMR of compound **F6**

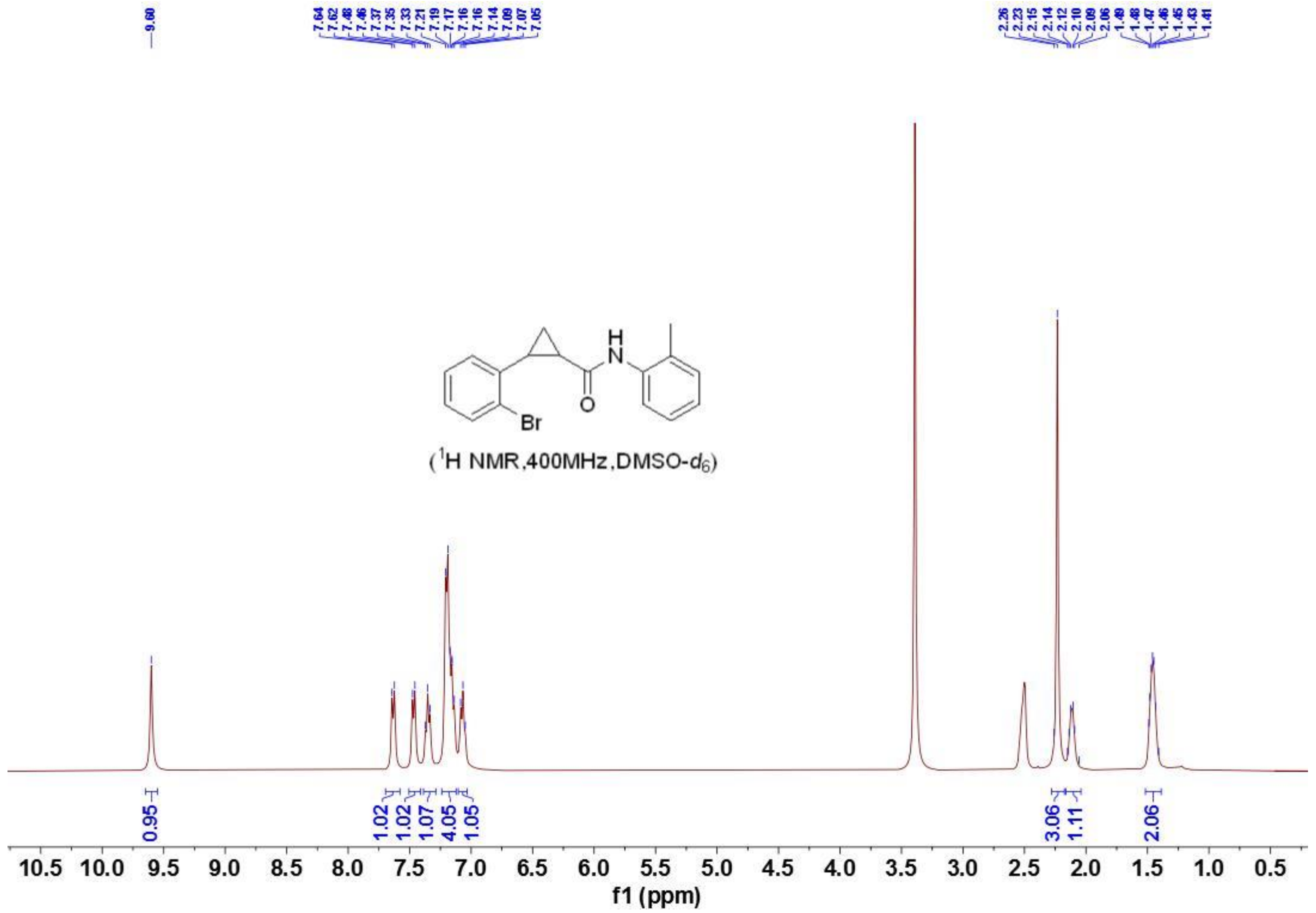

^{1}H NMR of compound **F7**

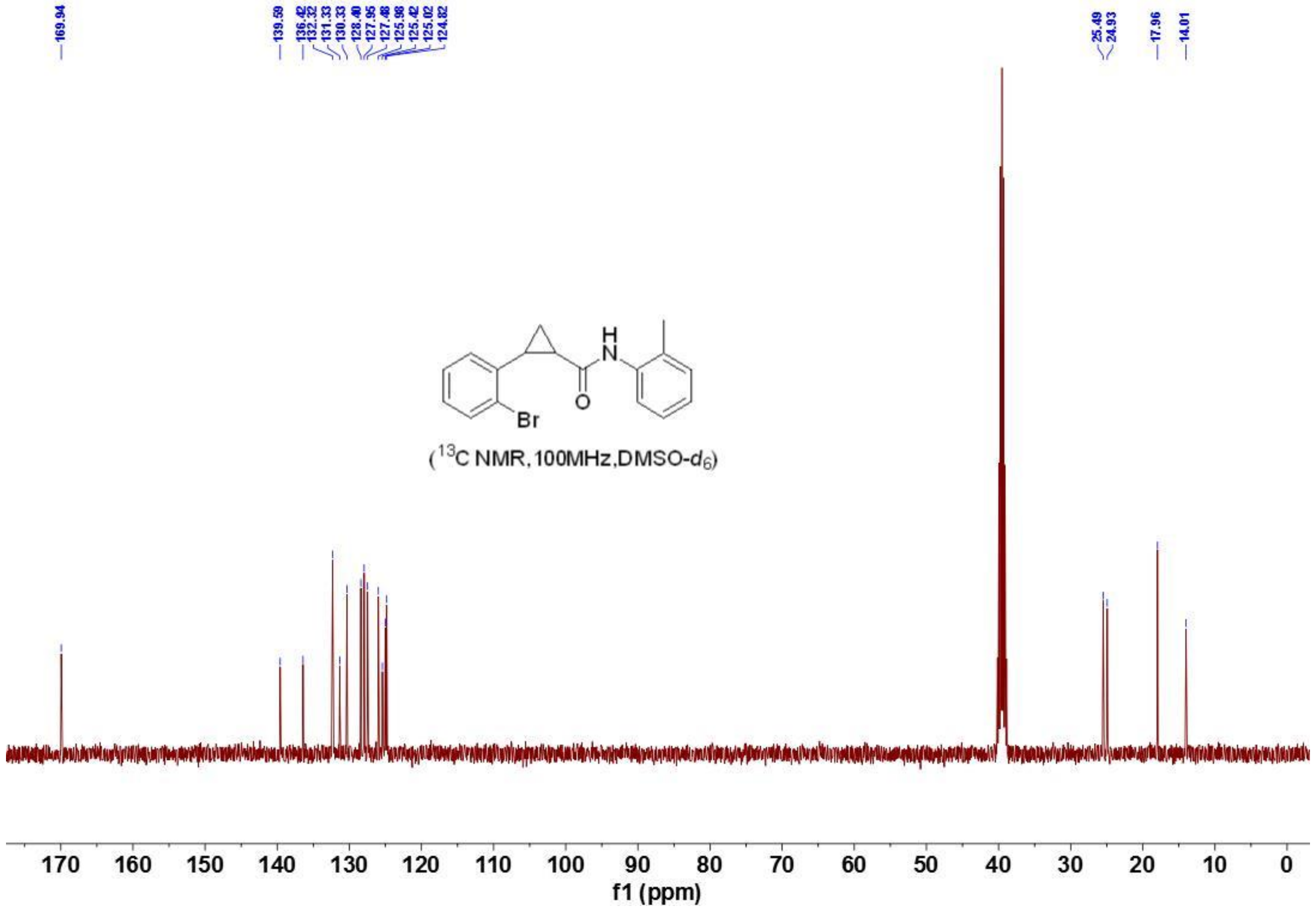

^{13}C NMR of compound **F7**

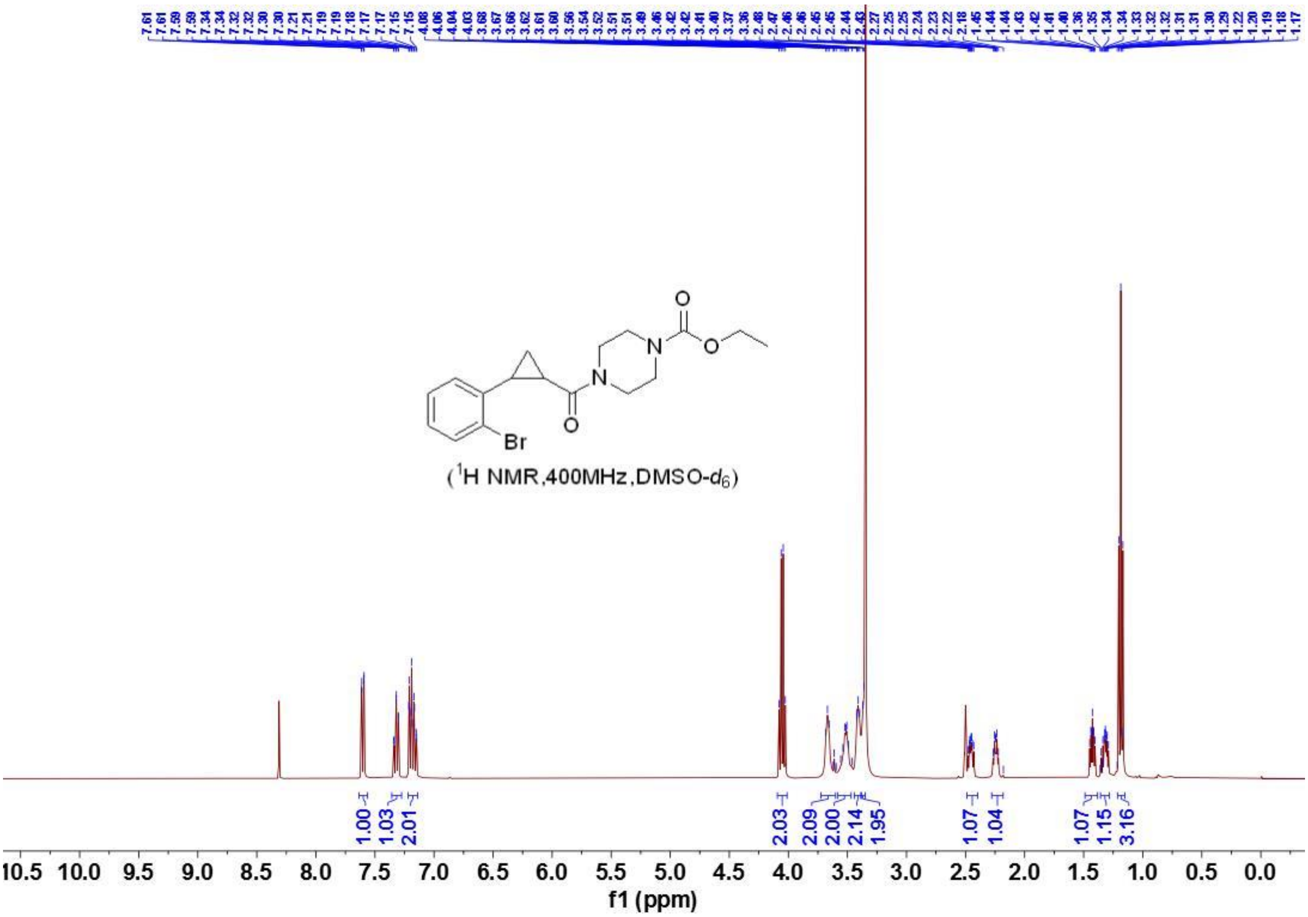

^{1}H NMR of compound **F8**

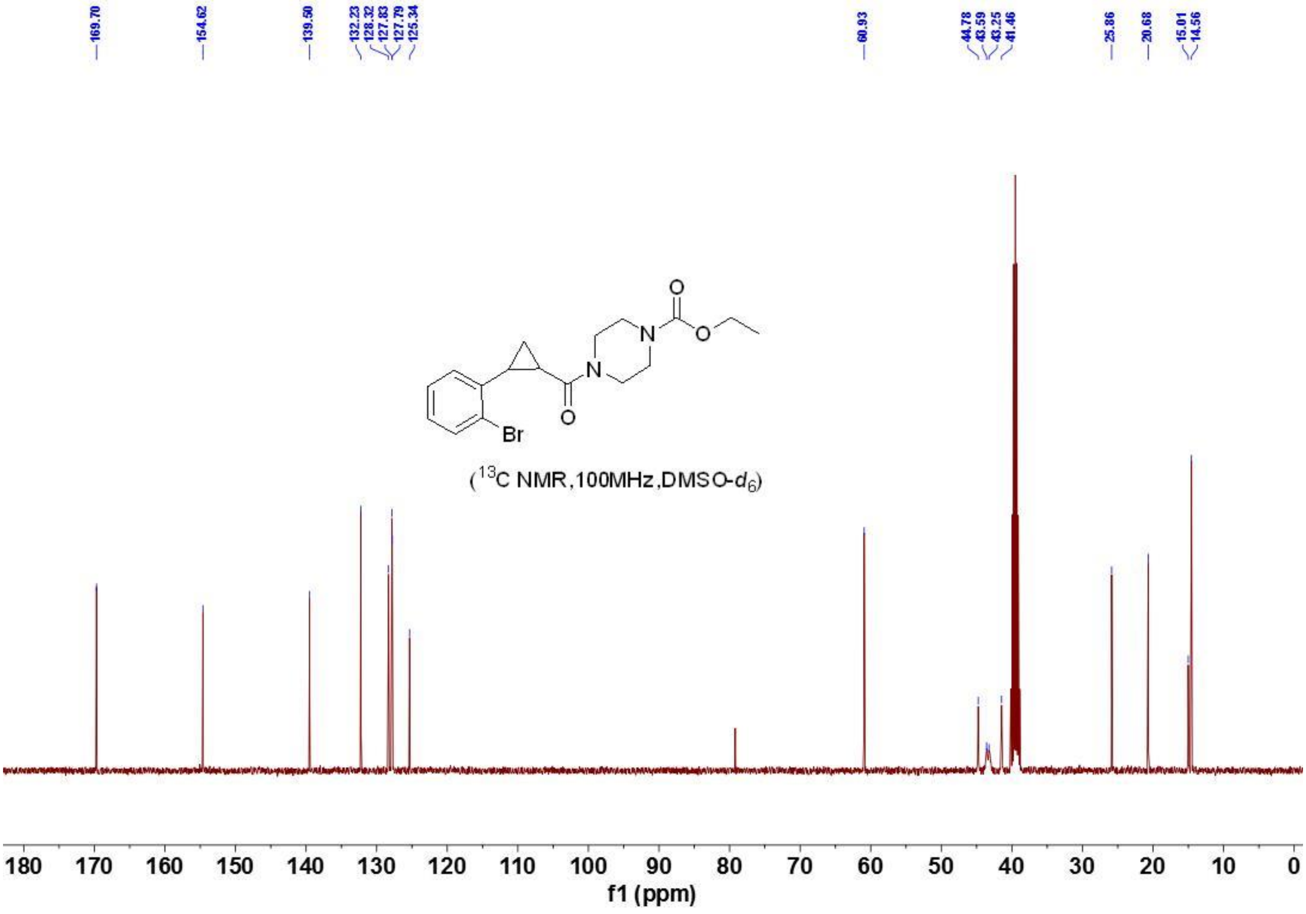

^{13}C NMR of compound **F8**

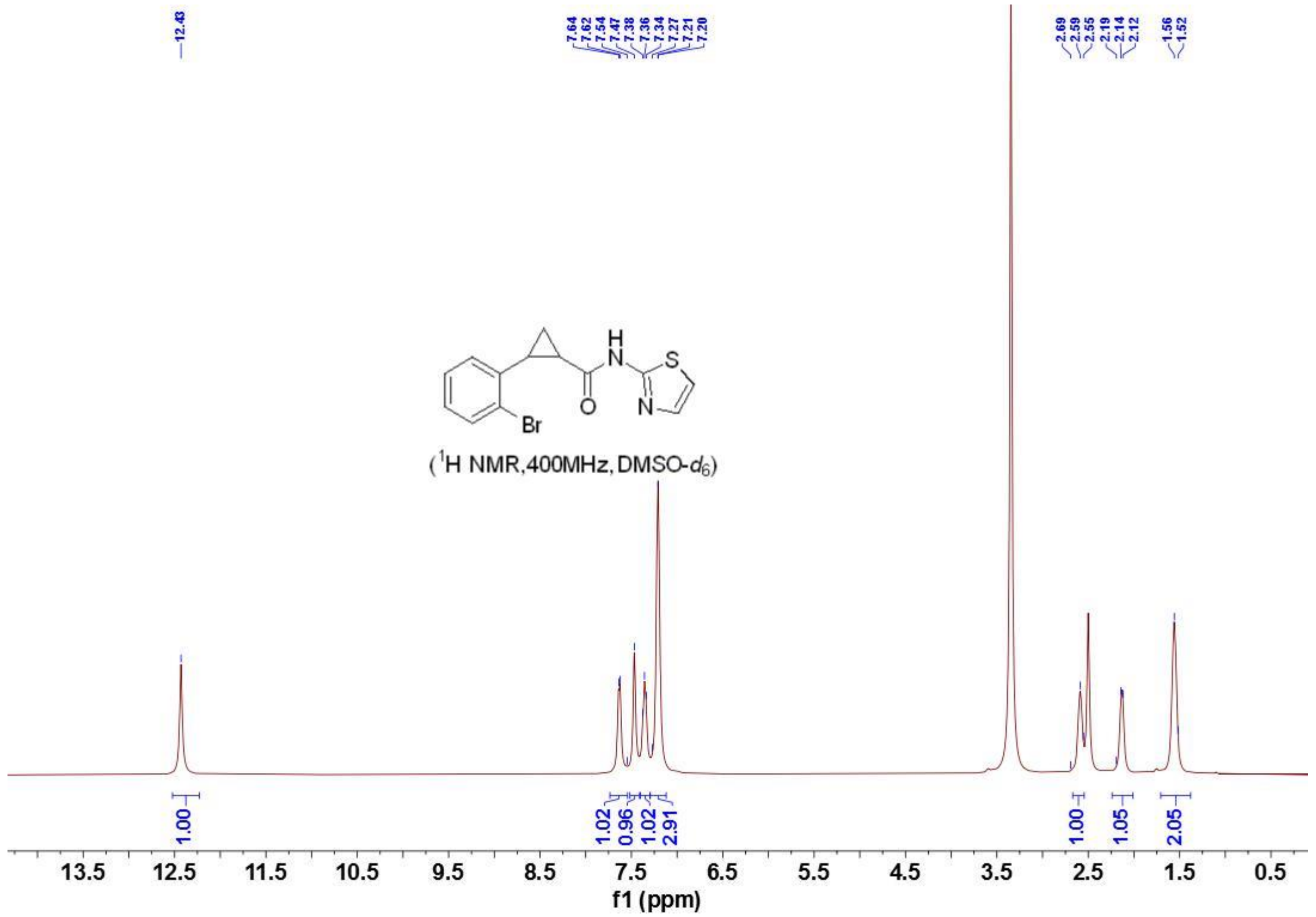

^{1}H NMR of compound **F9**

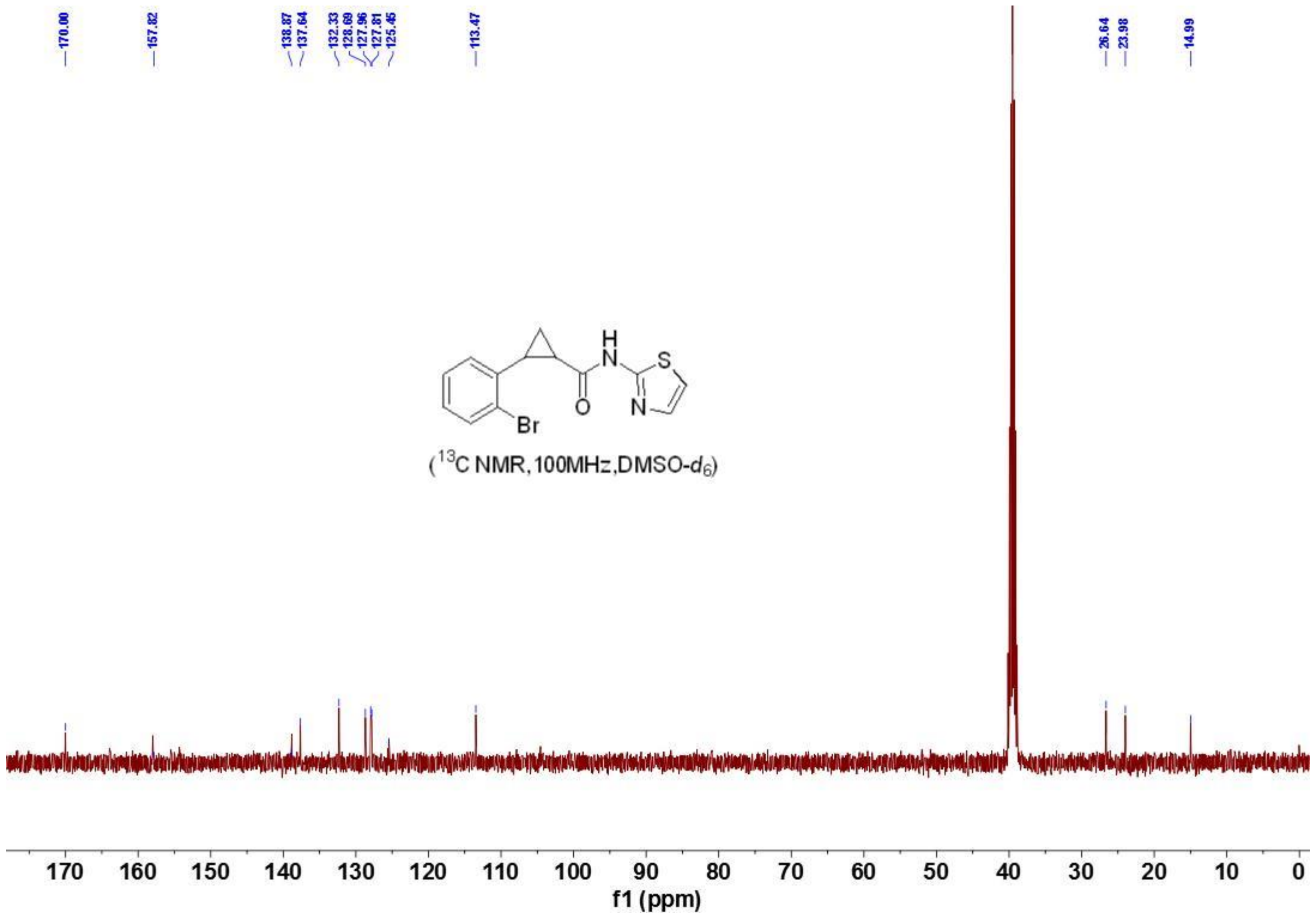

^{13}C NMR of compound **F9**

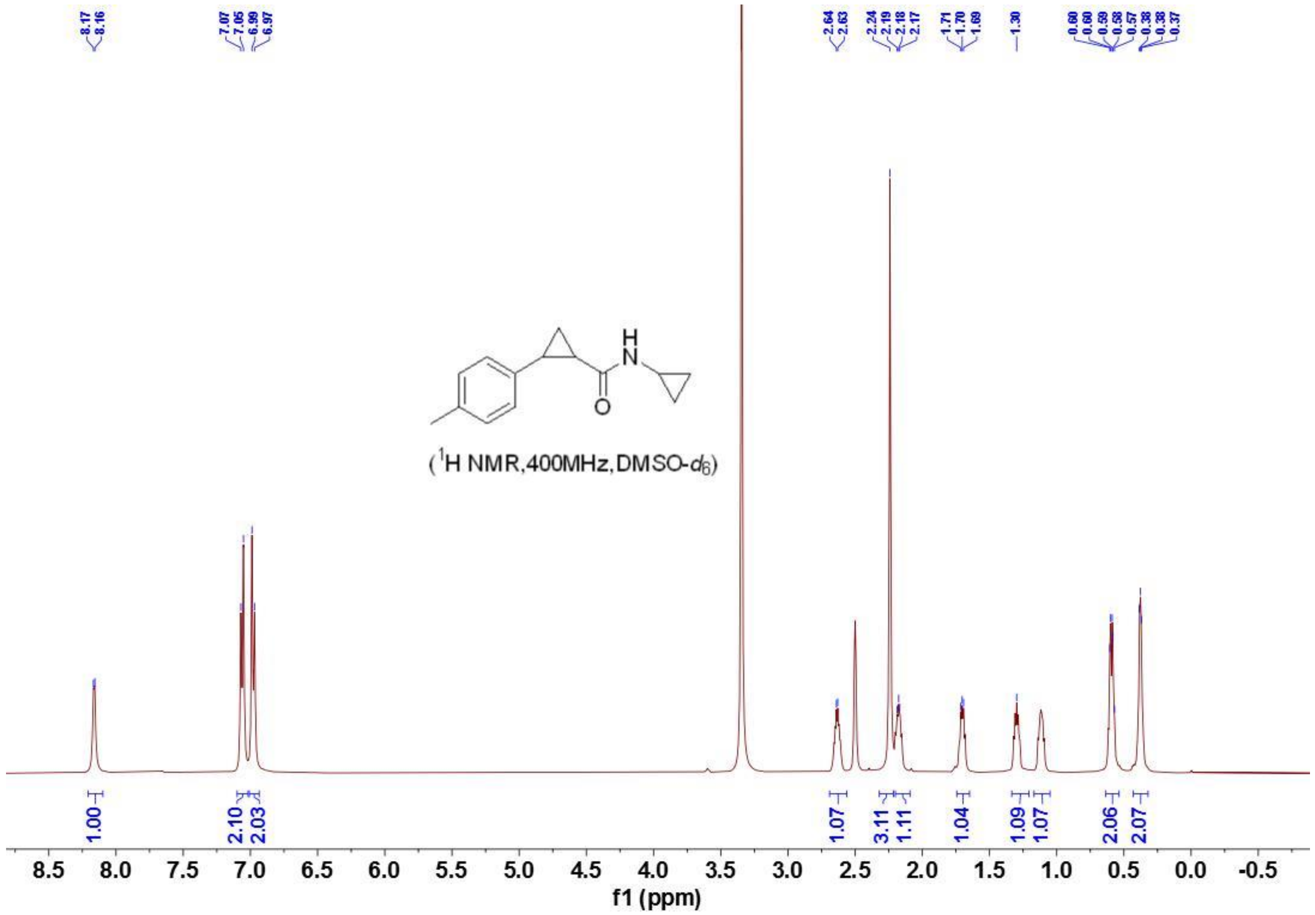

^{1}H NMR of compound **F10**

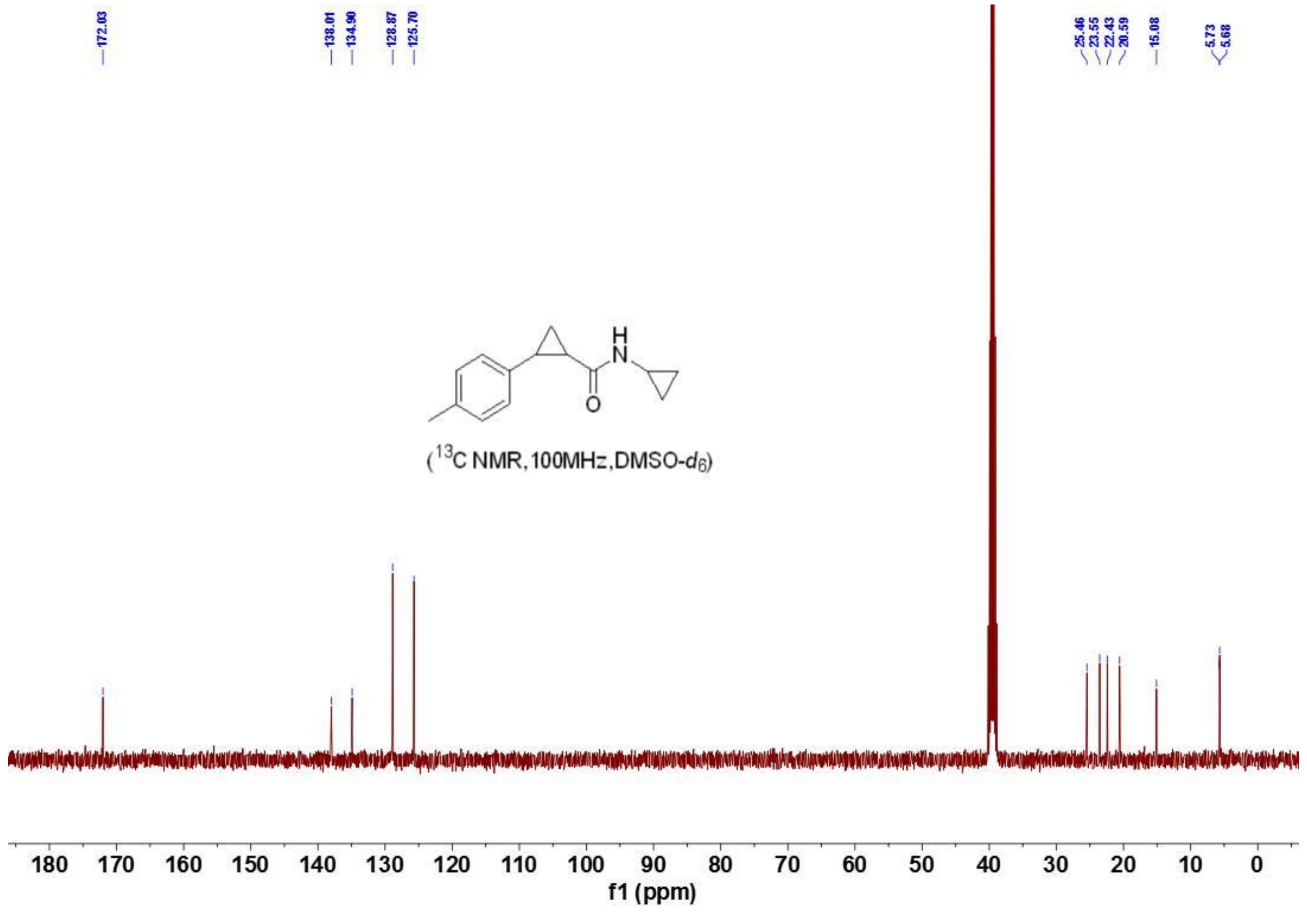

^{13}C NMR of compound **F10**

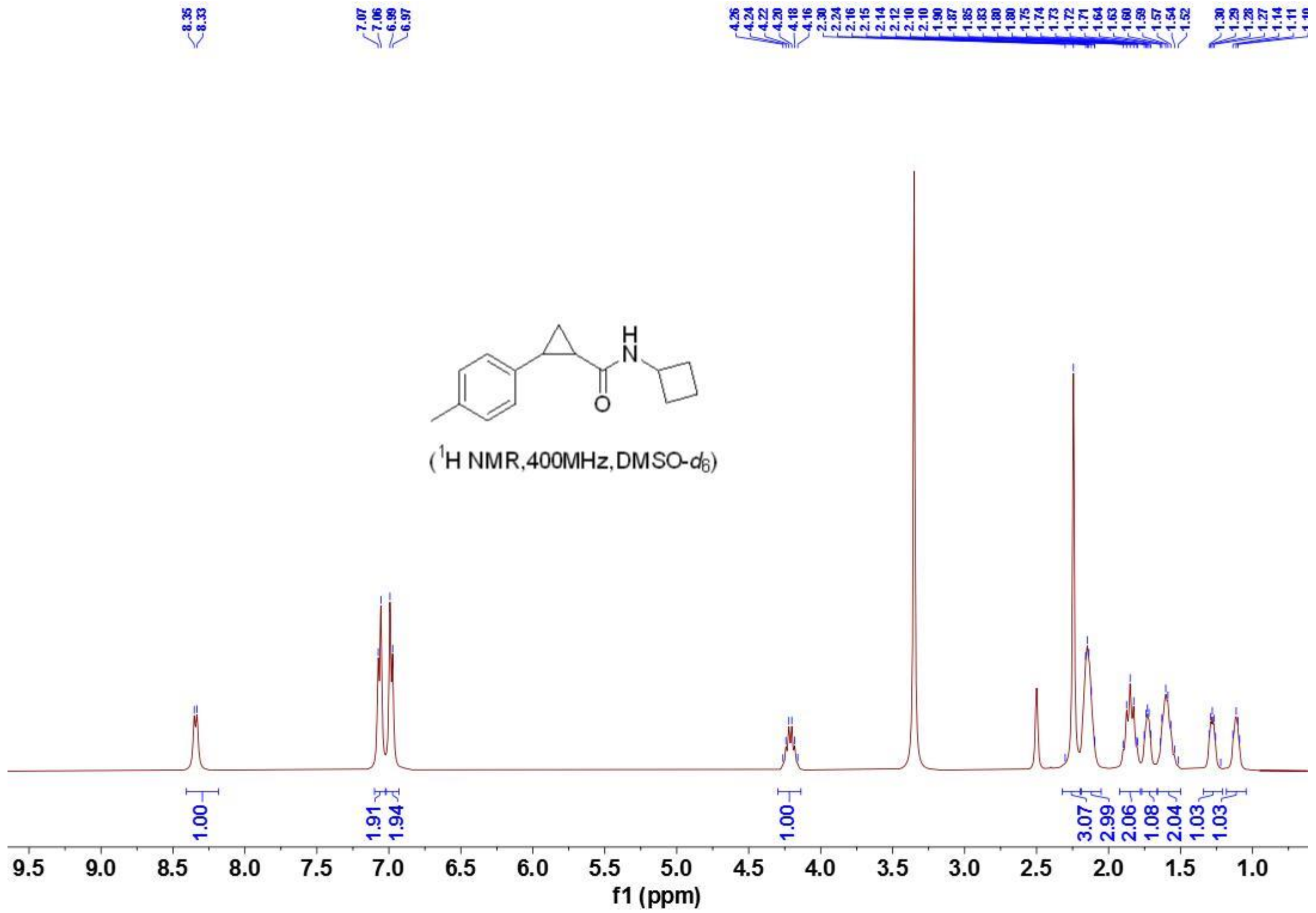

^{1}H NMR of compound **F11**

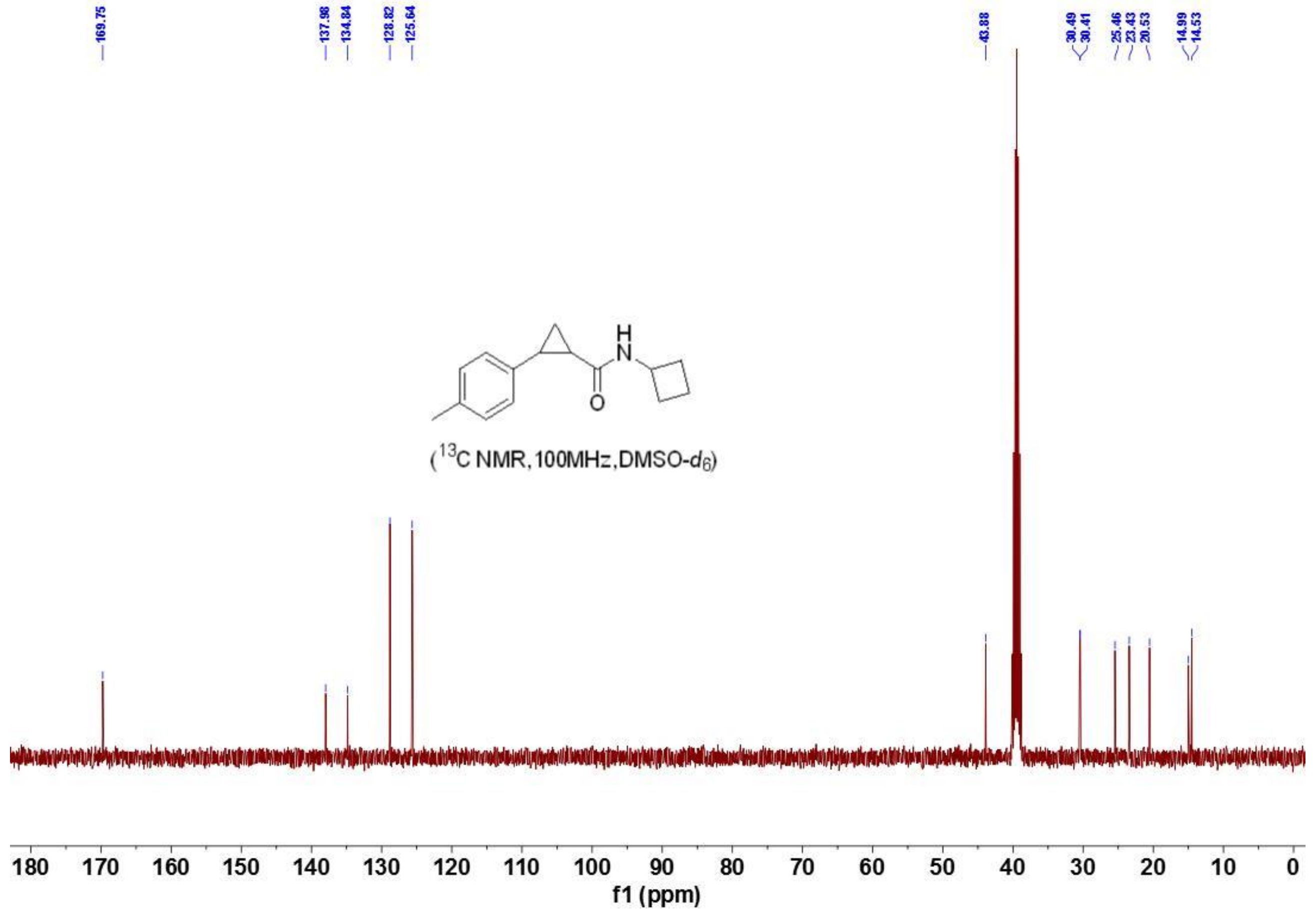

^{13}C NMR of compound **F11**

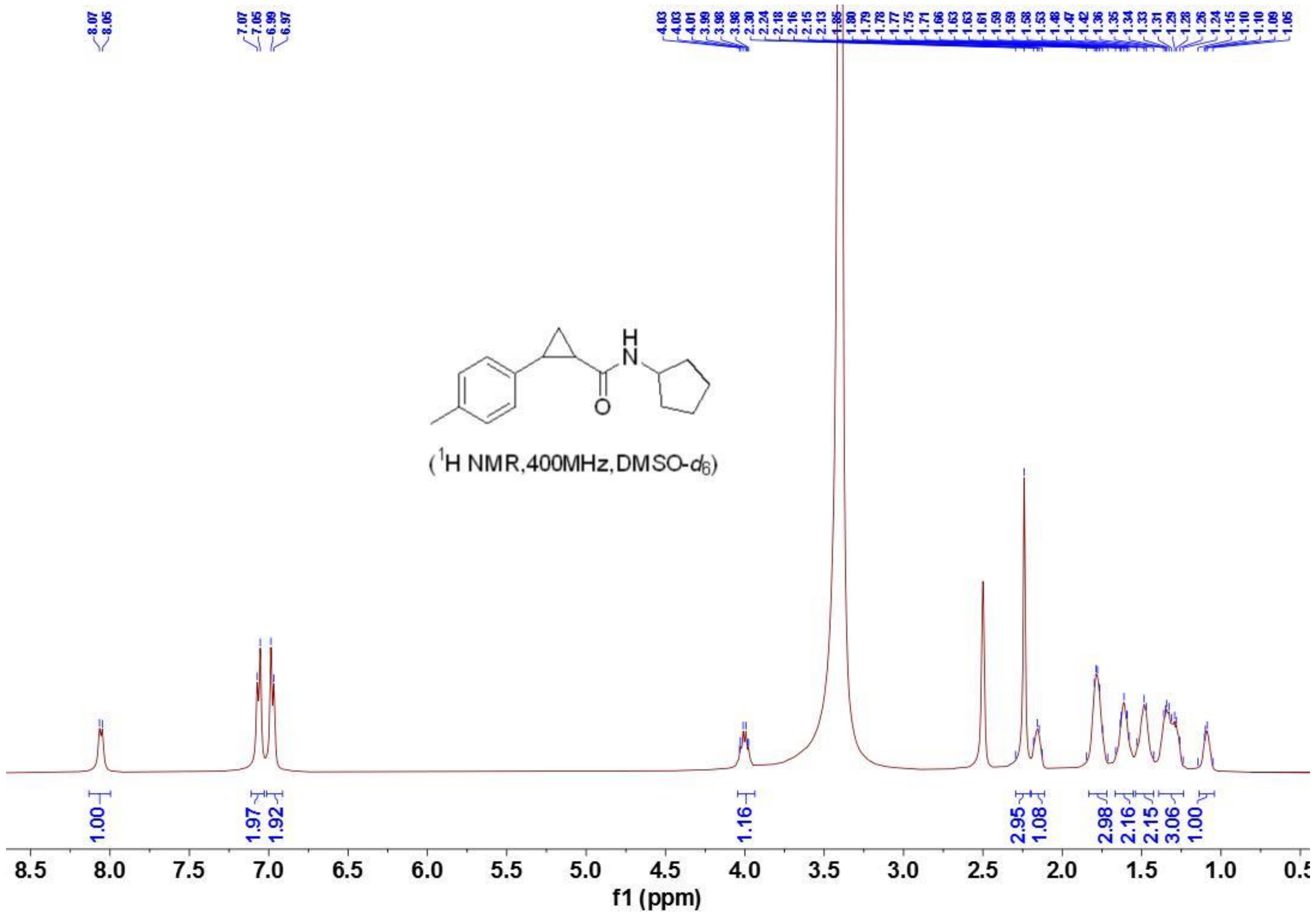

^{1}H NMR of compound **F12**

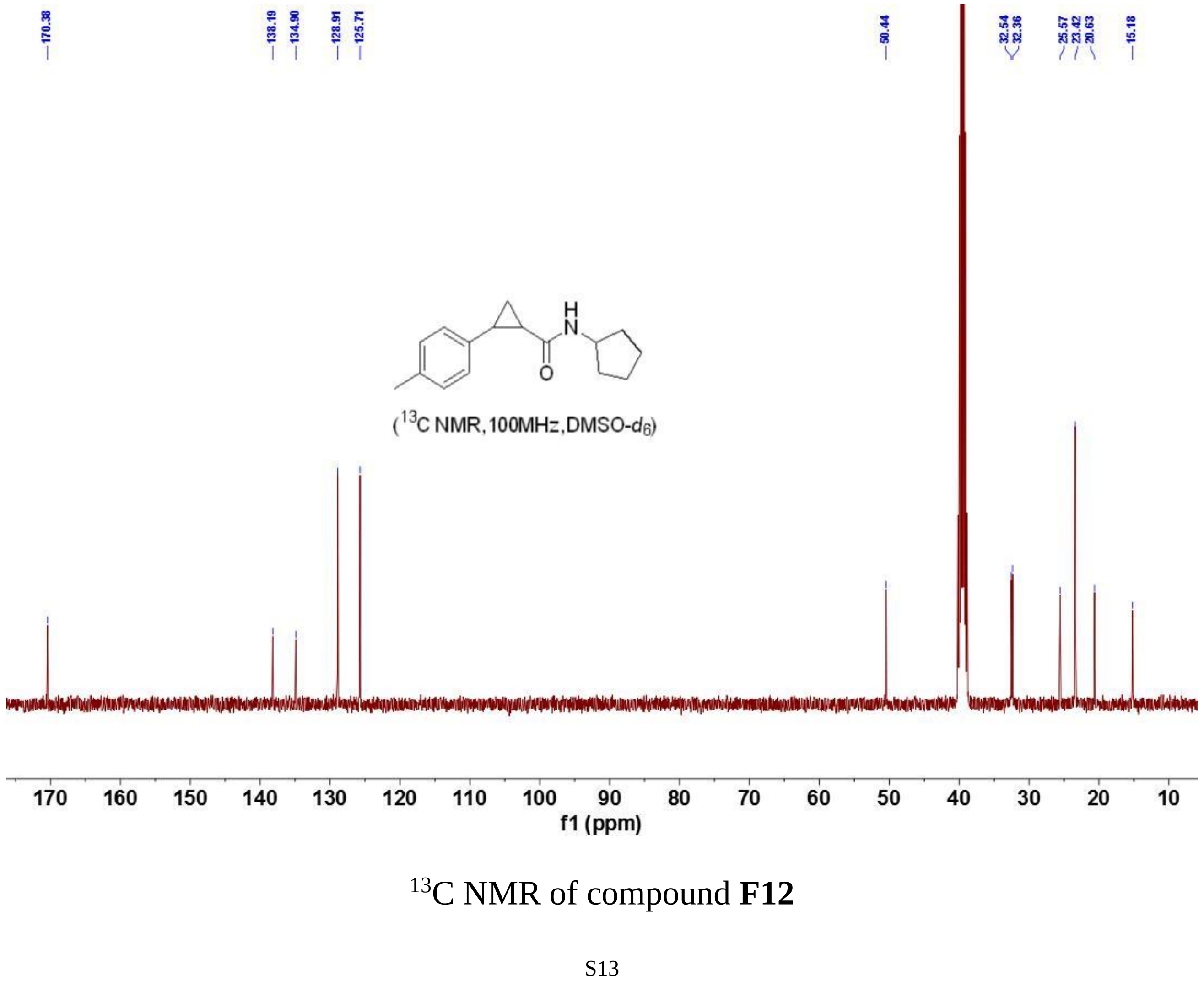

^{13}C NMR of compound **F12**

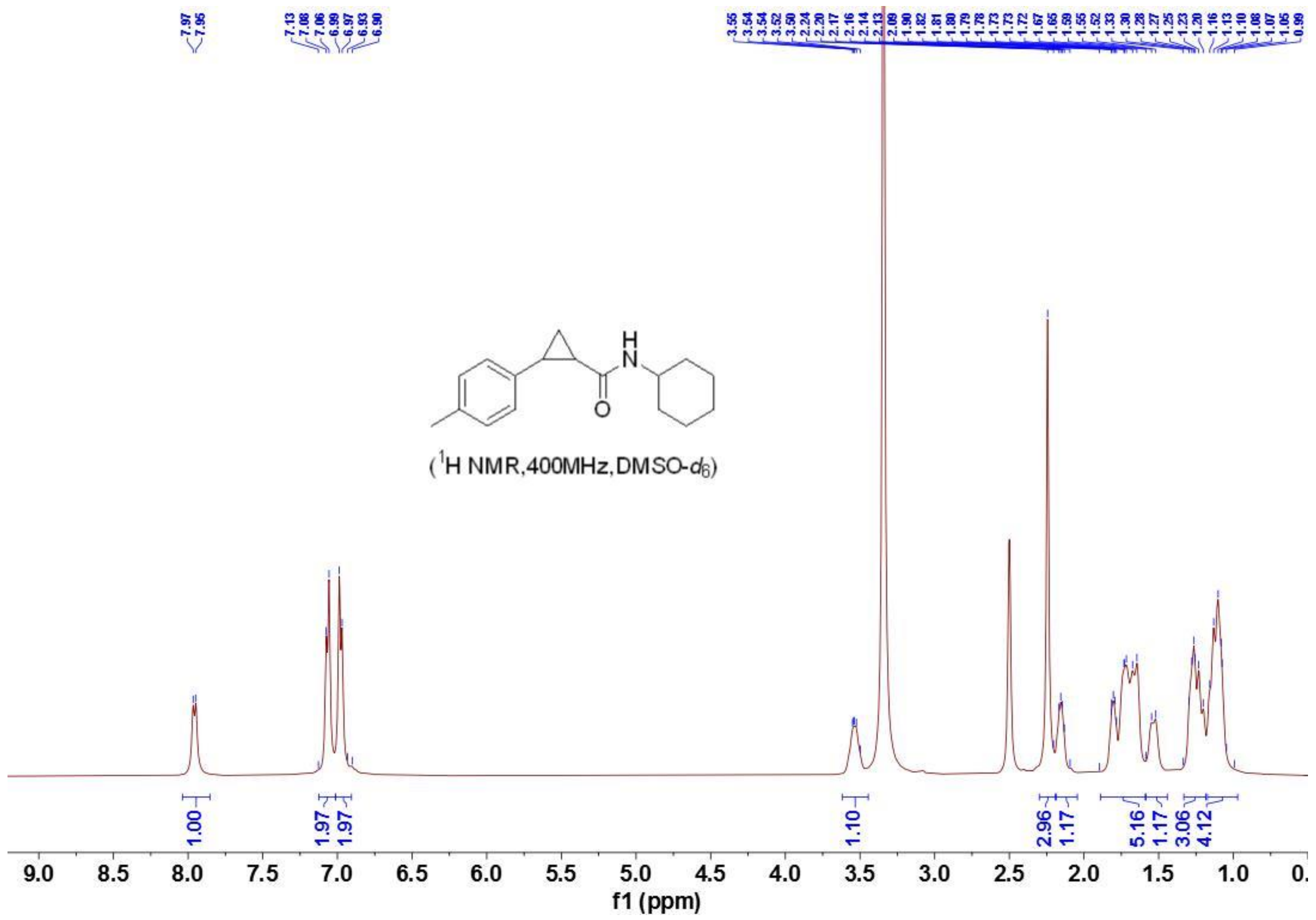

^{1}H NMR of compound **F13**

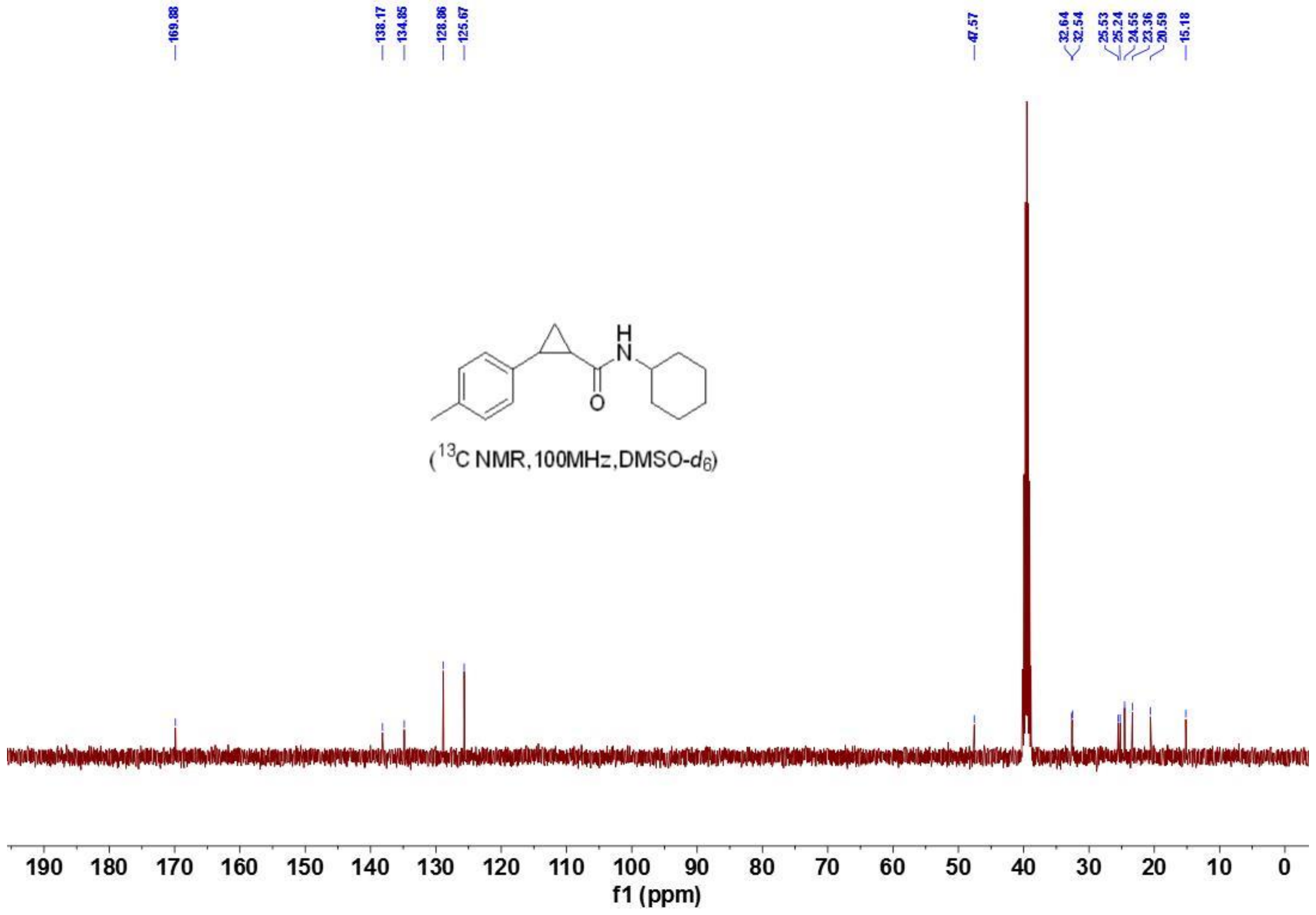

^{13}C NMR of compound **F13**

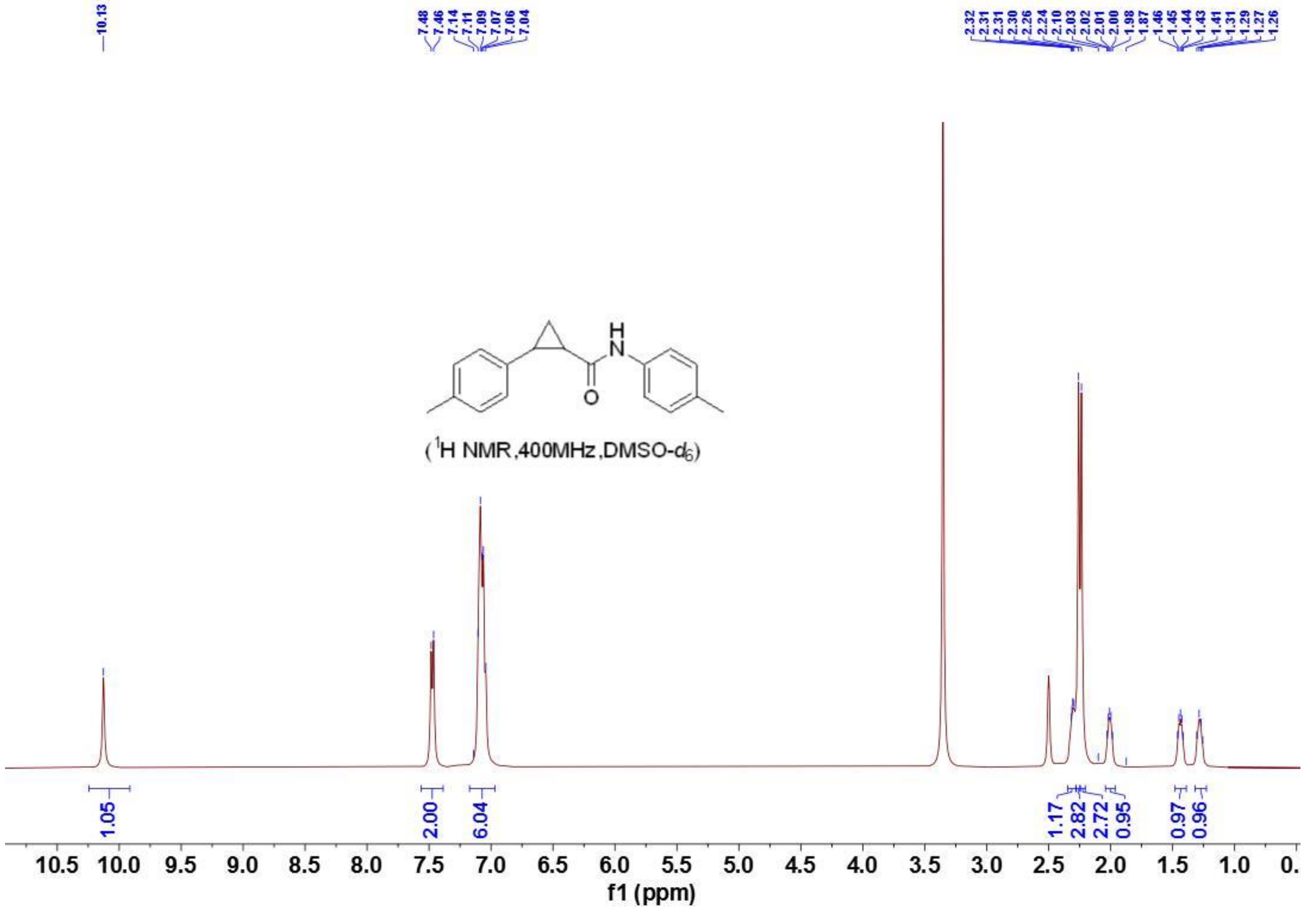

^{1}H NMR of compound **F14**

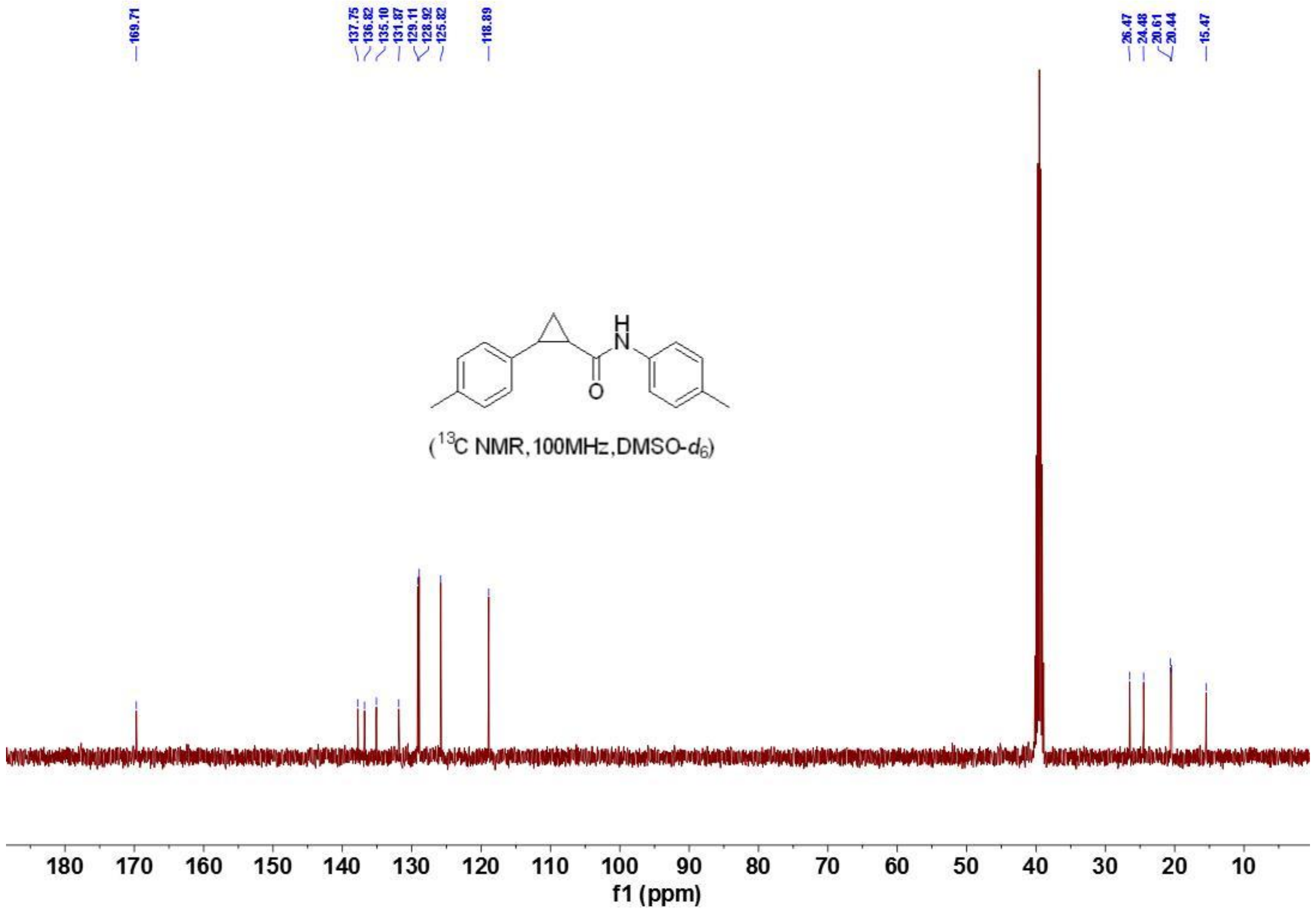

^{13}C NMR of compound **F14**

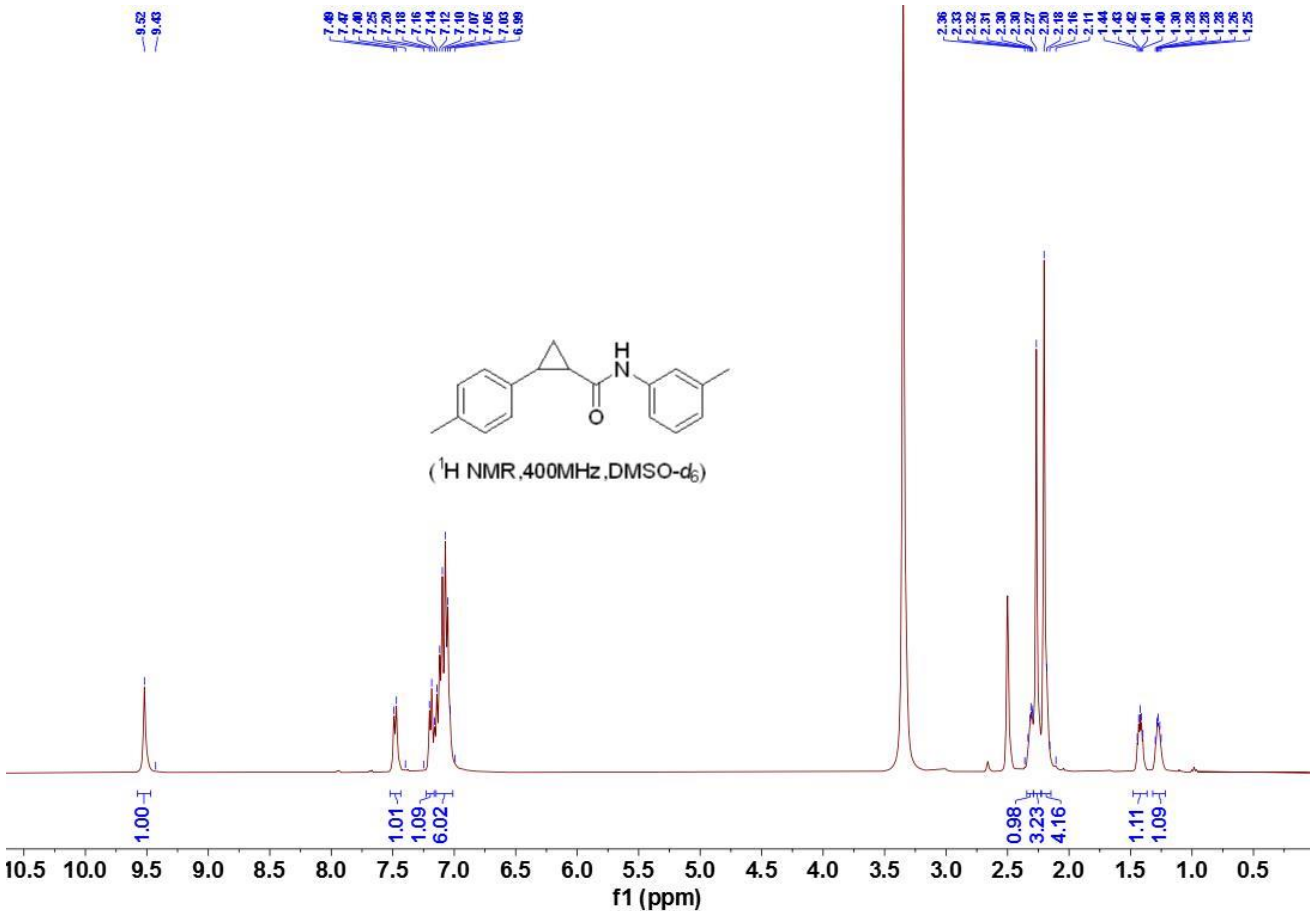

^{1}H NMR of compound **F15**

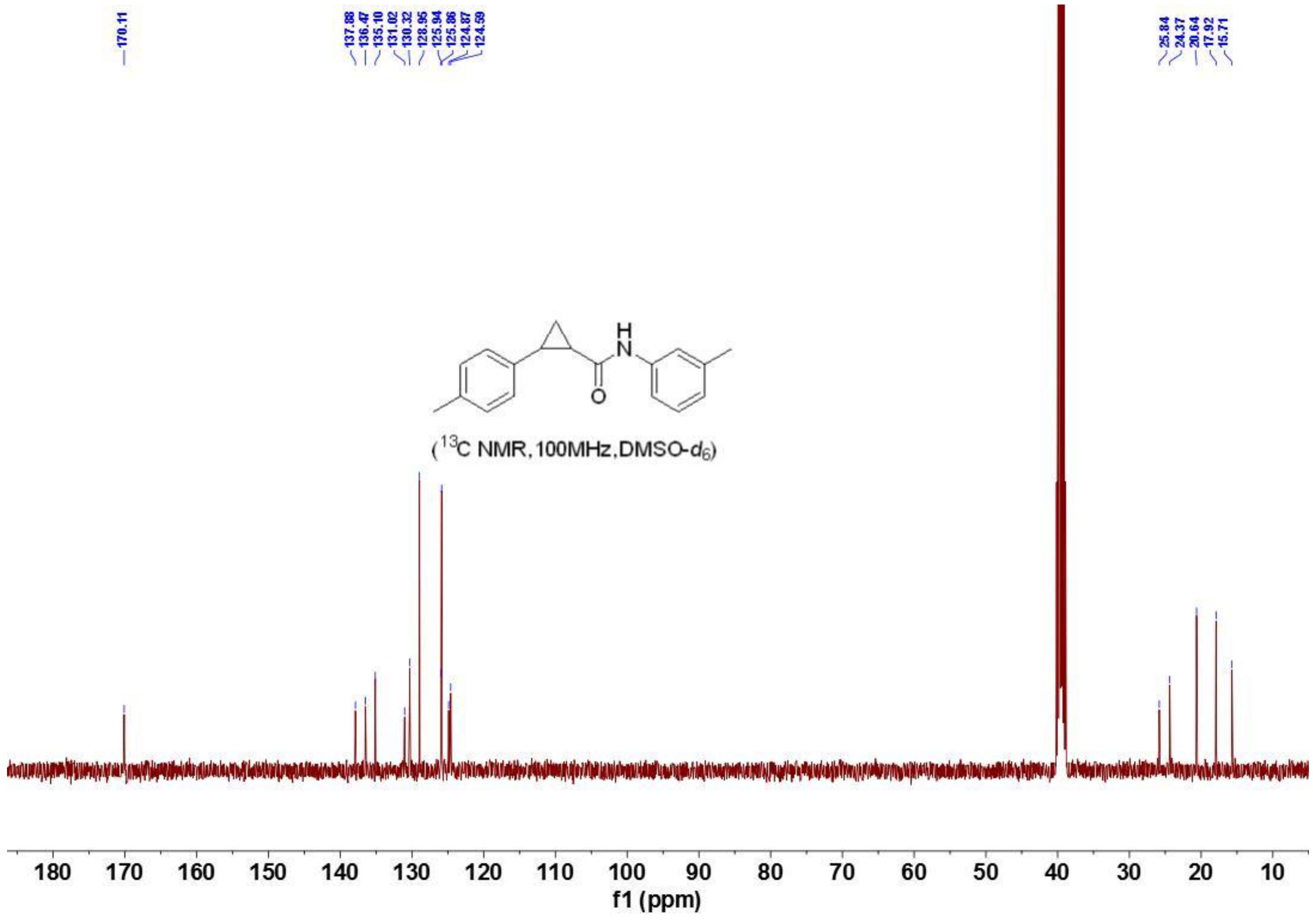

^{13}C NMR of compound **F15**

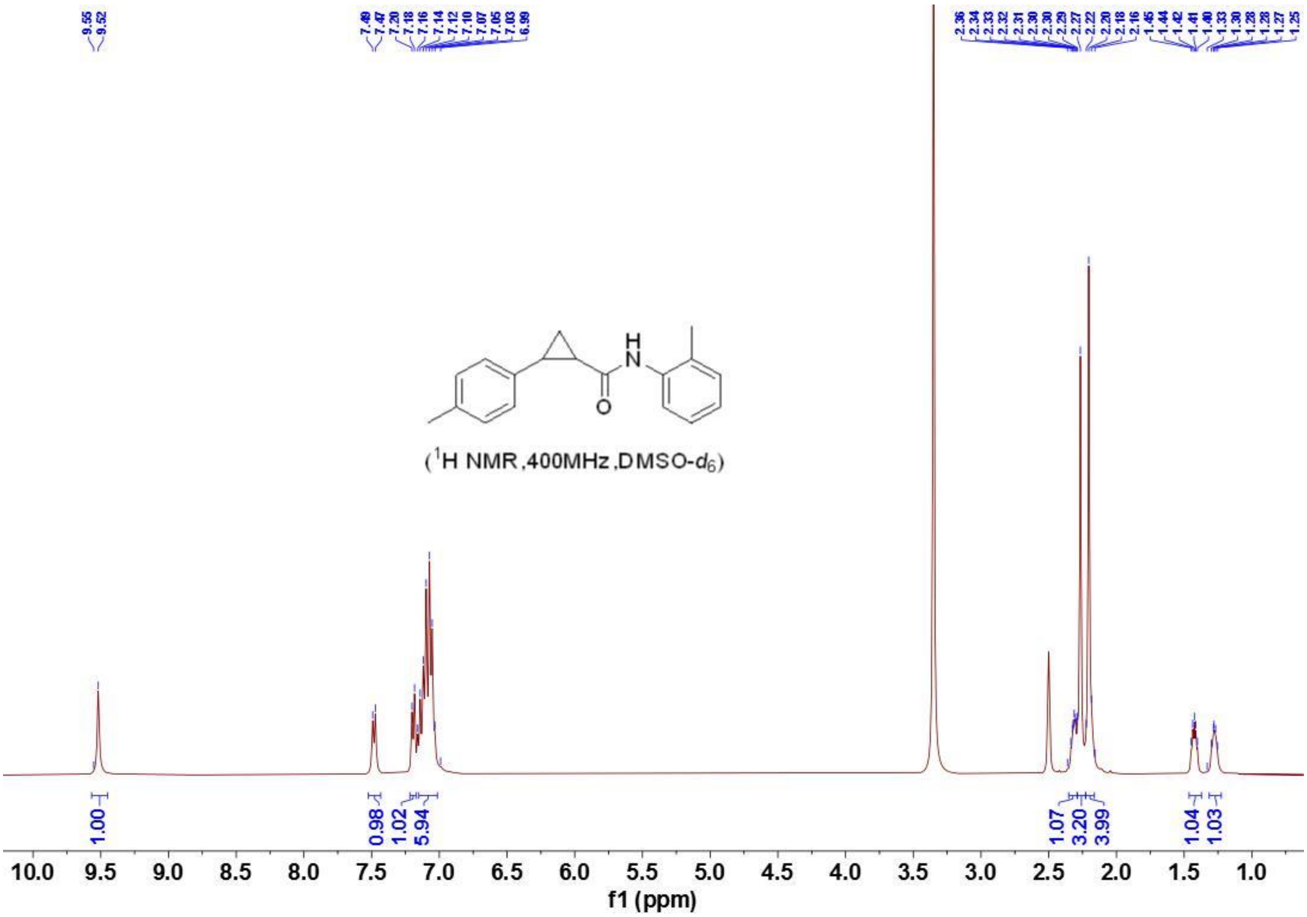

^{1}H NMR of compound **F16**

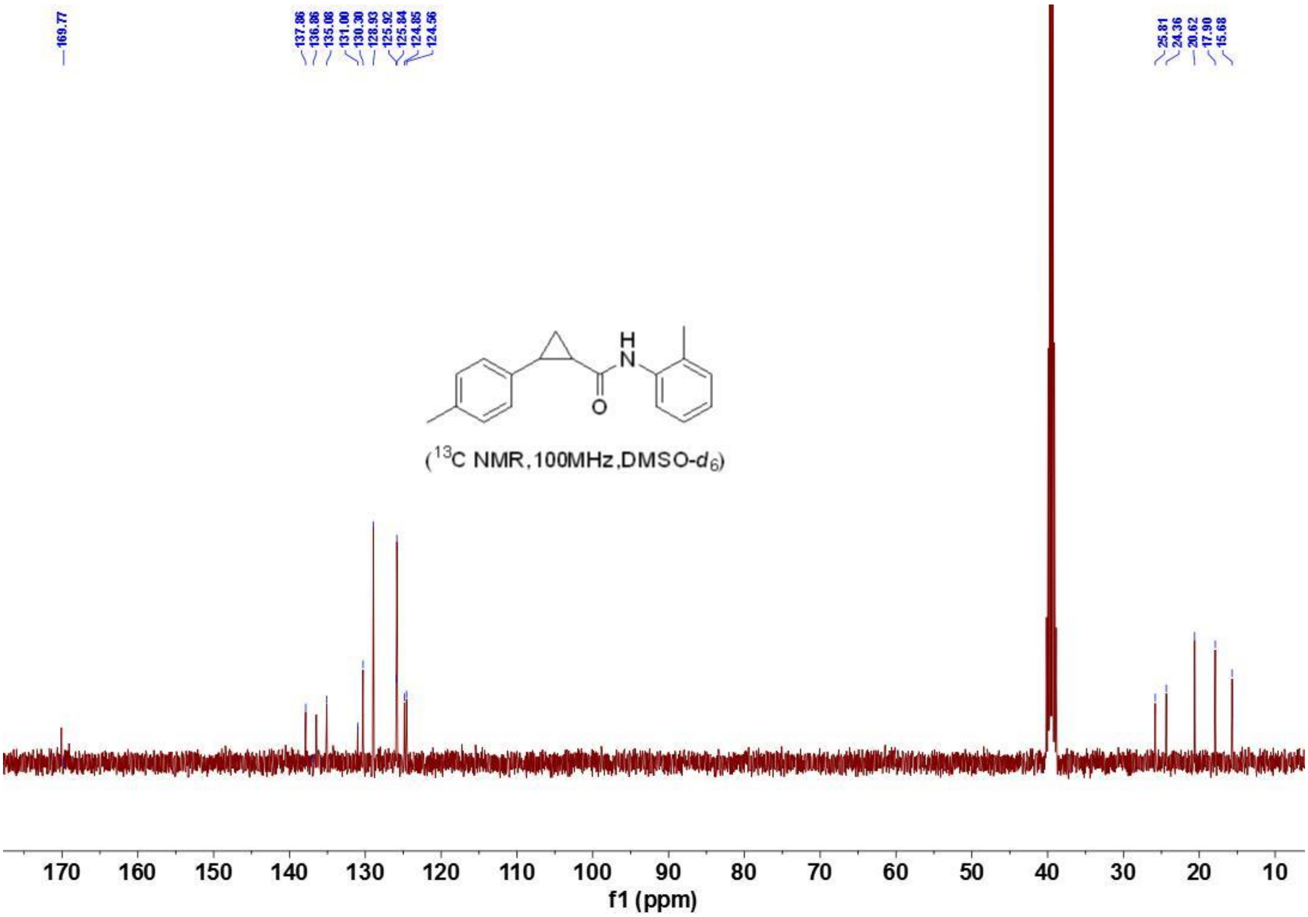

^{13}C NMR of compound **F16**

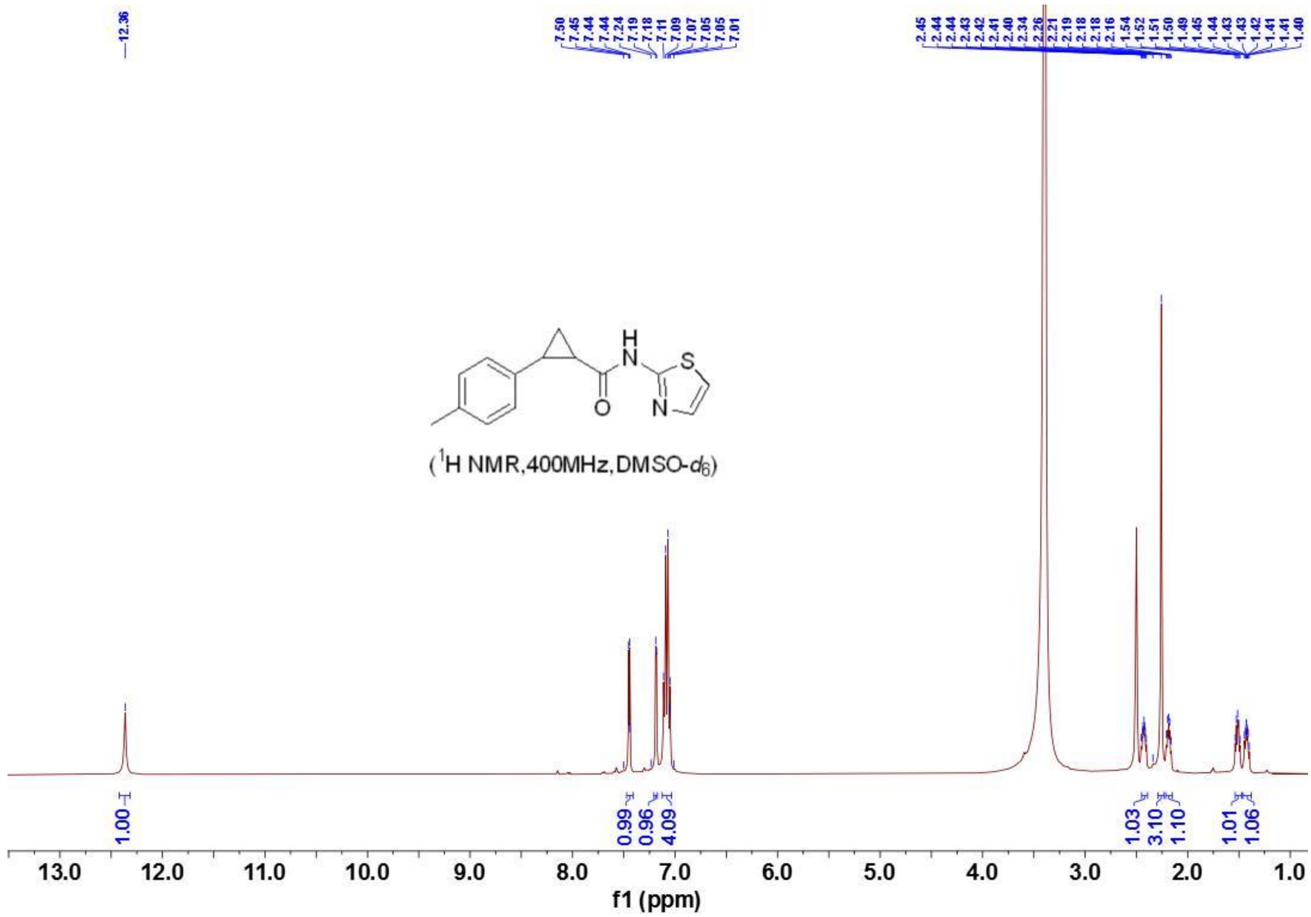

^{1}H NMR of compound **F17**

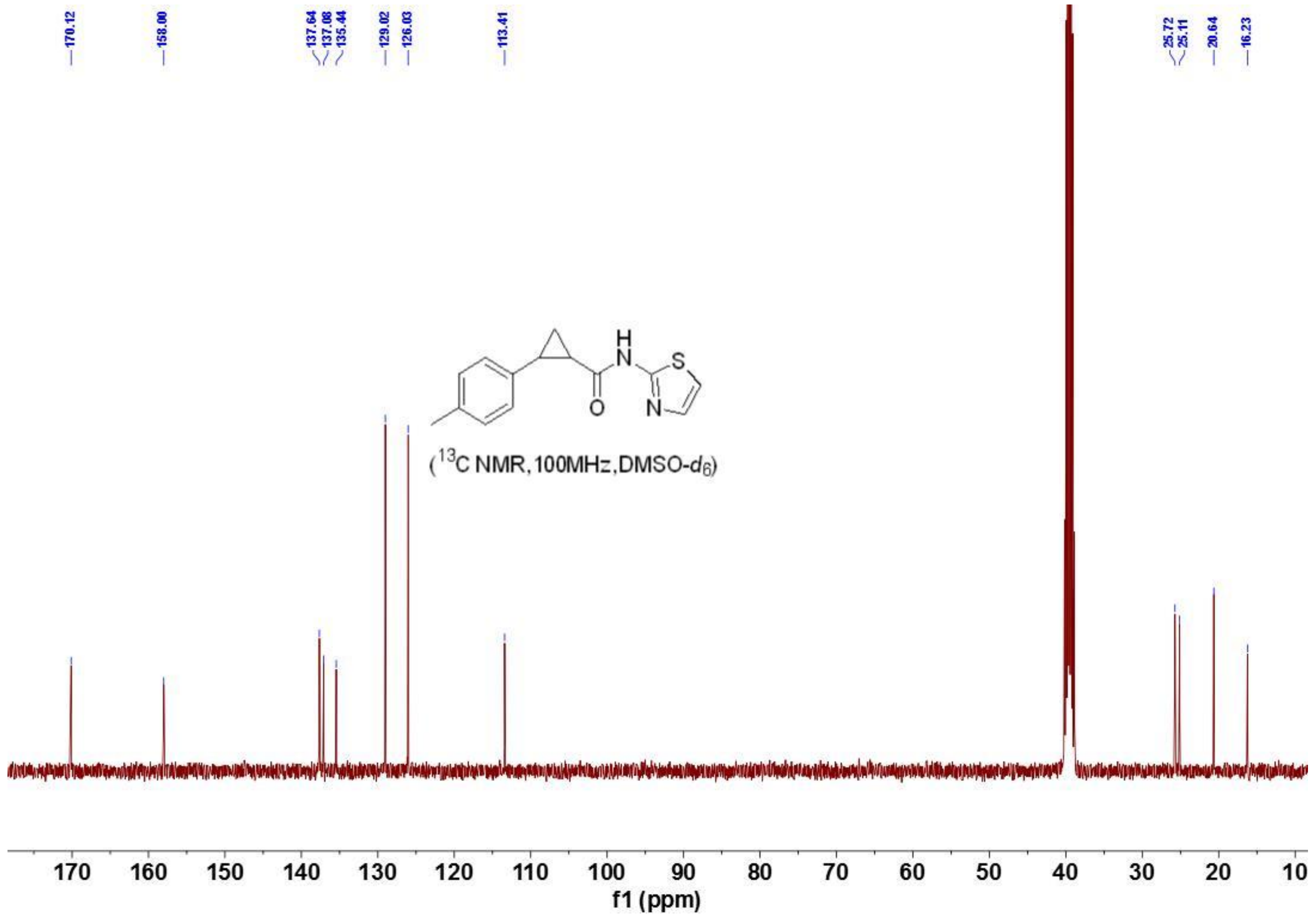

^{13}C NMR of compound **F17**

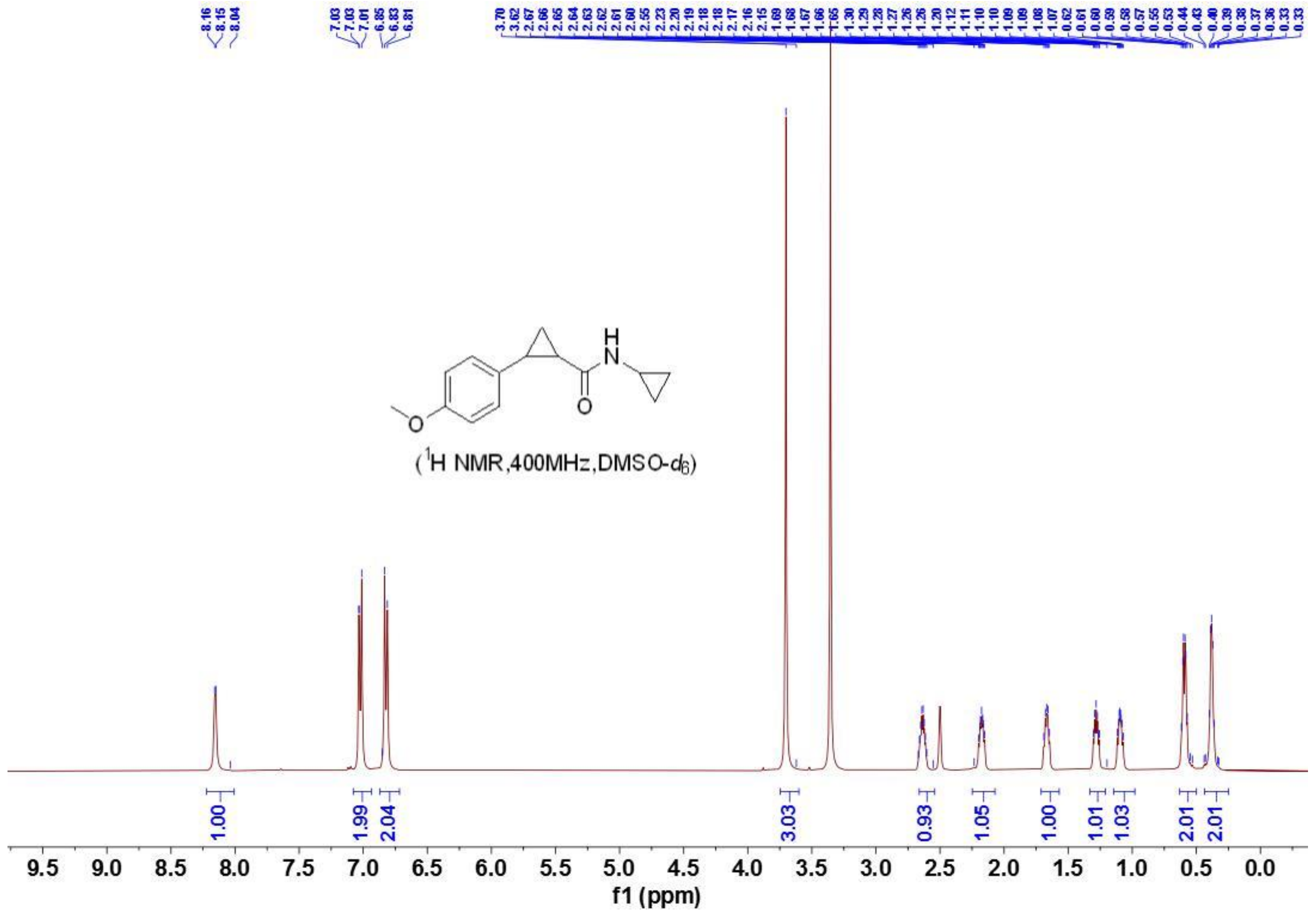

^{1}H NMR of compound **F18**

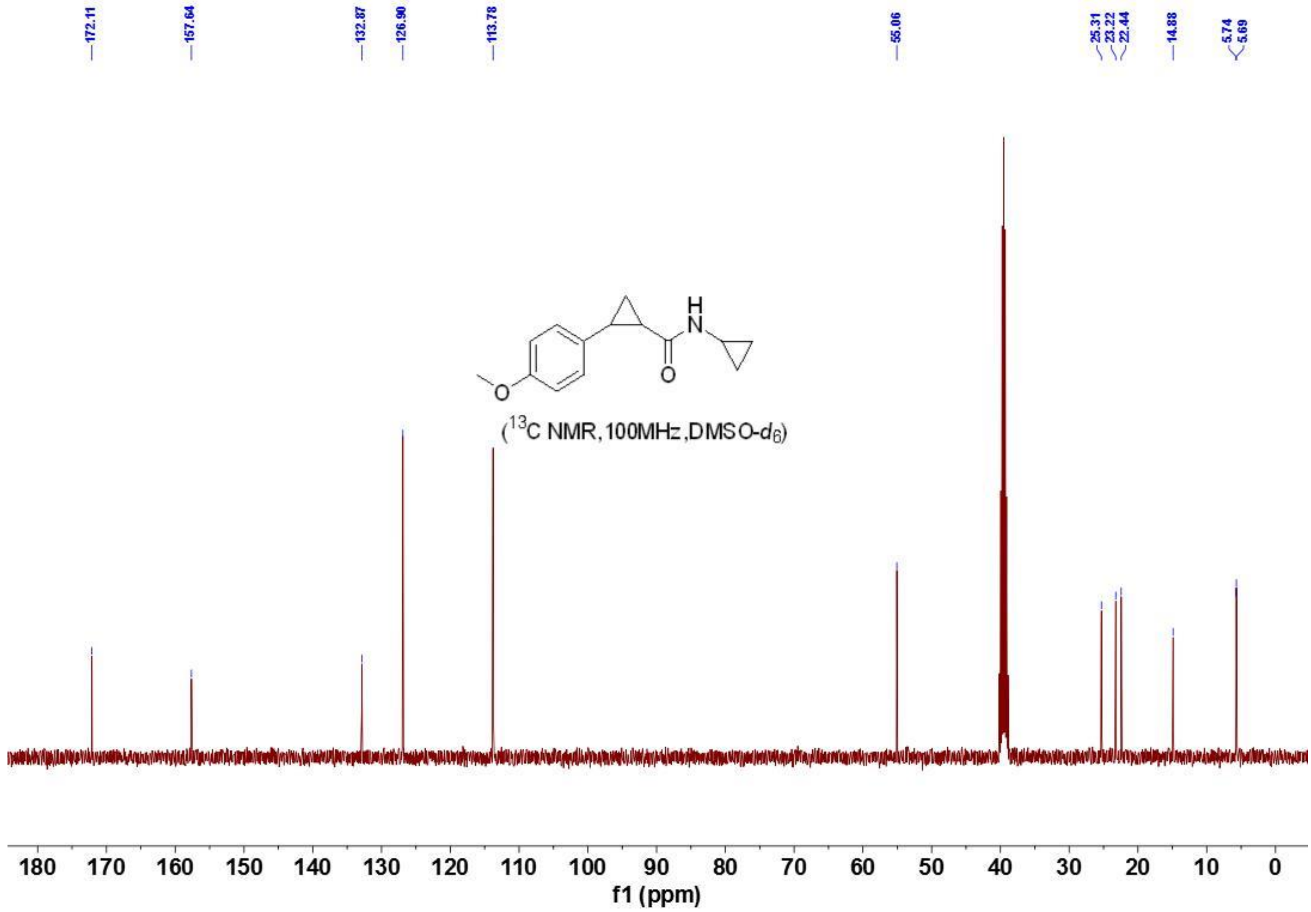

^{13}C NMR of compound **F18**

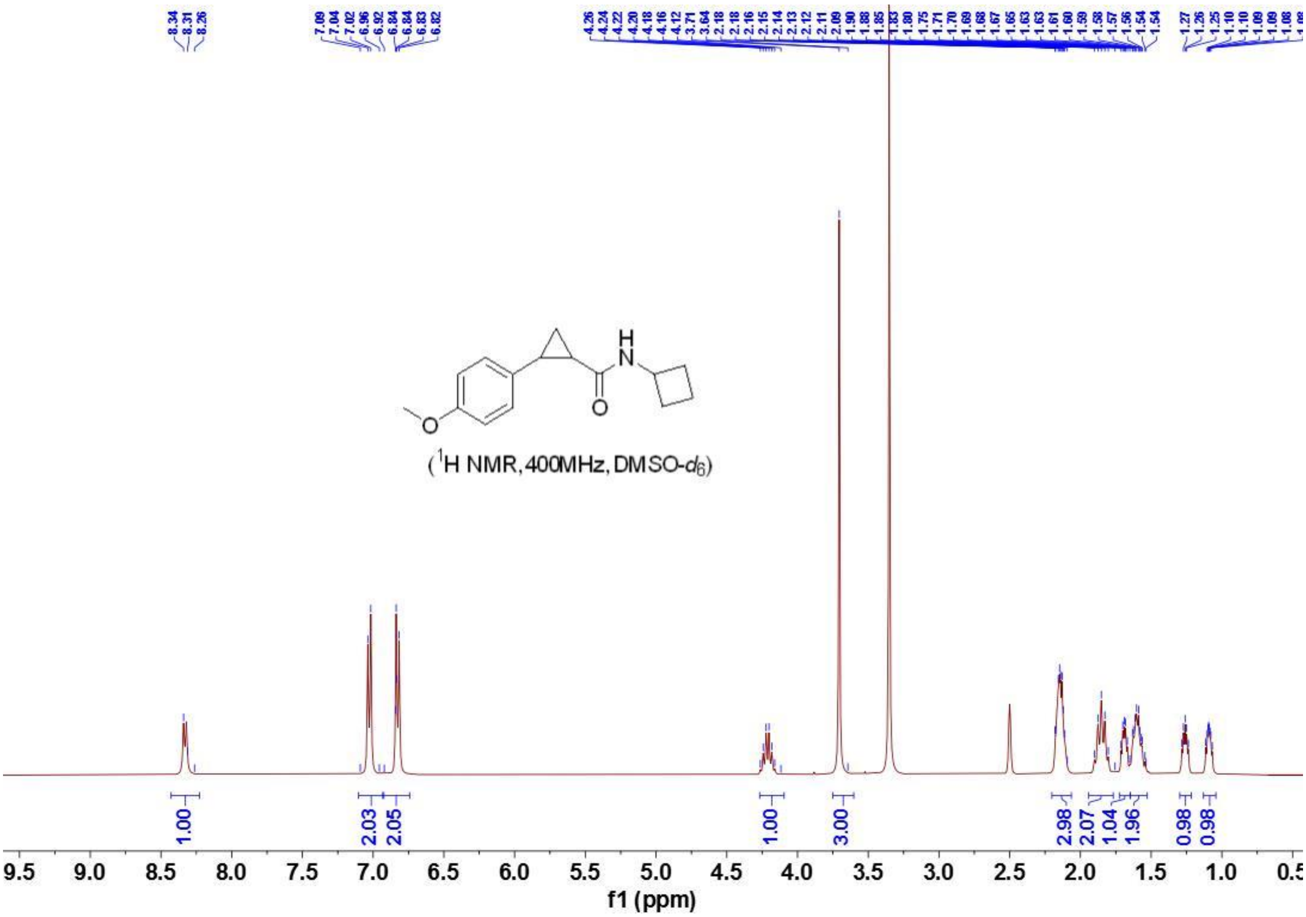

^{1}H NMR of compound **F19**

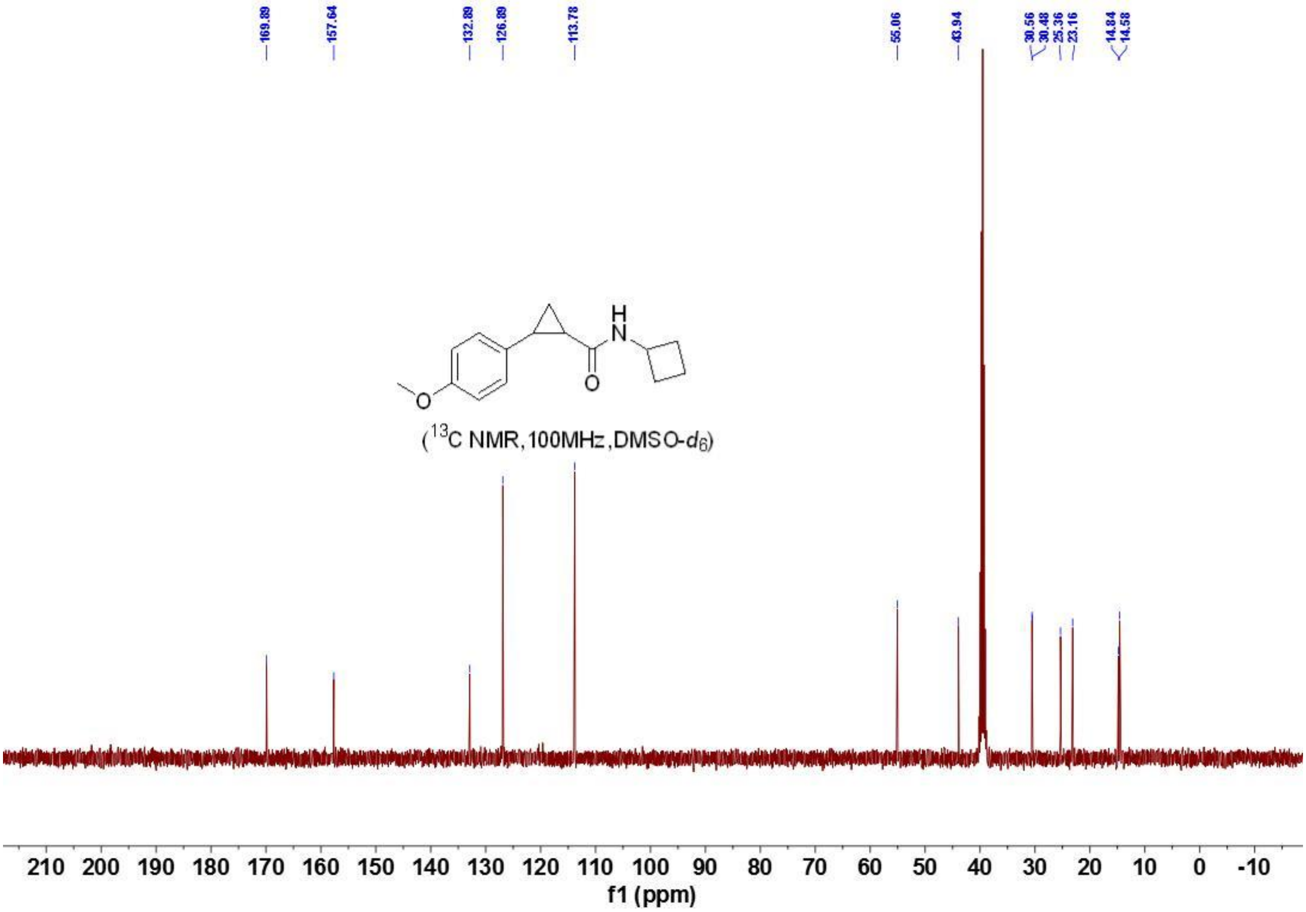

^{13}C NMR of compound **F19**

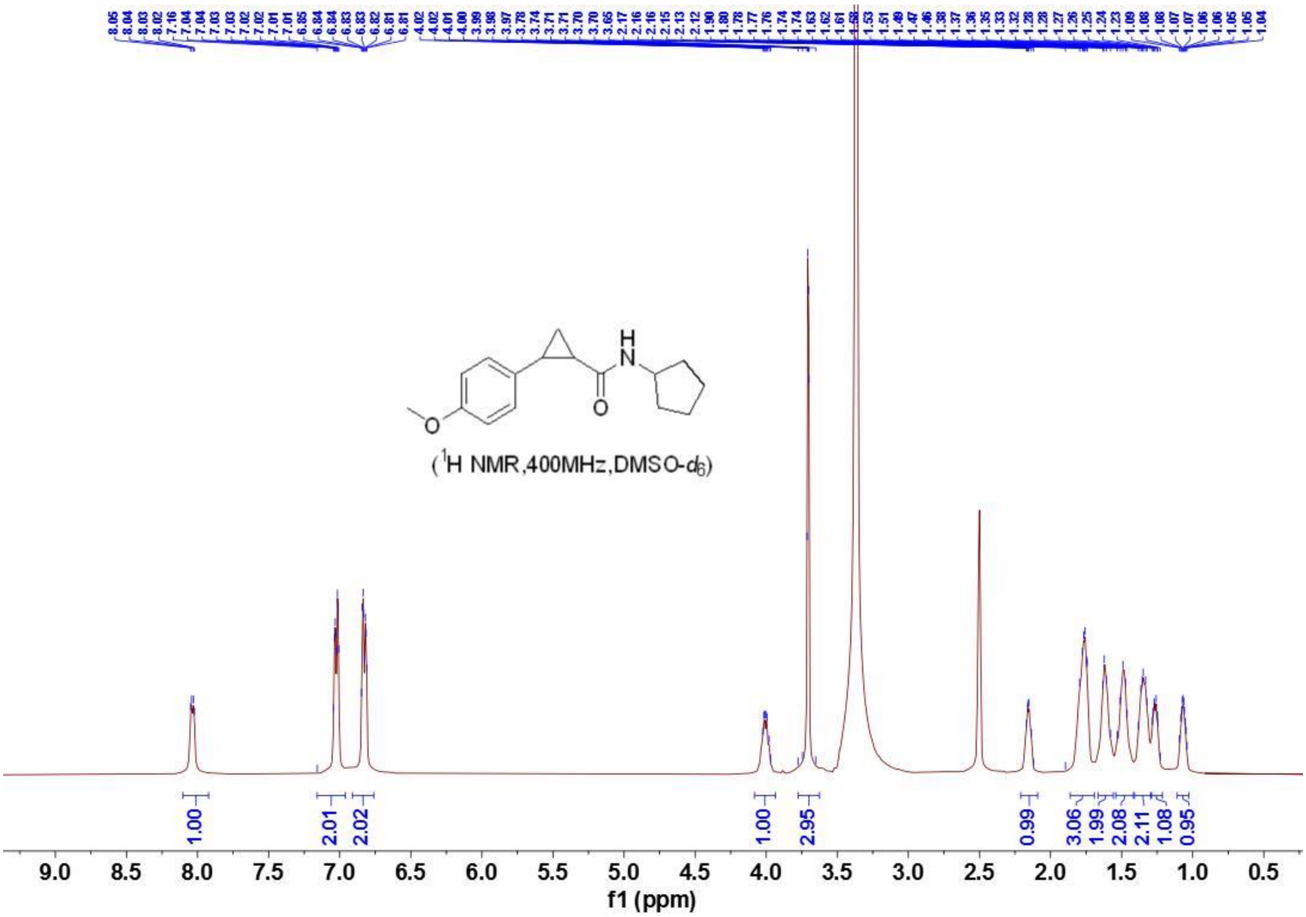

^{1}H NMR of compound **F20**

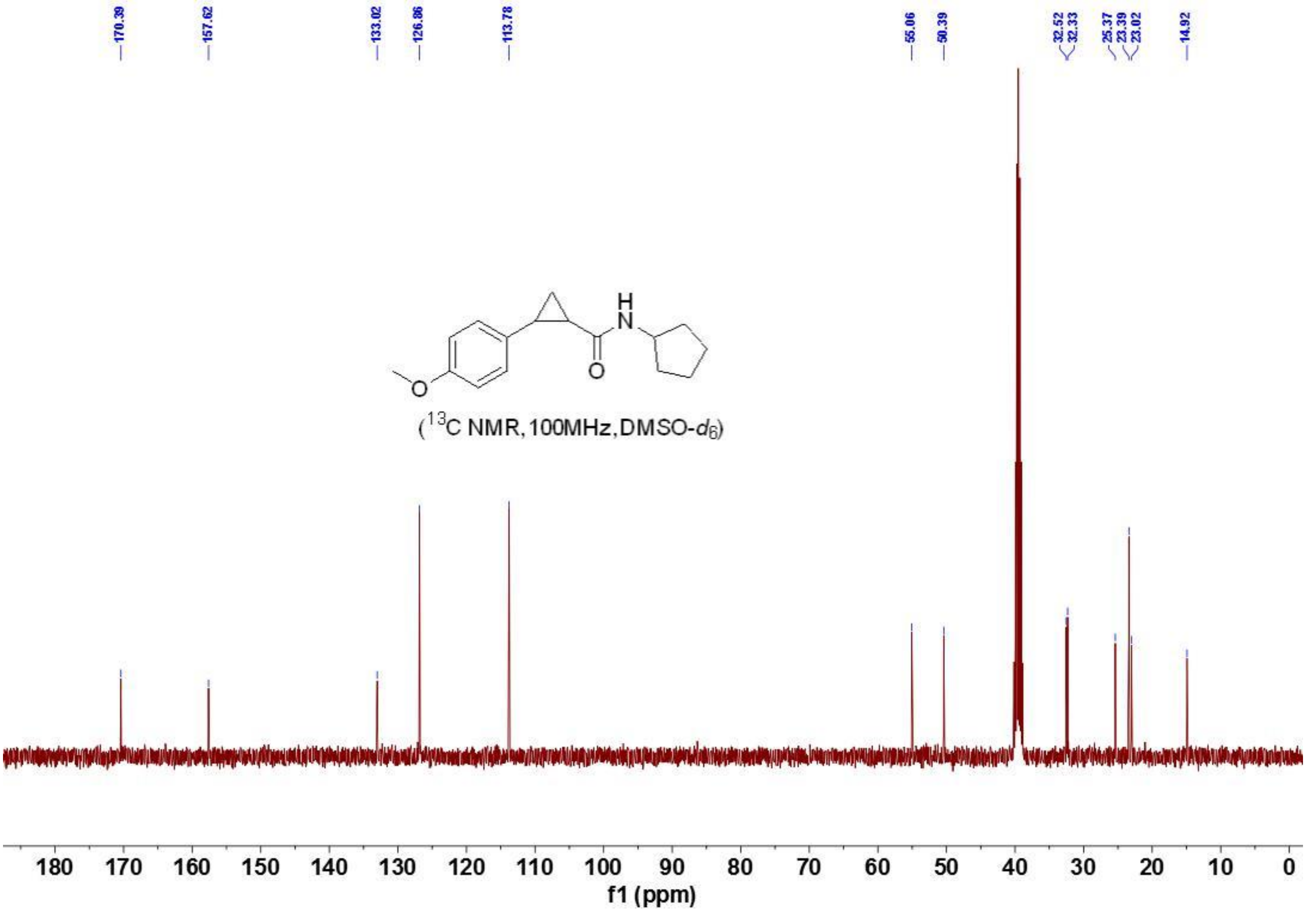

^{13}C NMR of compound **F20**

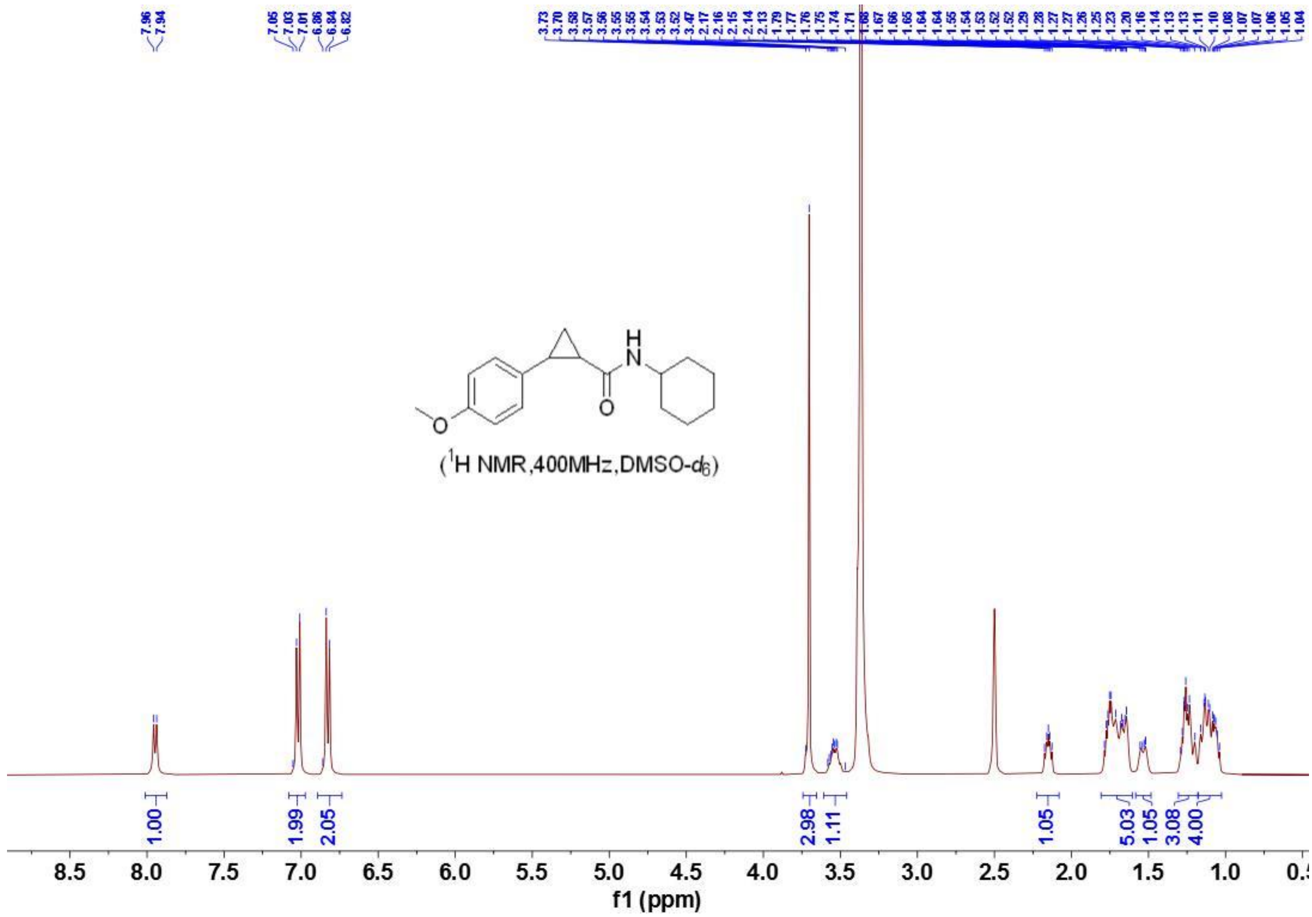

^{1}H NMR of compound **F21**

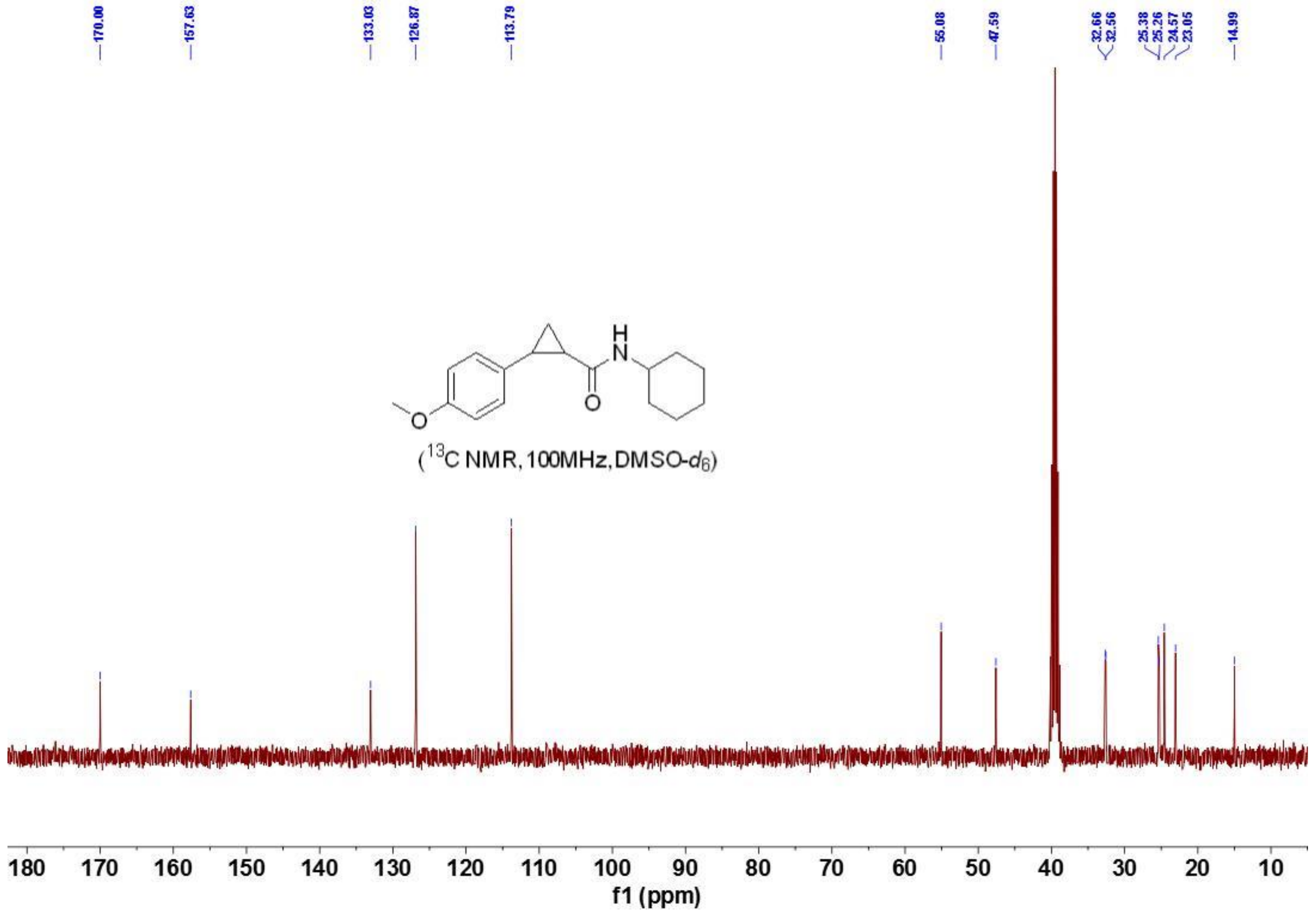

^{13}C NMR of compound **F21**

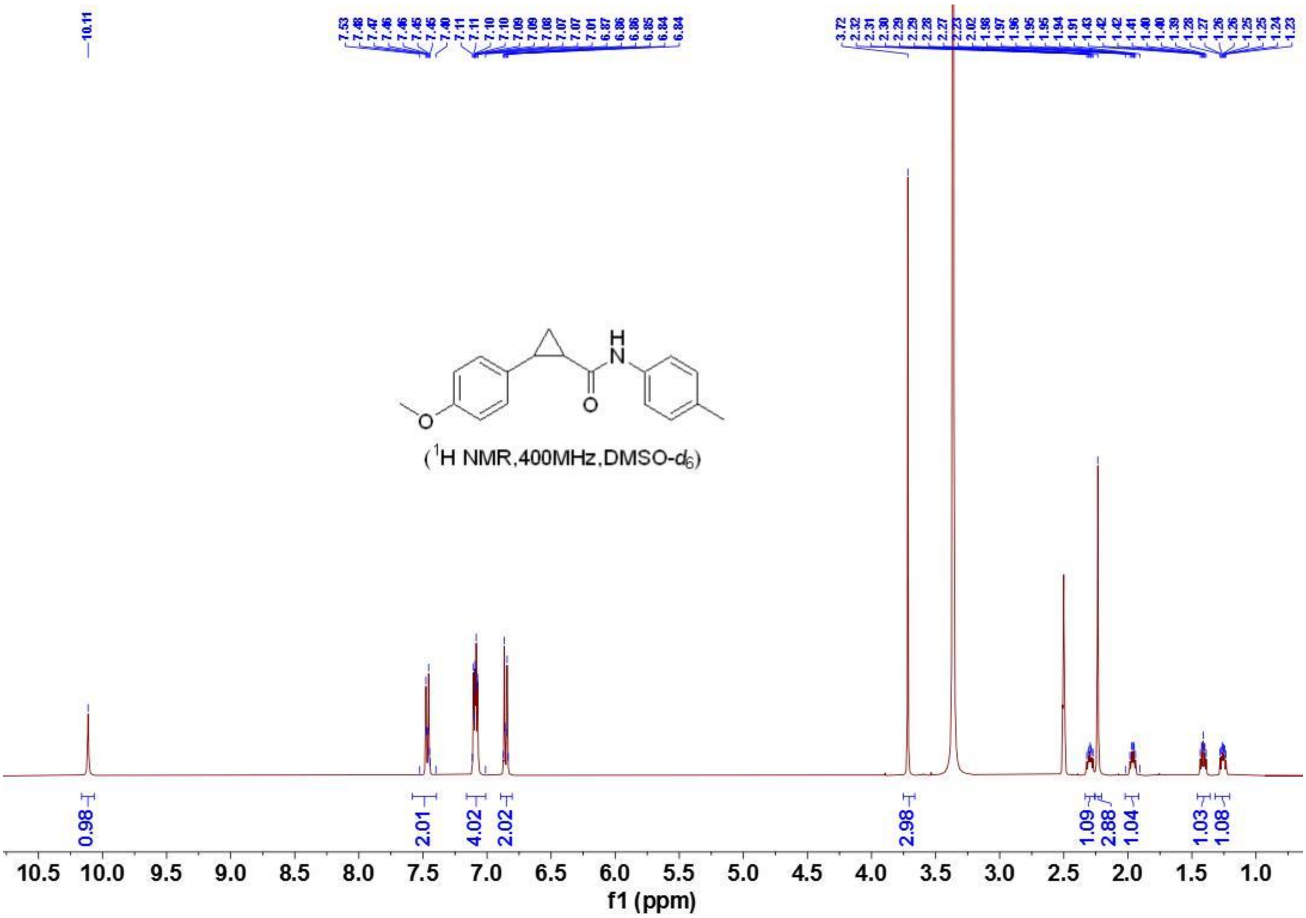

^{1}H NMR of compound **F22**

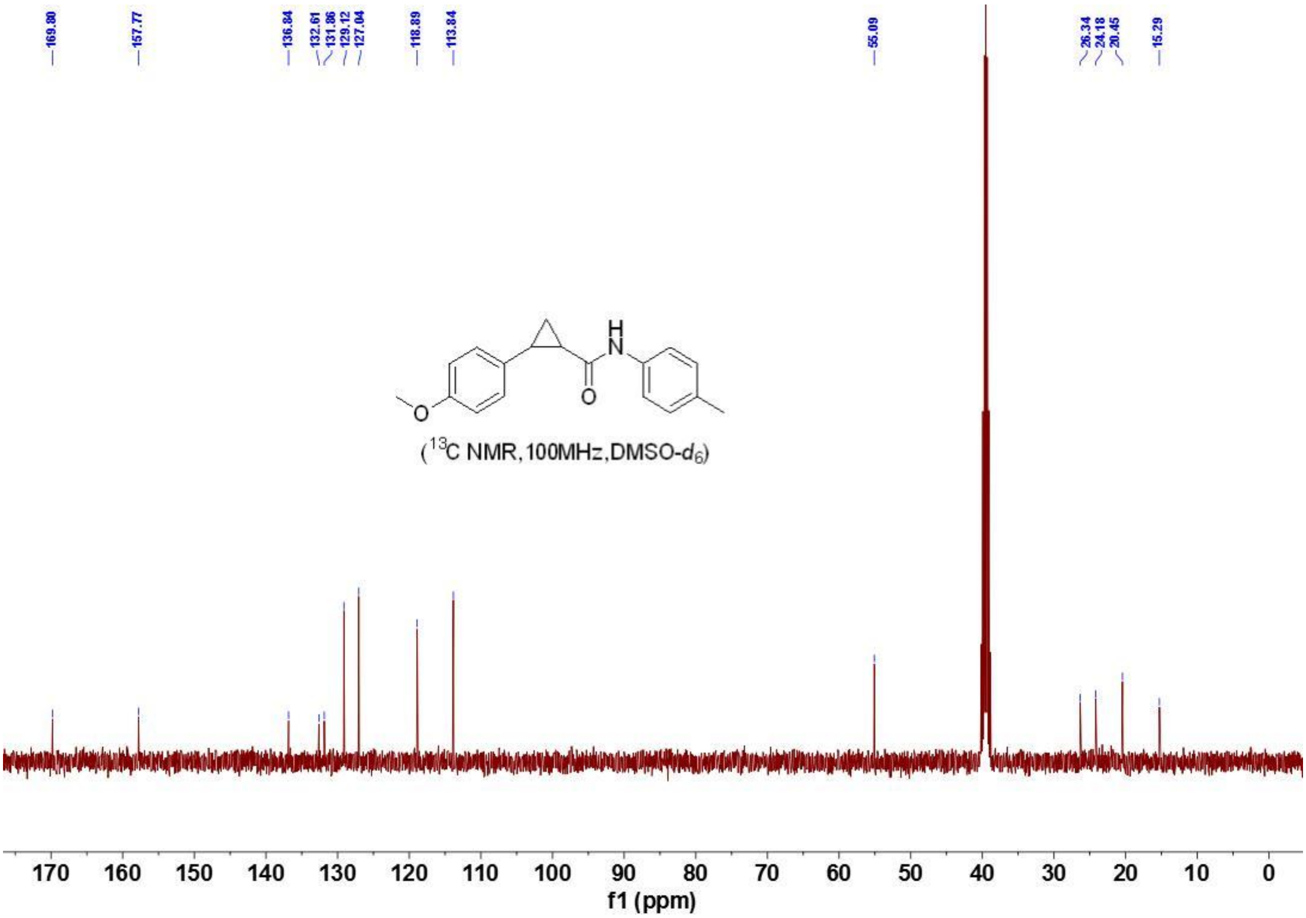

^{13}C NMR of compound **F22**

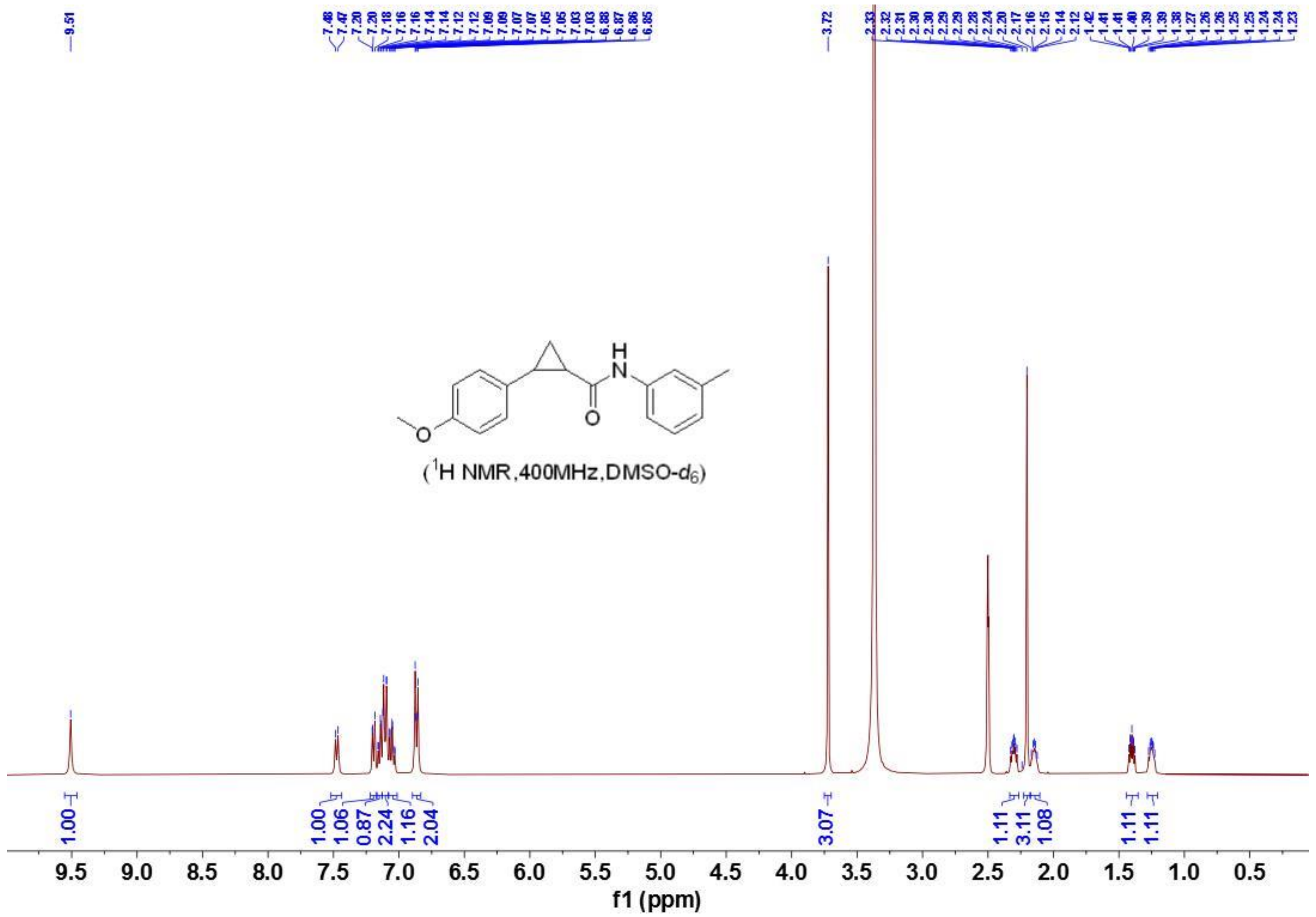

^{1}H NMR of compound **F23**

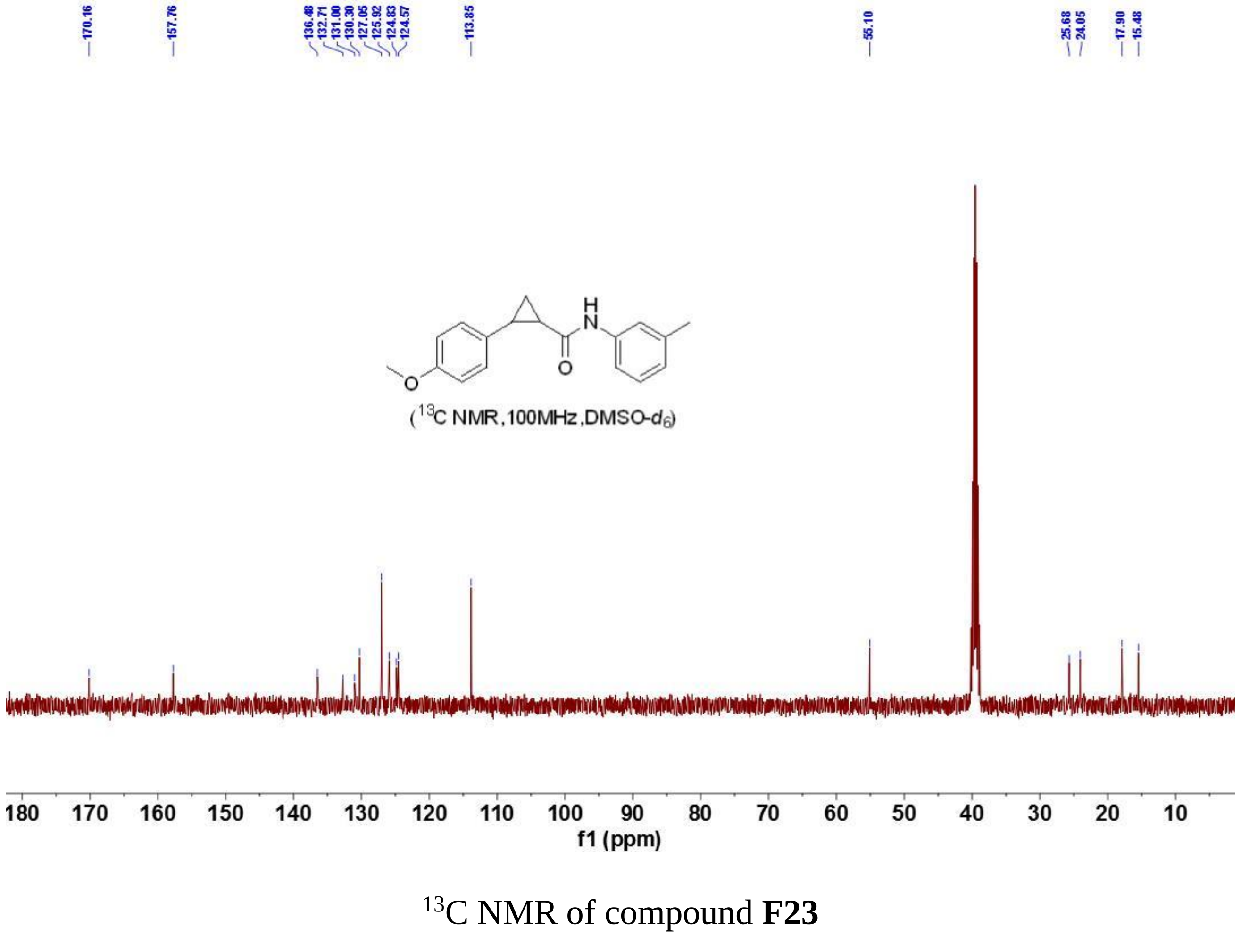

^{13}C NMR of compound **F23**

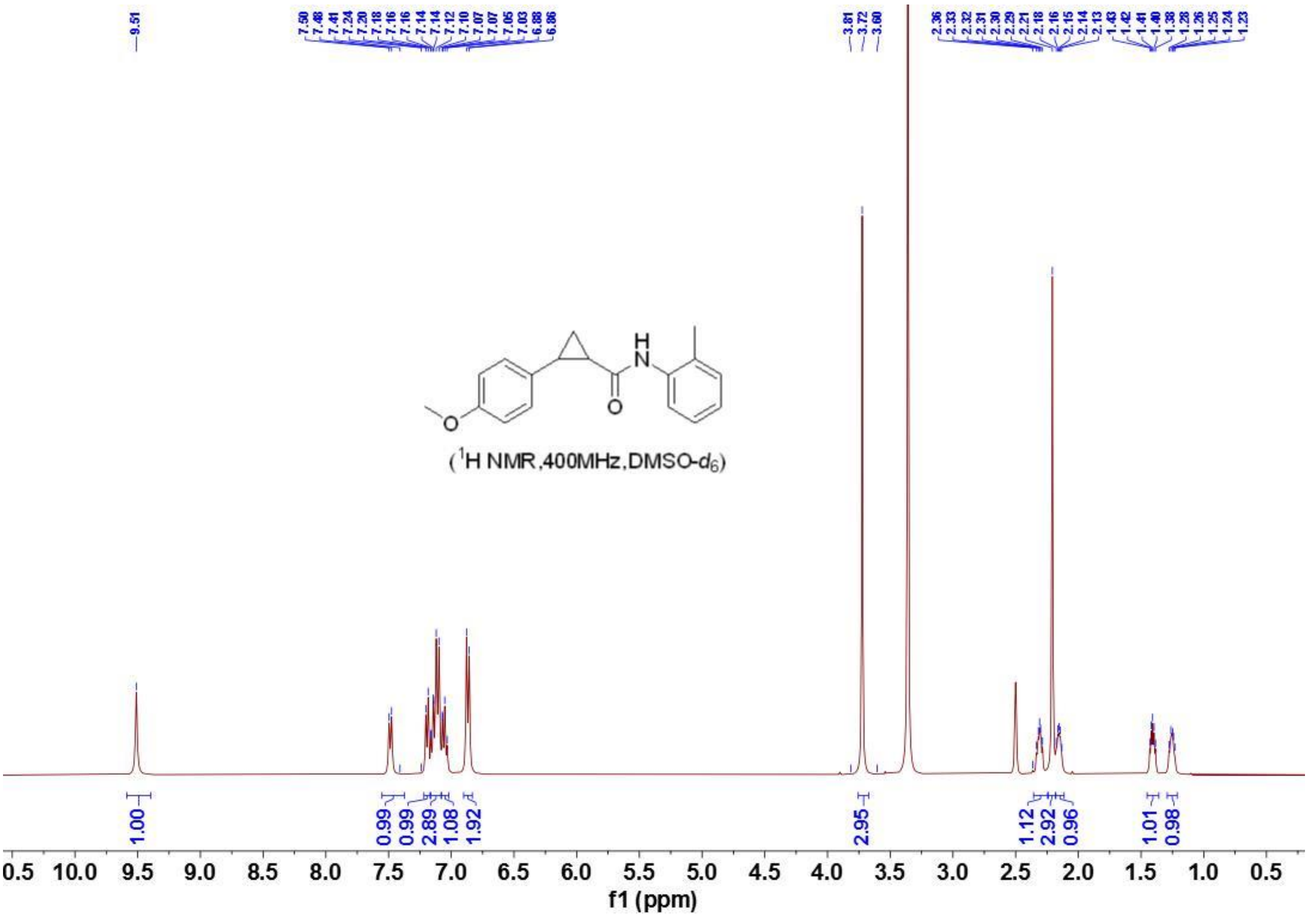

^{1}H NMR of compound **F24**

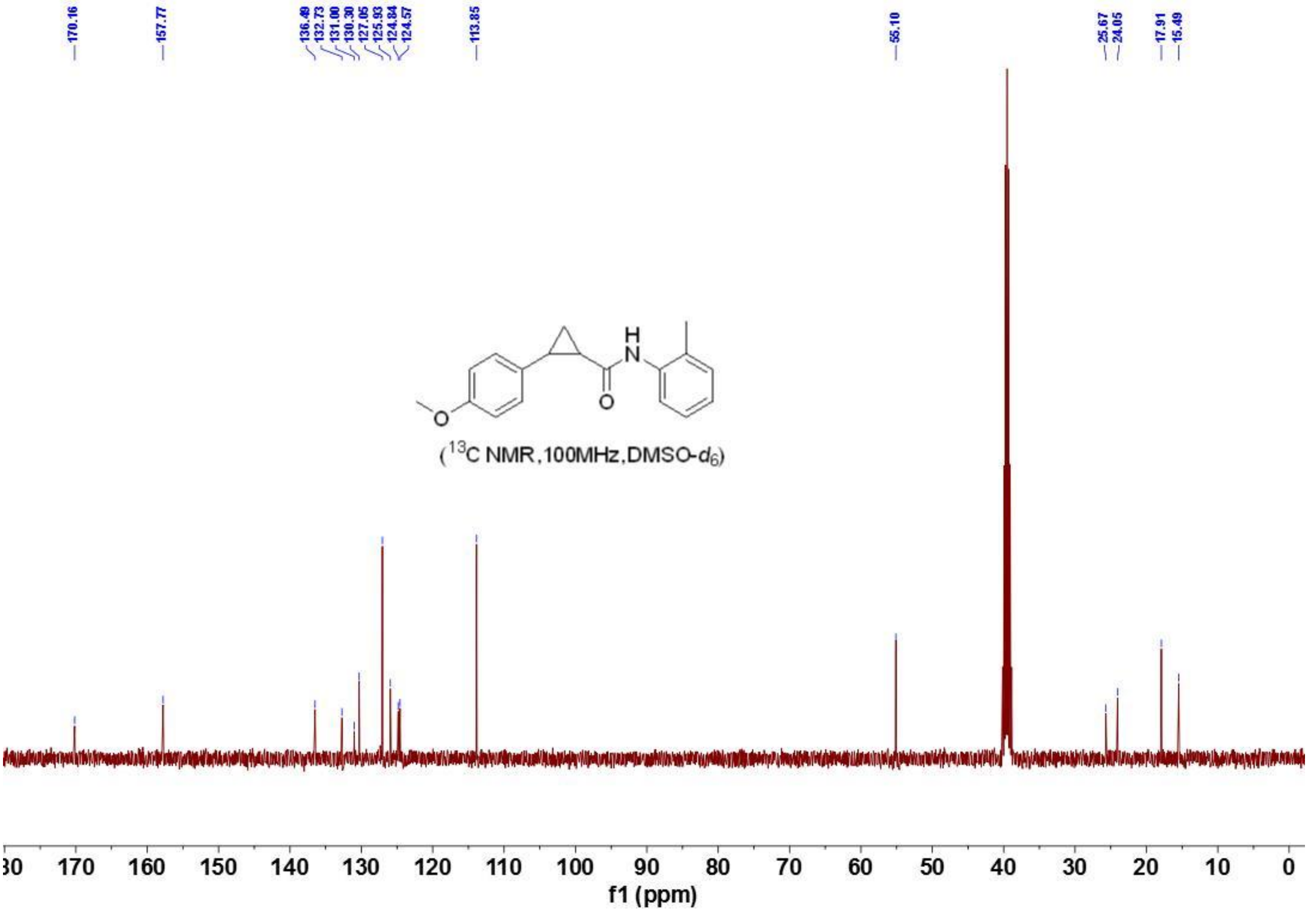

^{13}C NMR of compound **F24**

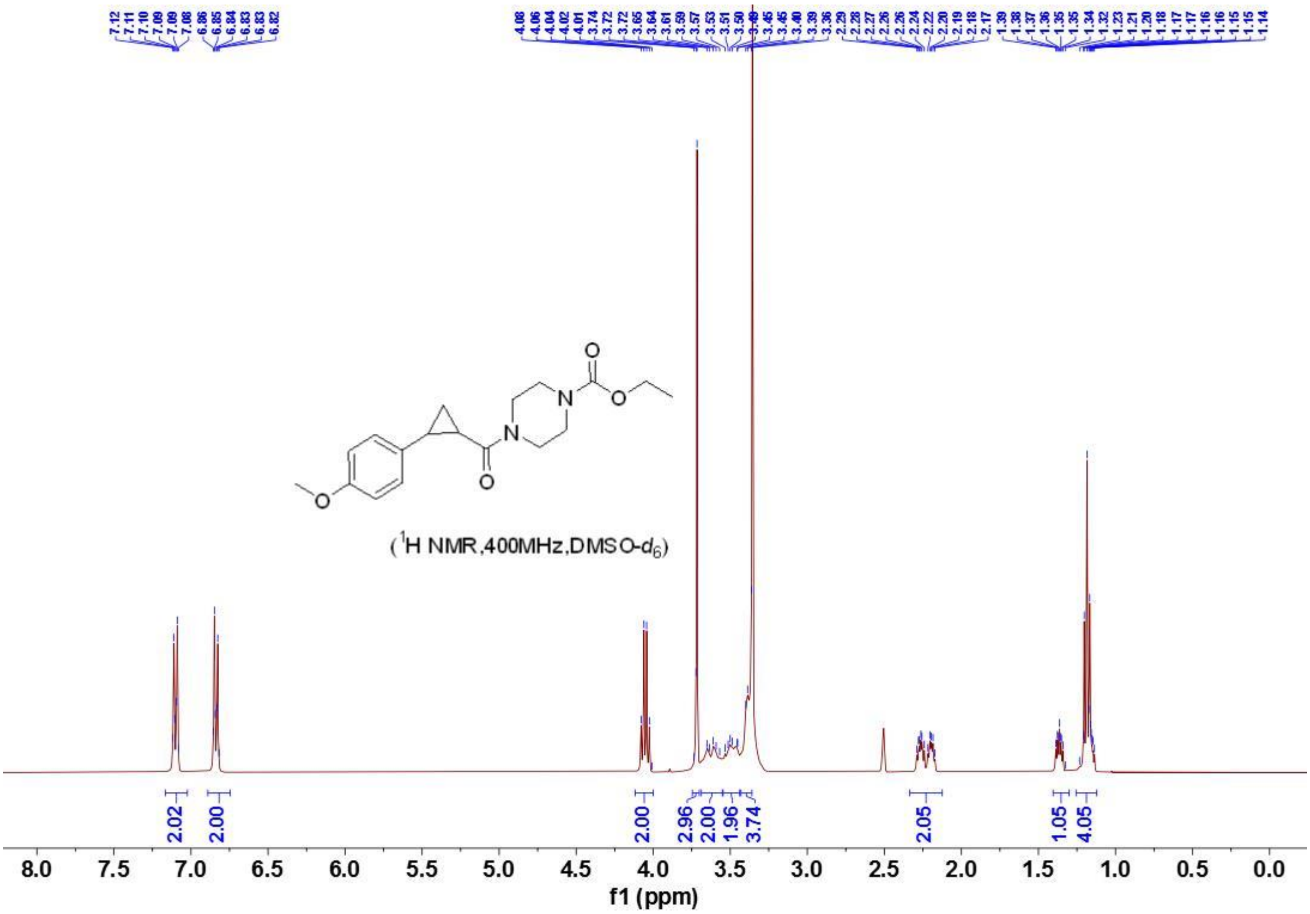

^{1}H NMR of compound **F25**

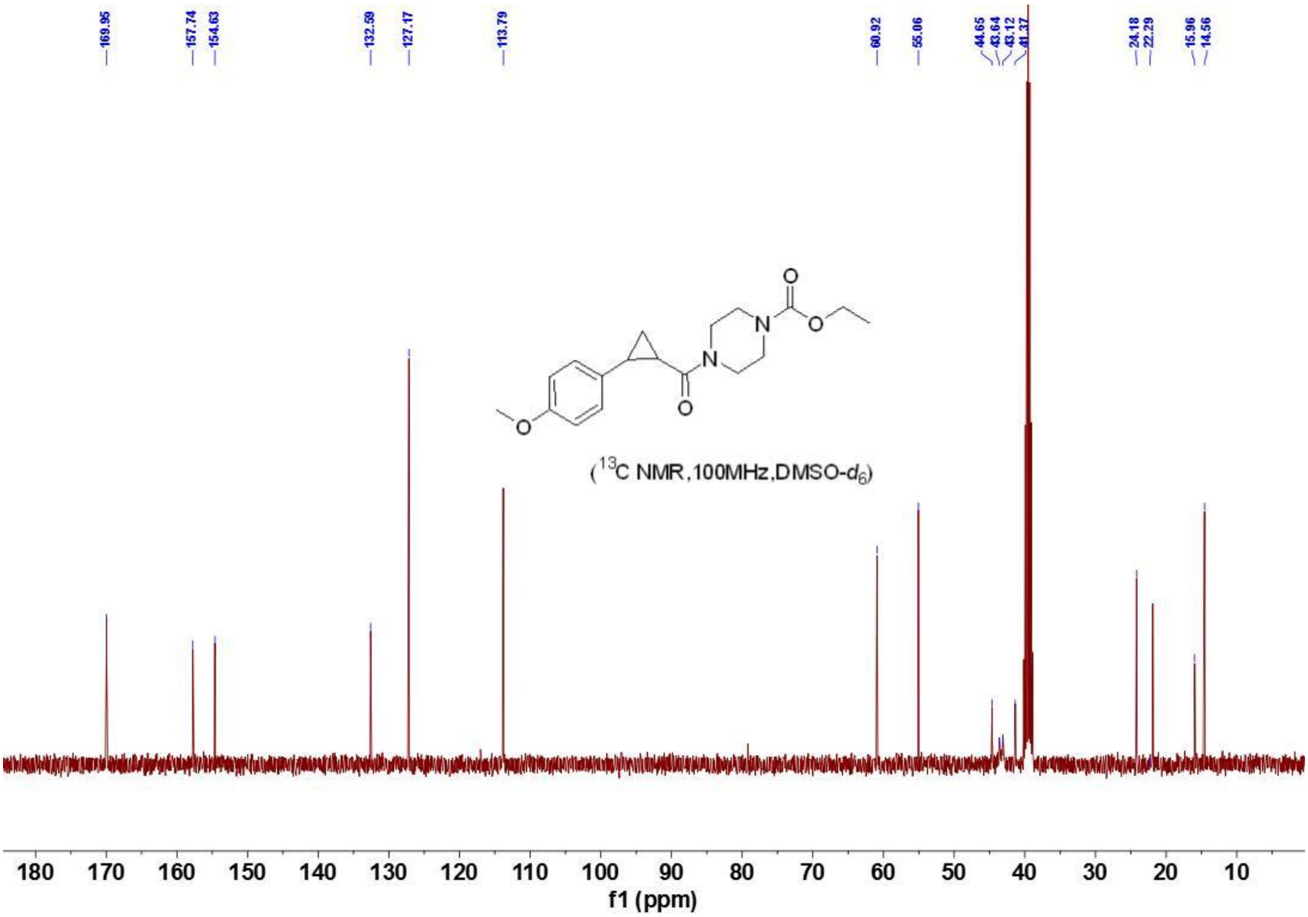

^{13}C NMR of compound **F25**

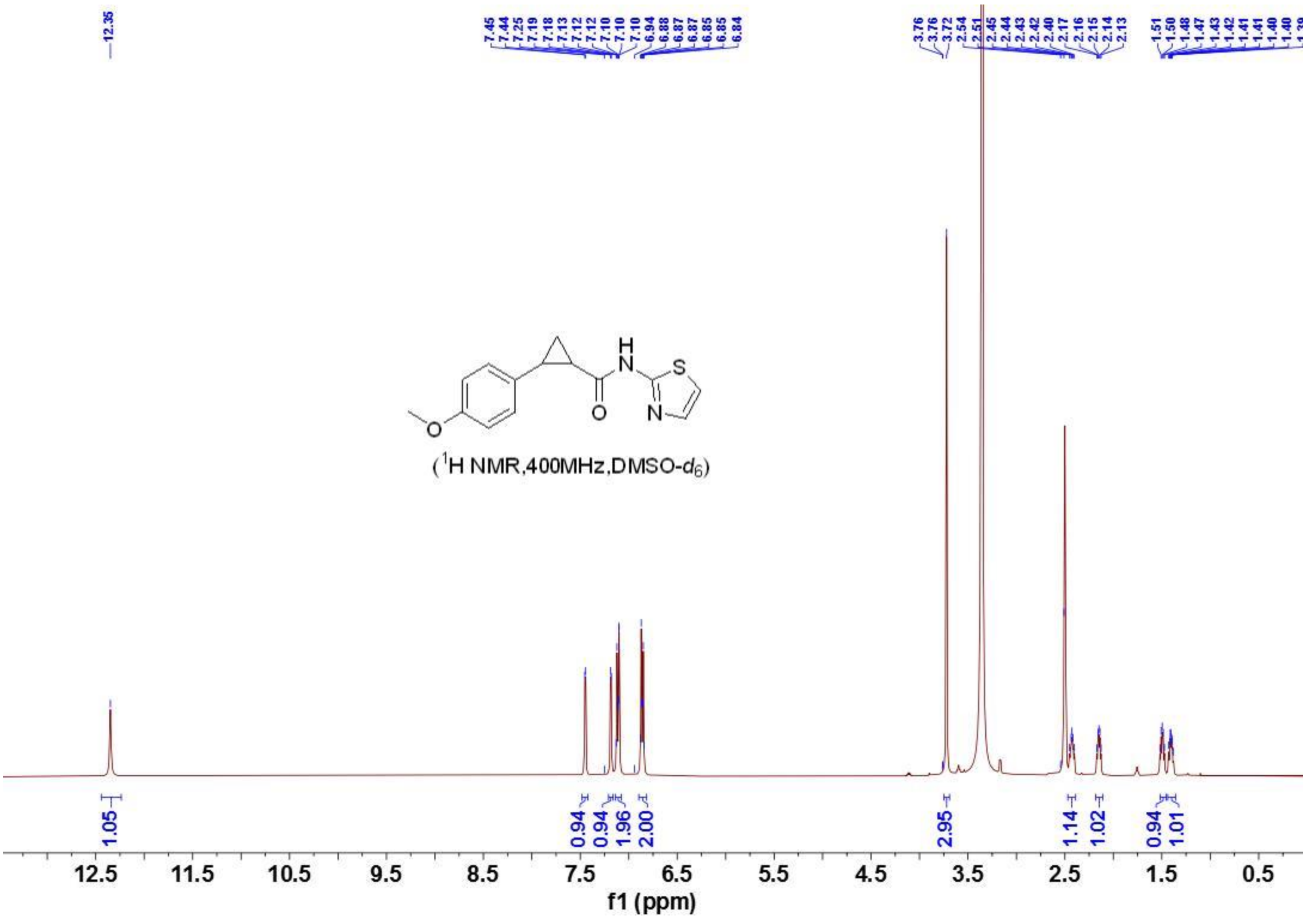

^{1}H NMR of compound **F26**

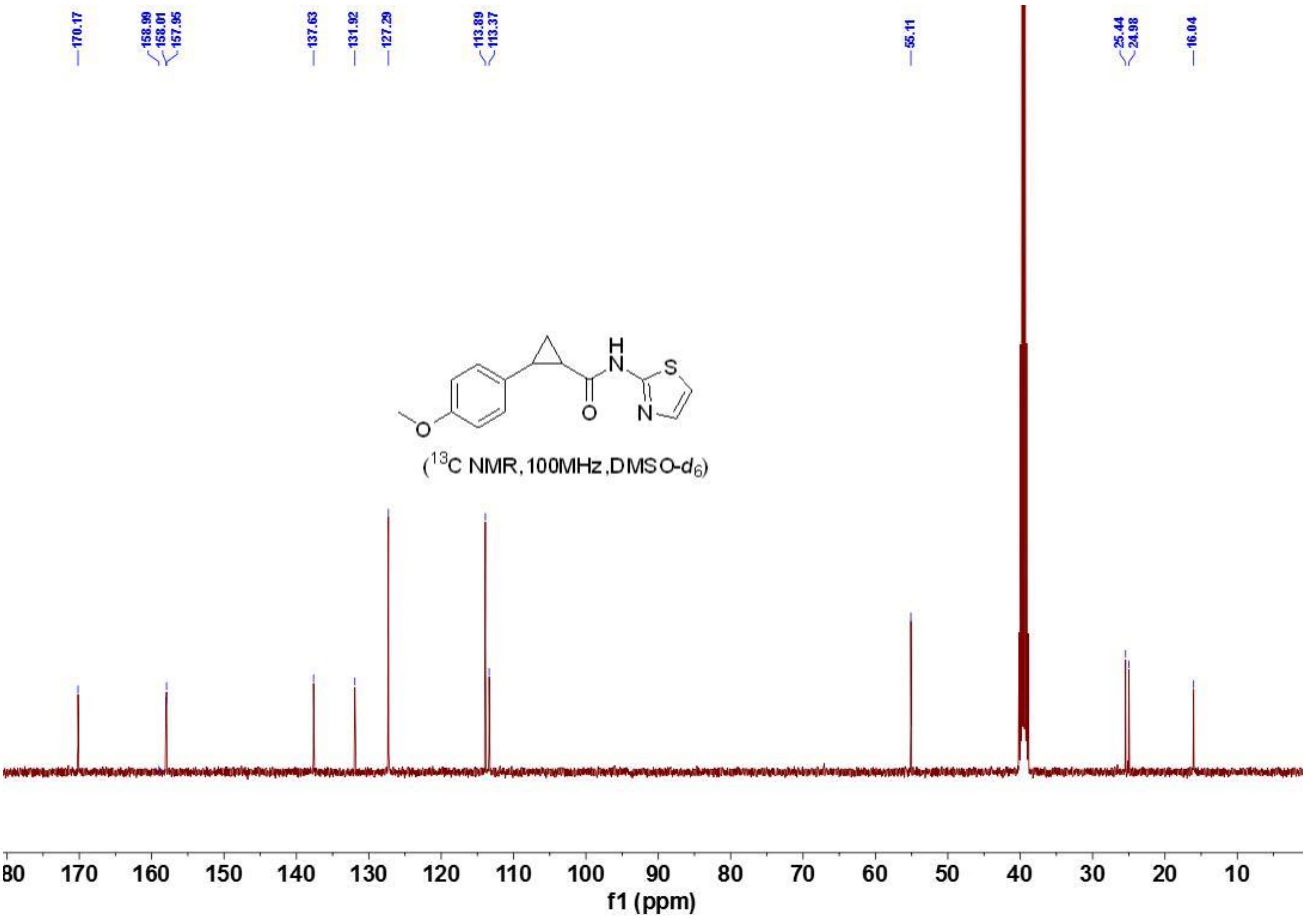

^{13}C NMR of compound **F26**

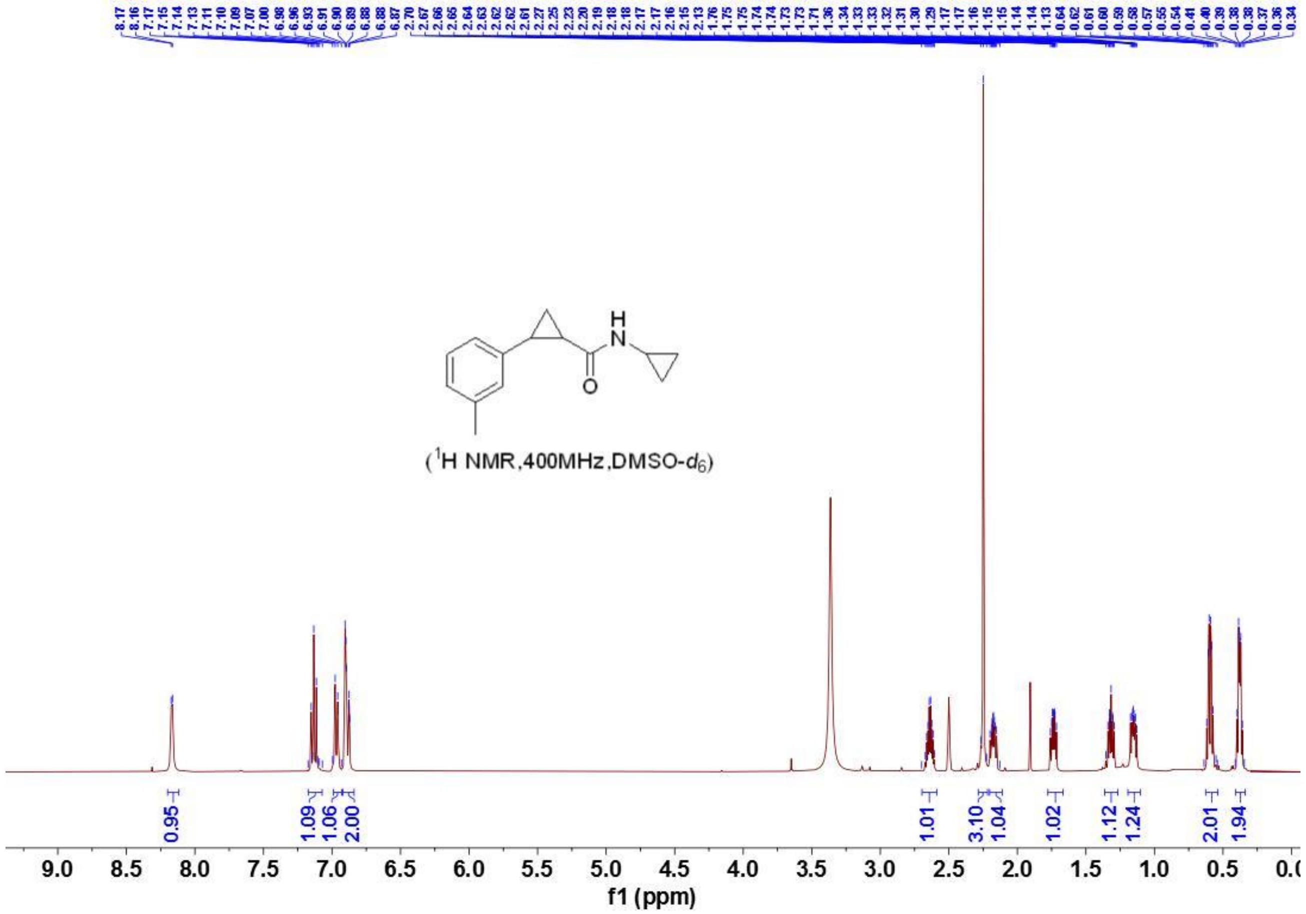

^{1}H NMR of compound **F27**

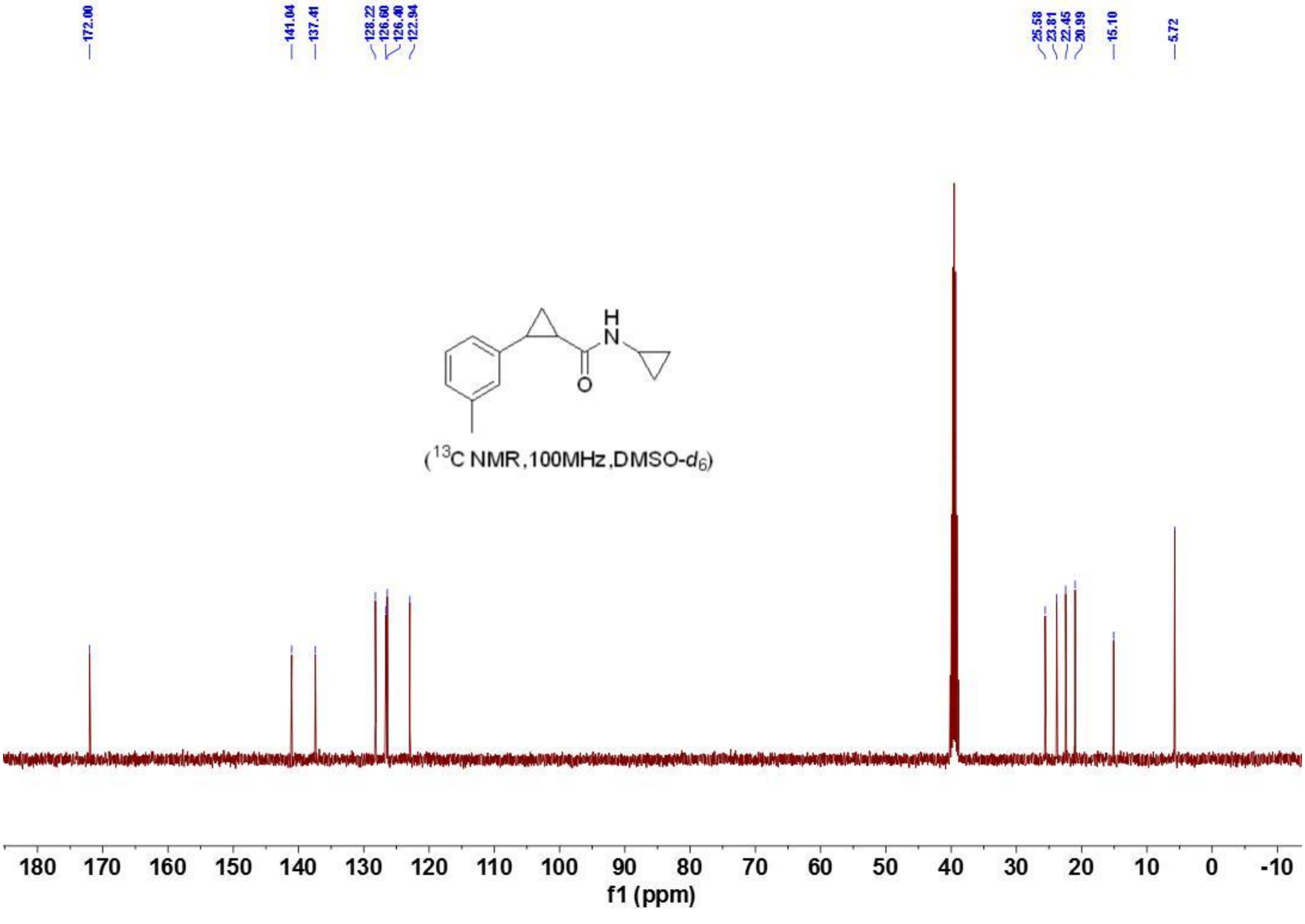

^{13}C NMR of compound **F27**

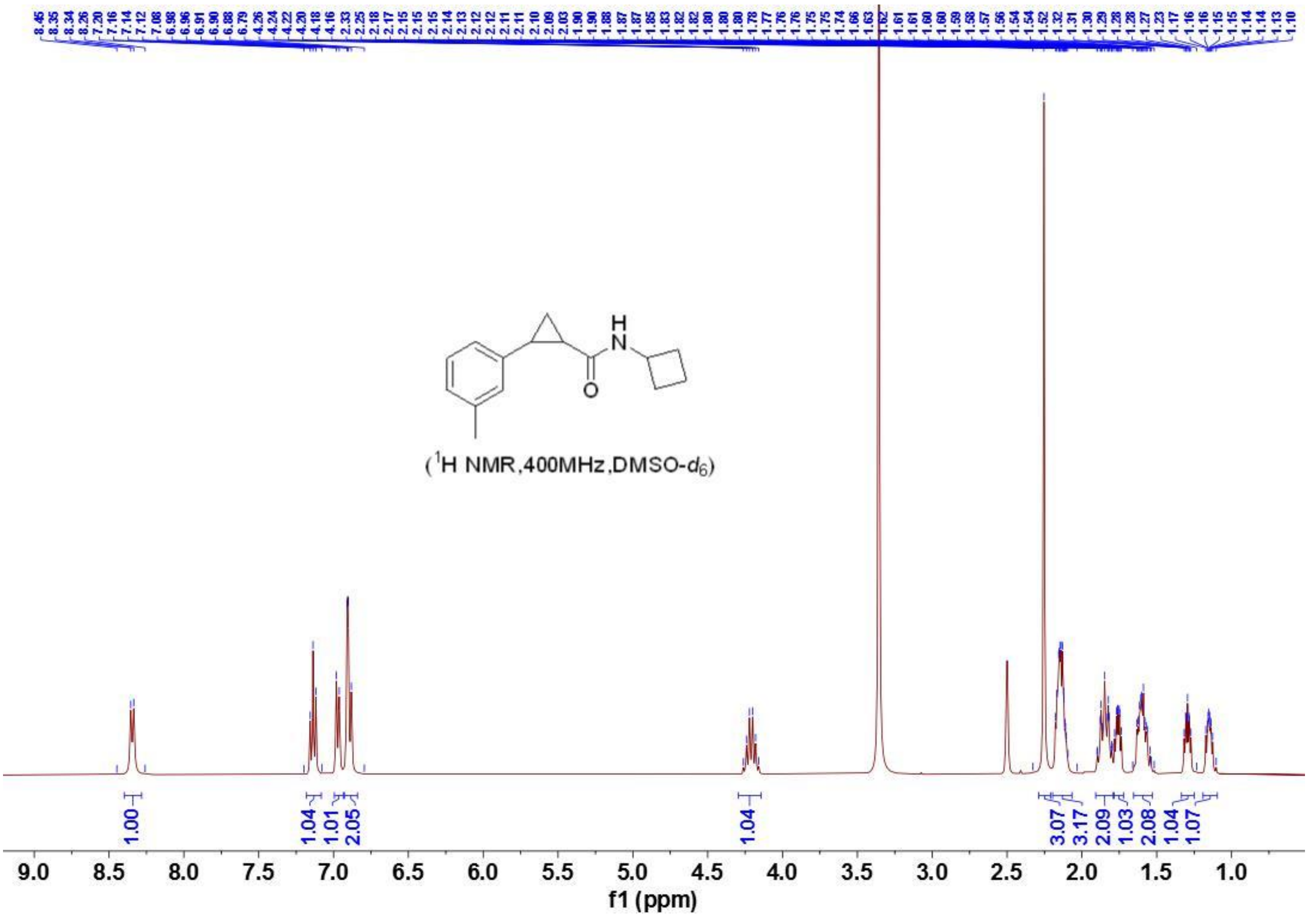

^{1}H NMR of compound **F28**

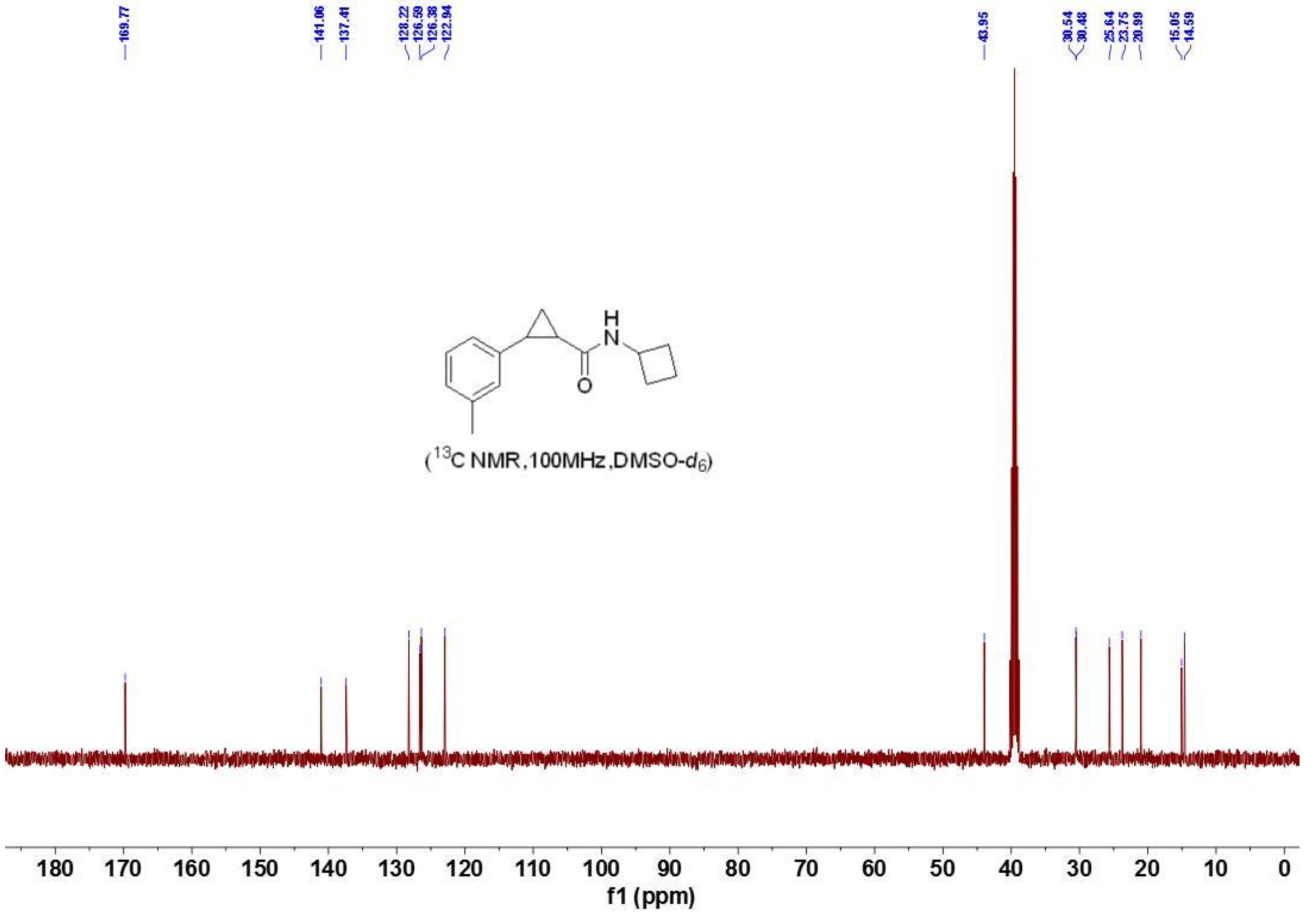

^{13}C NMR of compound **F28**

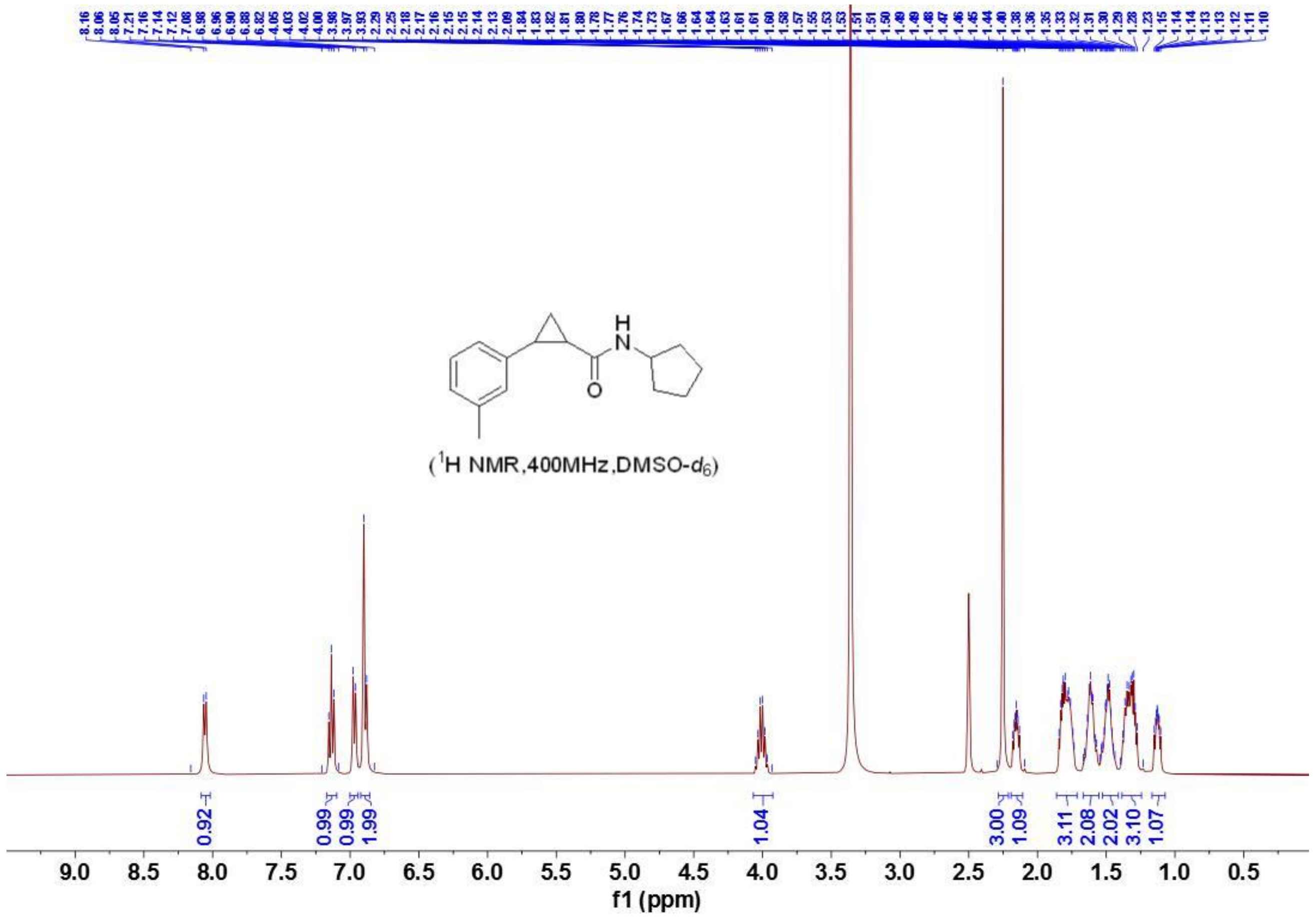

^{1}H NMR of compound **F29**

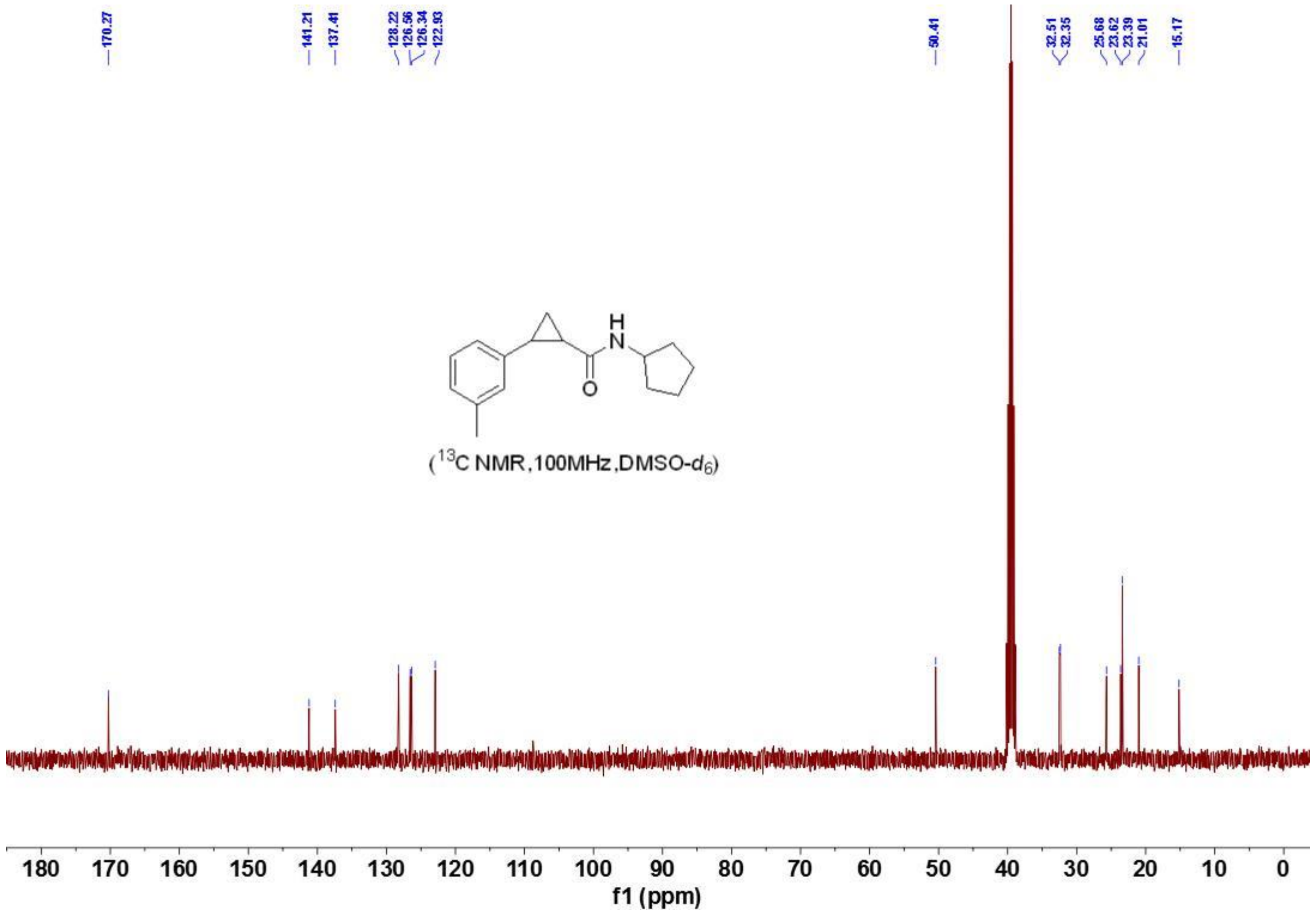

^{13}C NMR of compound **F29**

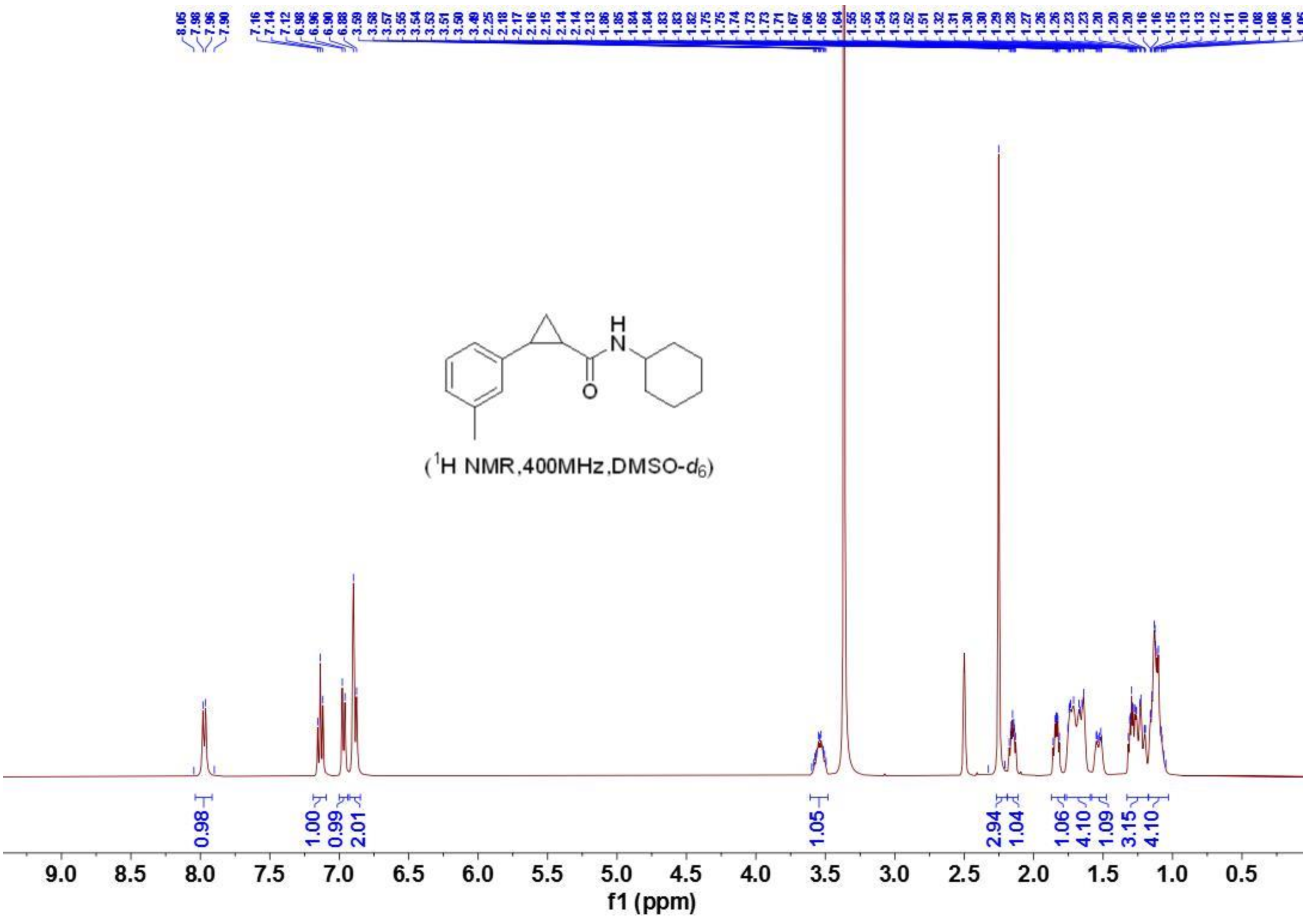

^{1}H NMR of compound **F30**

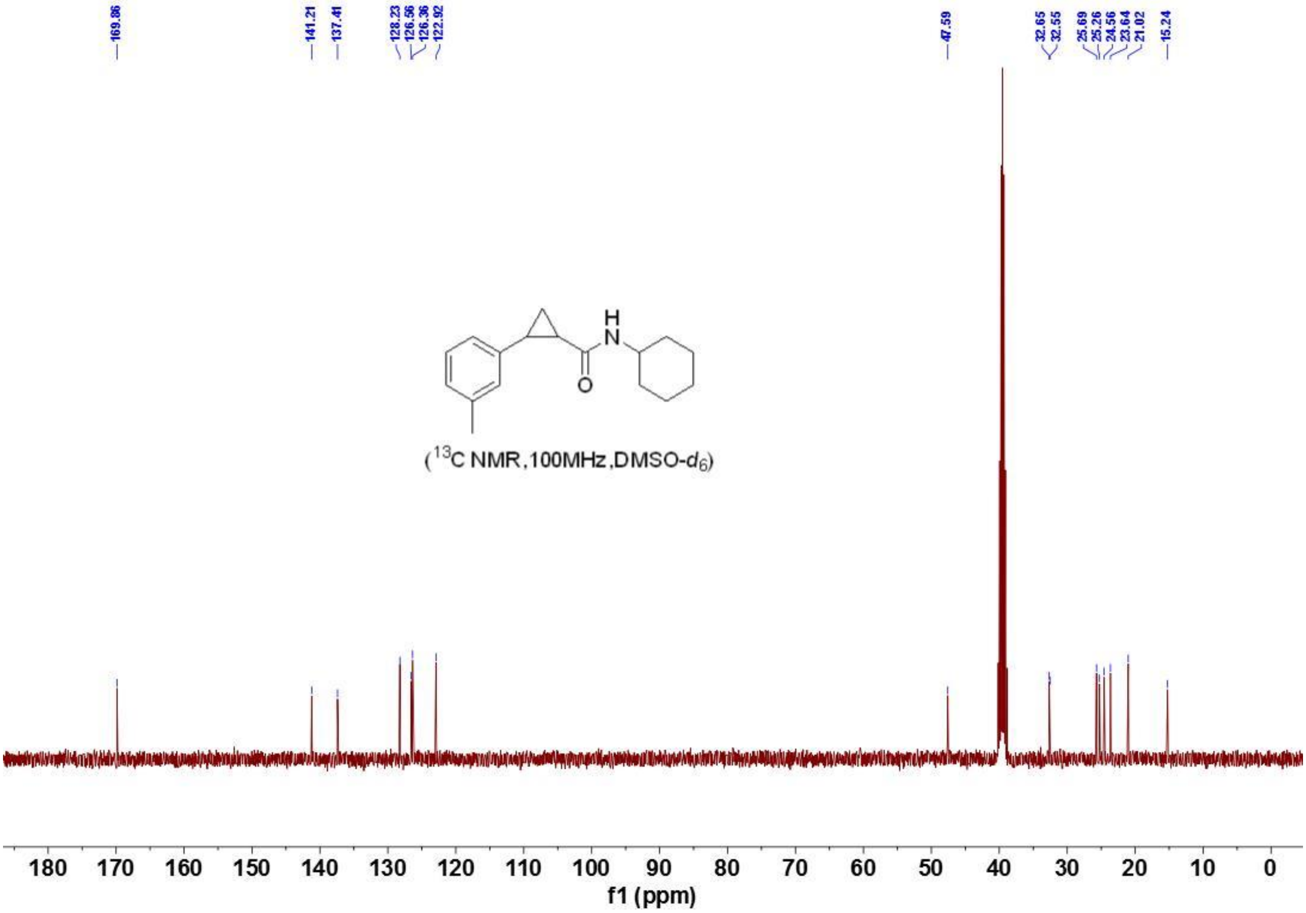

^{13}C NMR of compound **F30**

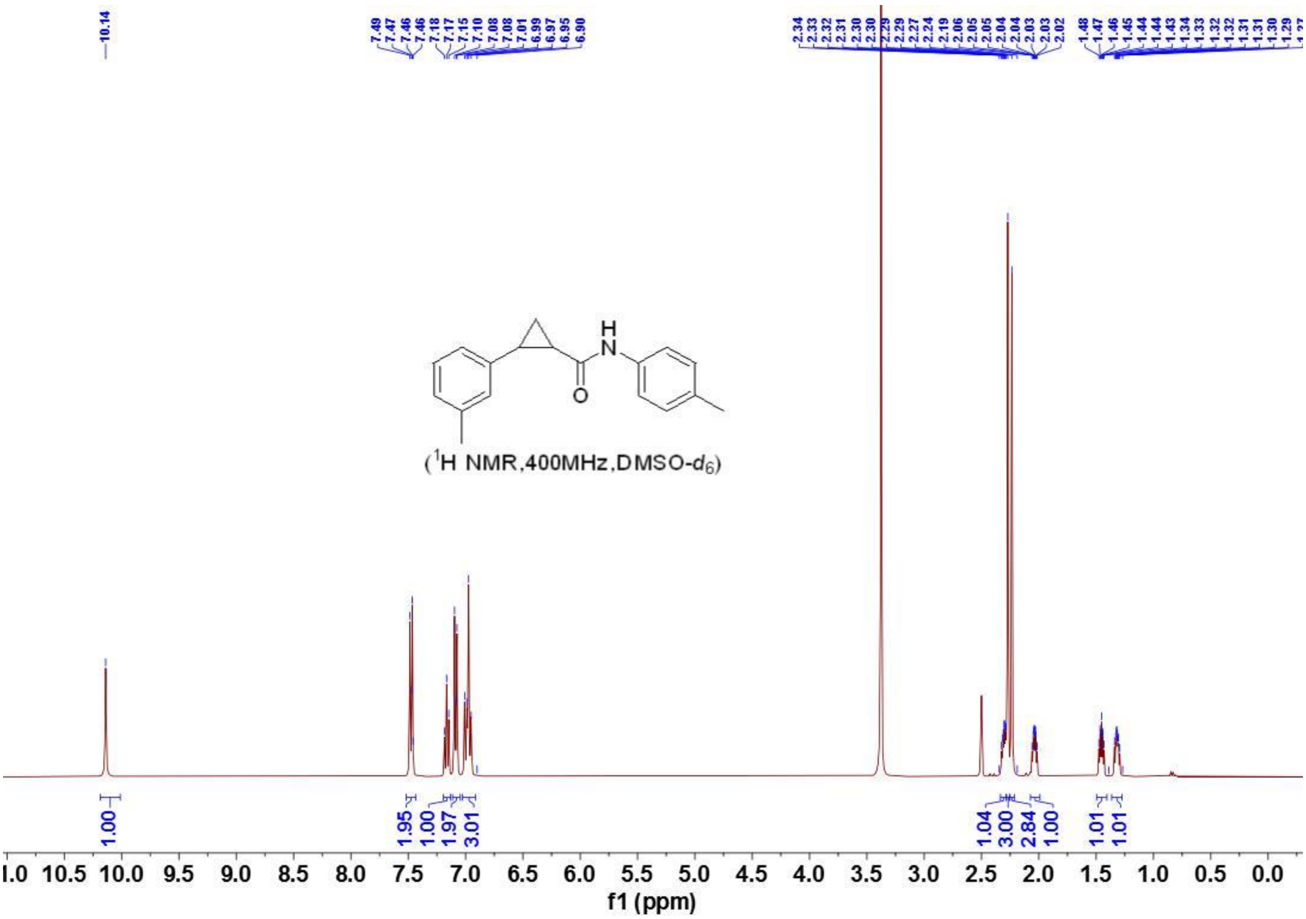

^{1}H NMR of compound **F31**

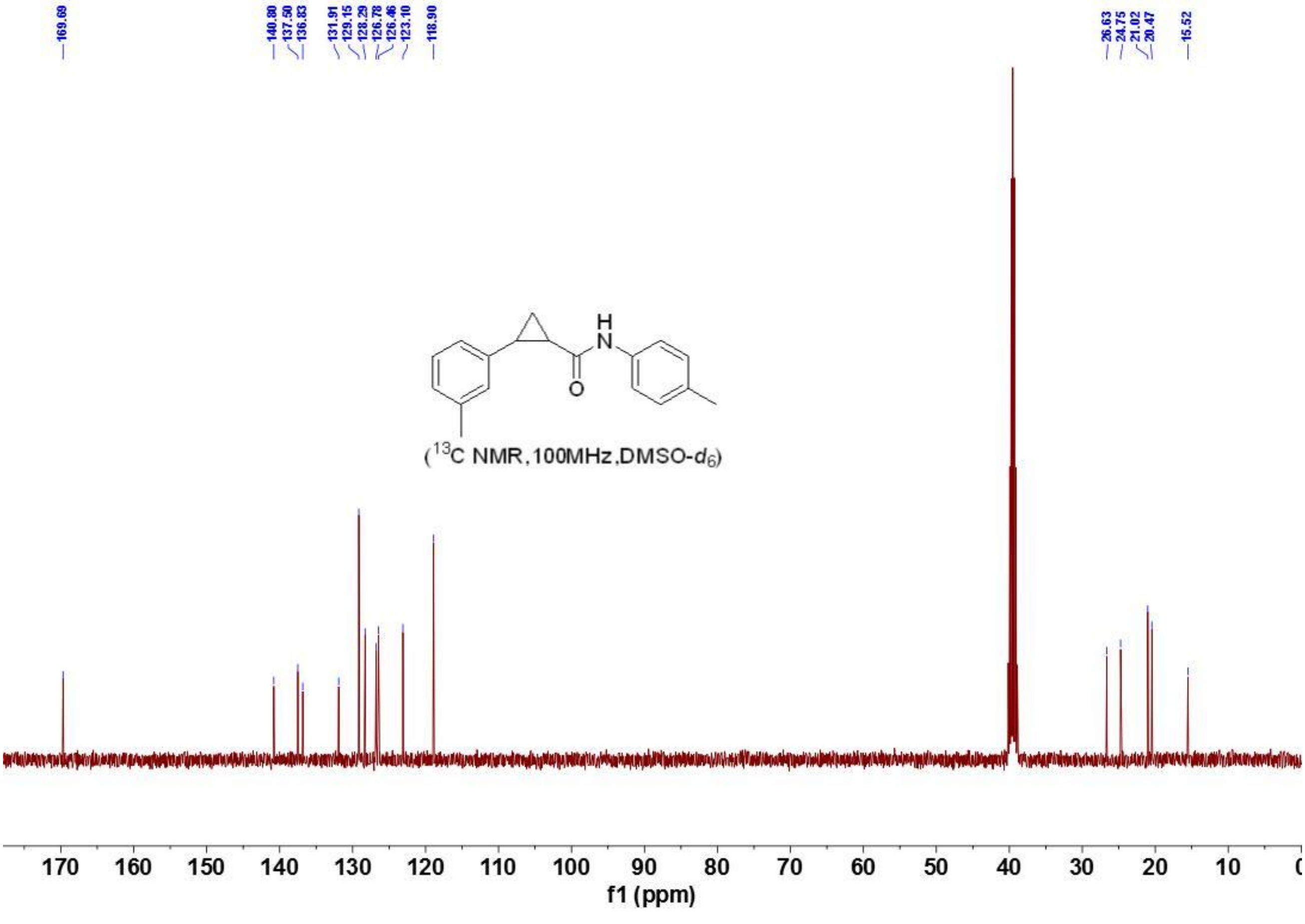

^{13}C NMR of compound **F31**

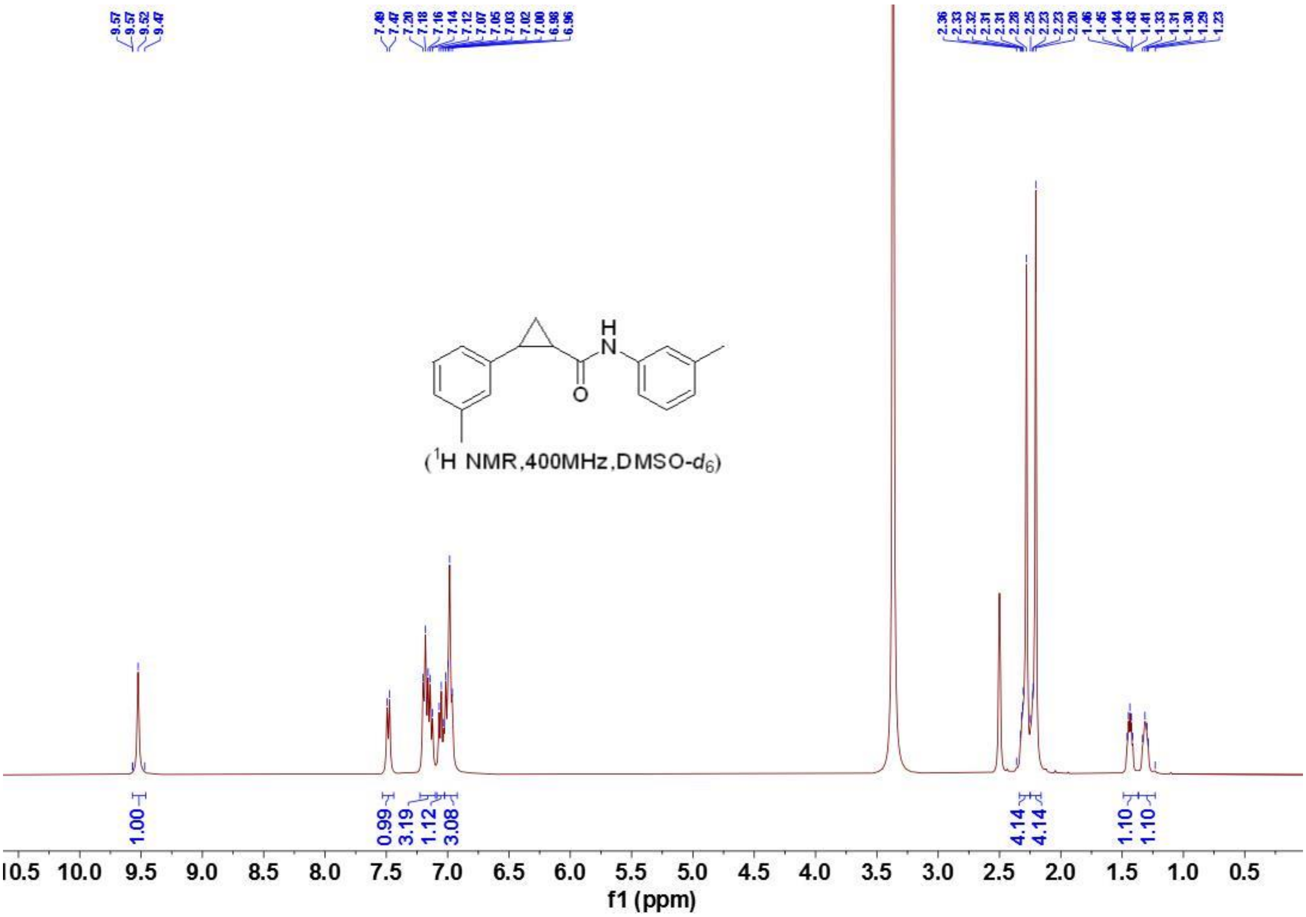

^{1}H NMR of compound **F32**

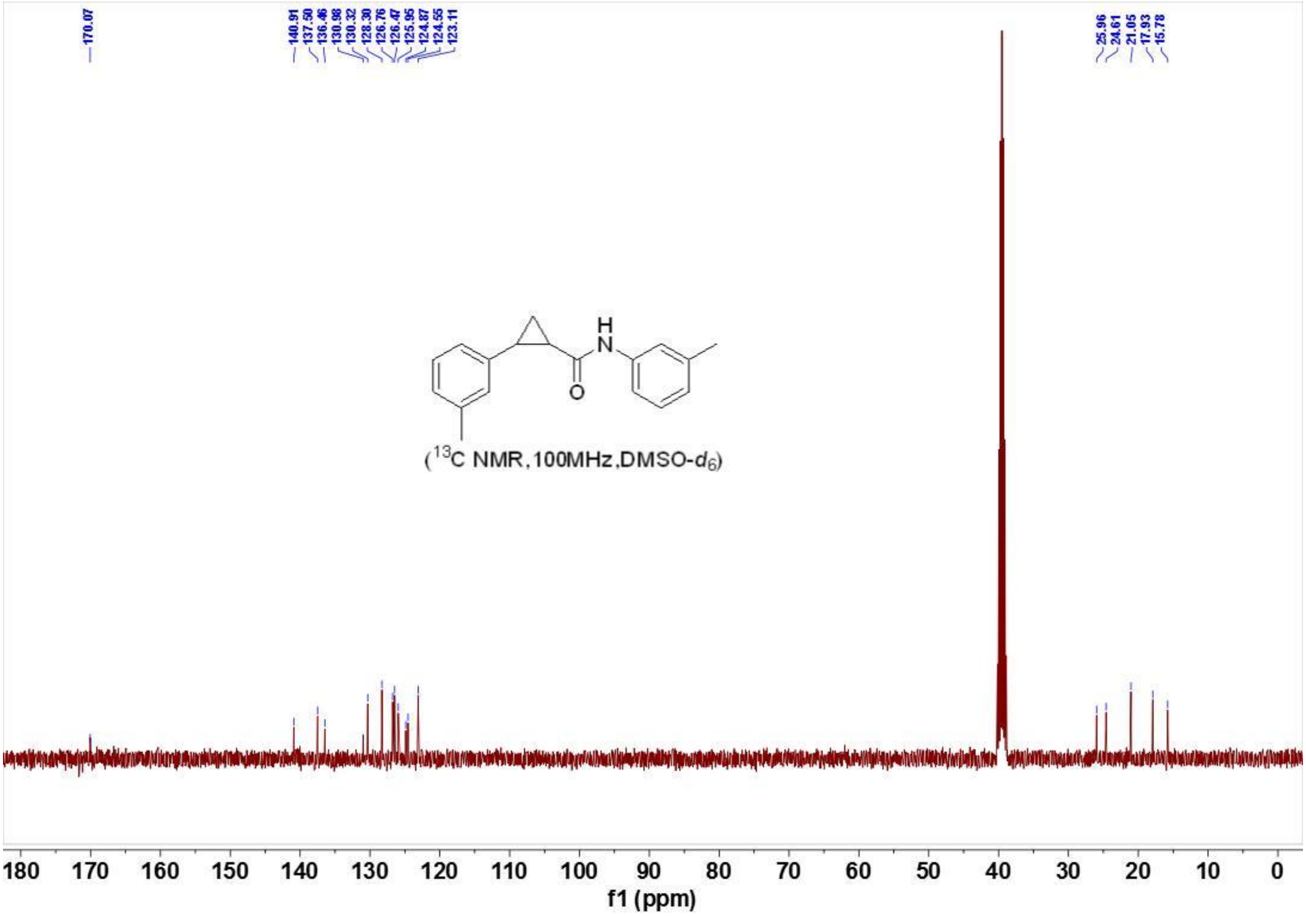

^{13}C NMR of compound **F32**

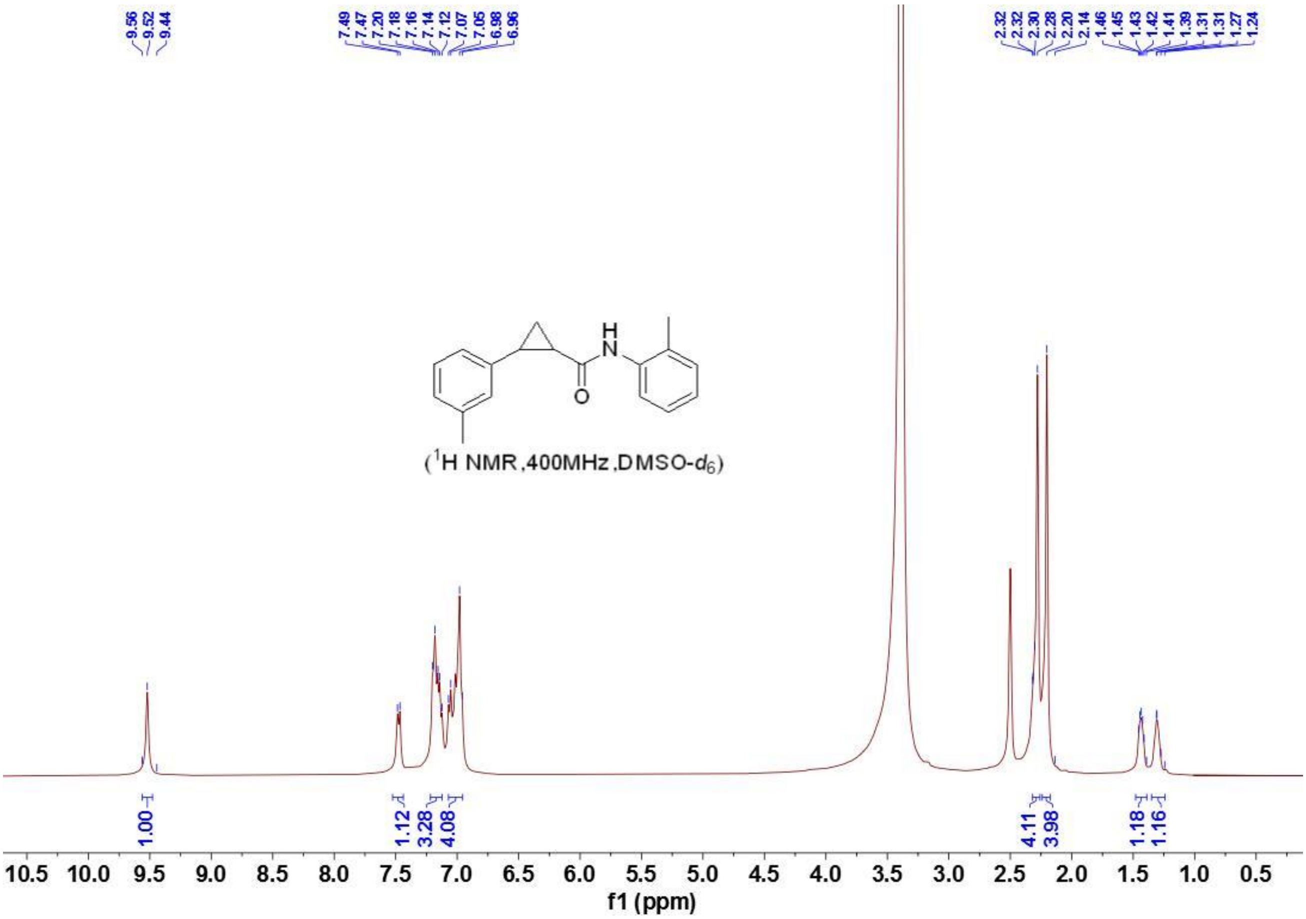

^{1}H NMR of compound **F33**

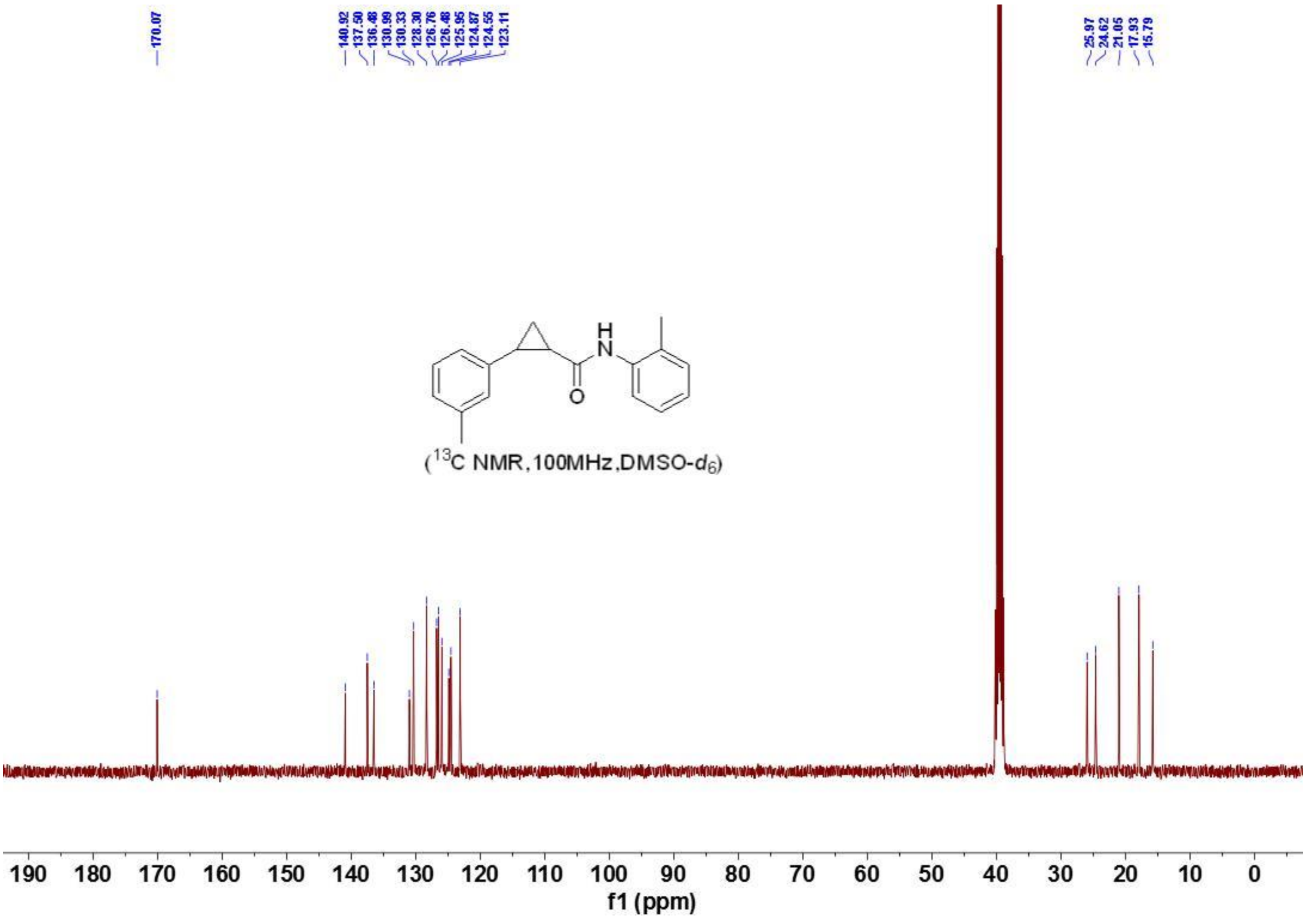

^{13}C NMR of compound **F33**

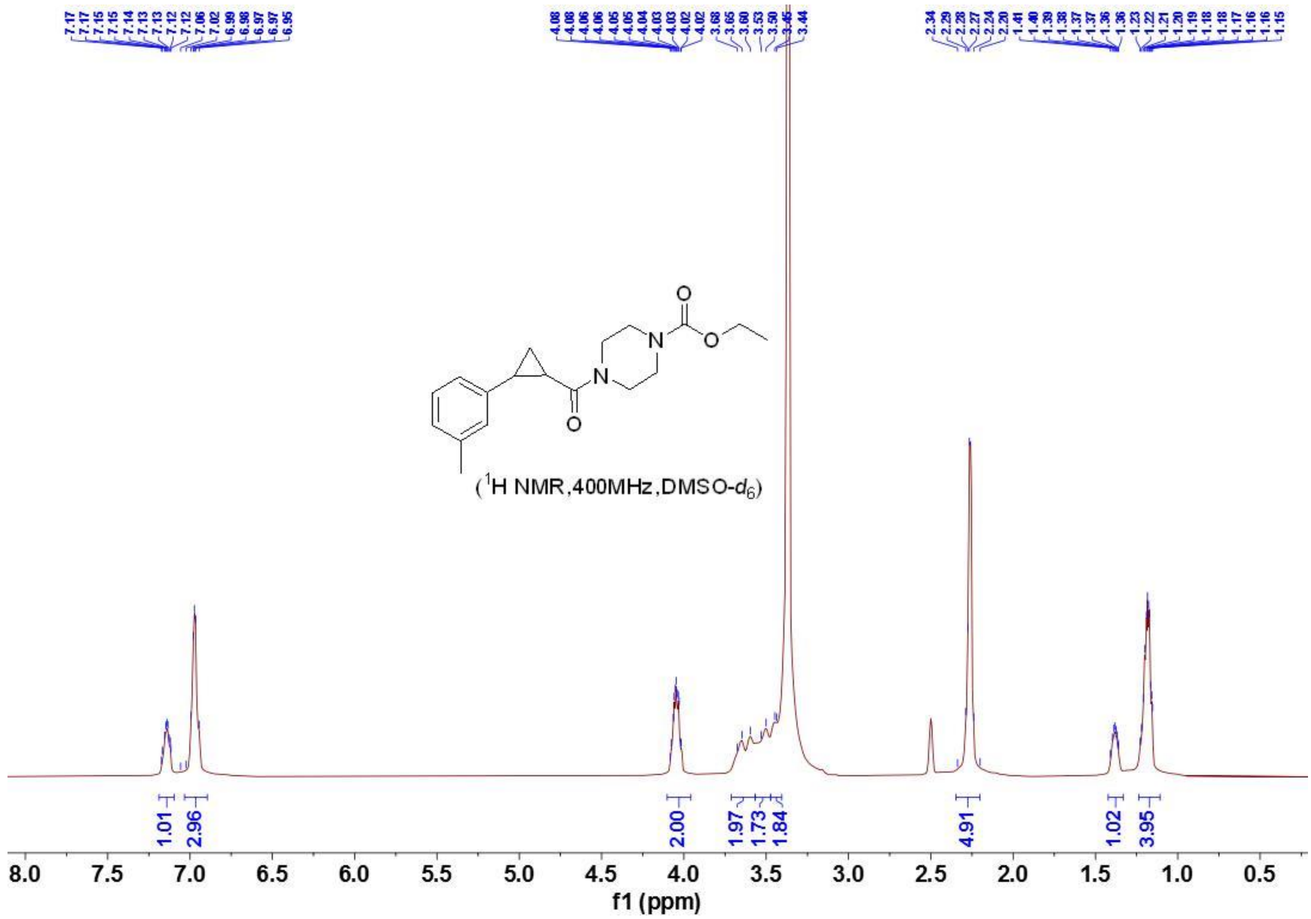

^1H NMR of compound **F34**

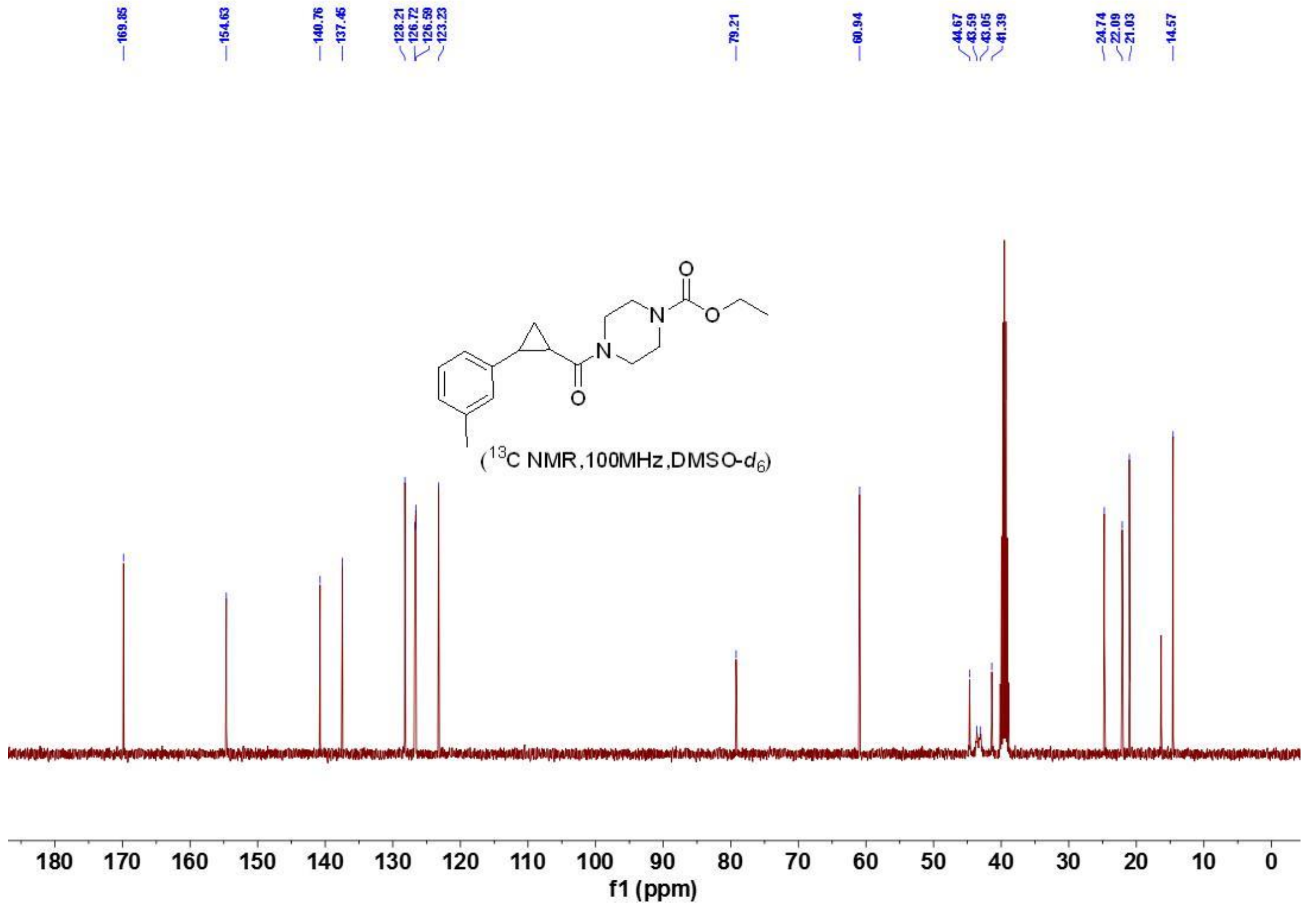

^{13}C NMR of compound **F34**

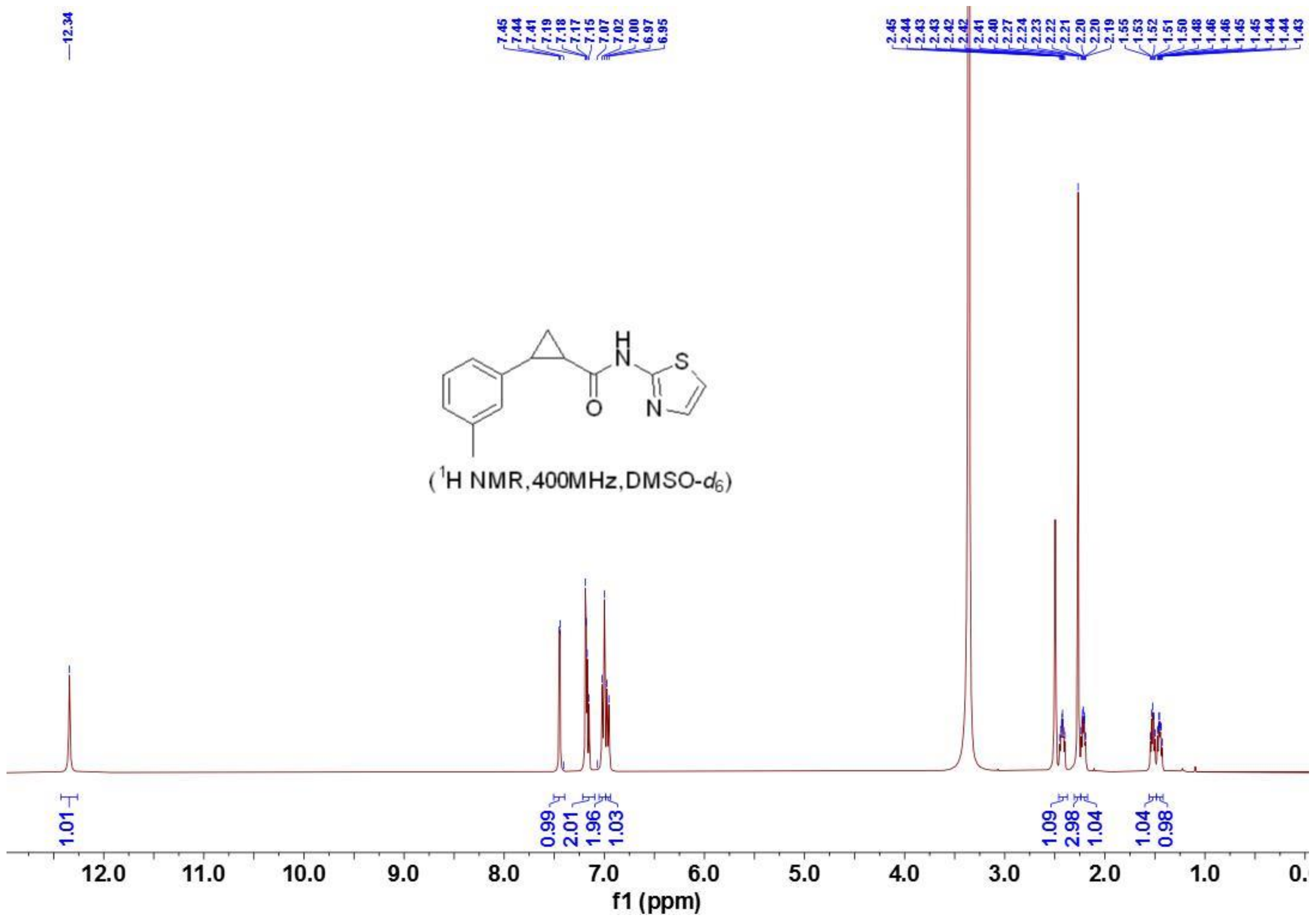

^{1}H NMR of compound **F35**

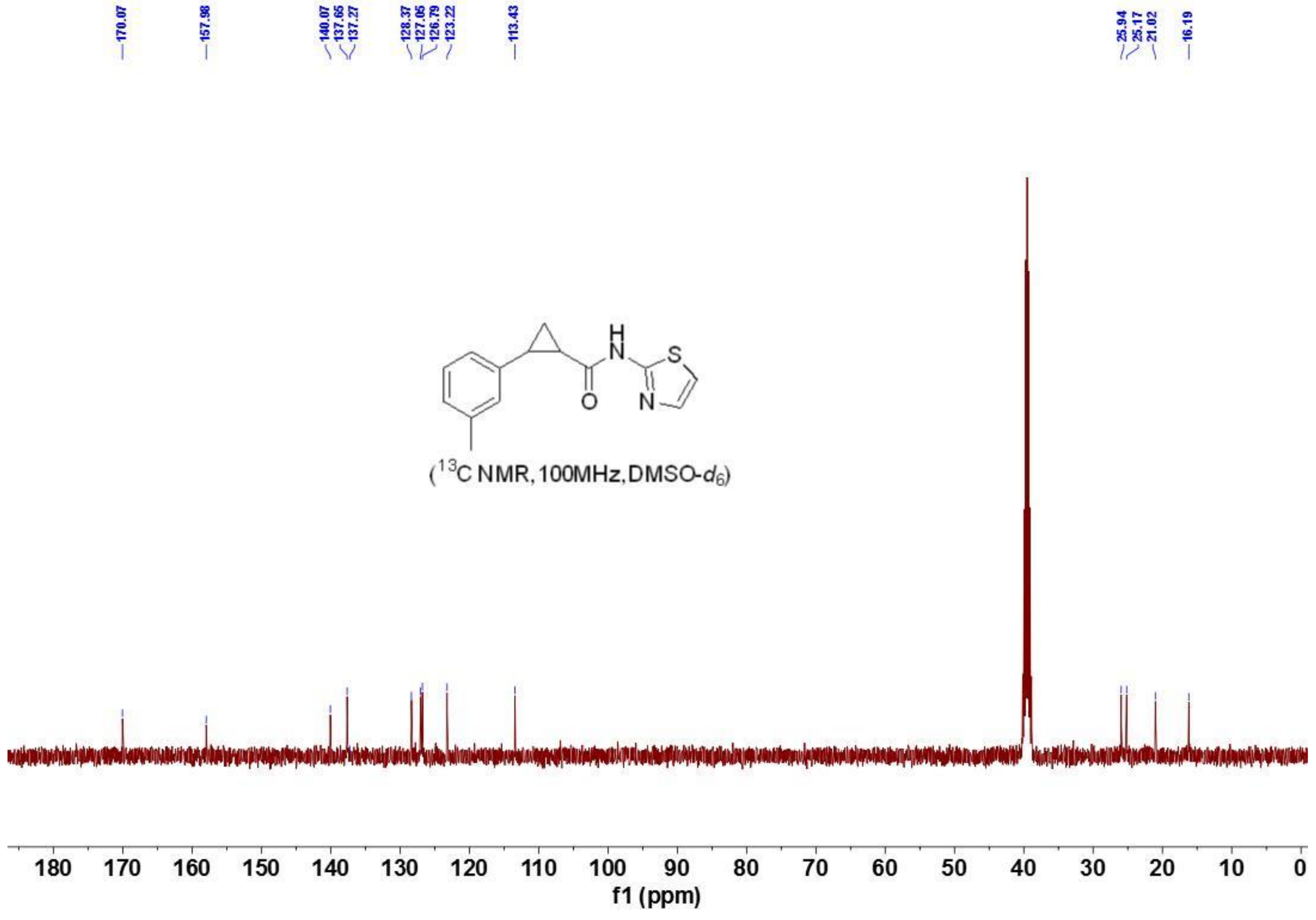

^{13}C NMR of compound **F35**

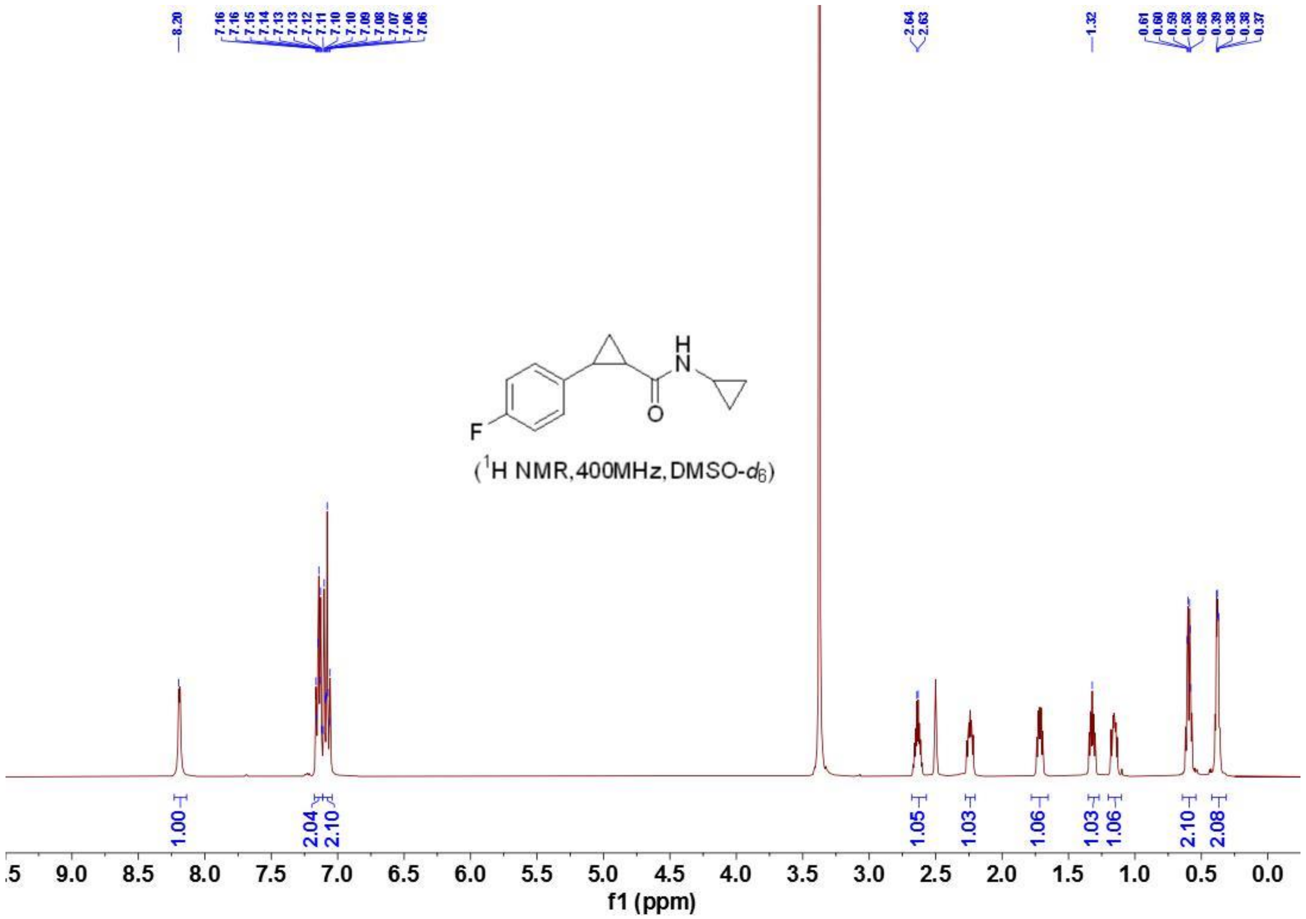

^{1}H NMR of compound **F36**

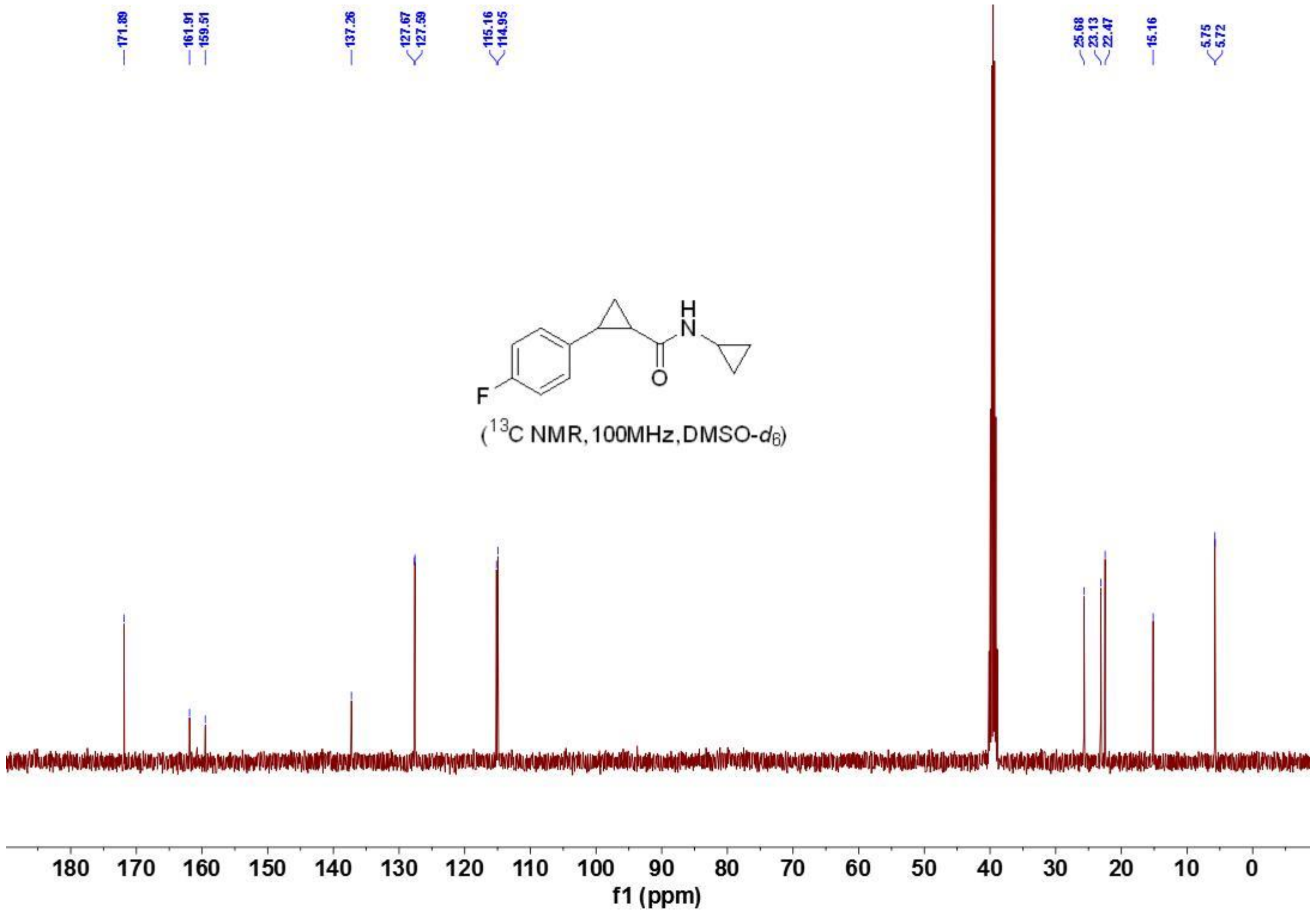

^{13}C NMR of compound **F36**

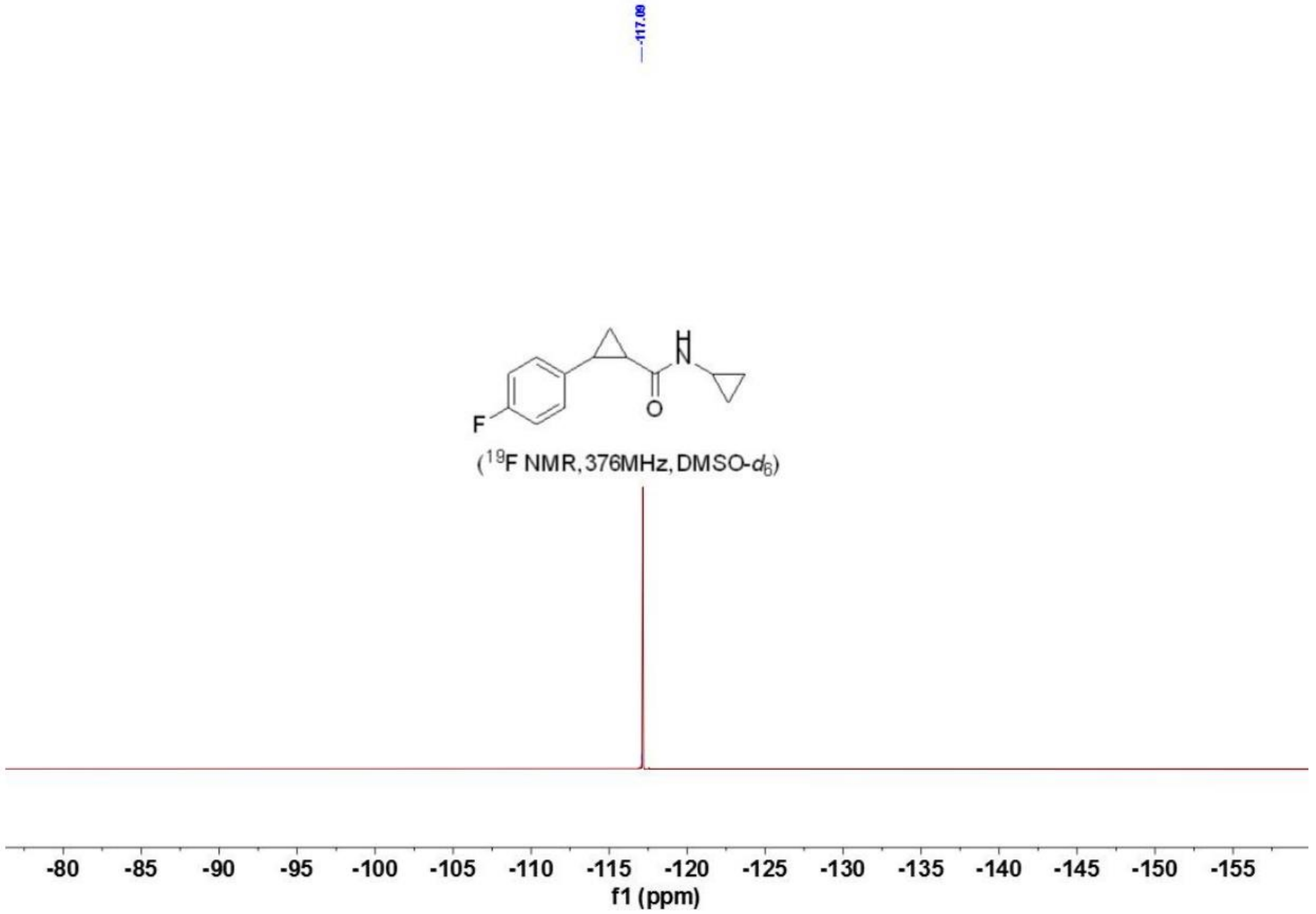

^{19}F NMR of compound **F36**

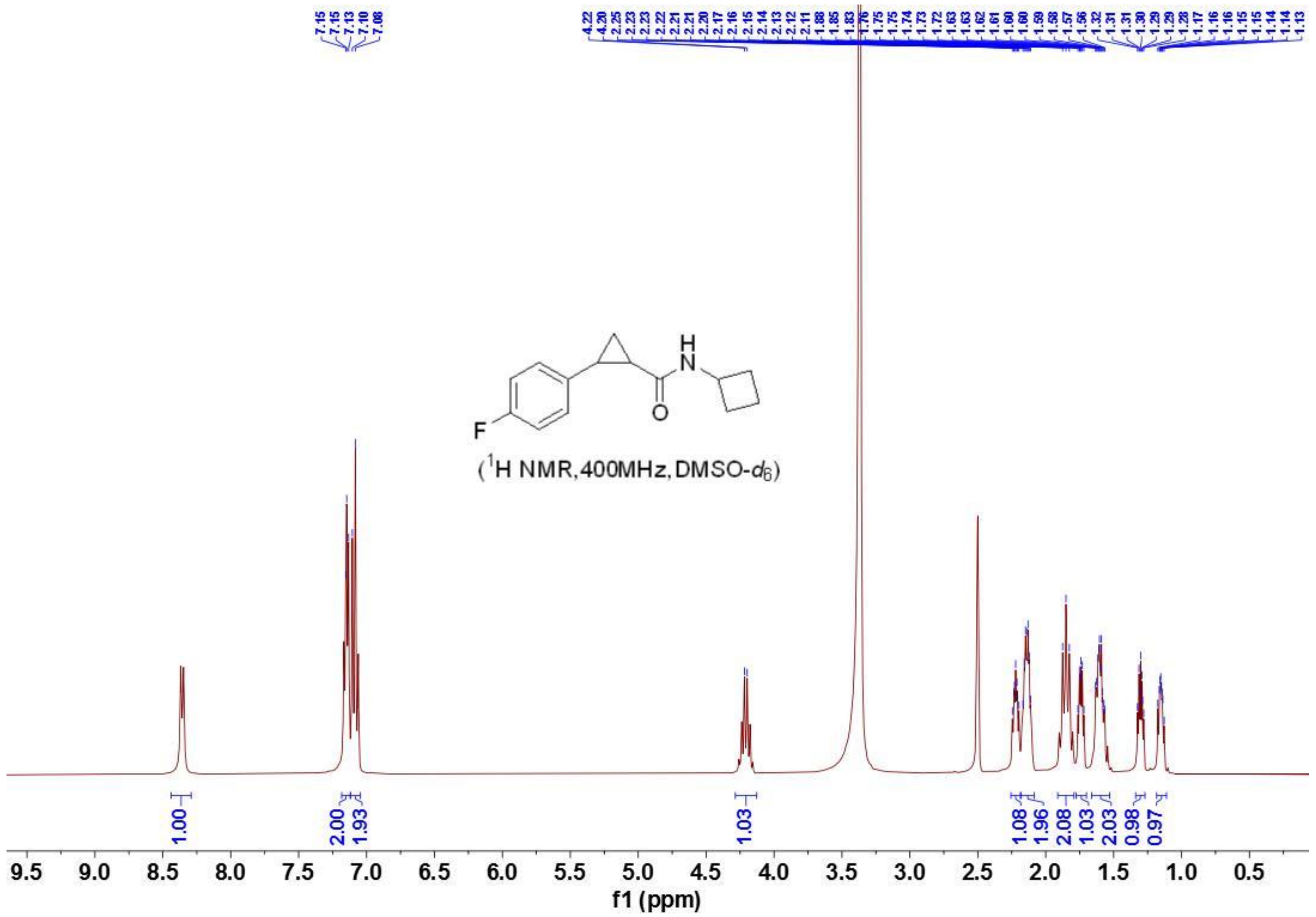

^{1}H NMR of compound **F37**

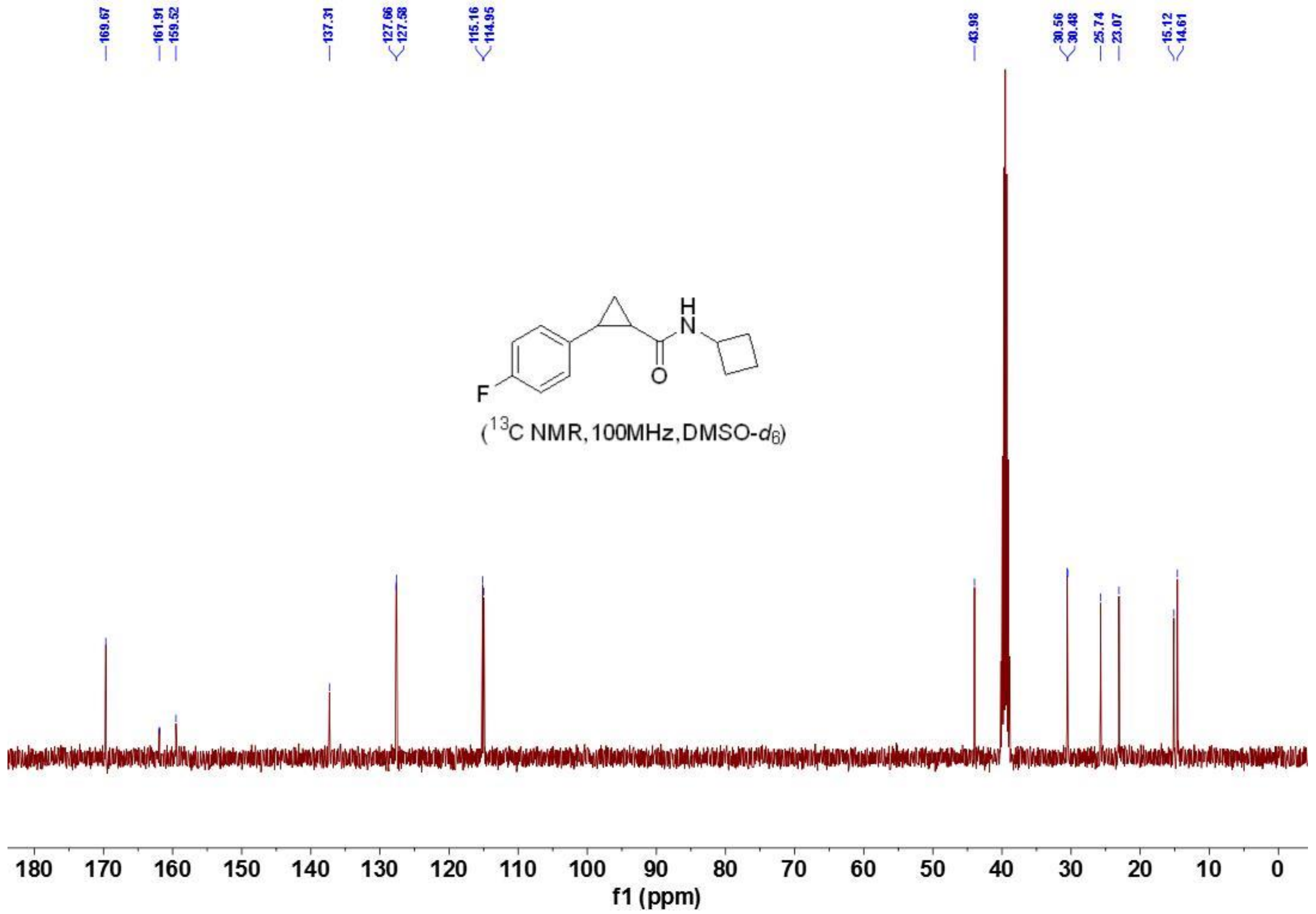

^{13}C NMR of compound **F37**

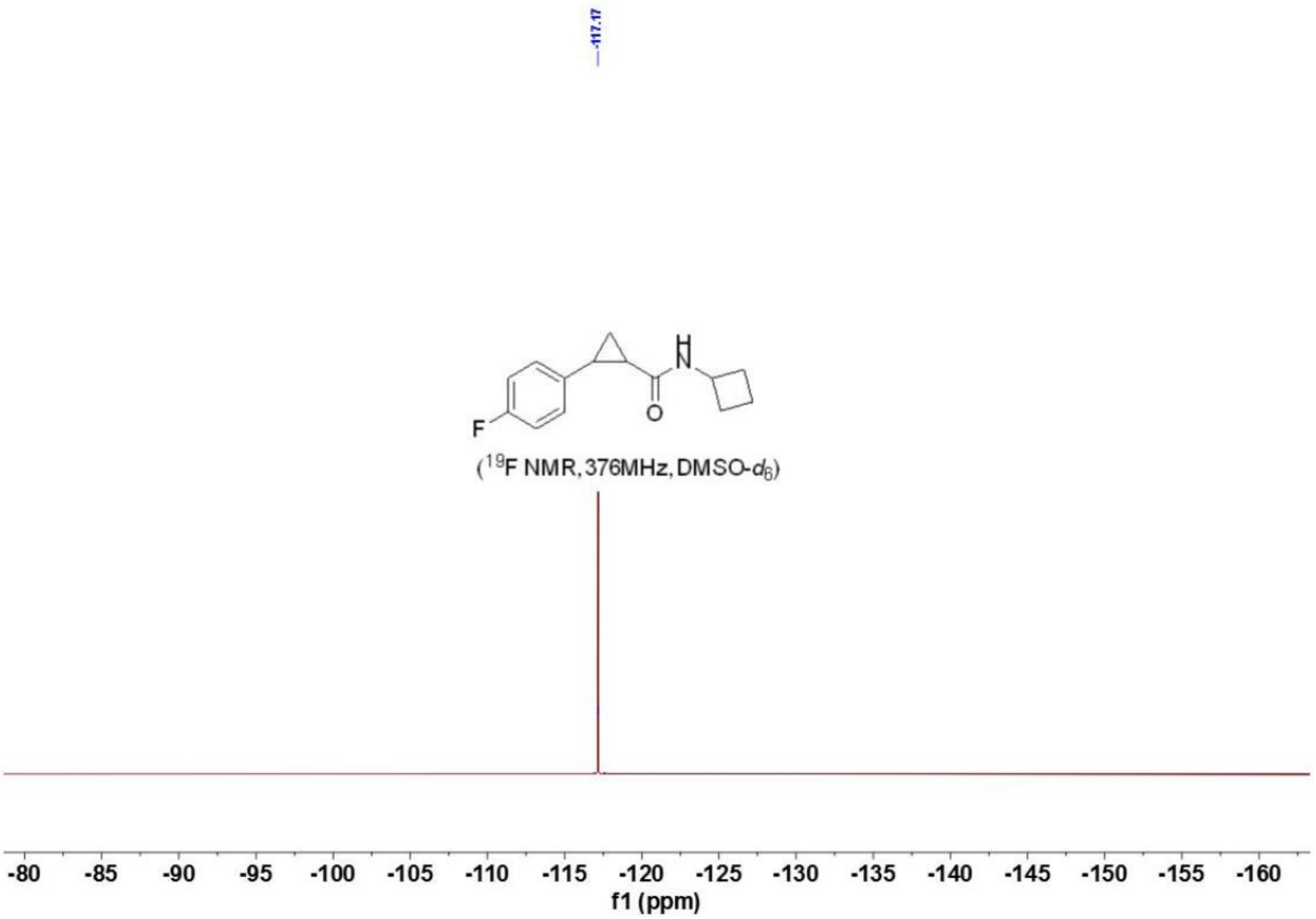

^{19}F NMR of compound **F37**

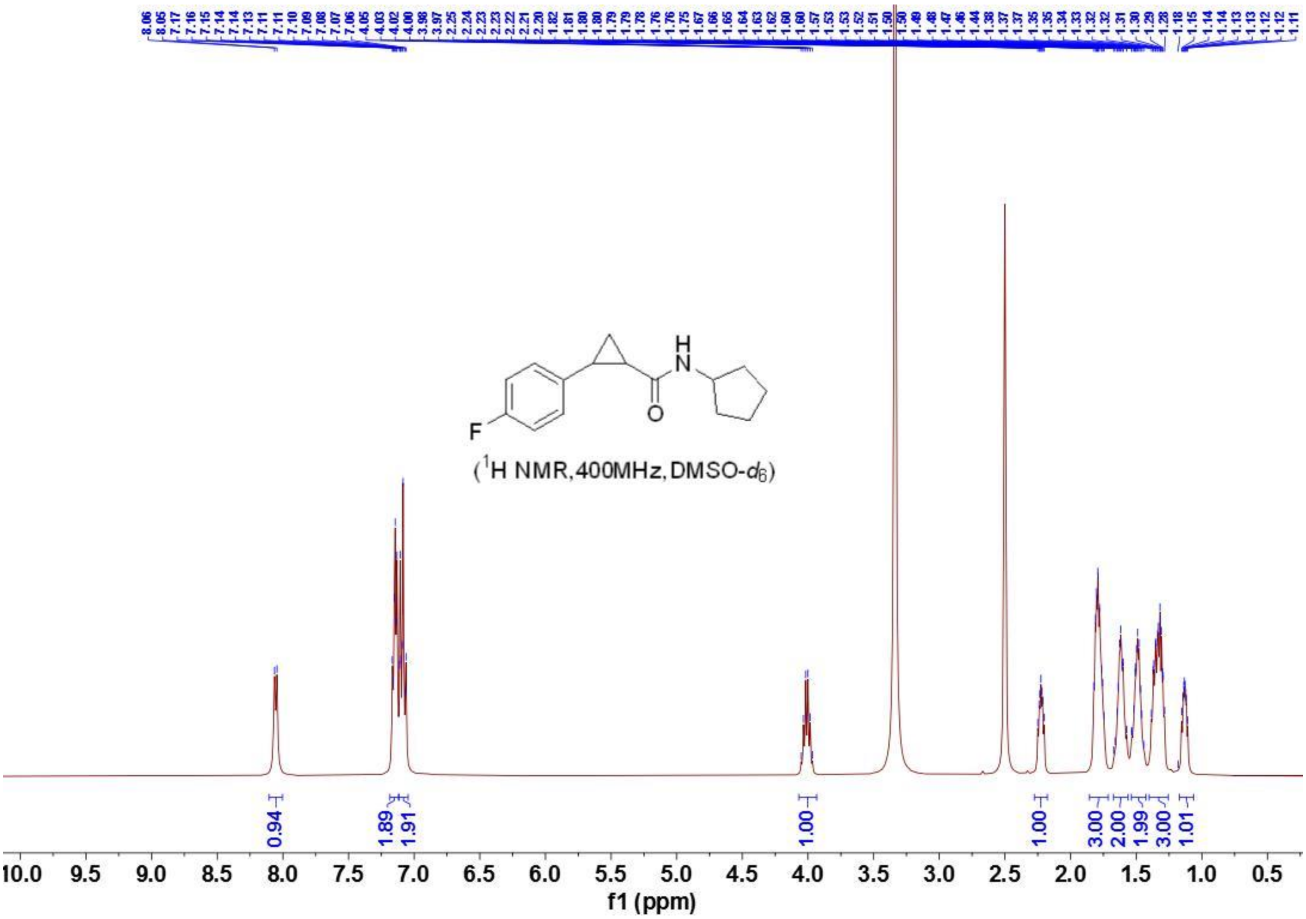

^{1}H NMR of compound **F38**

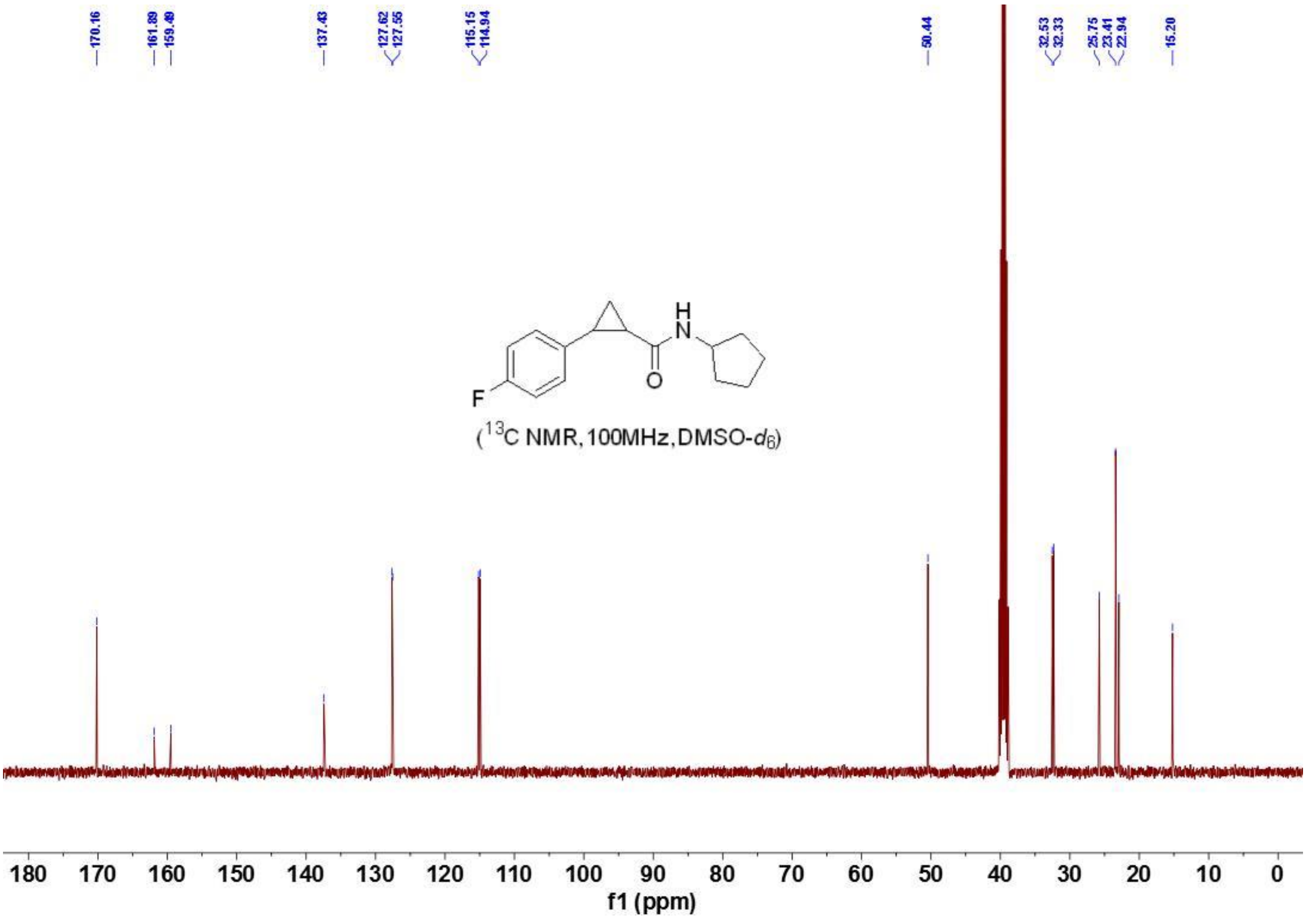

^{13}C NMR of compound **F38**

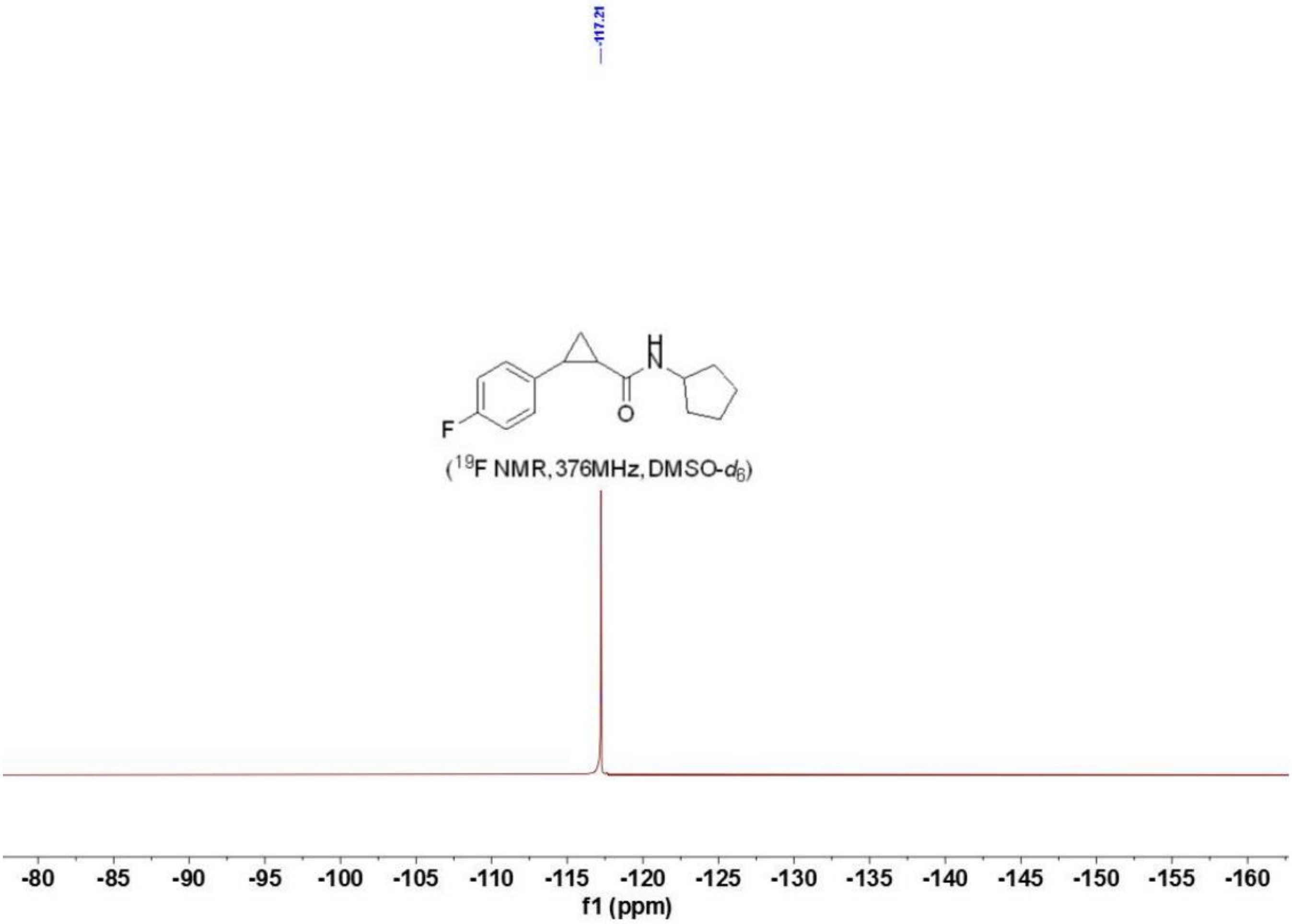

^{19}F NMR of compound **F38**

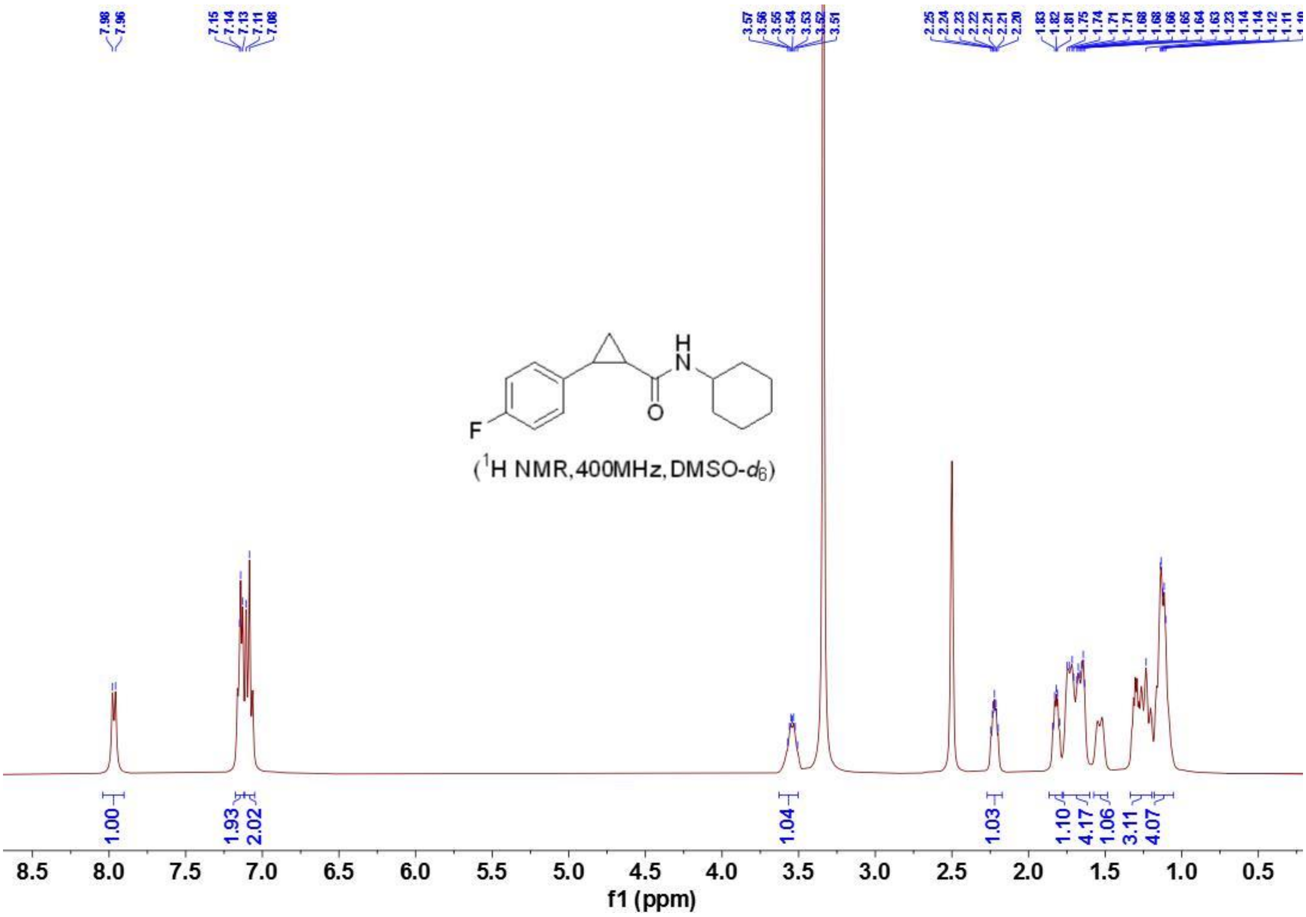

^{1}H NMR of compound **F39**

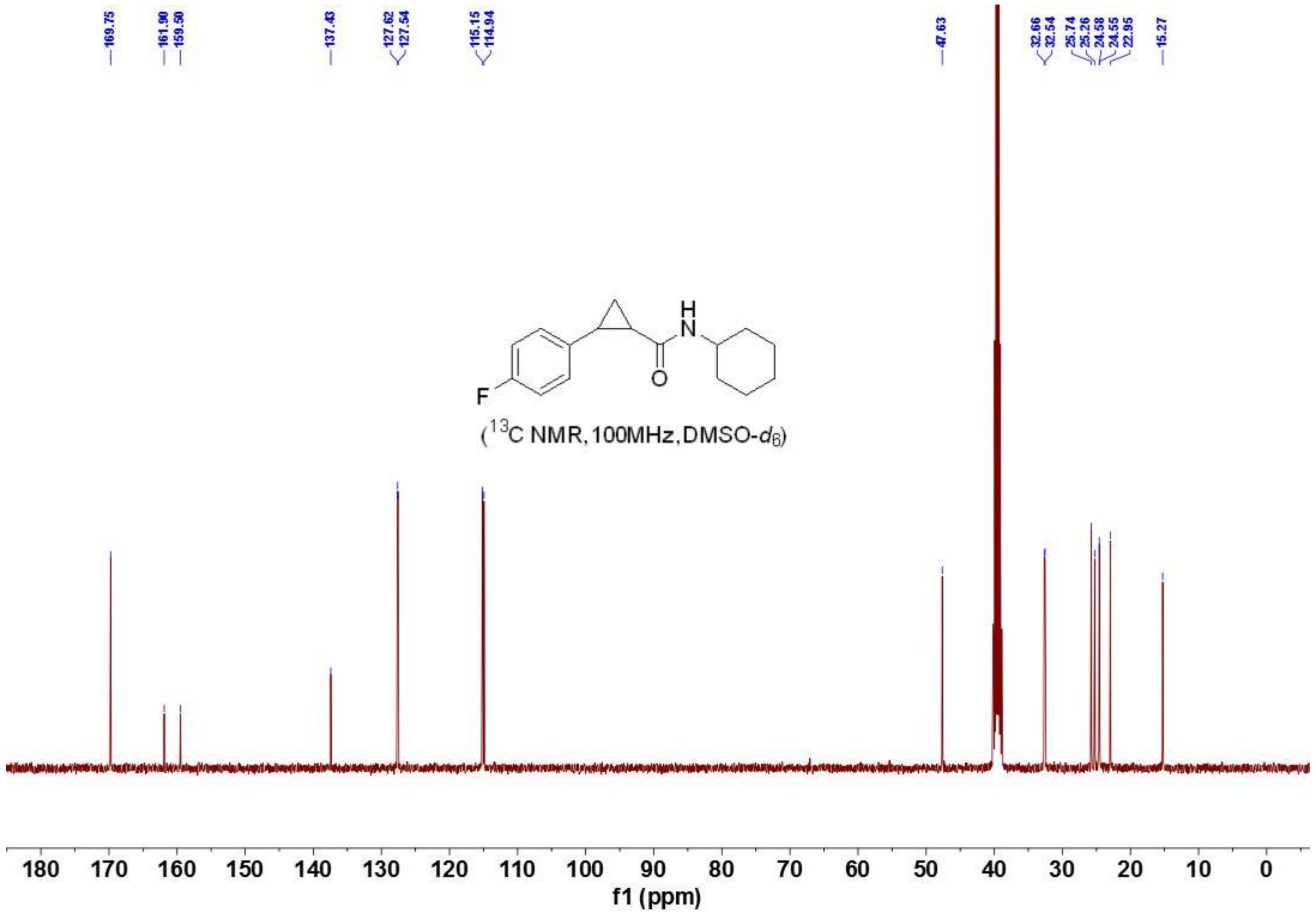

^{13}C NMR of compound **F39**

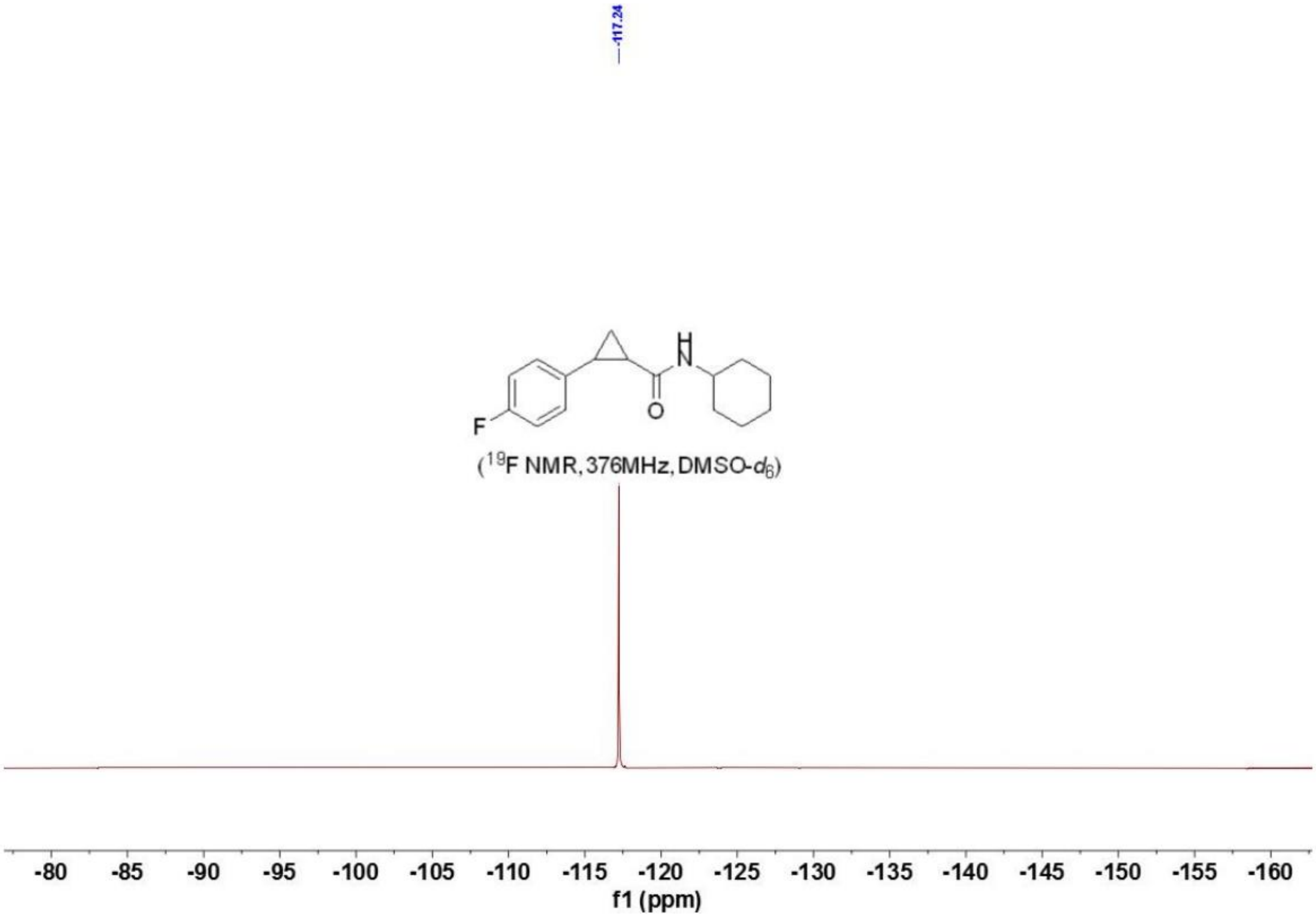

^{19}F NMR of compound **F39**

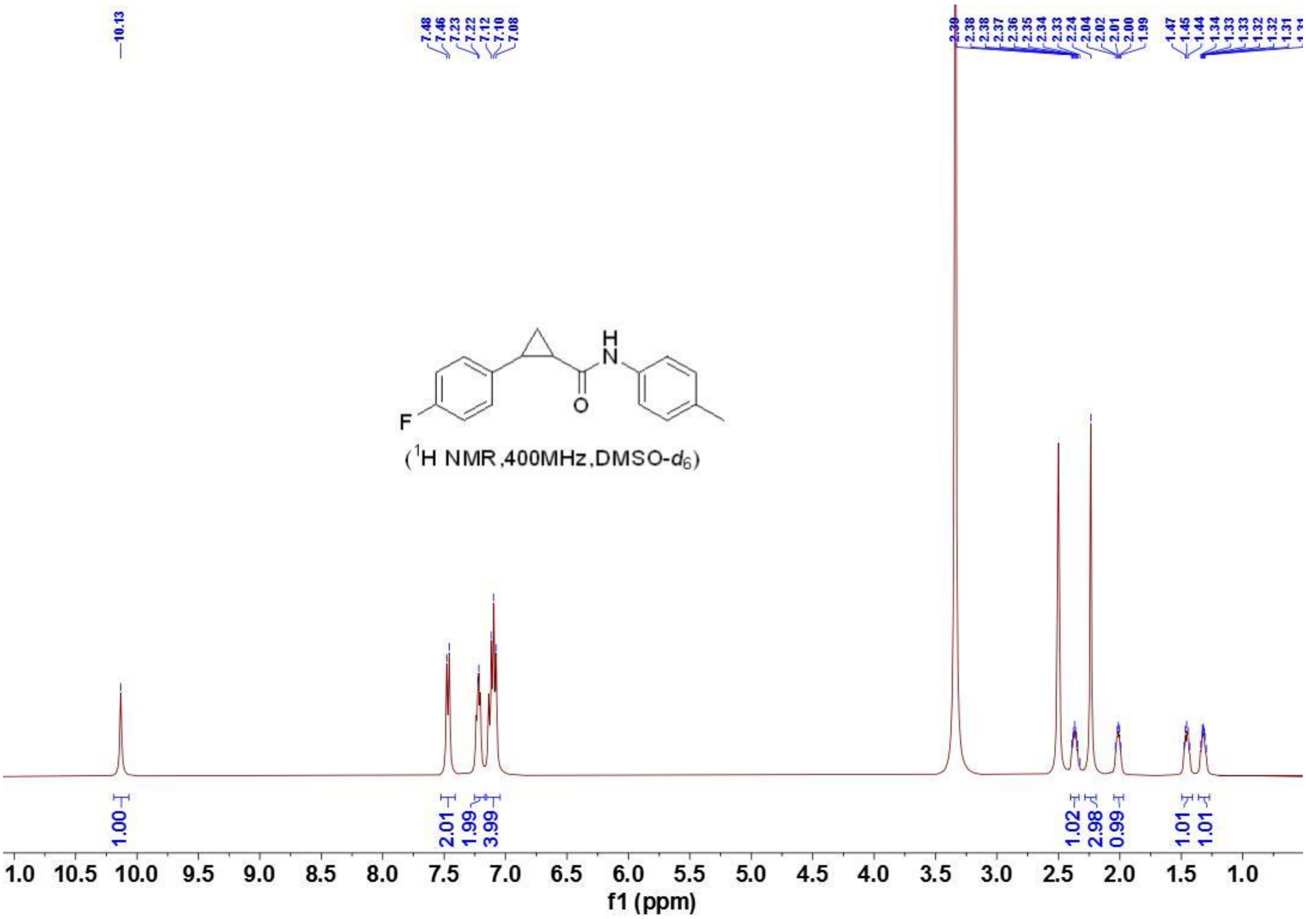

^{1}H NMR of compound **F40**

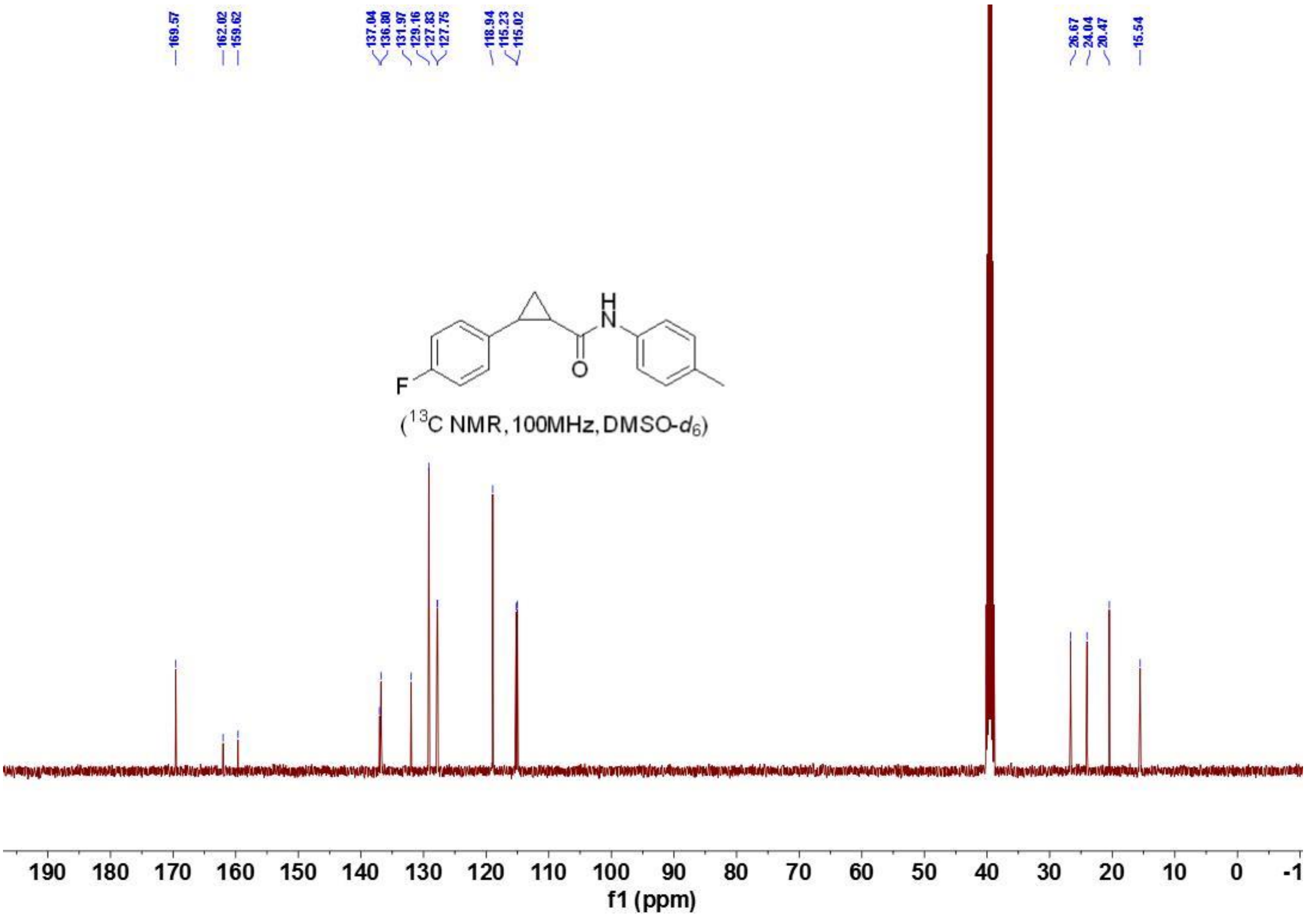

^{13}C NMR of compound **F40**

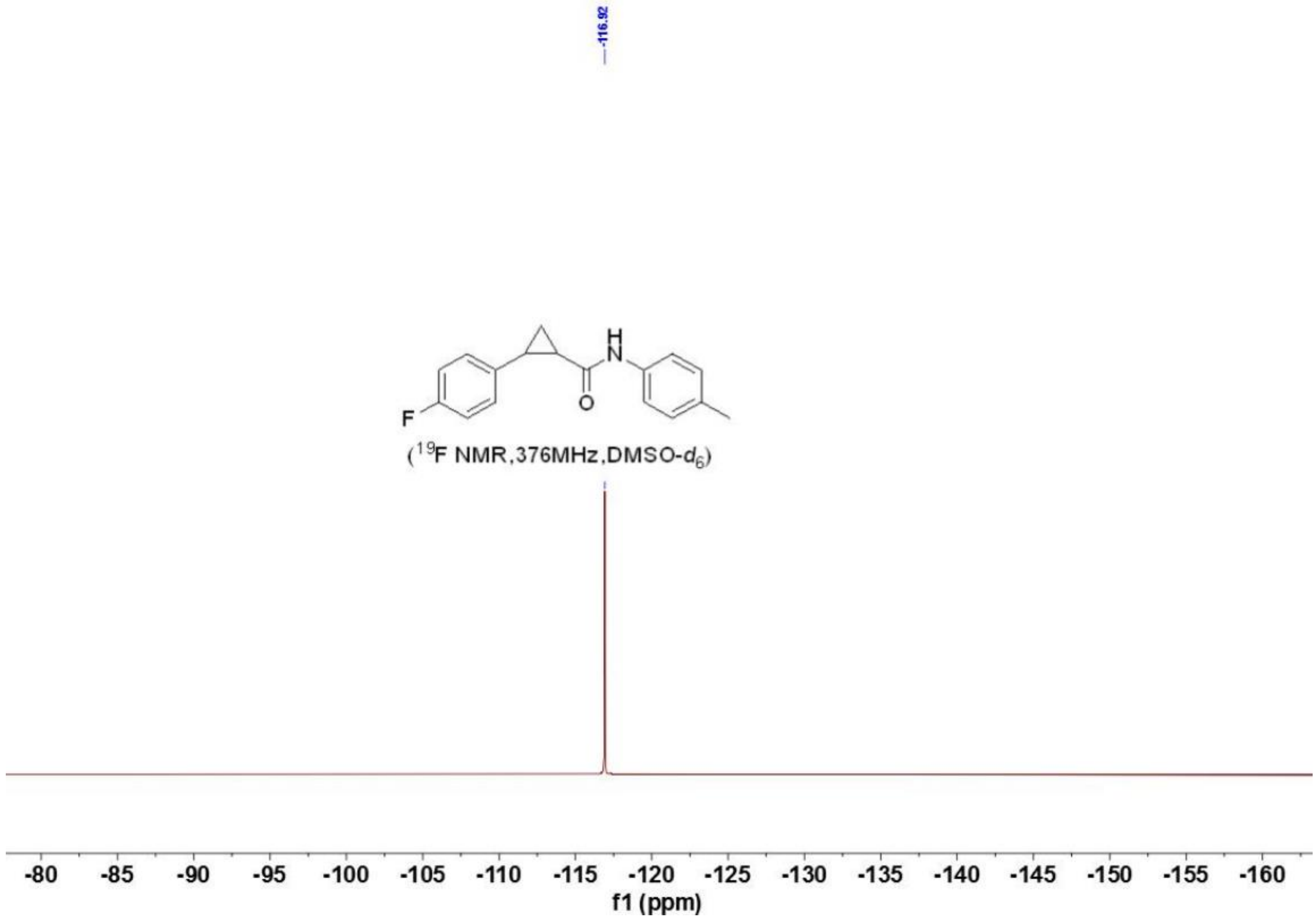

^{19}F NMR of compound **F40**

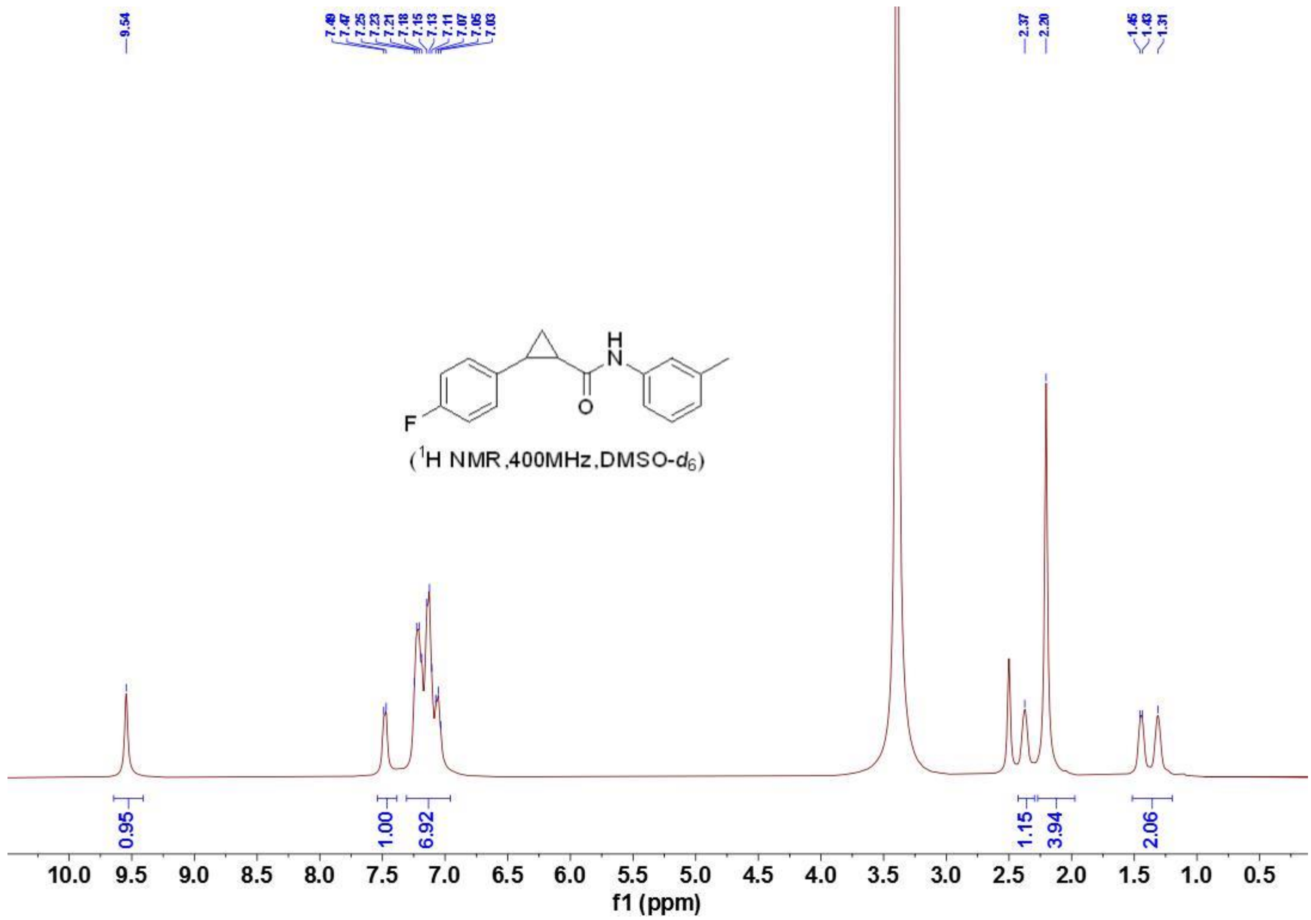

^{1}H NMR of compound **F41**

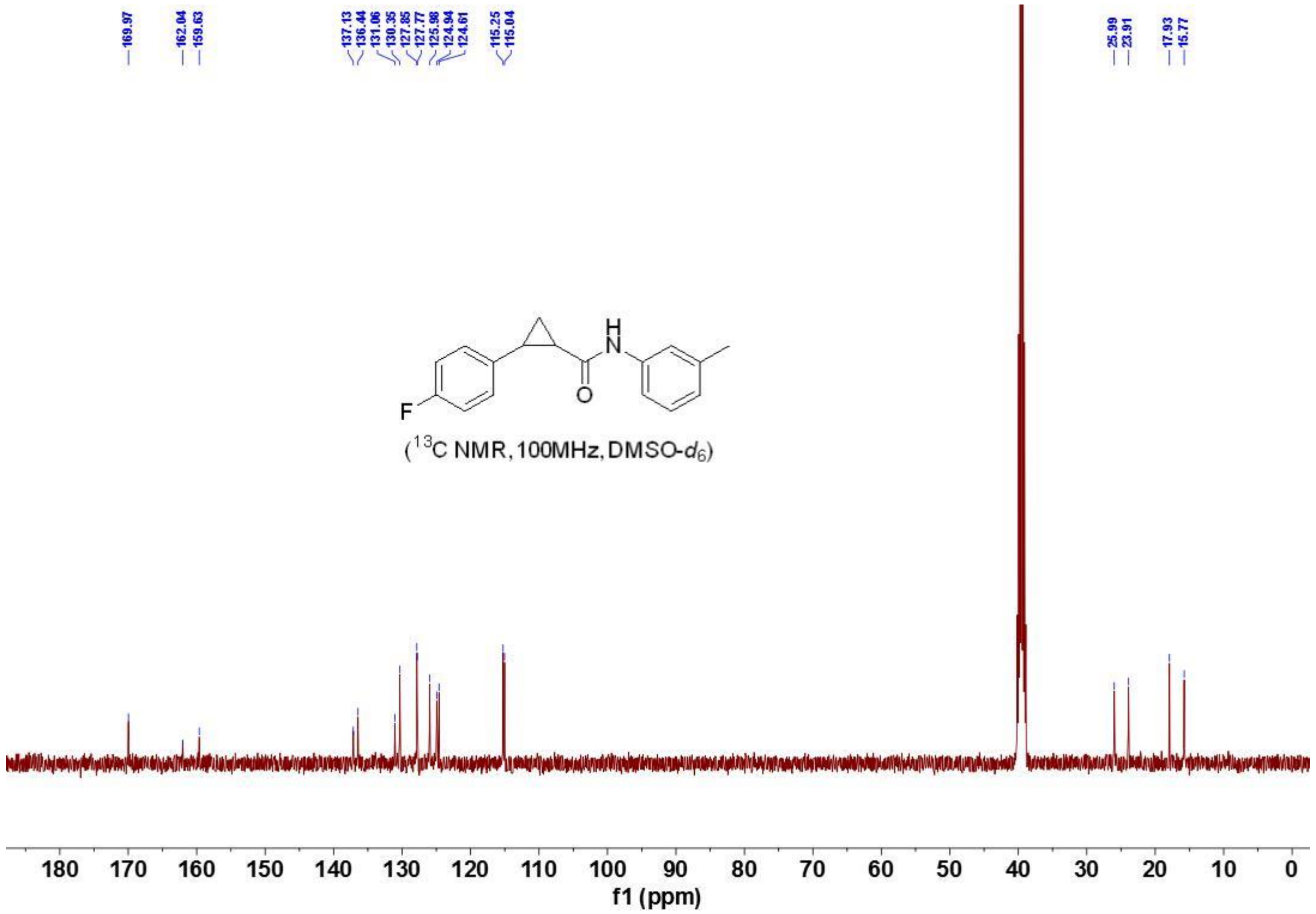

^{13}C NMR of compound **F41**

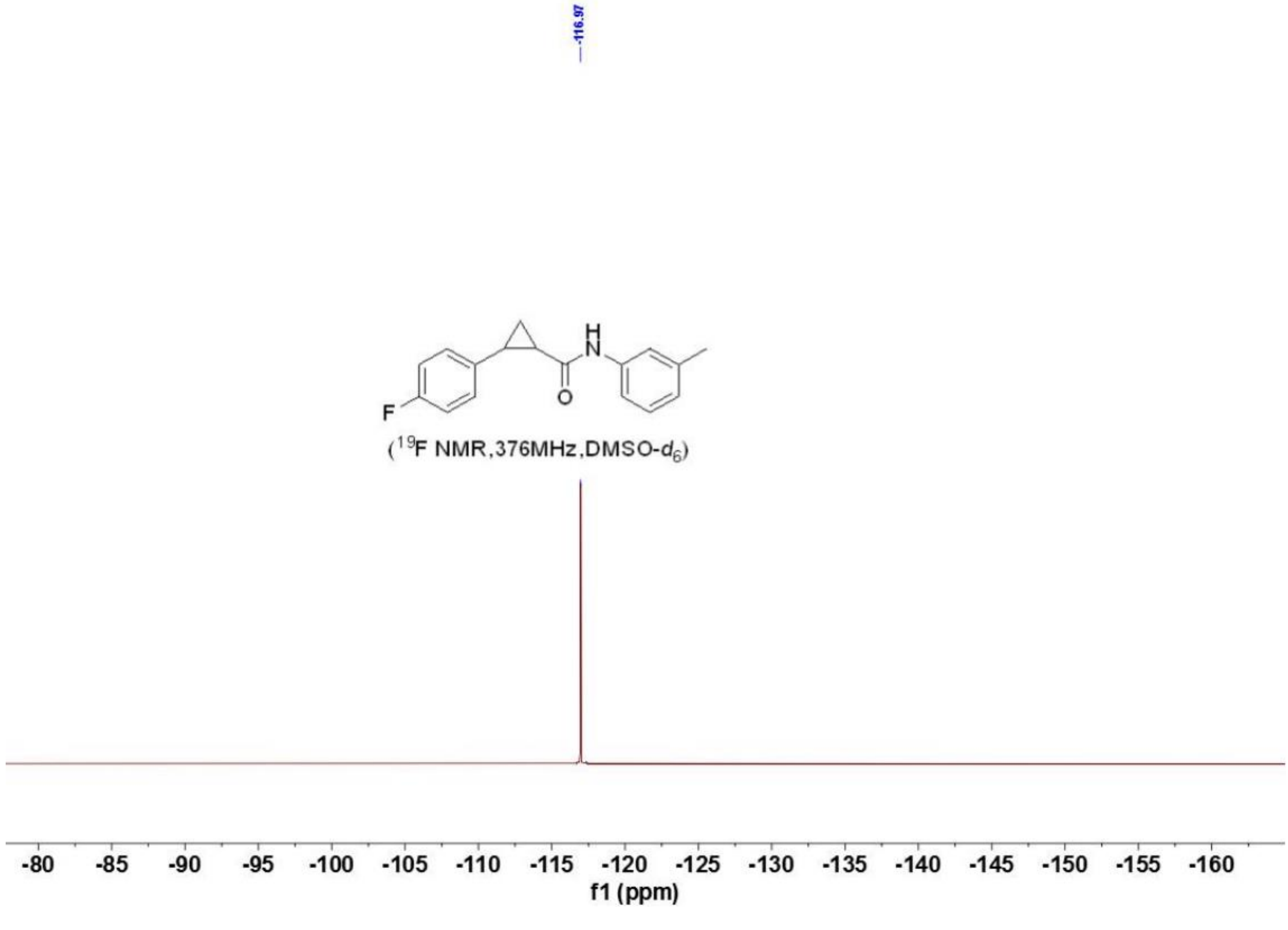

^{19}F NMR of compound **F41**

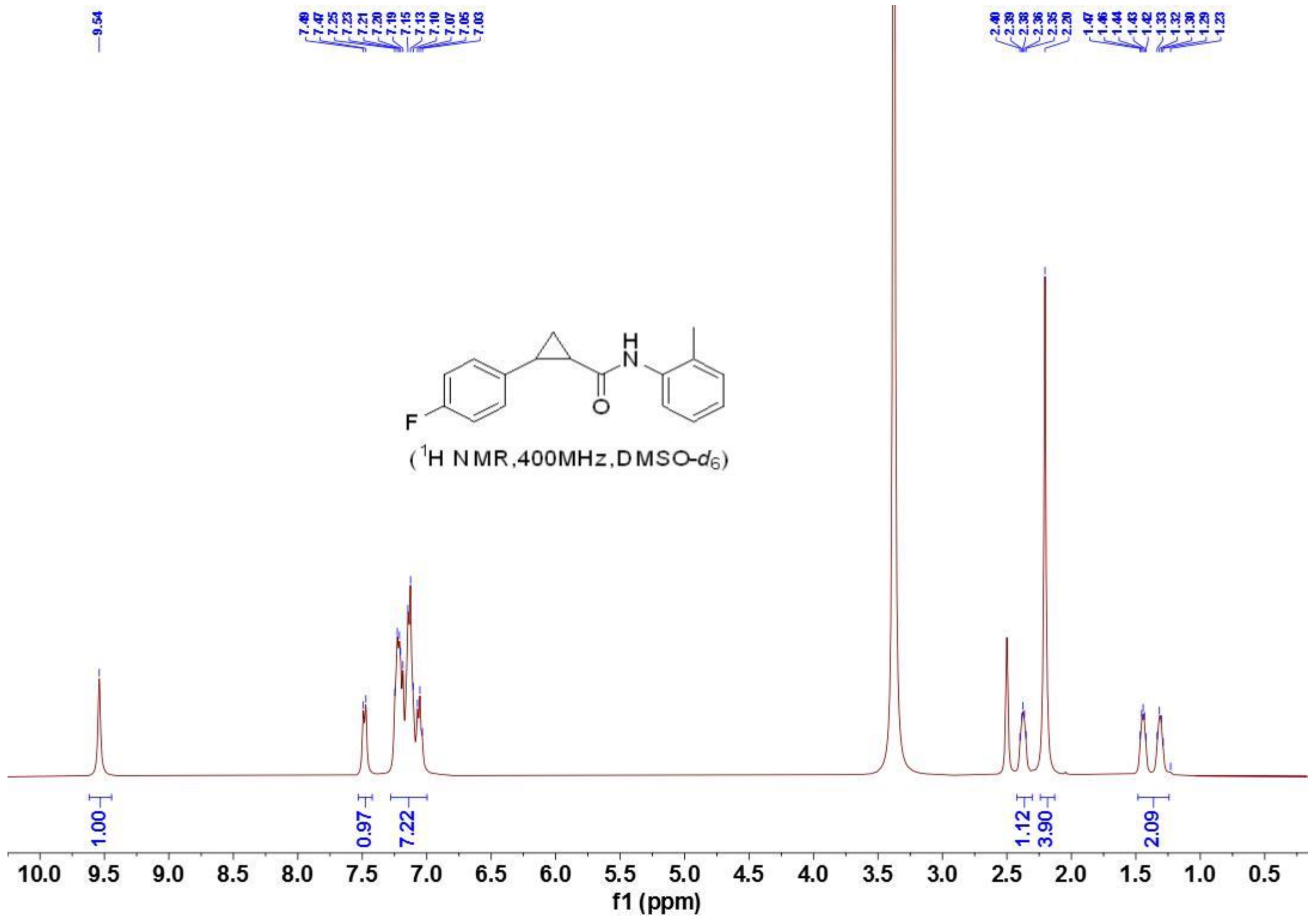

^{1}H NMR of compound **F42**

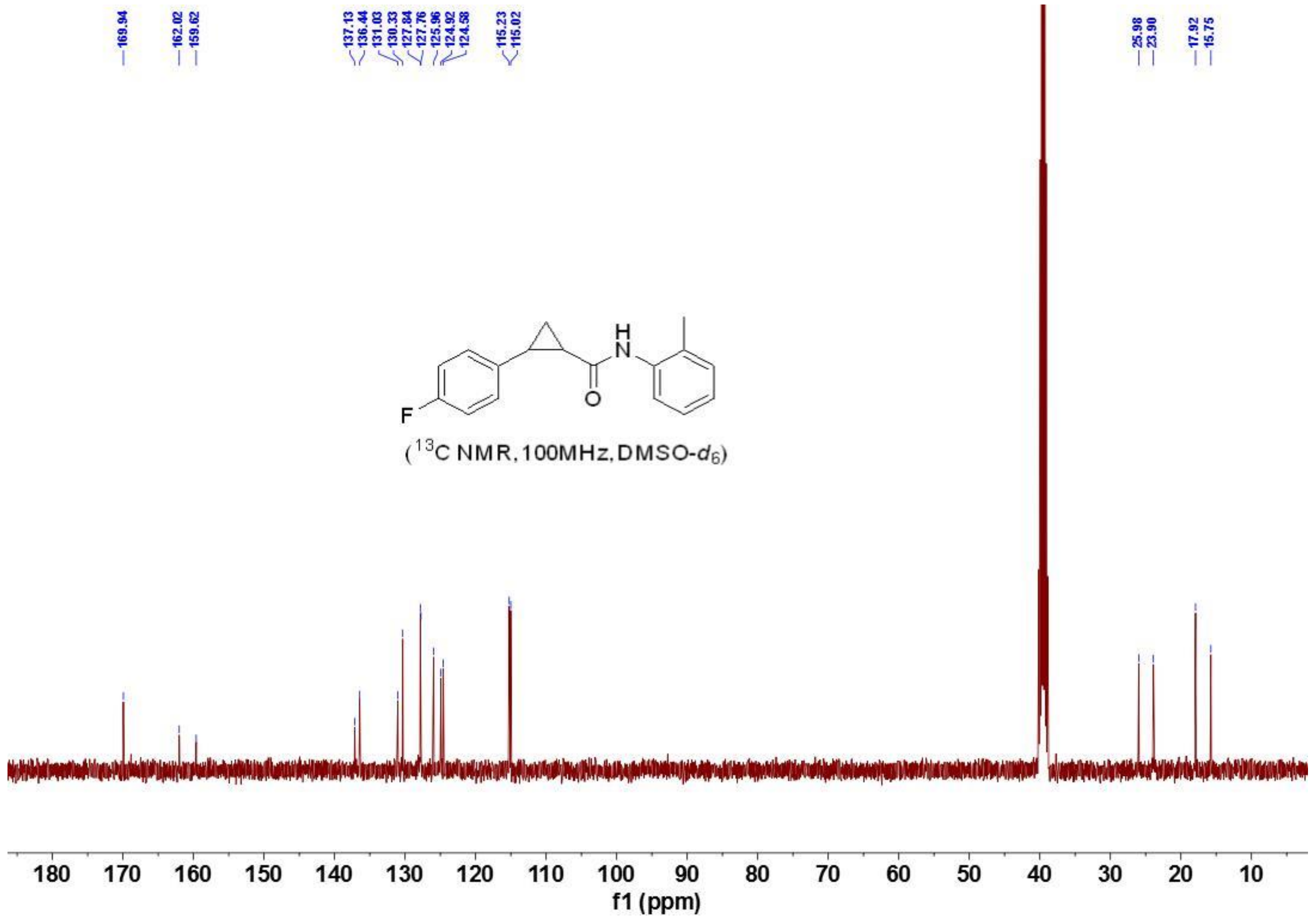

^{13}C NMR of compound **F42**

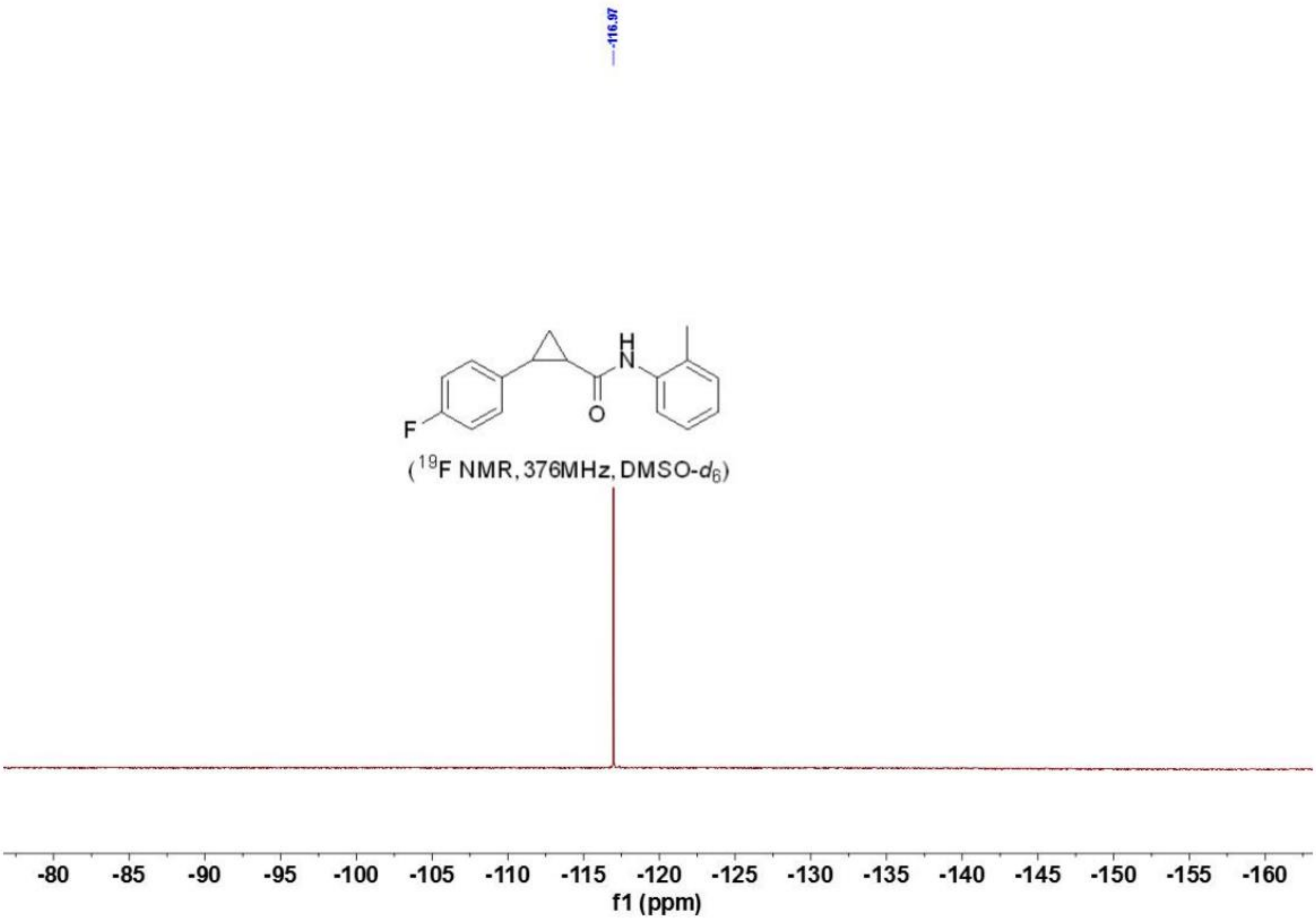

^{19}F NMR of compound **F42**

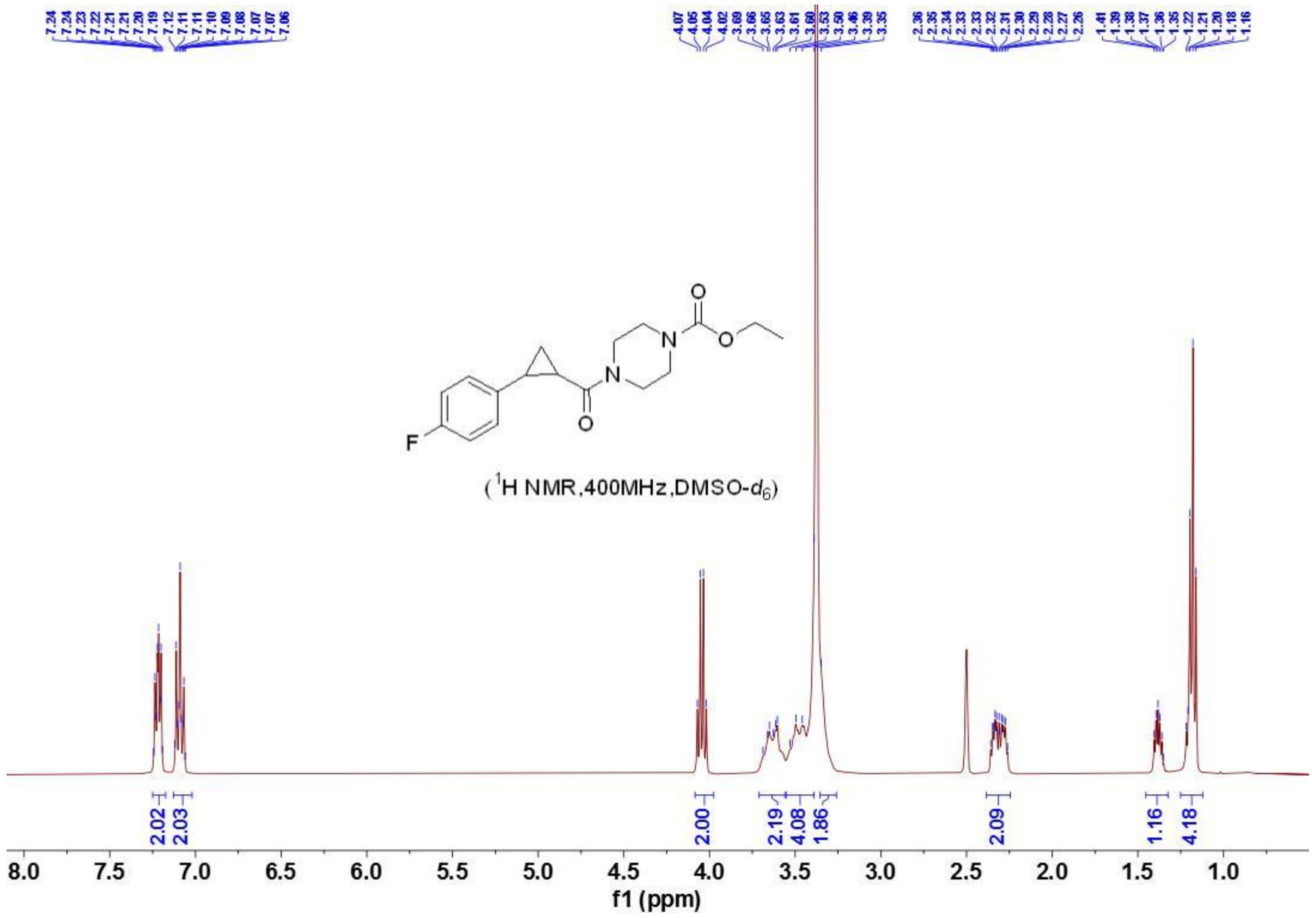

^{1}H NMR of compound **F43**

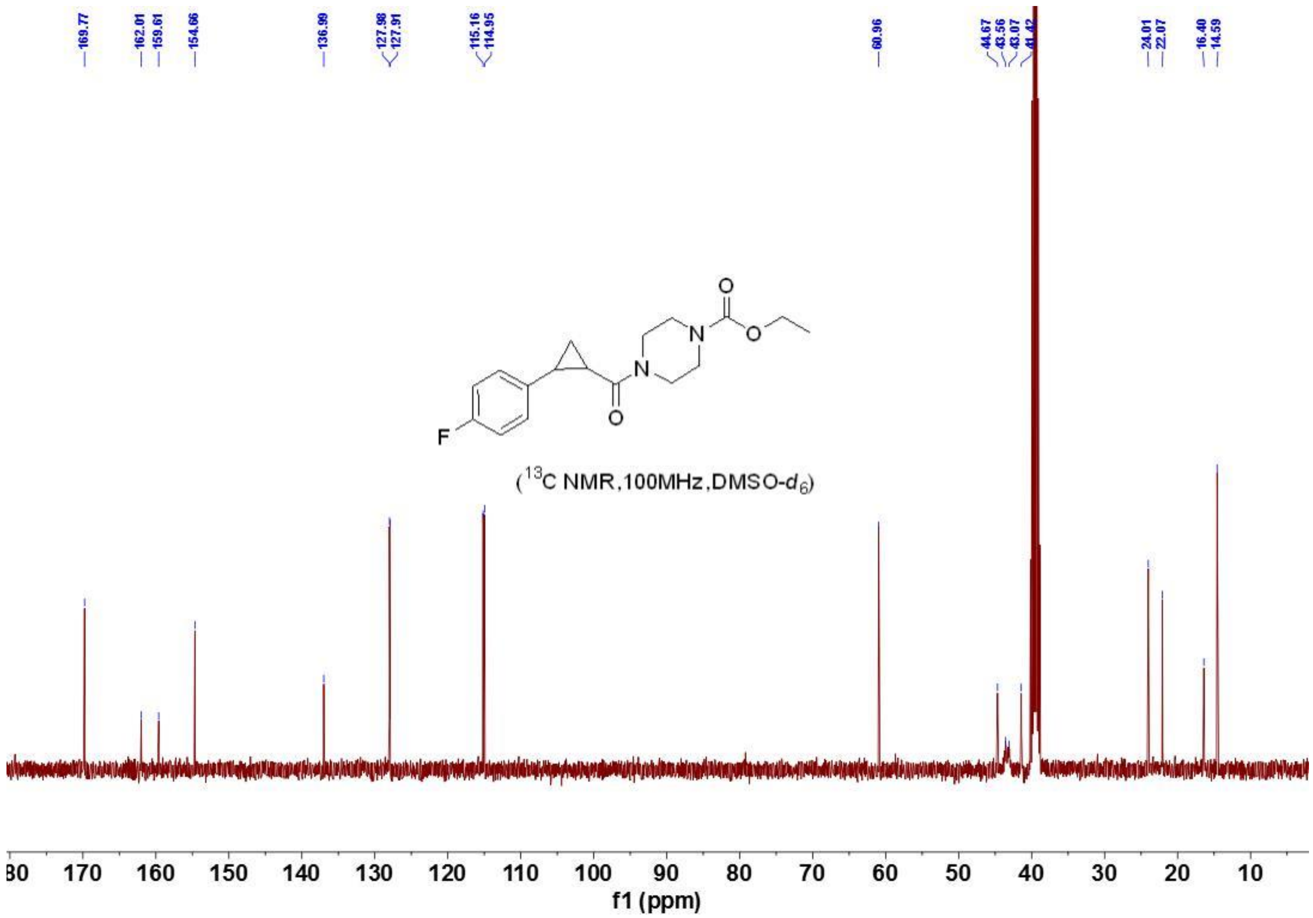

^{13}C NMR of compound **F43**

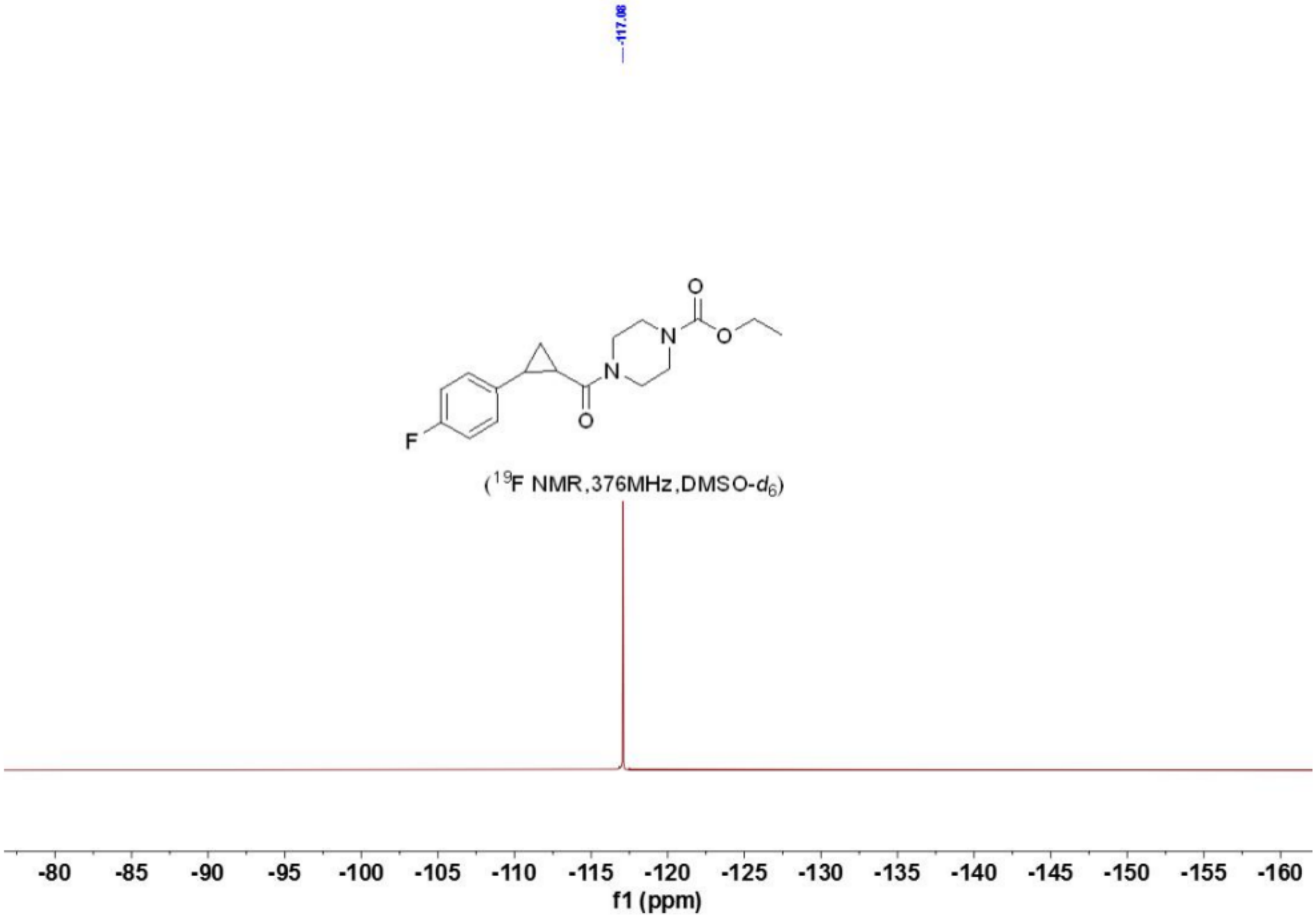

^{19}F NMR of compound **F43**

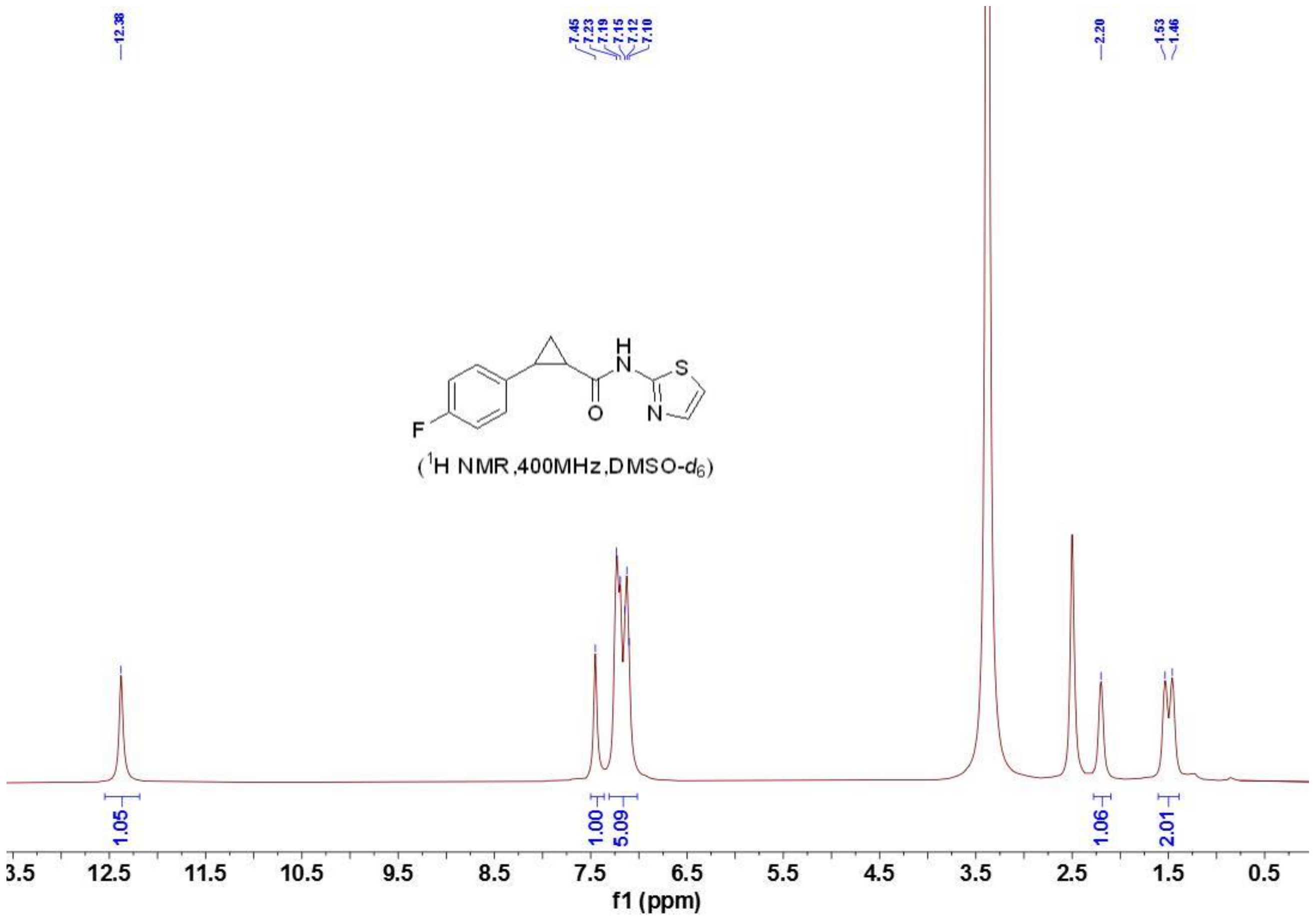

^{1}H NMR of compound **F44**

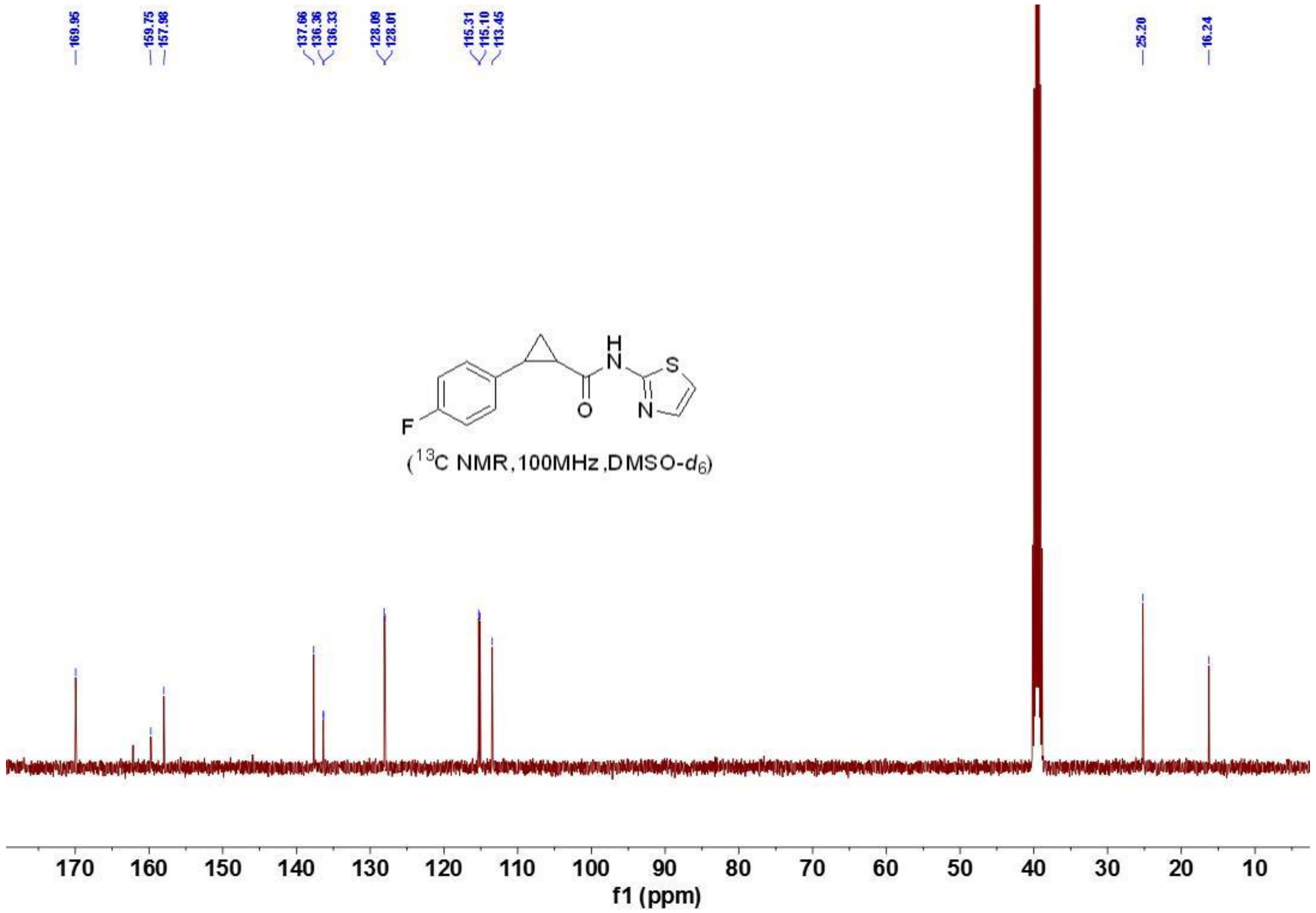

^{13}C NMR of compound **F44**

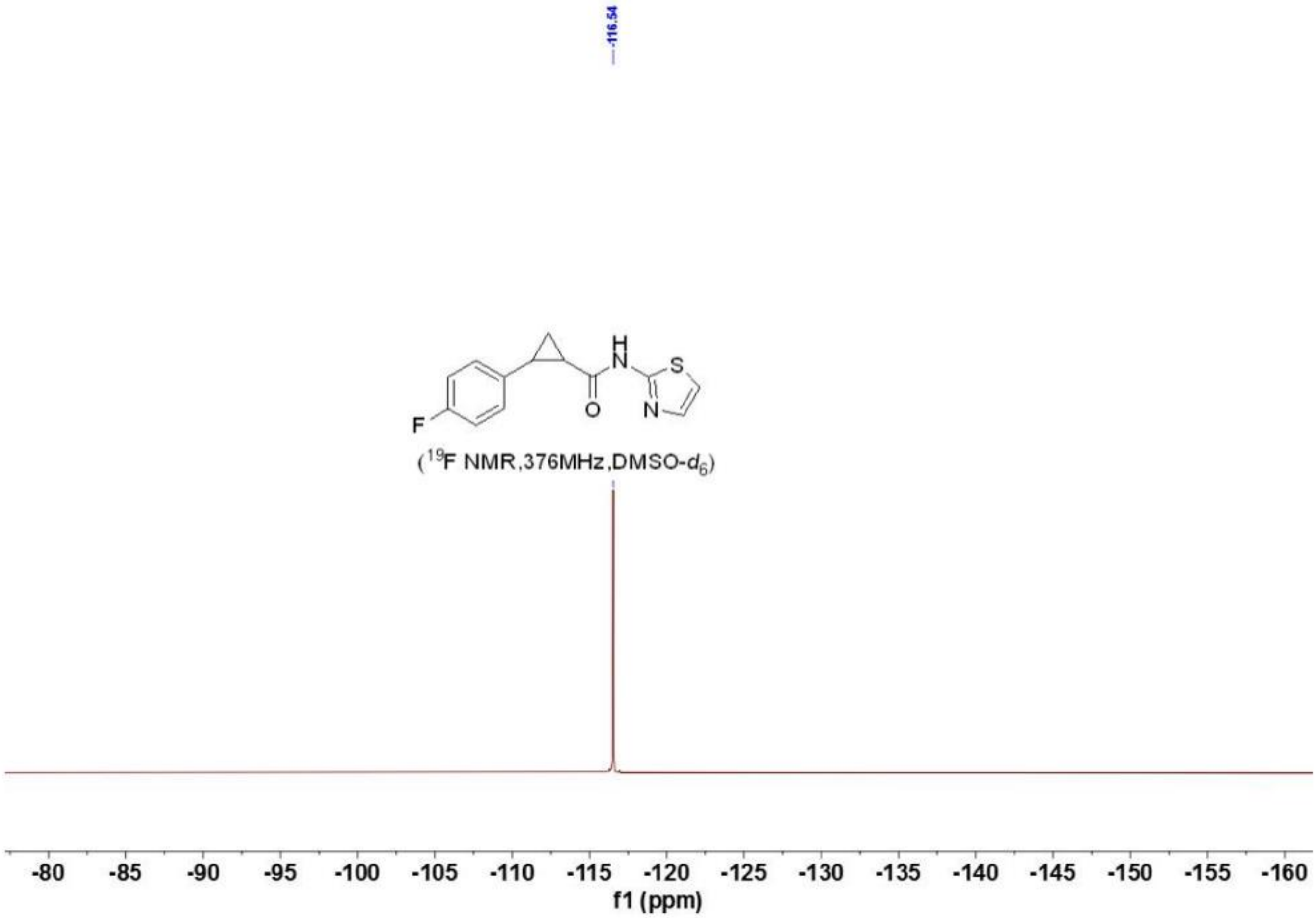

^{19}F NMR of compound **F44**

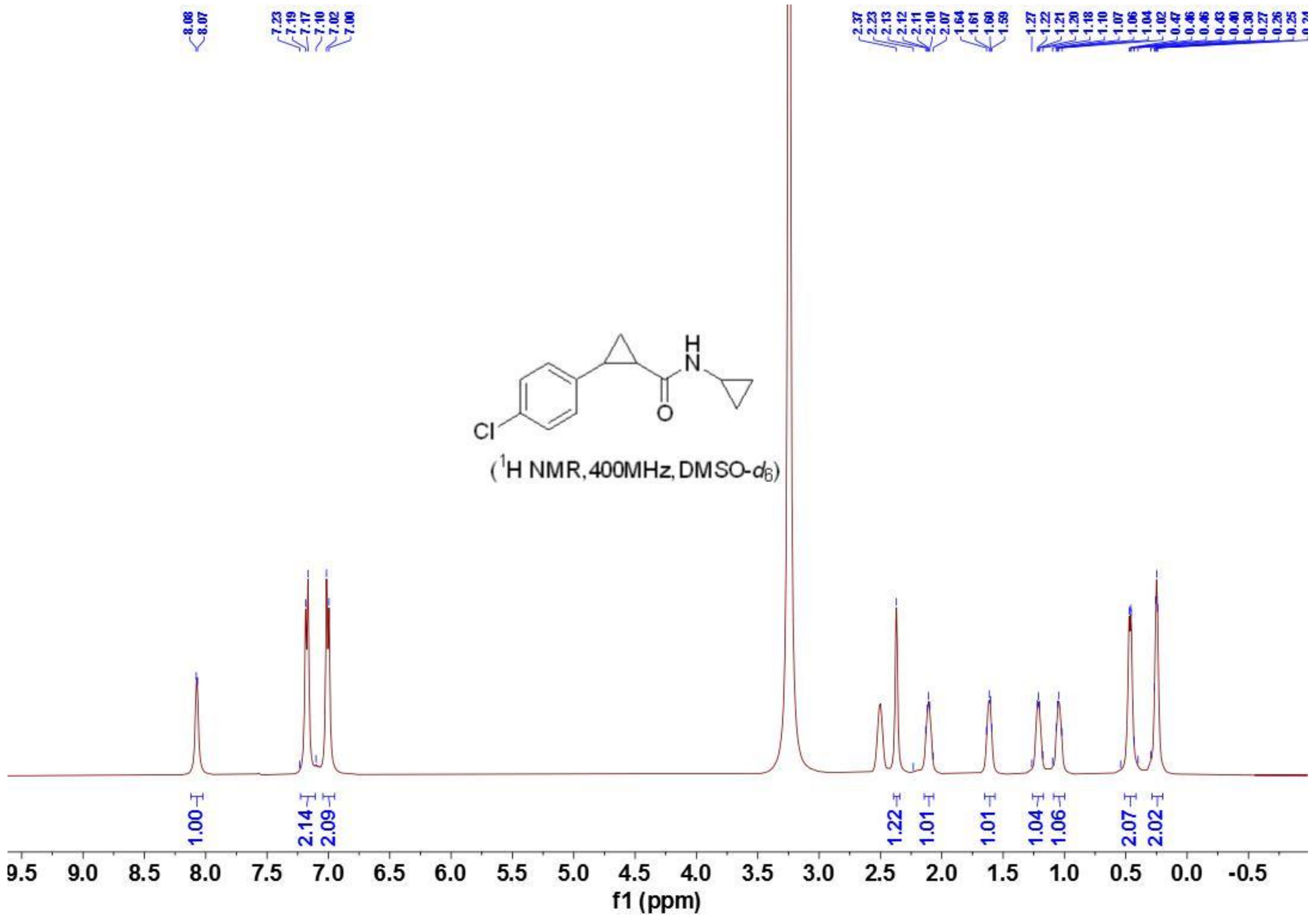

^{1}H NMR of compound **F45**

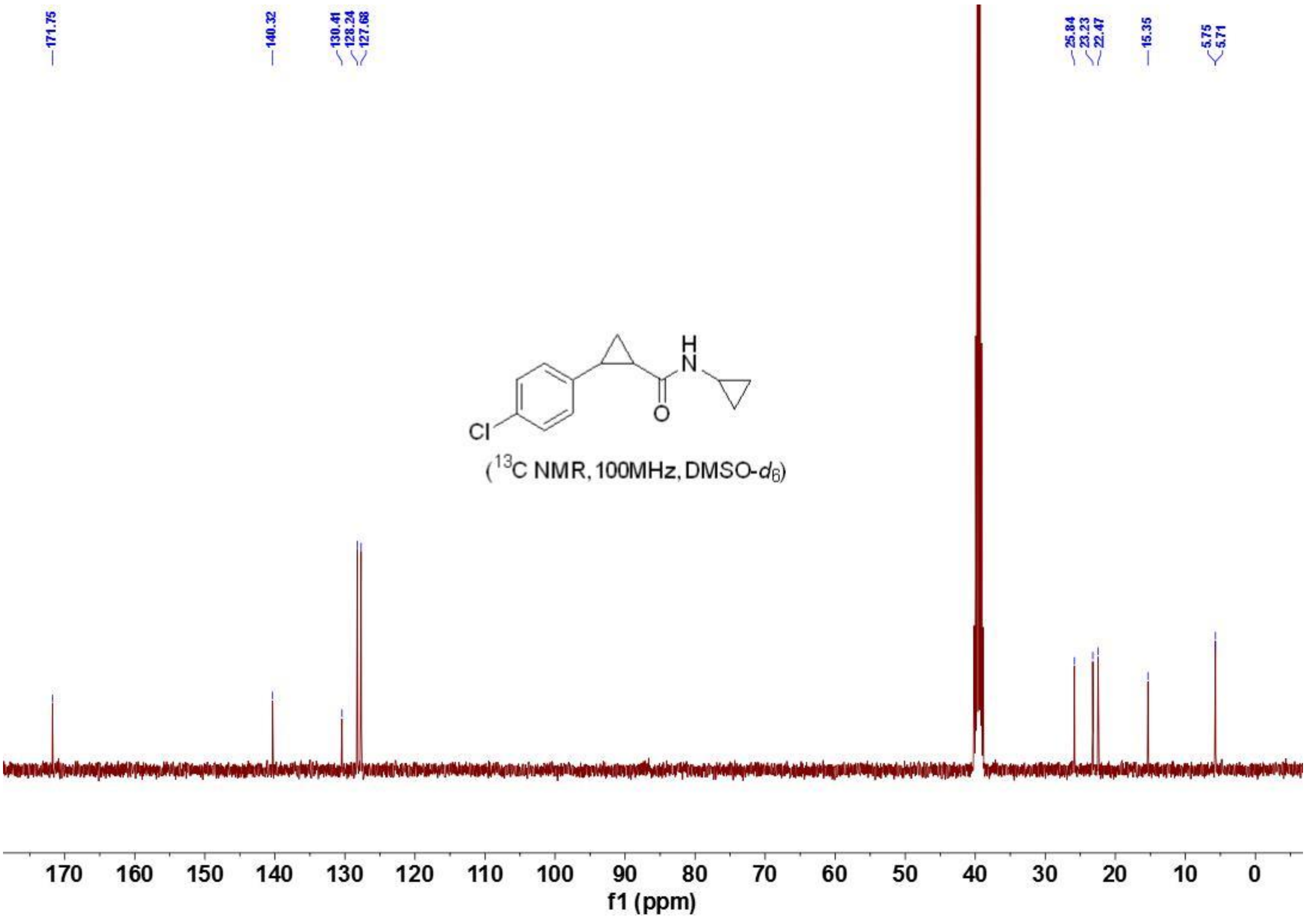

^{13}C NMR of compound **F45**

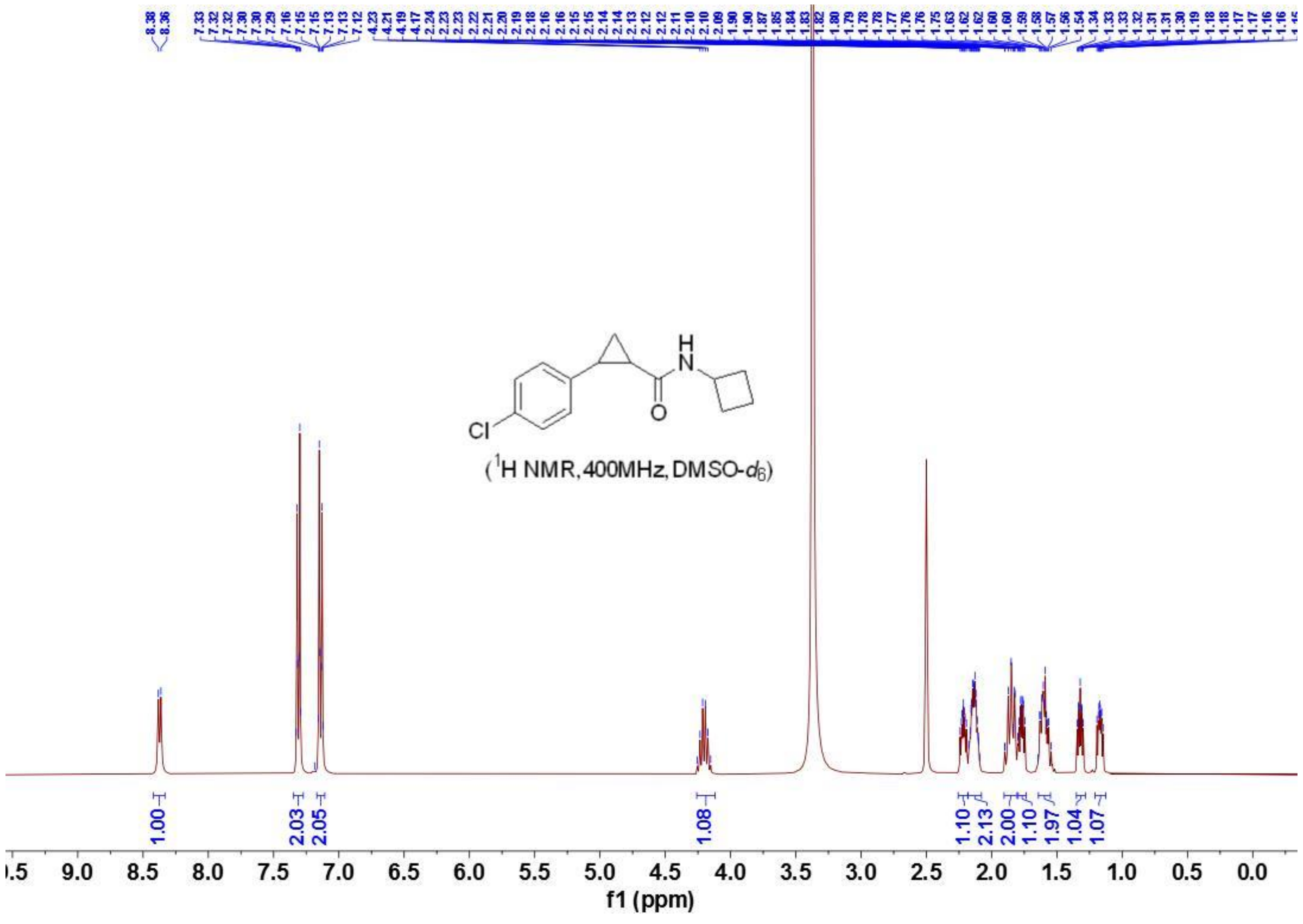

^{1}H NMR of compound **F46**

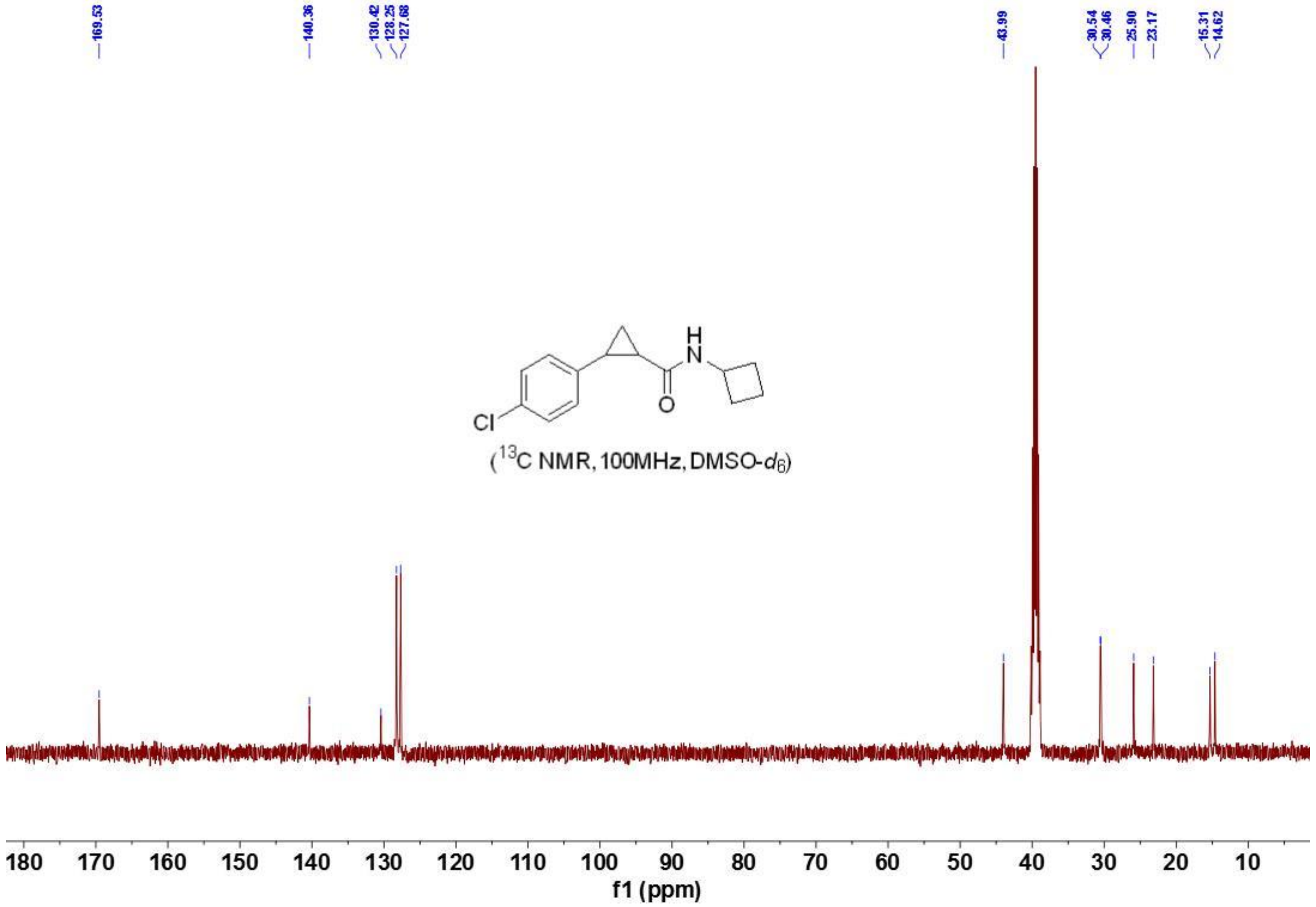

^{13}C NMR of compound **F46**

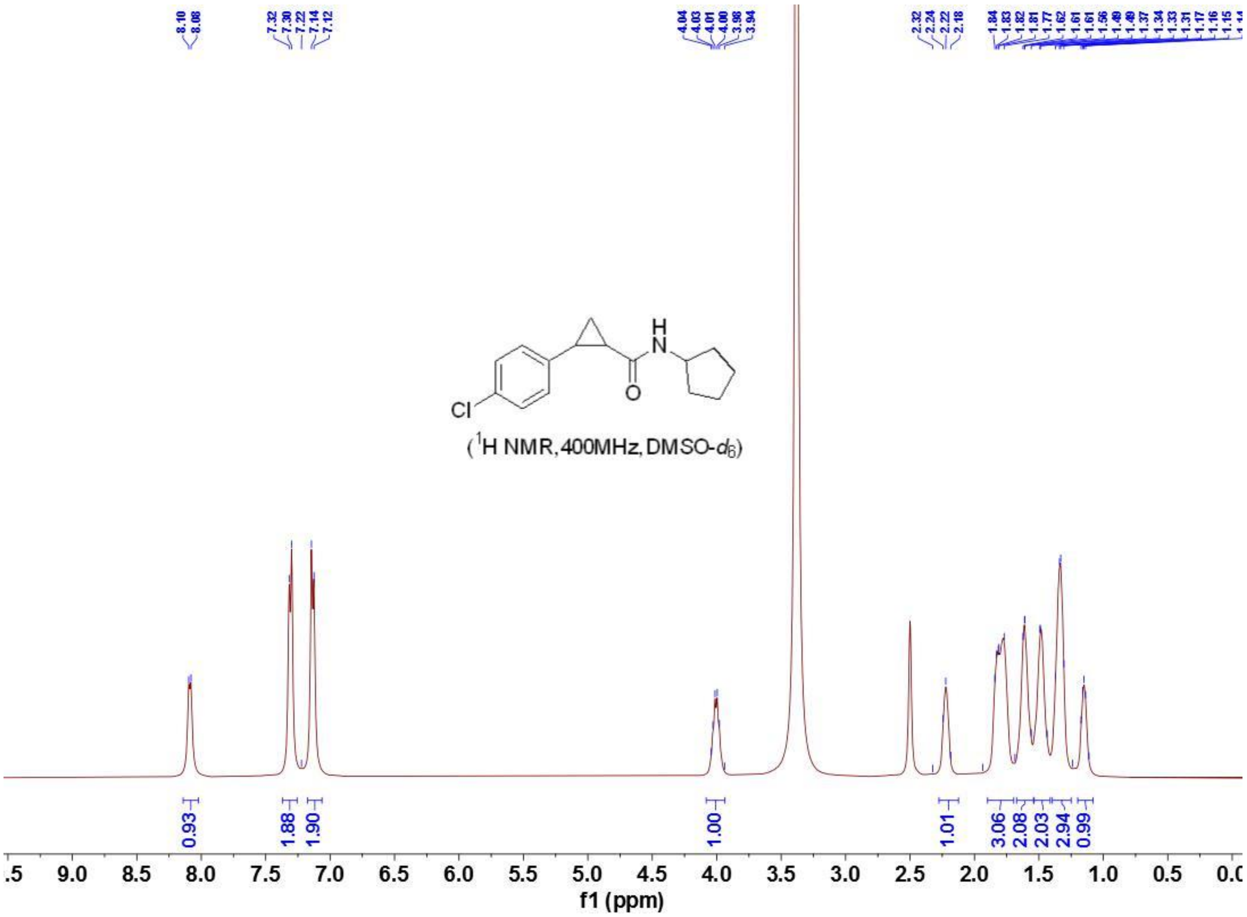

^{1}H NMR of compound **F47**

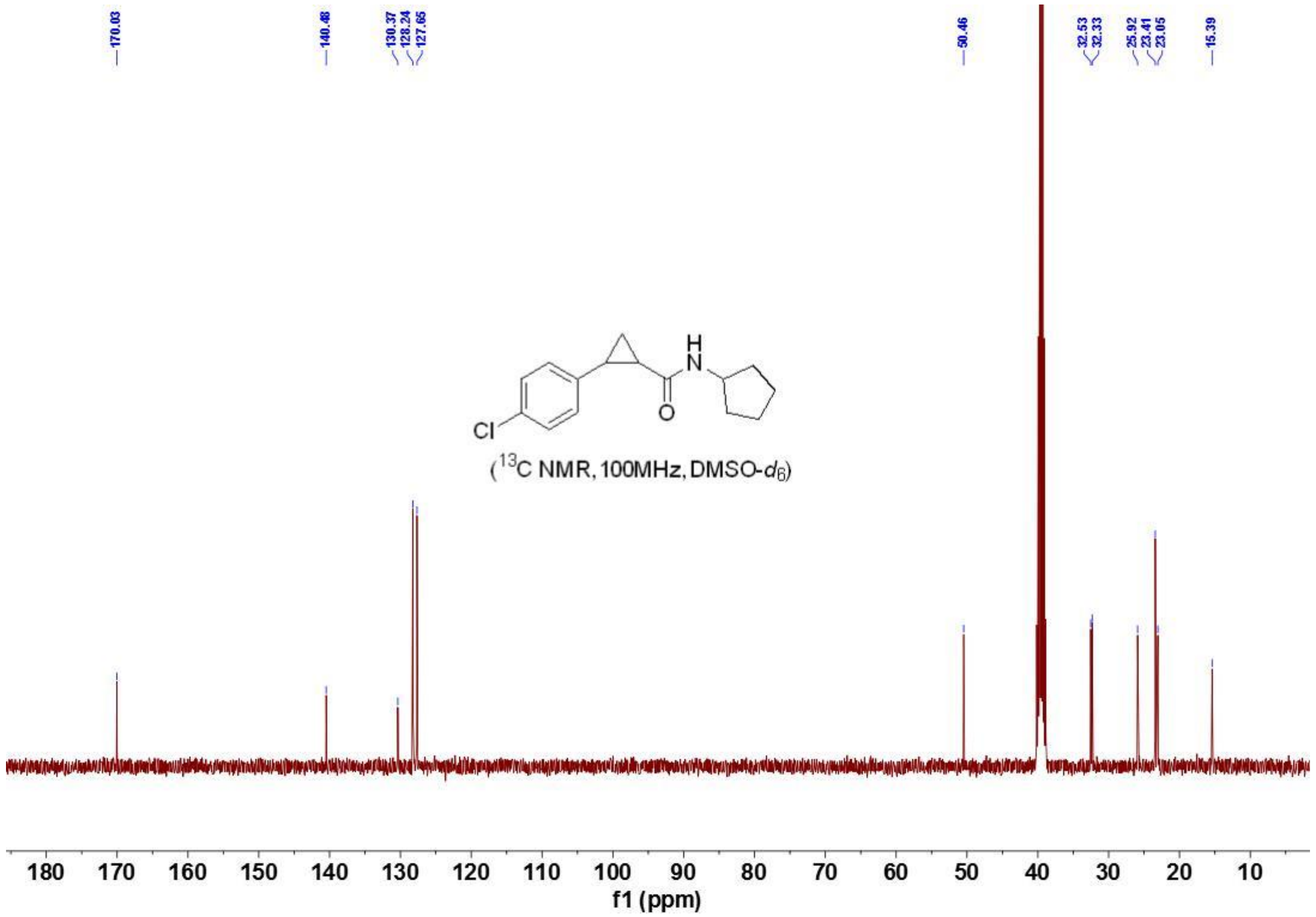

^{13}C NMR of compound **F47**

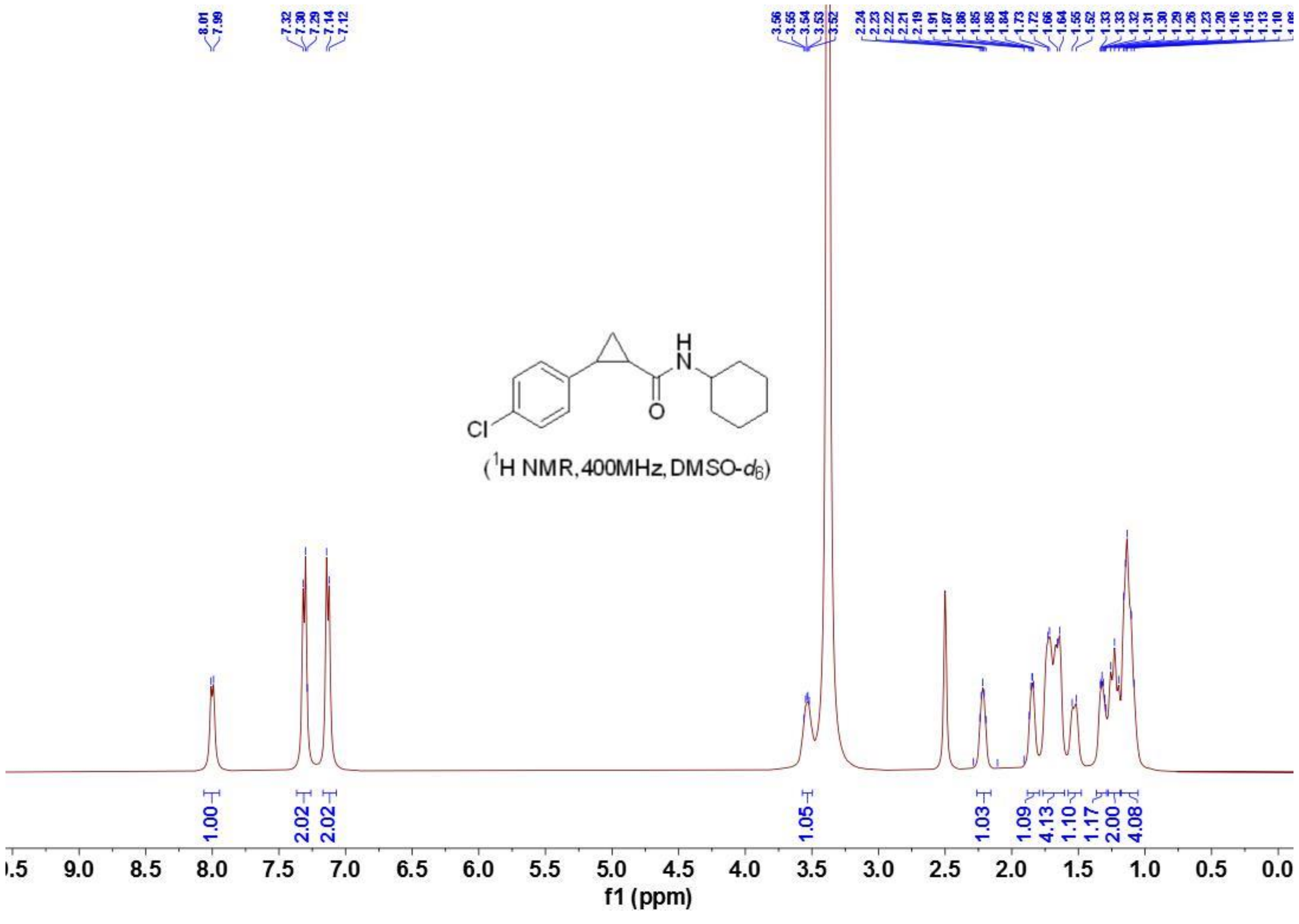

^{1}H NMR of compound **F48**

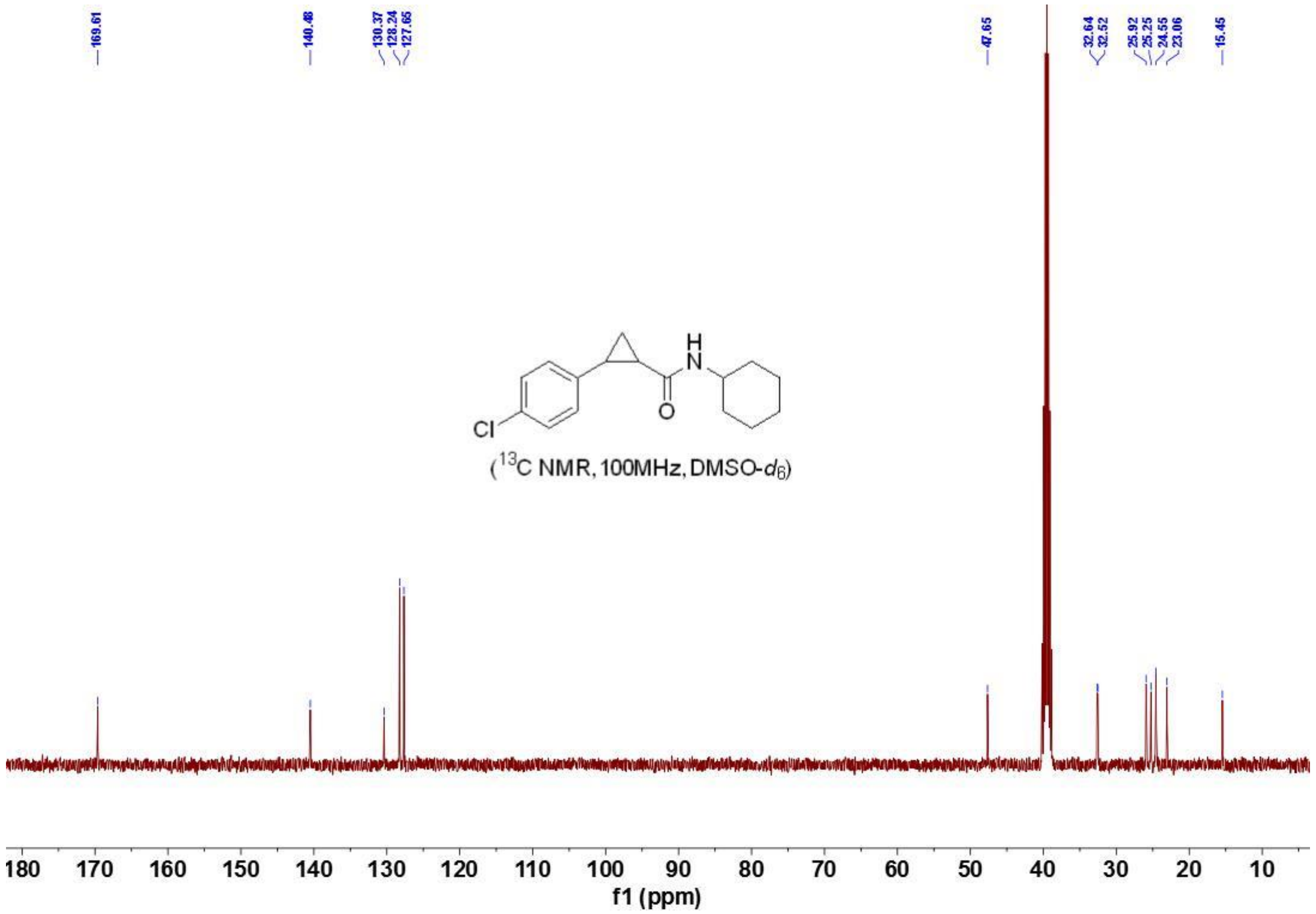

^{13}C NMR of compound **F48**

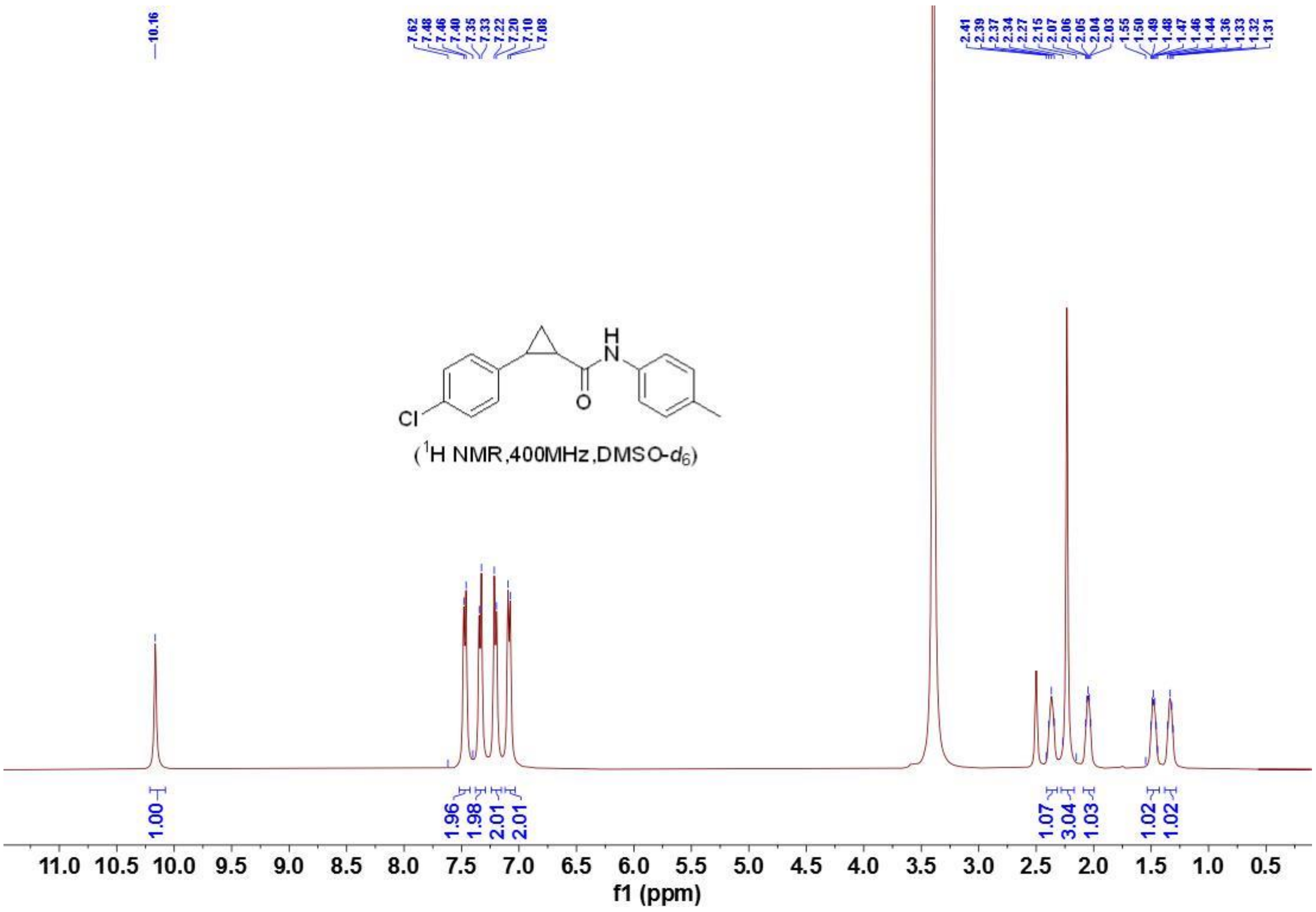

^{1}H NMR of compound **F49**

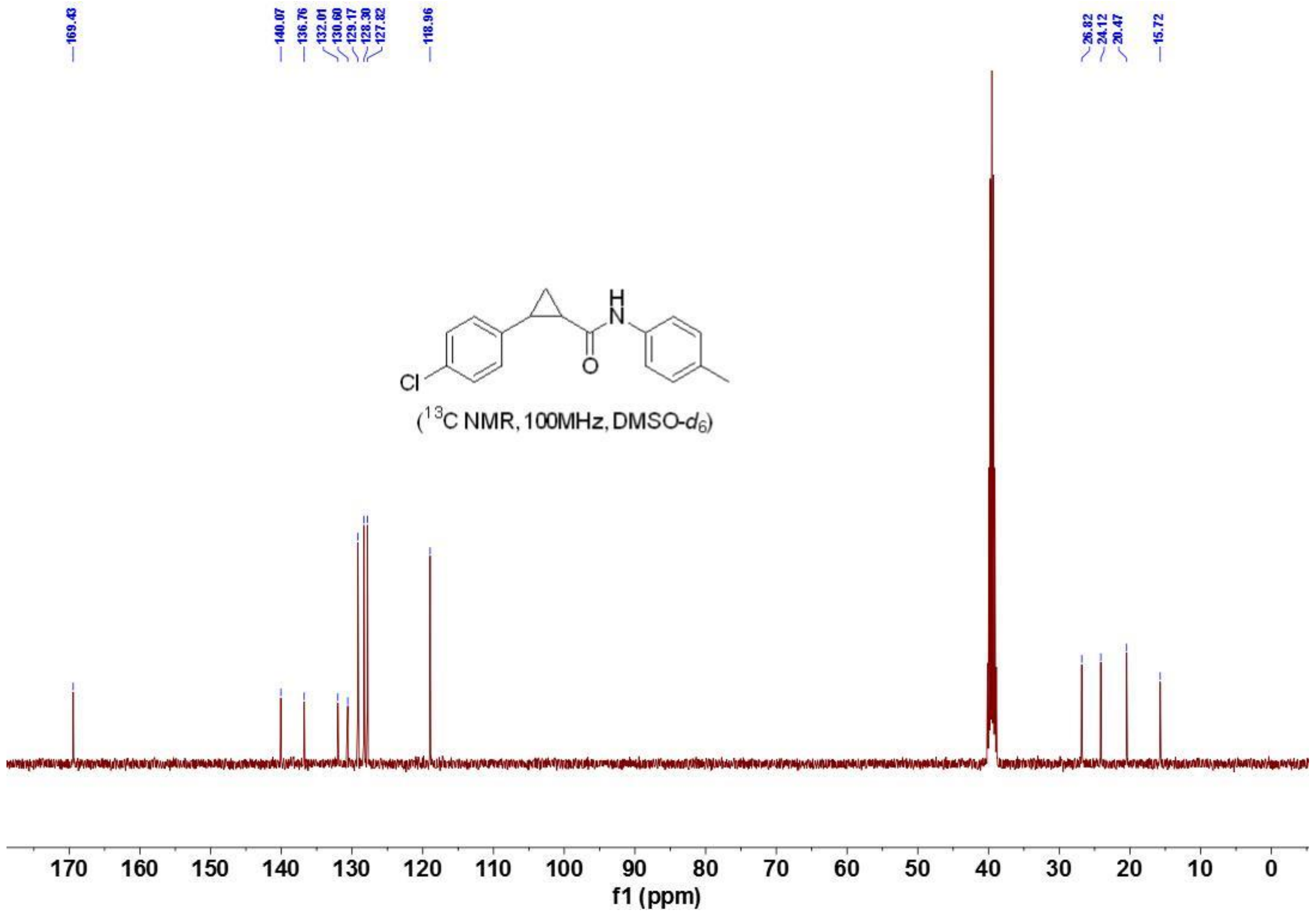

^{13}C NMR of compound **F49**

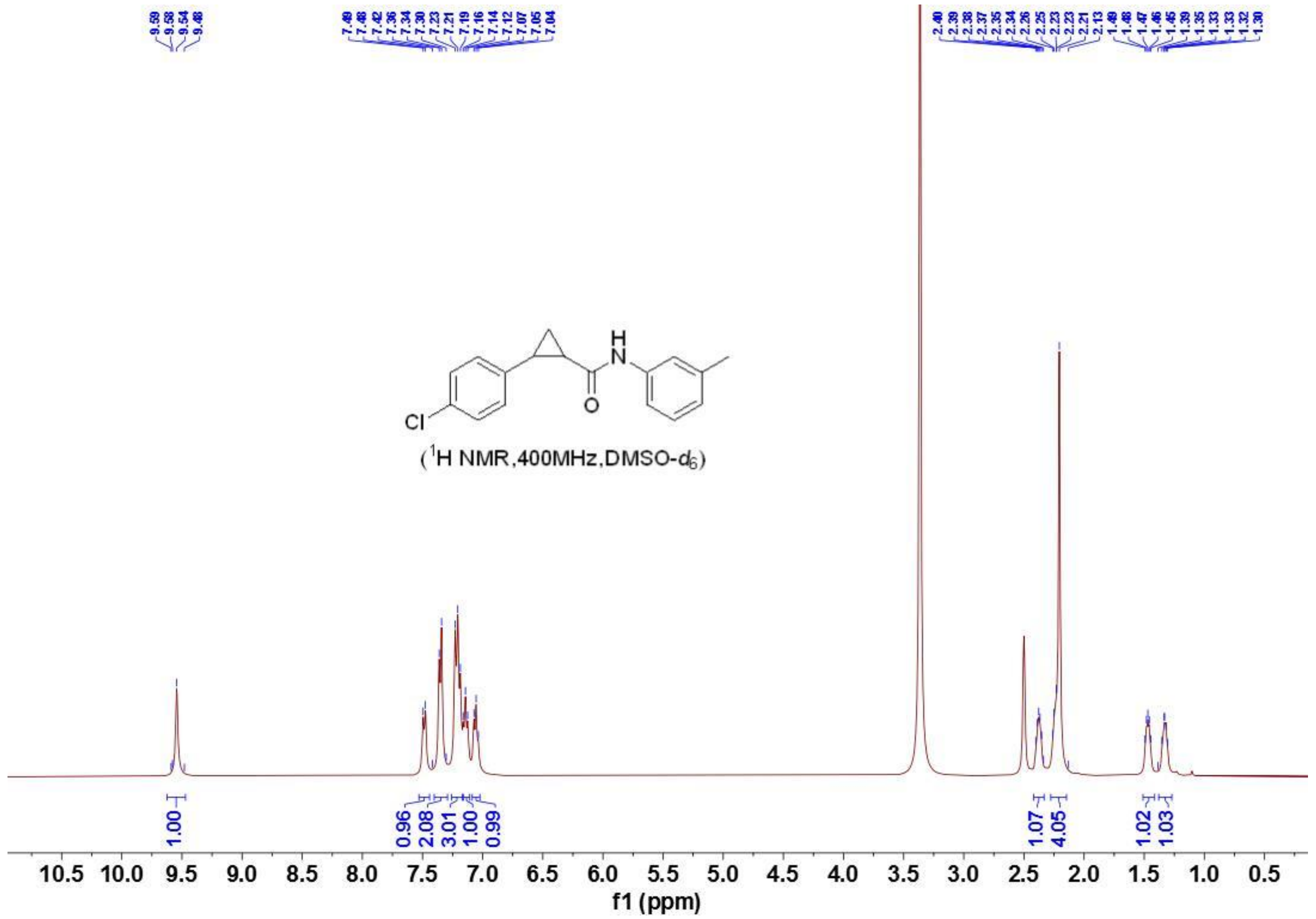

^{1}H NMR of compound **F50**

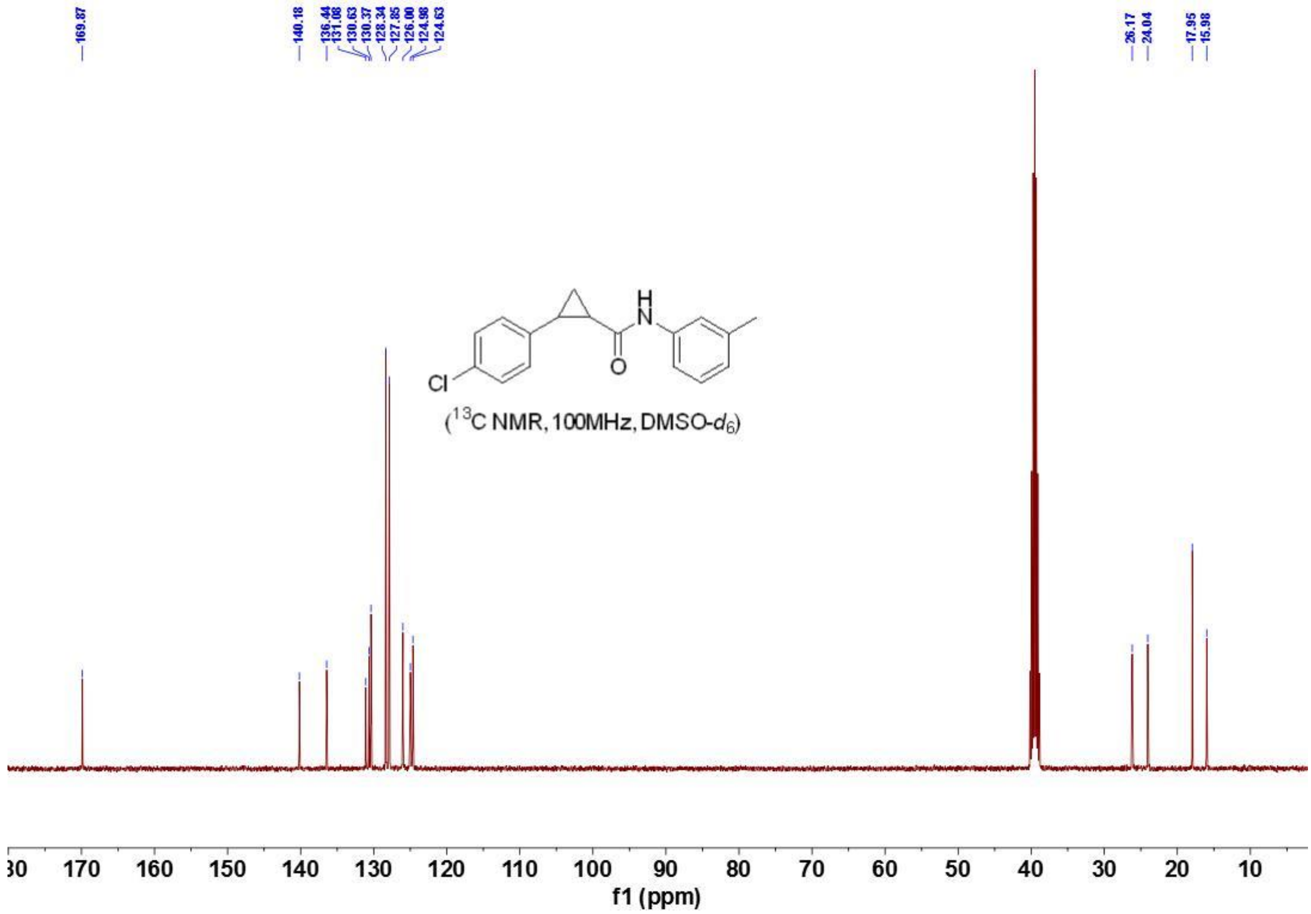

^{13}C NMR of compound **F50**

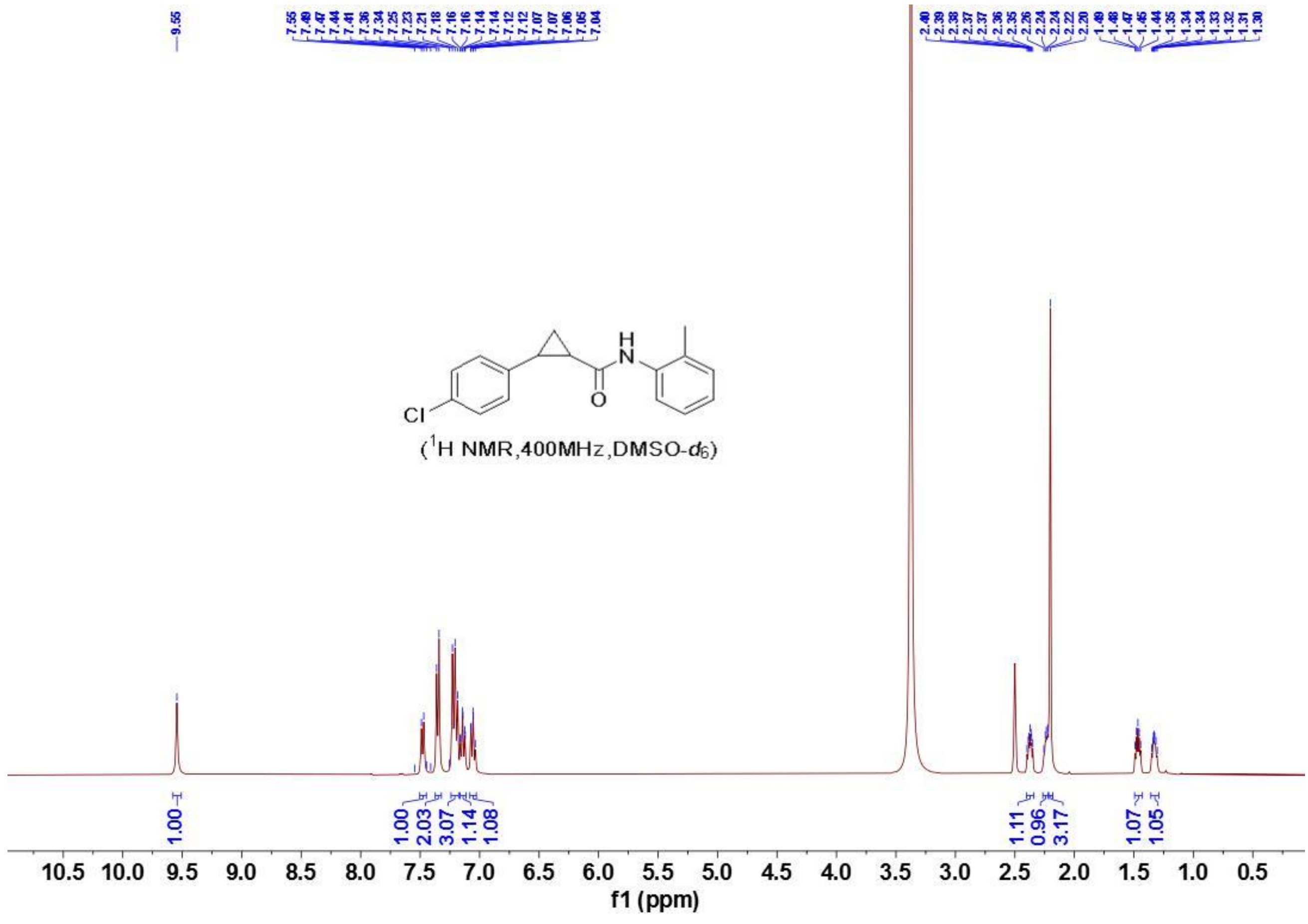

^{1}H NMR of compound **F51**

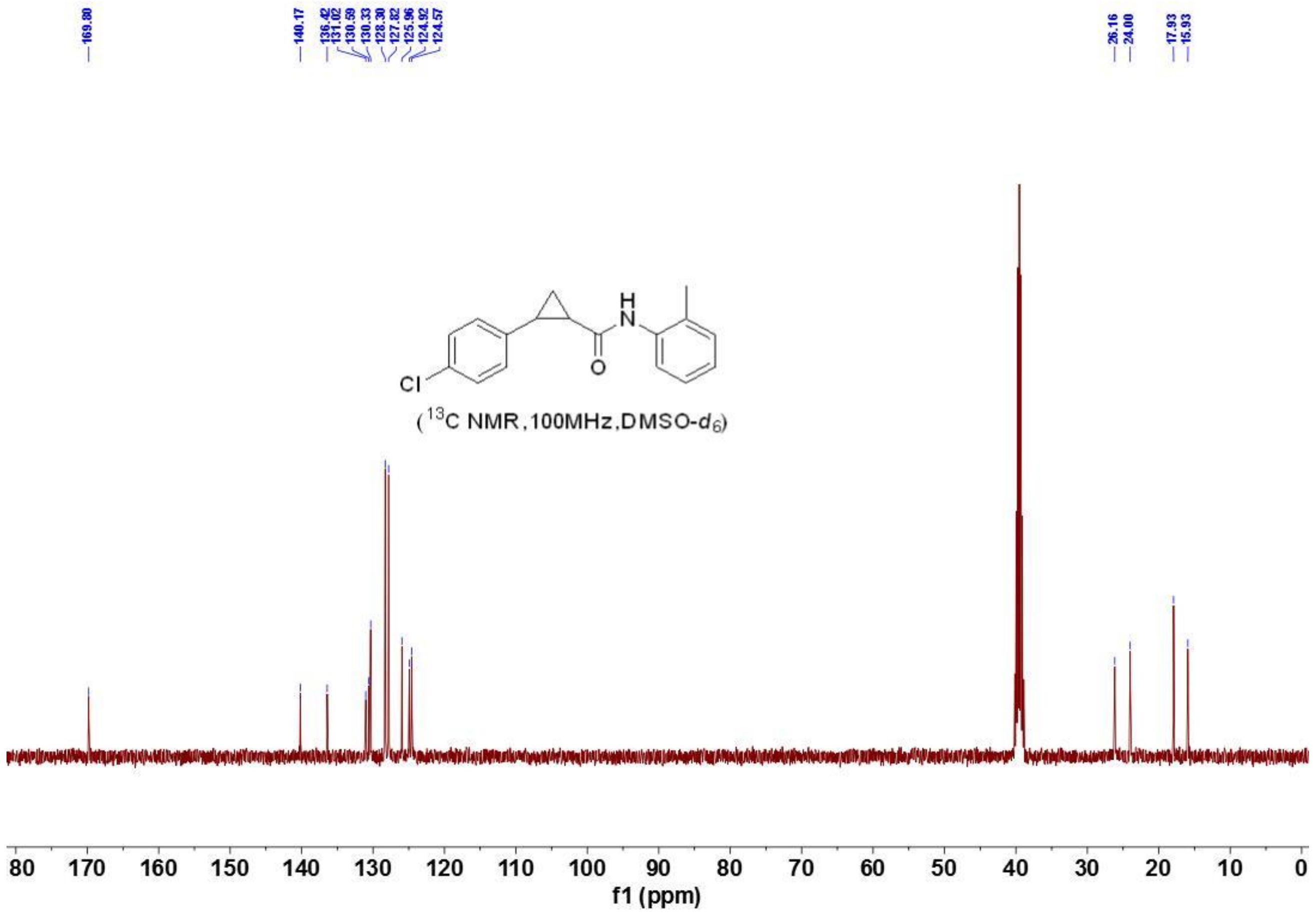

^{13}C NMR of compound **F51**

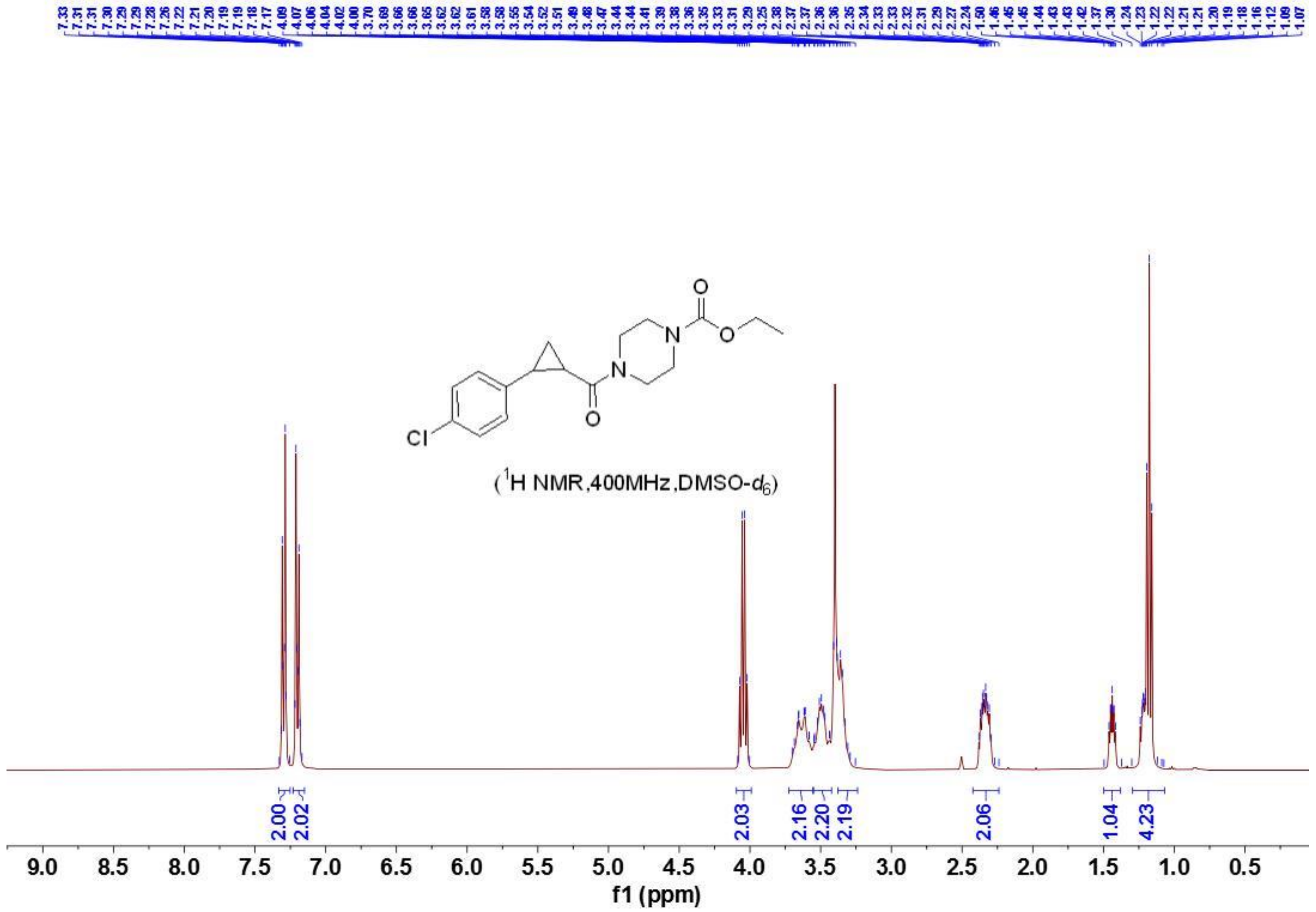

^{1}H NMR of compound **F52**

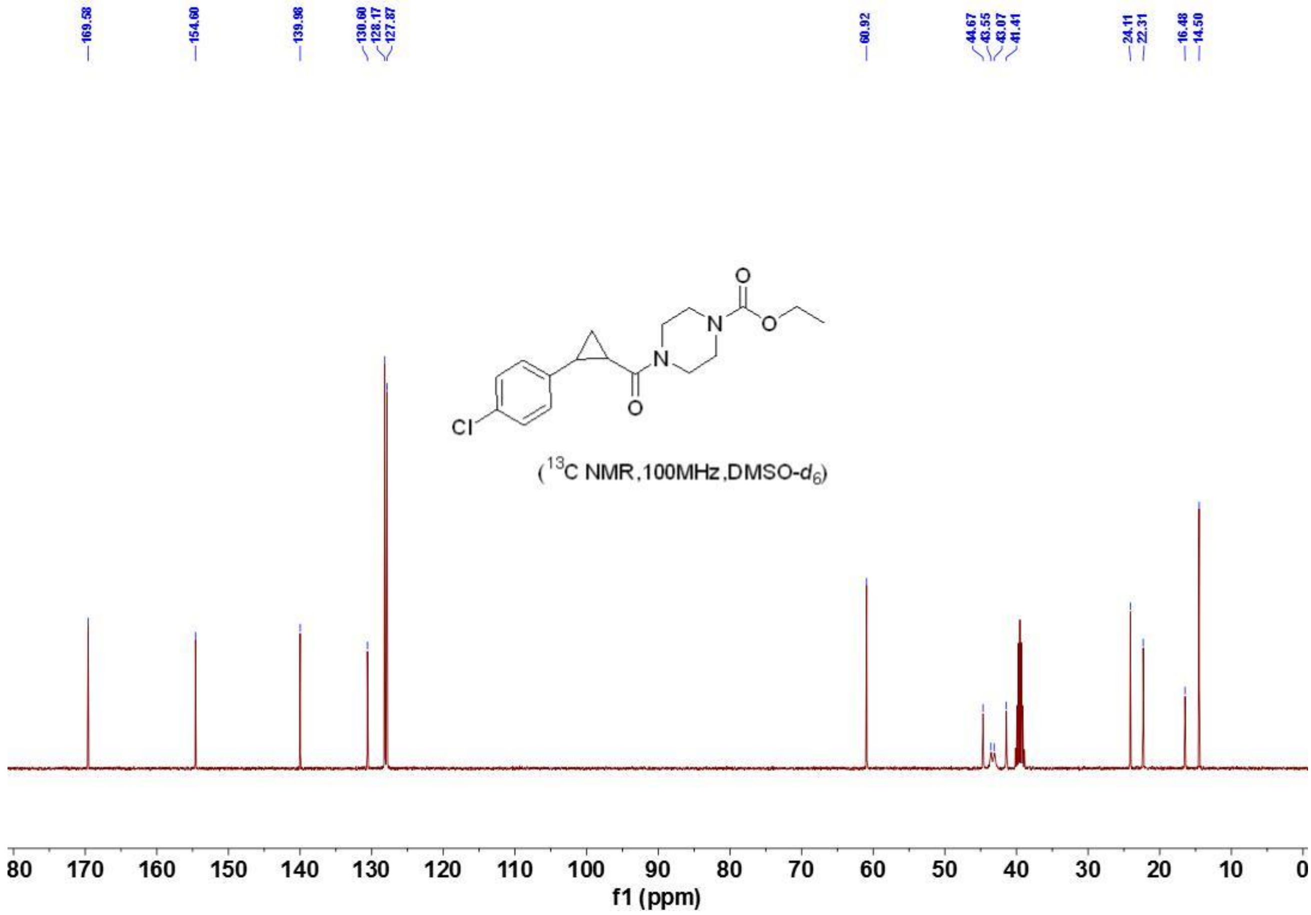

^{13}C NMR of compound **F52**

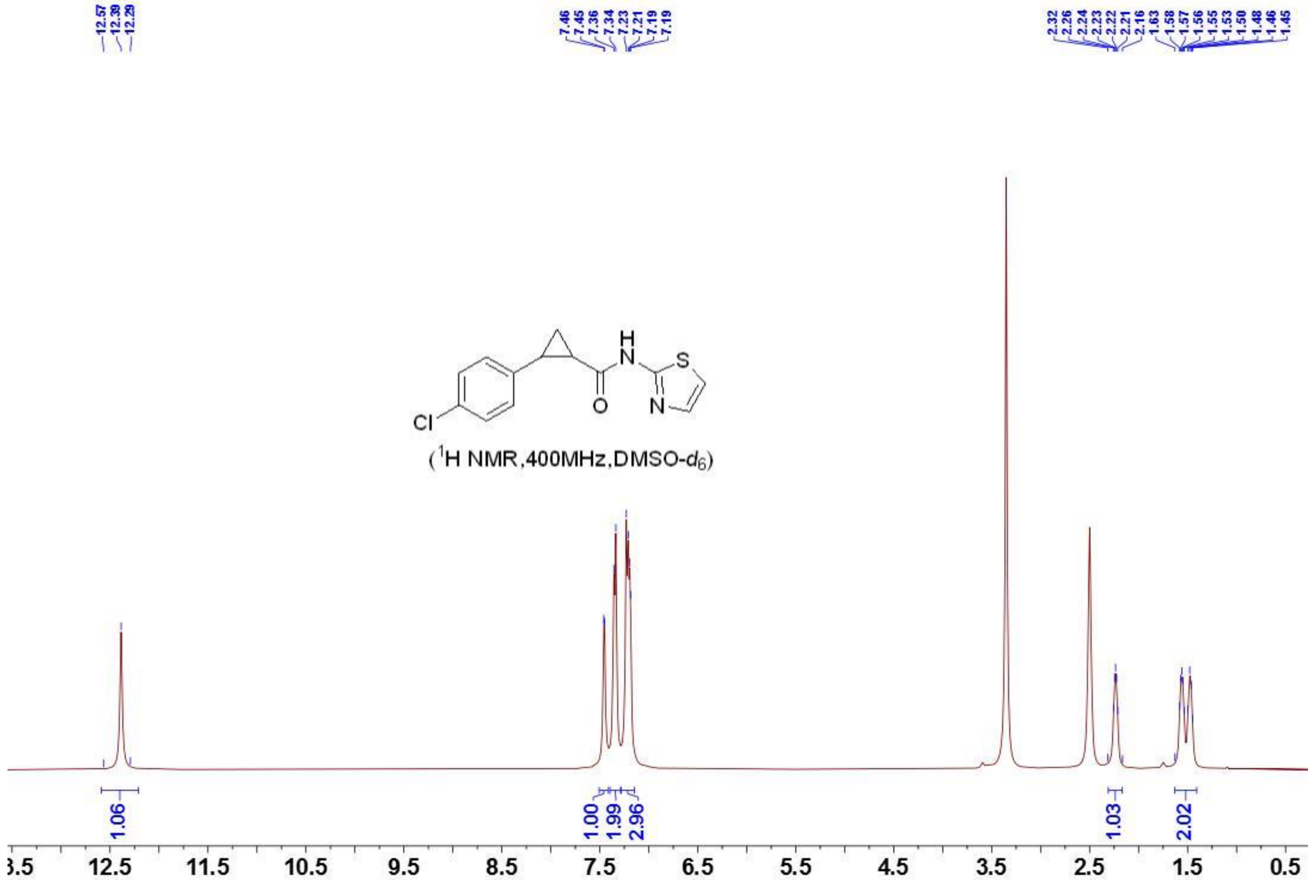

^{1}H NMR of compound **F53**

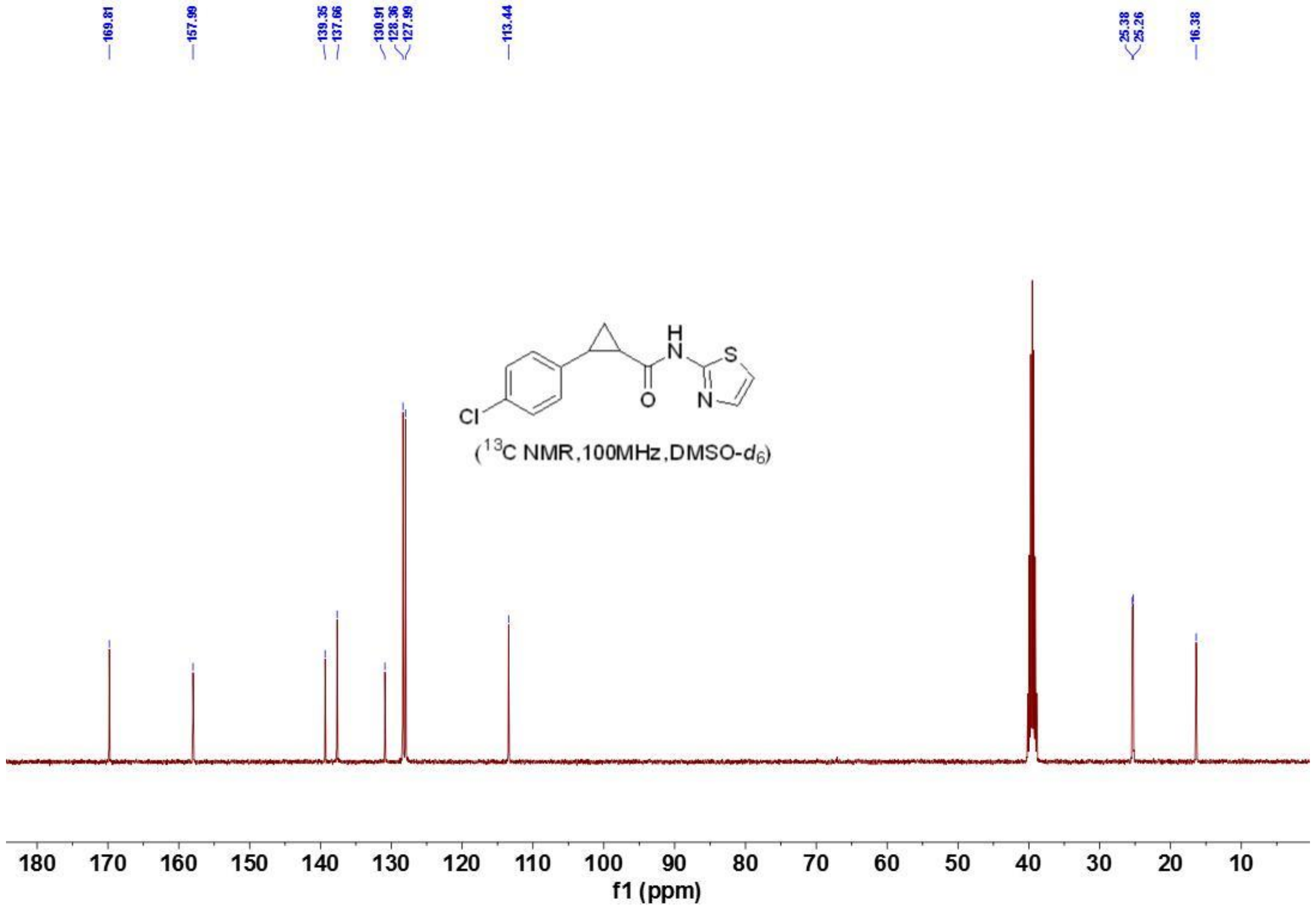

^{13}C NMR of compound **F53**

2. HRMS of all compounds

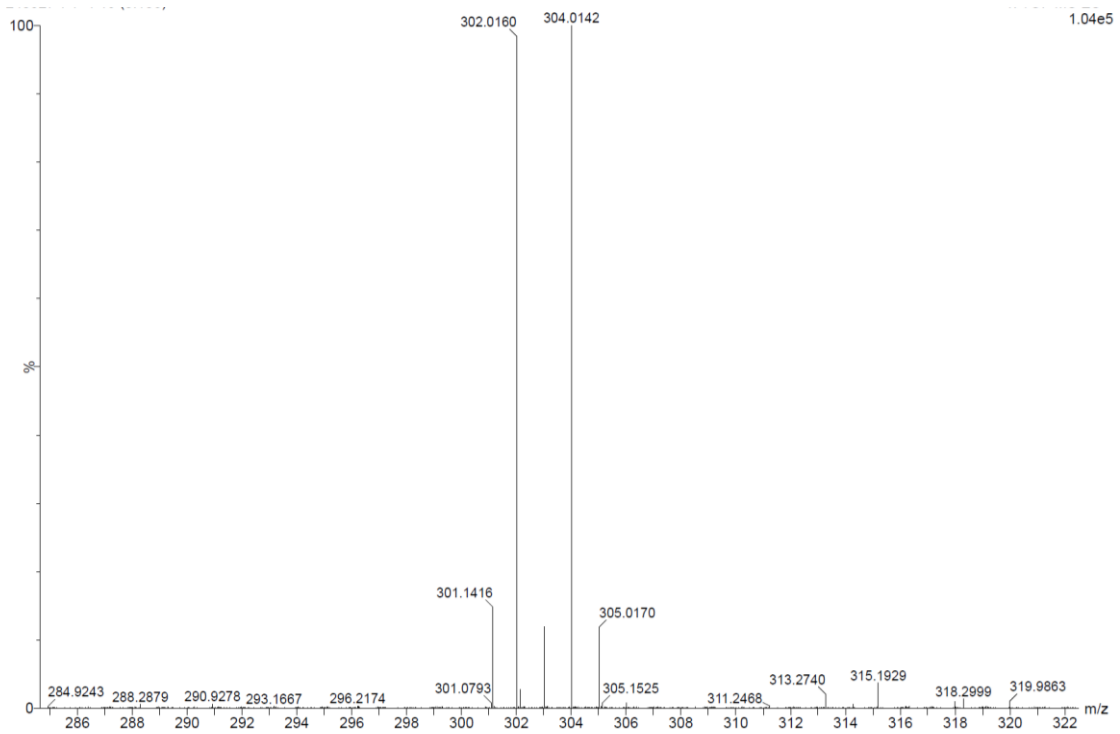

HRMS spectra of **F1**

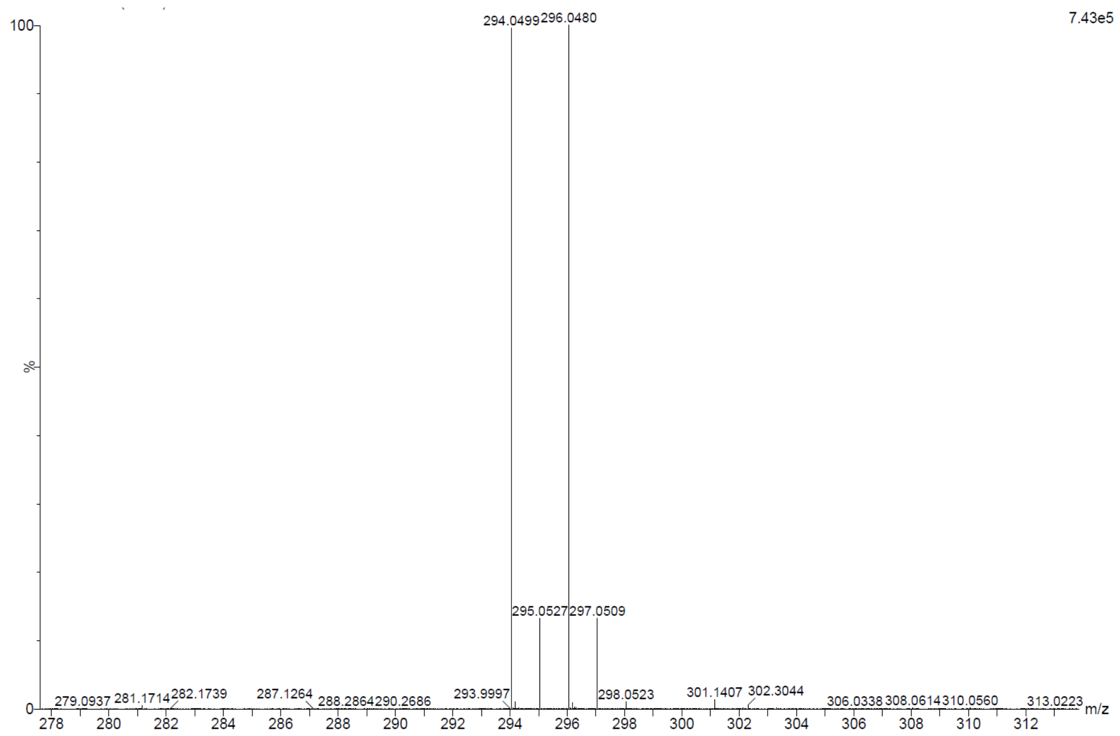

HRMS spectra of **F2**

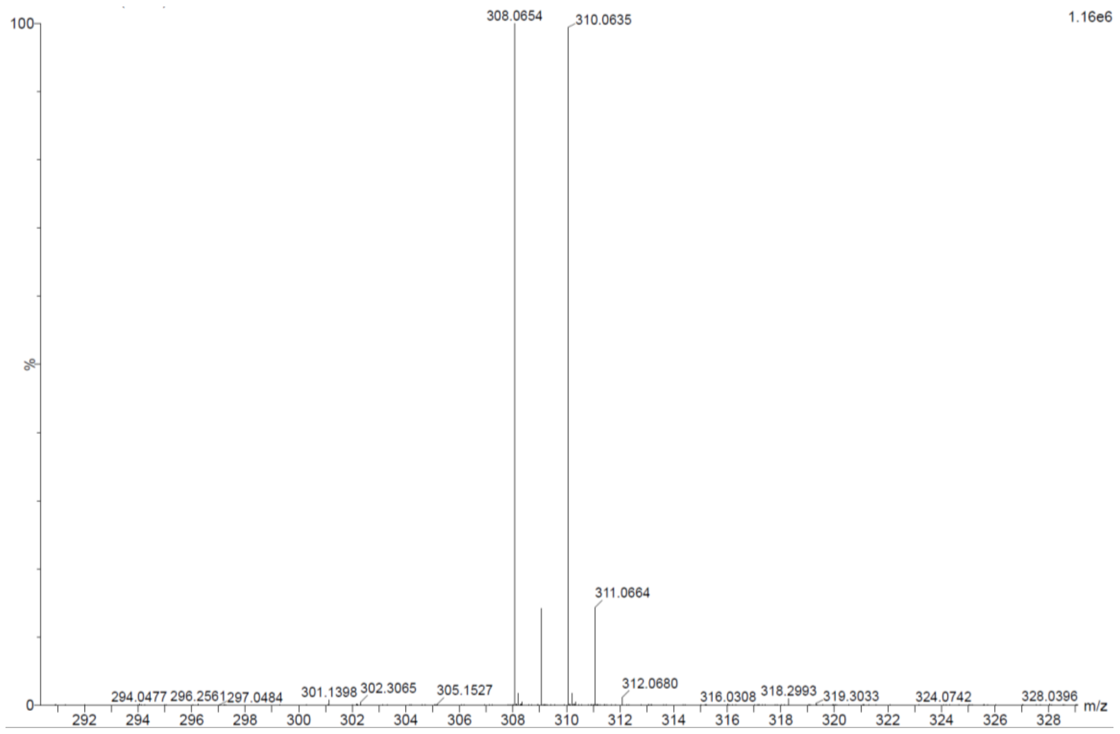

HRMS spectra of **F3**

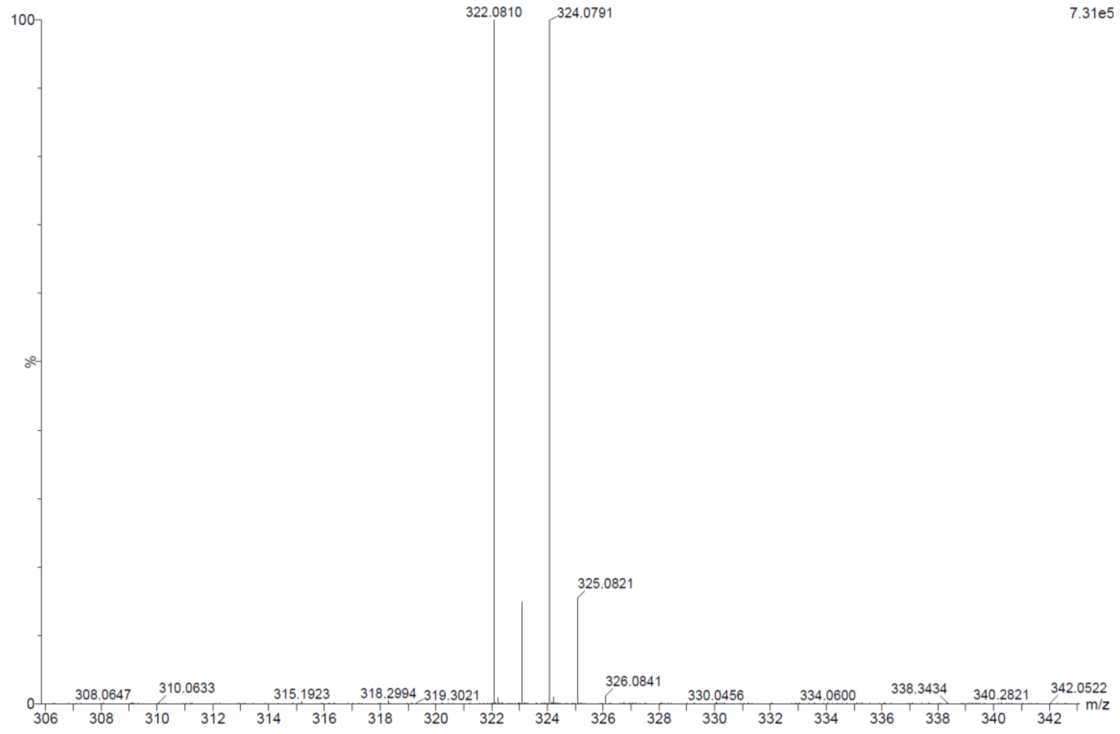

HRMS spectra of **F4**

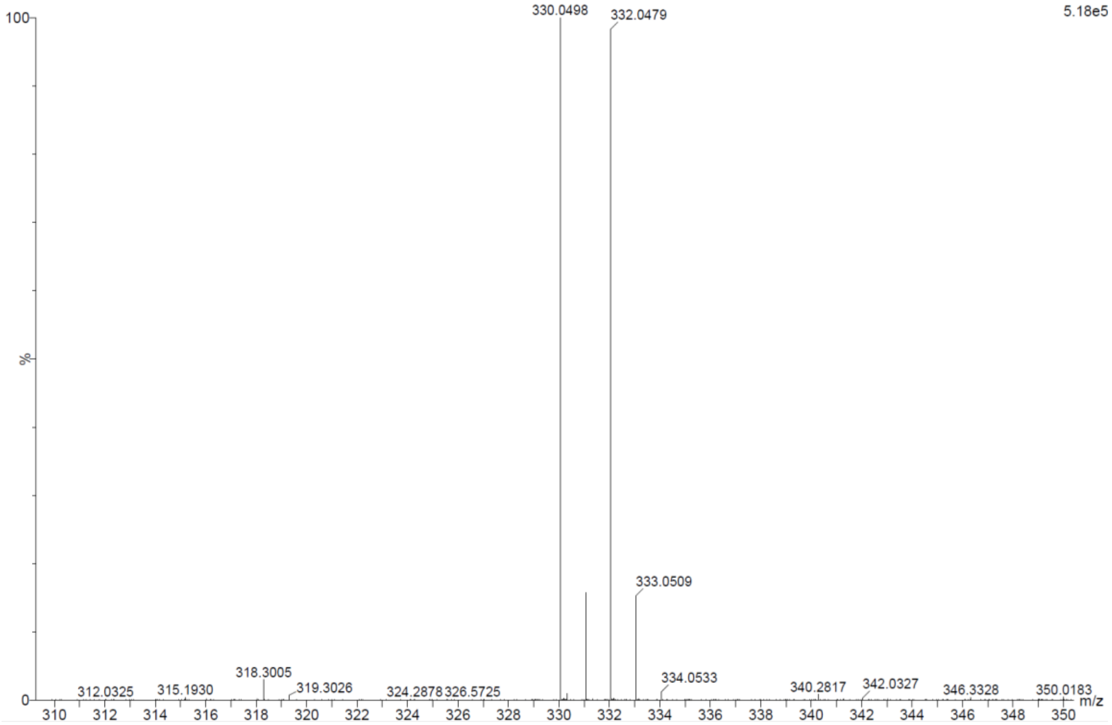

HRMS spectra of **F5**

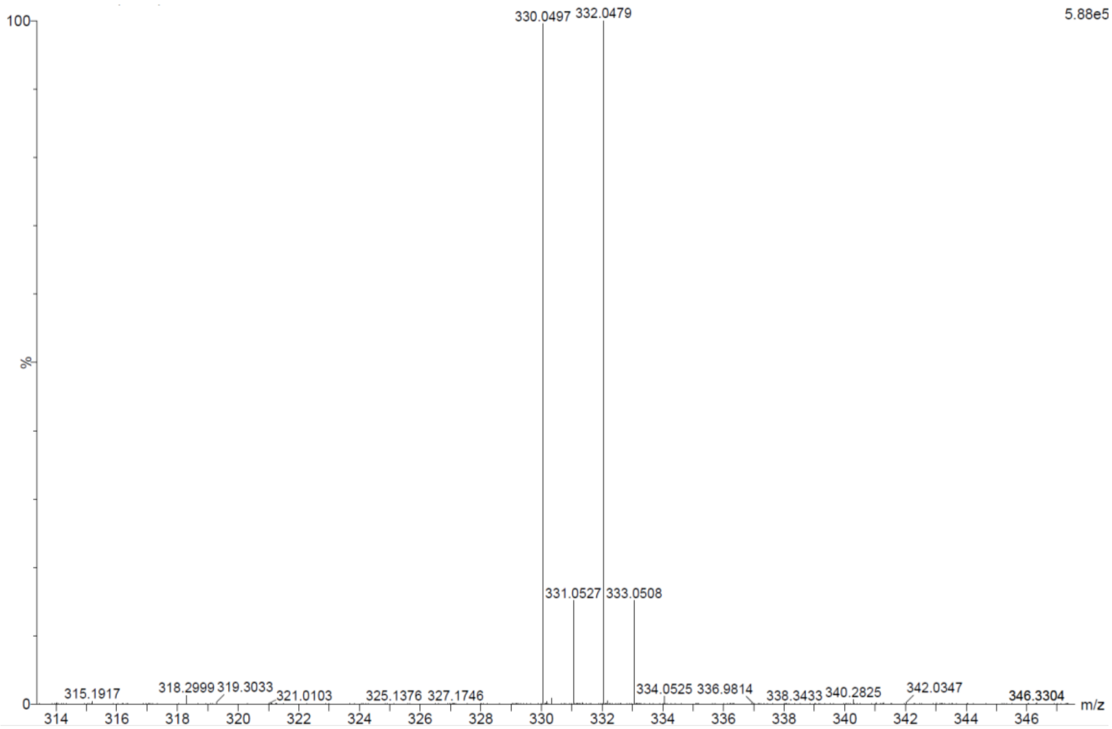

HRMS spectra of **F6**

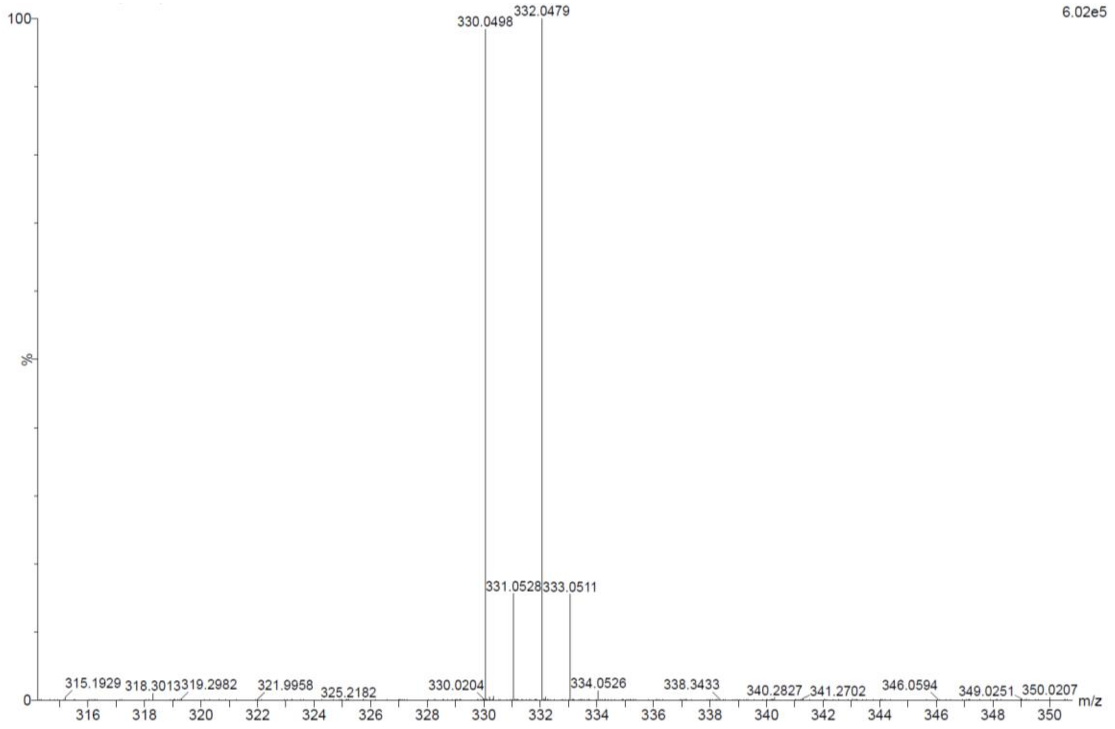

HRMS spectra of **F7**

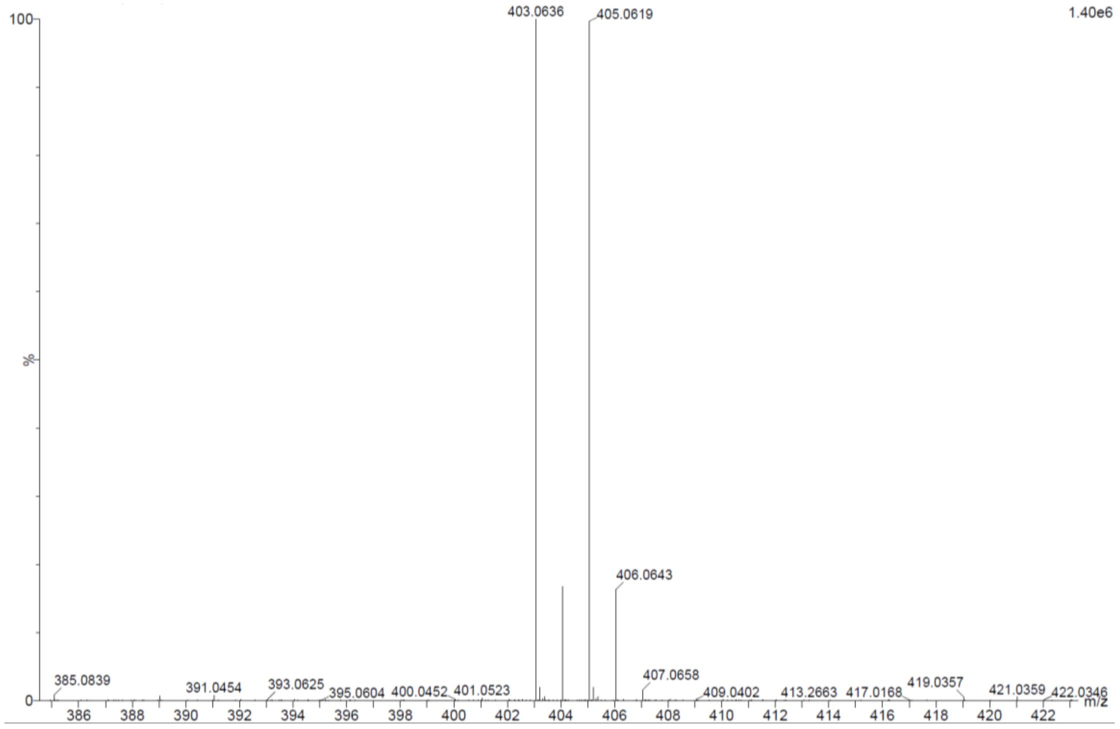

HRMS spectra of **F8**

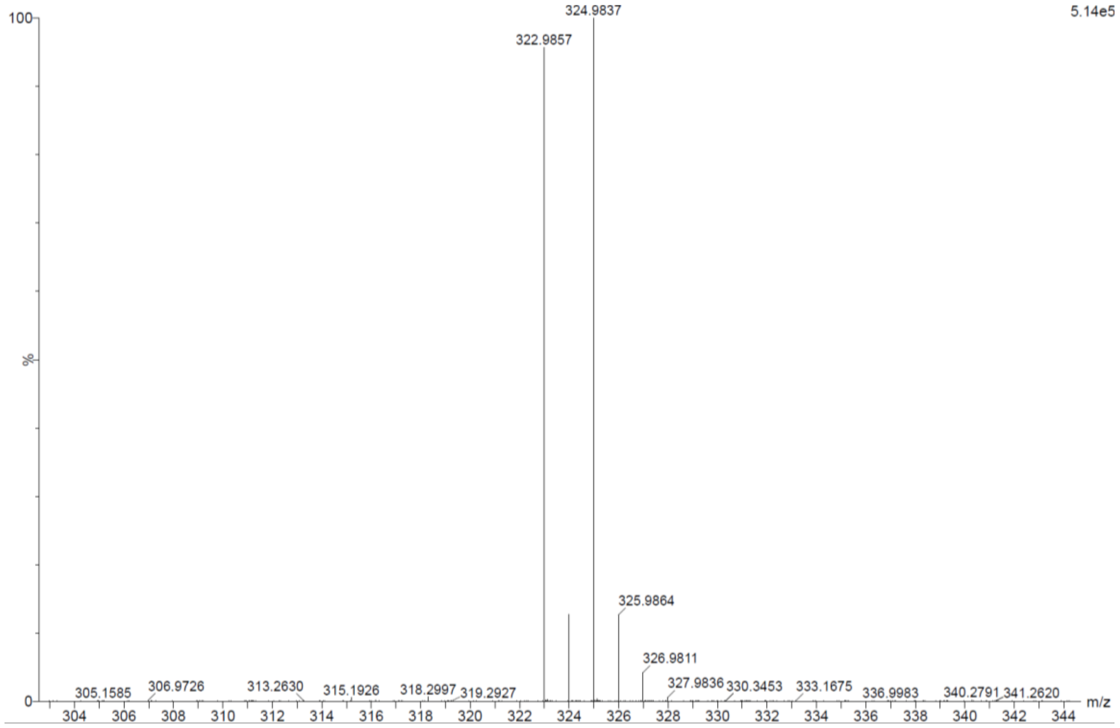

HRMS spectra of **F9**

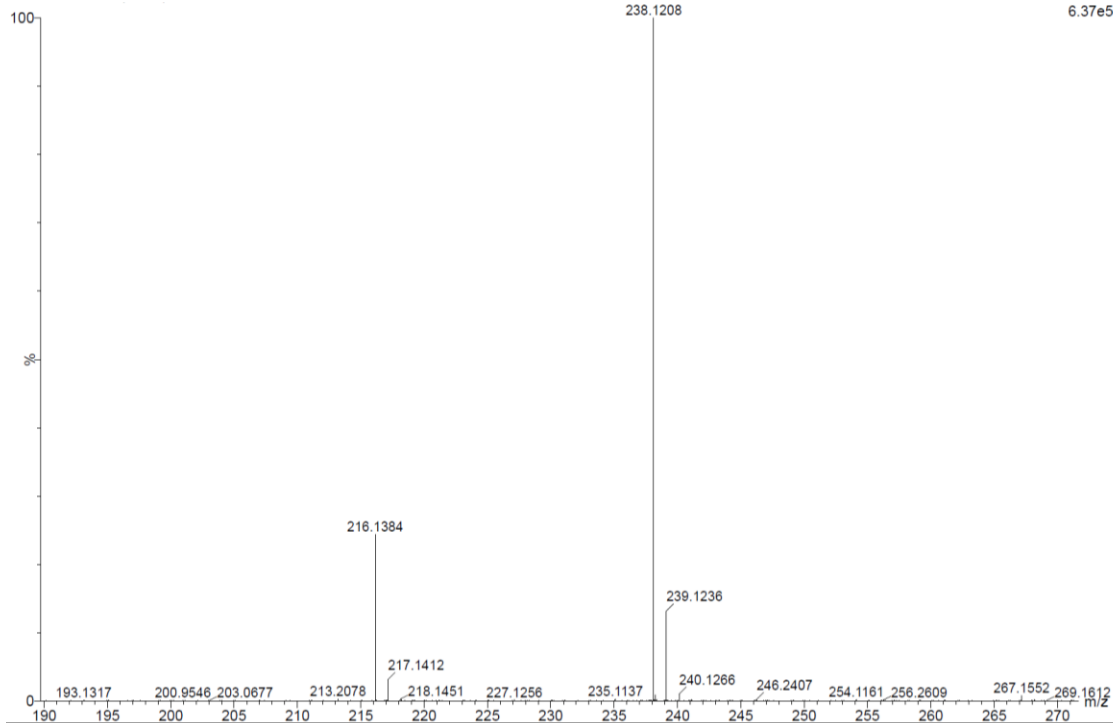

HRMS spectra of **F10**

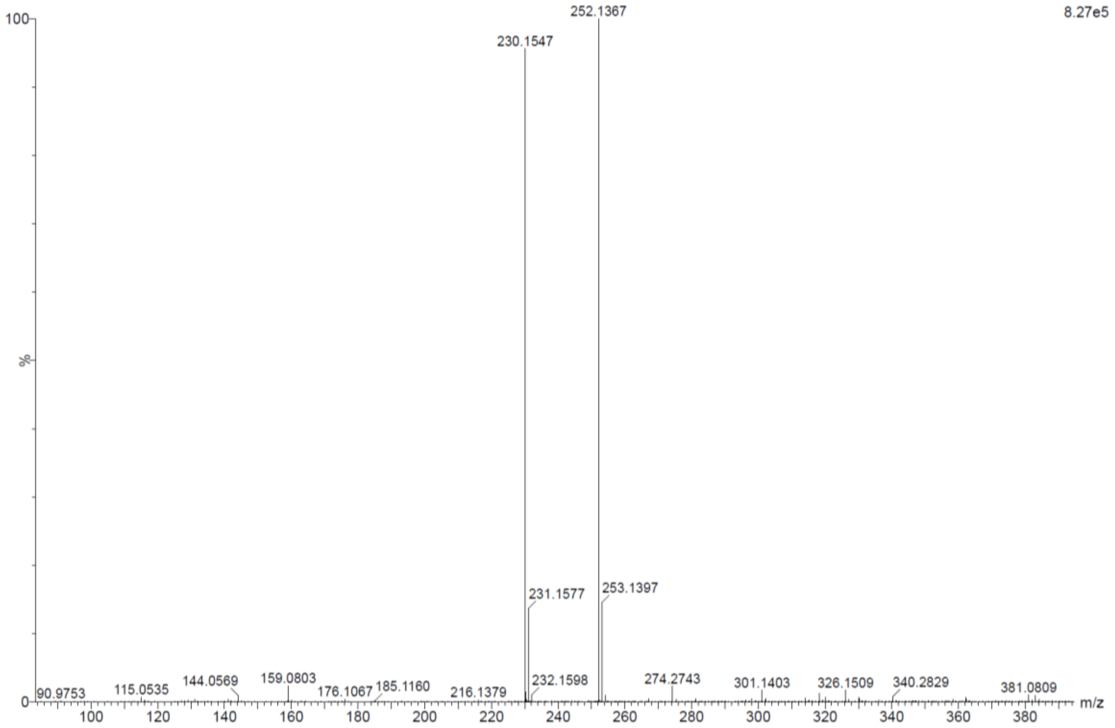

HRMS spectra of **F11**

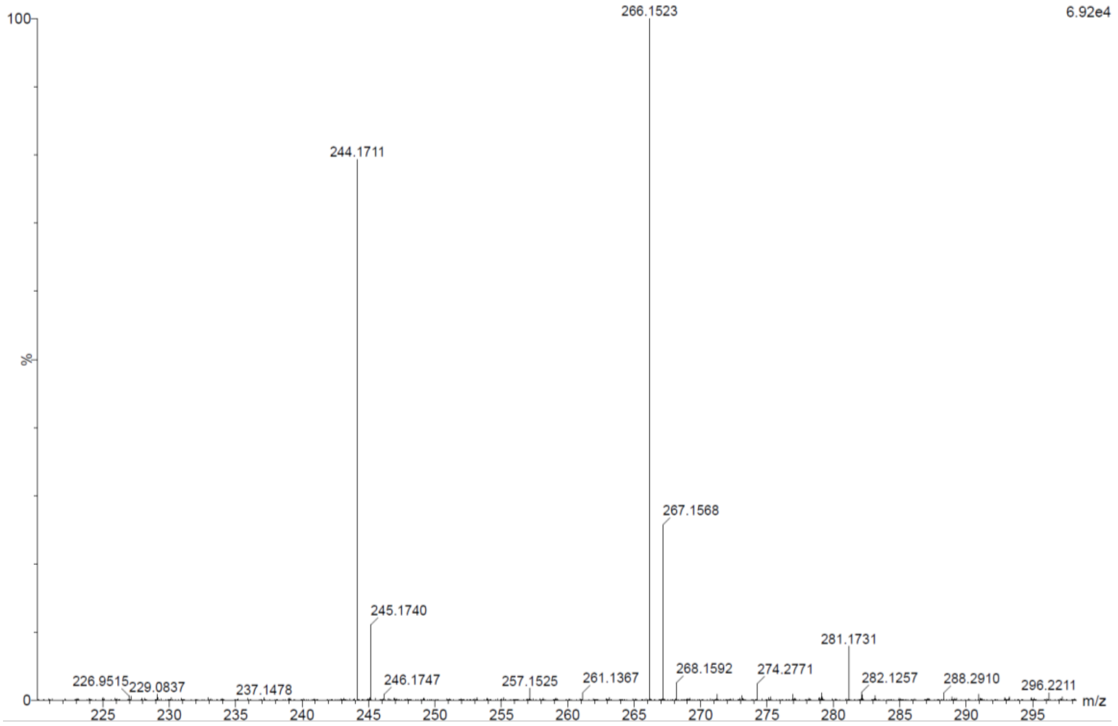

HRMS spectra of **F12**

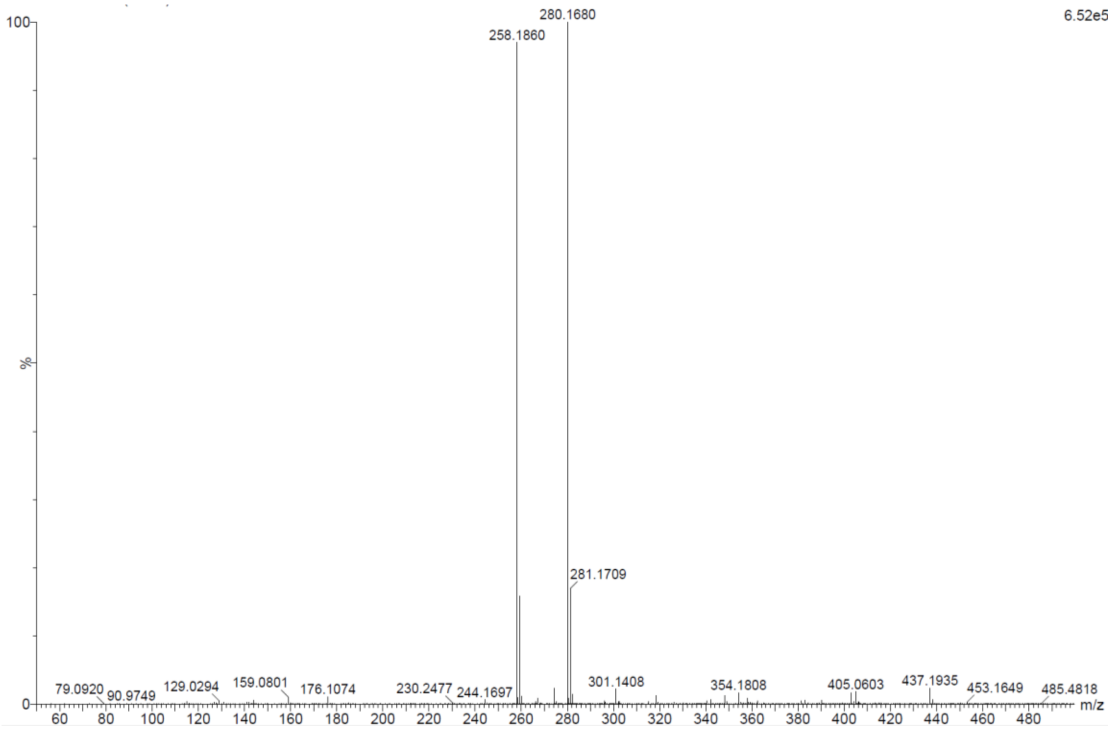

HRMS spectra of **F13**

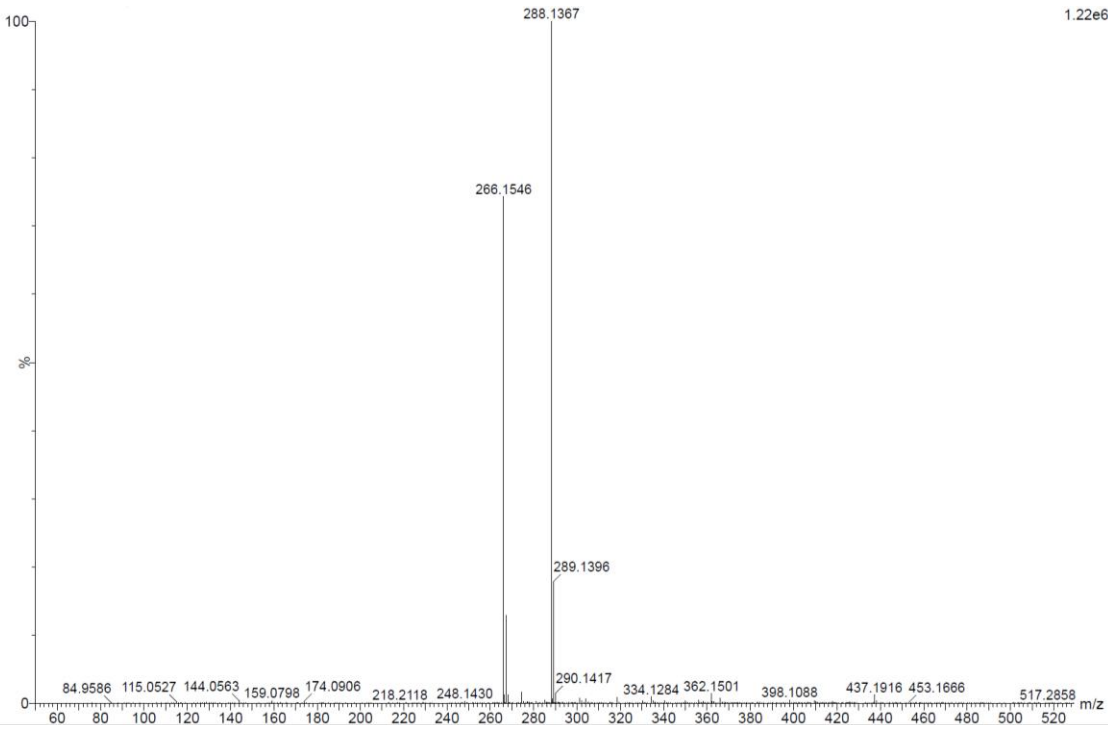

HRMS spectra of **F14**

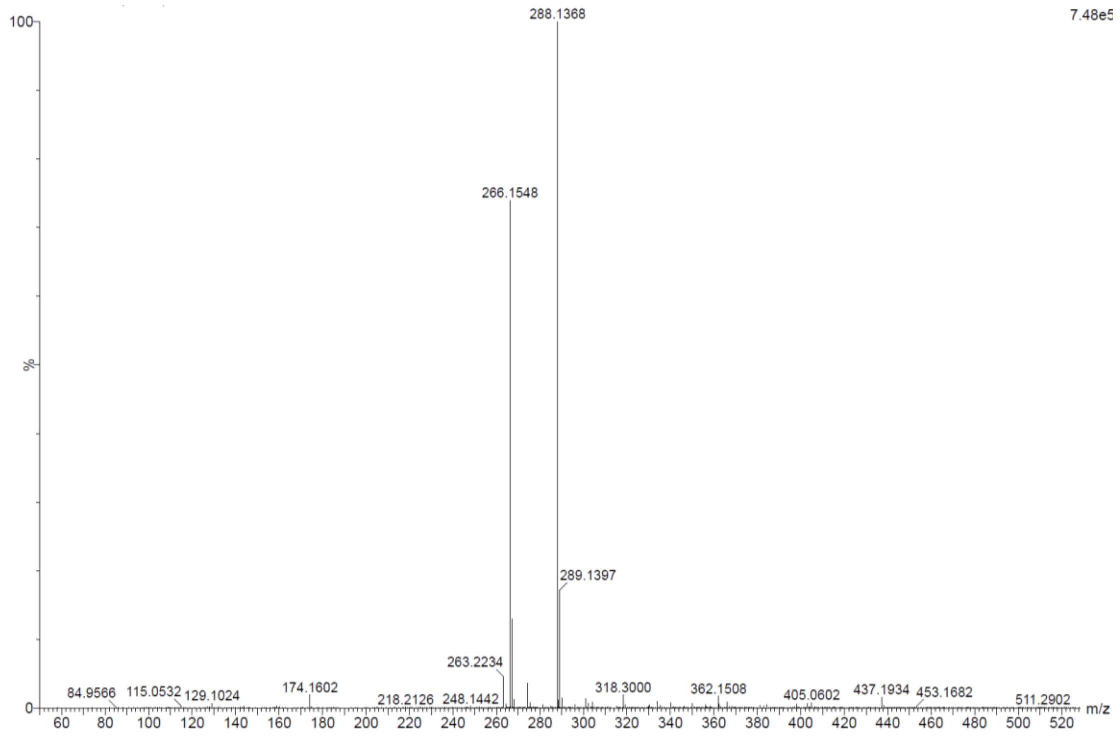

HRMS spectra of **F15**

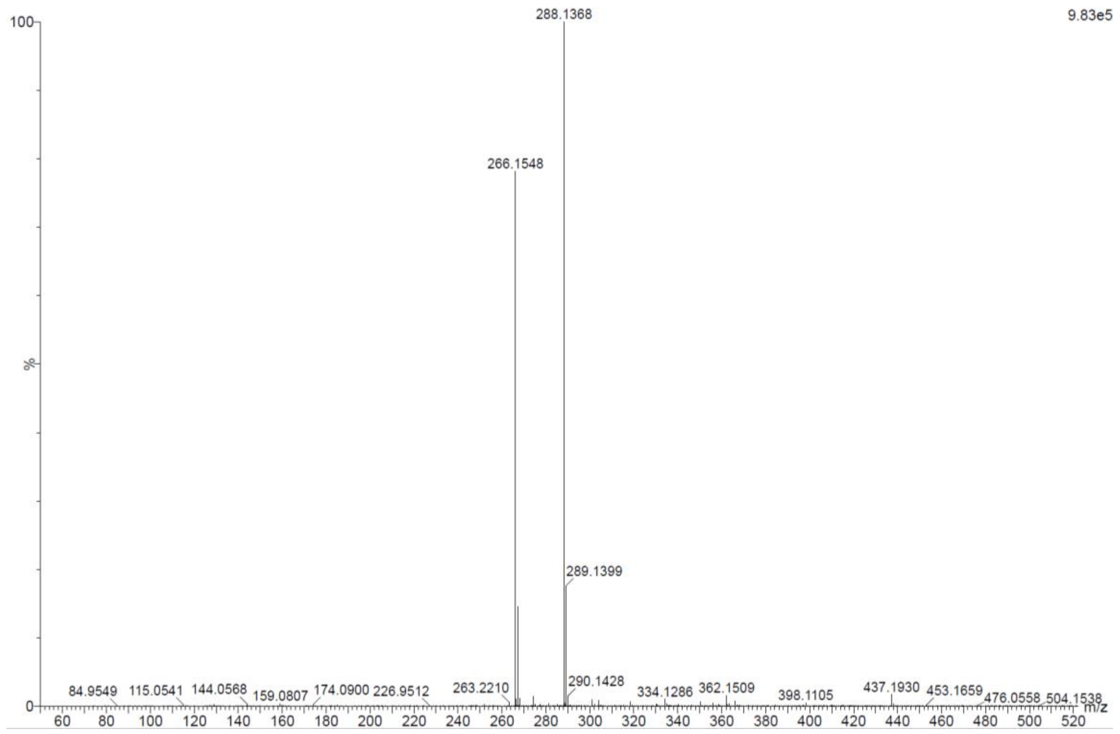

HRMS spectra of **F16**

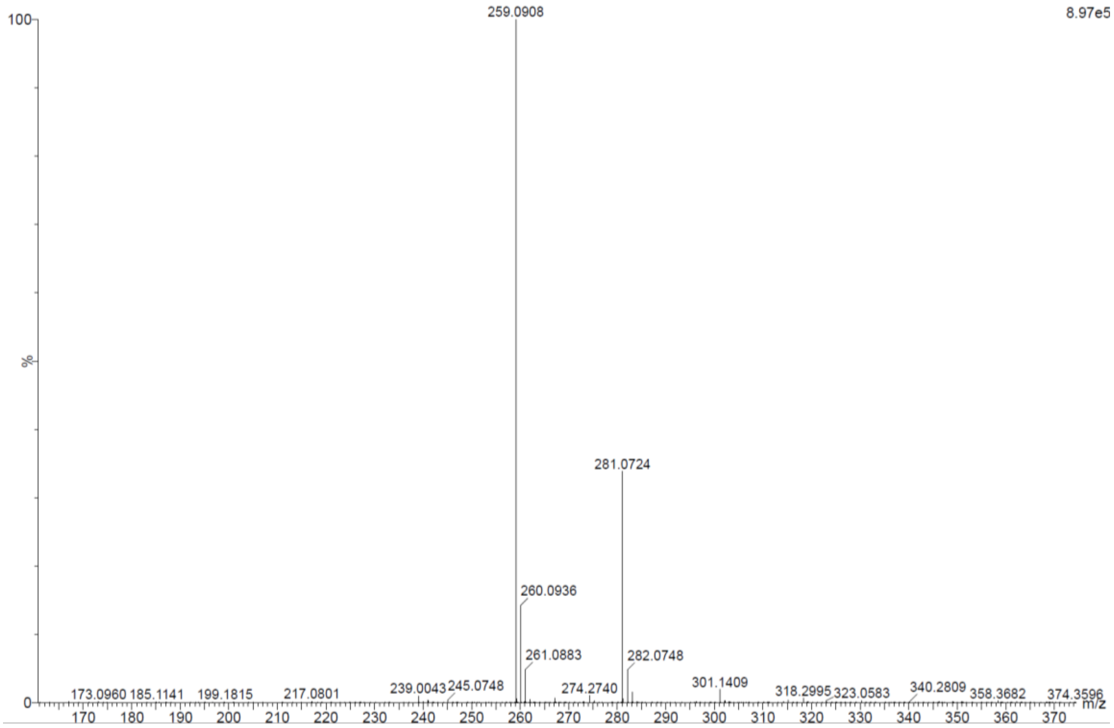

HRMS spectra of **F17**

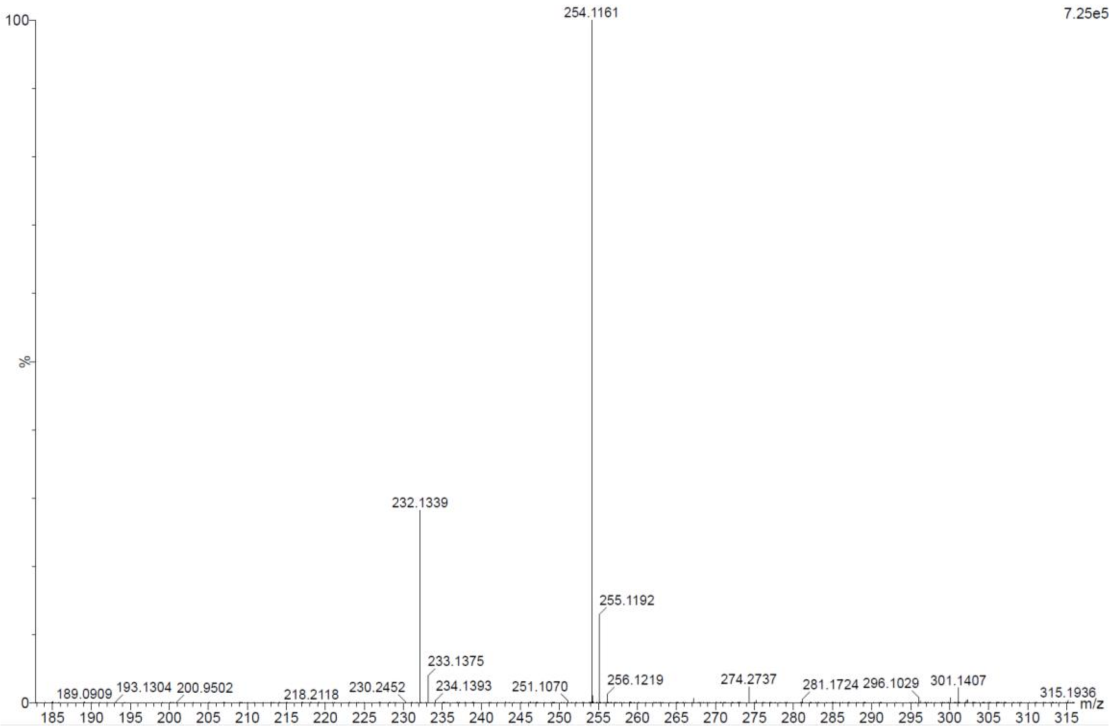

HRMS spectra of **F18**

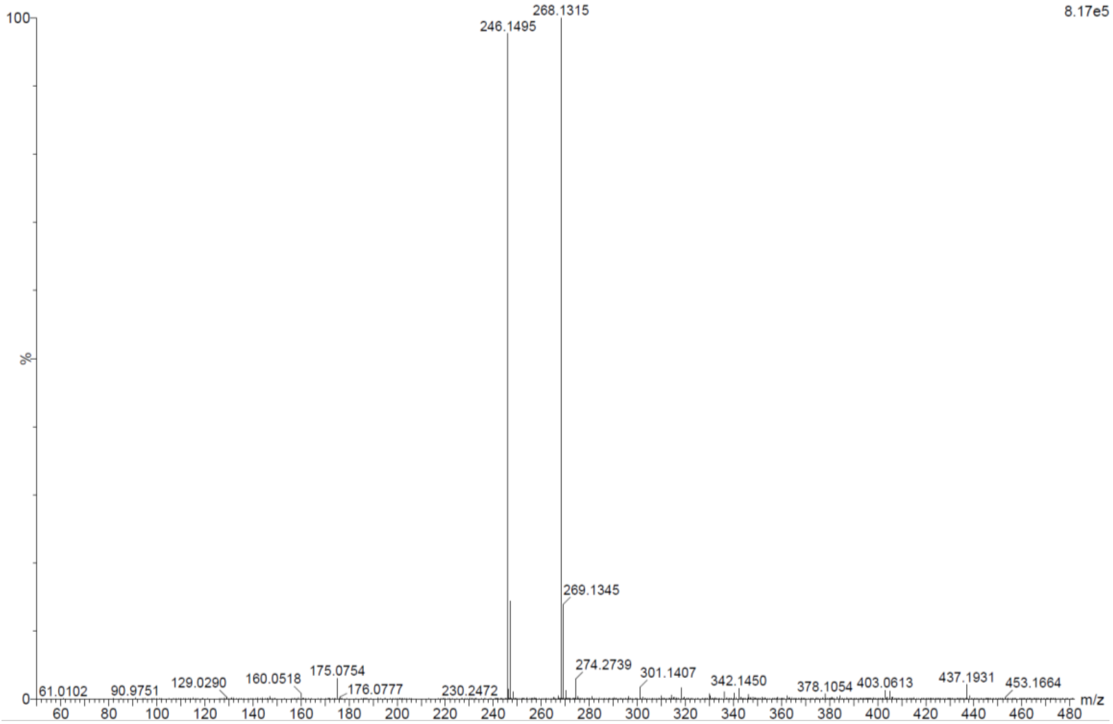

HRMS spectra of **F19**

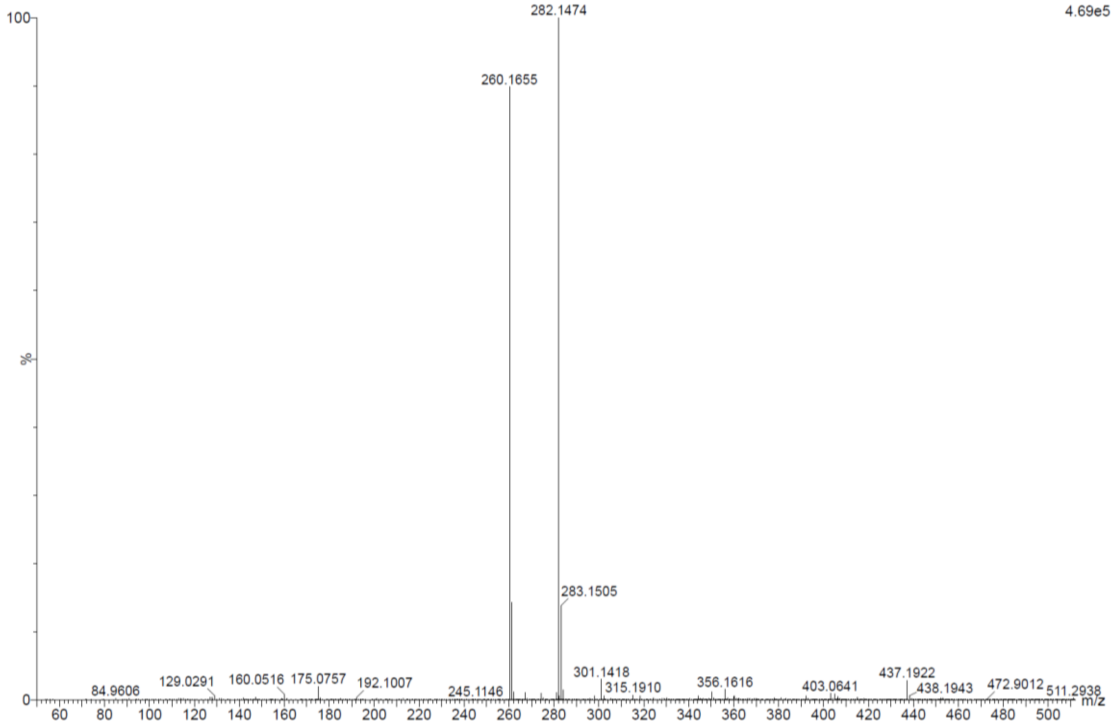

HRMS spectra of **F20**

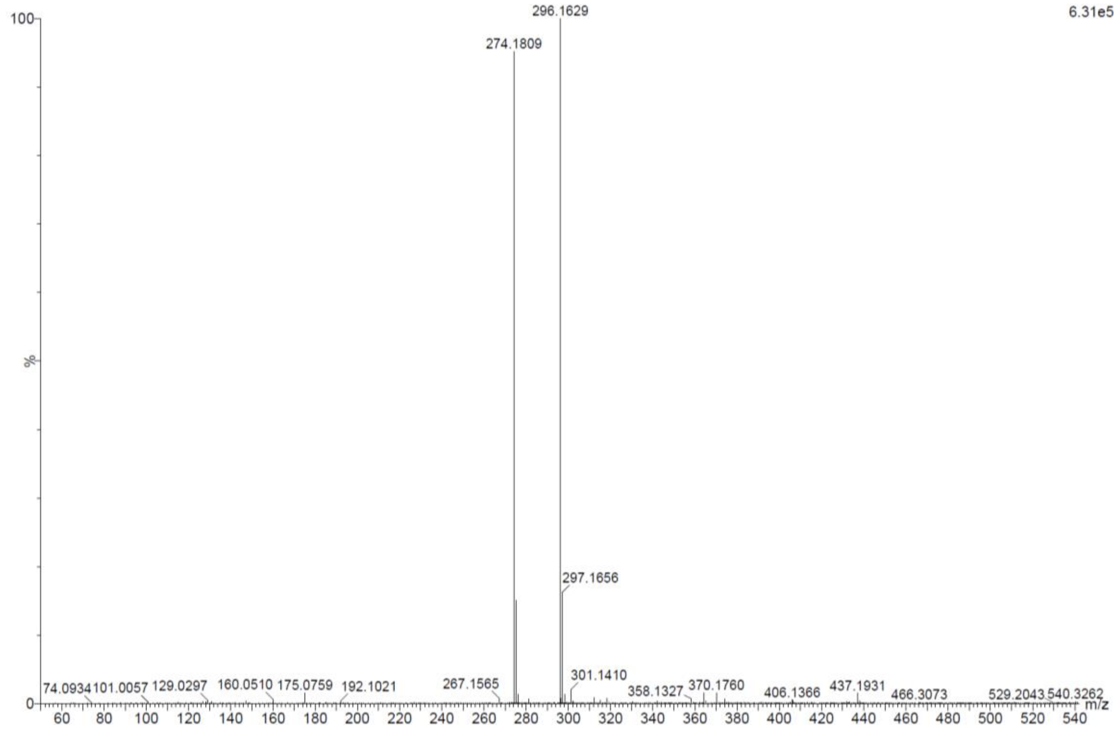

HRMS spectra of **F21**

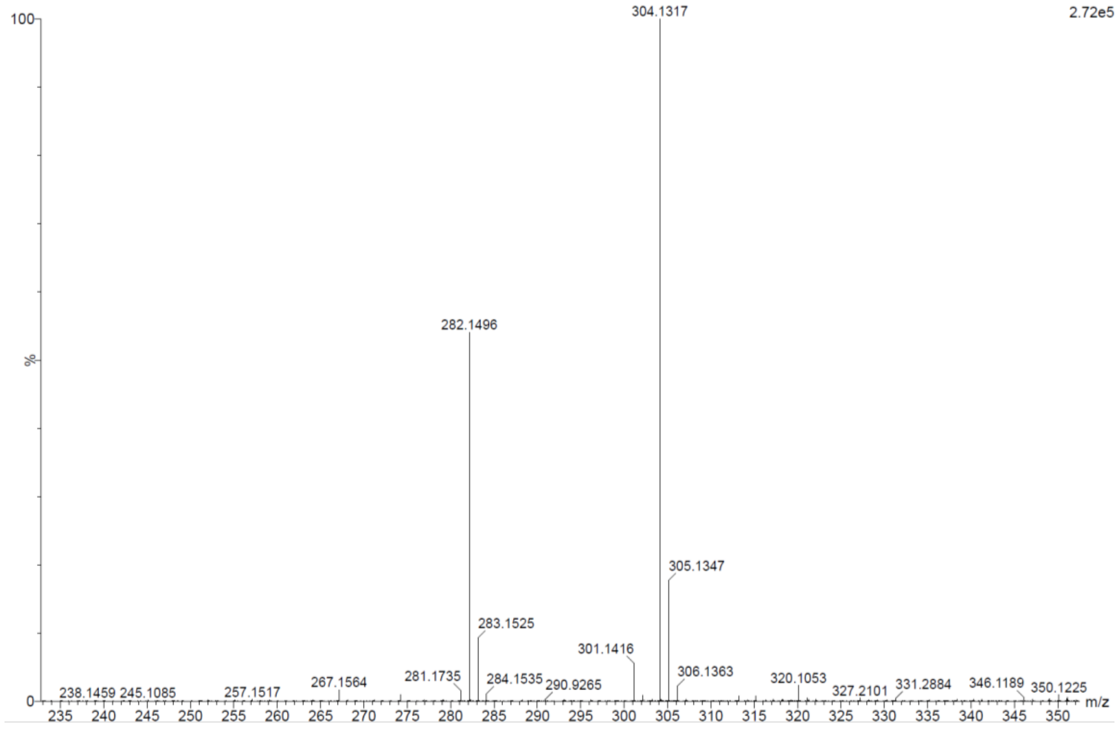

HRMS spectra of **F22**

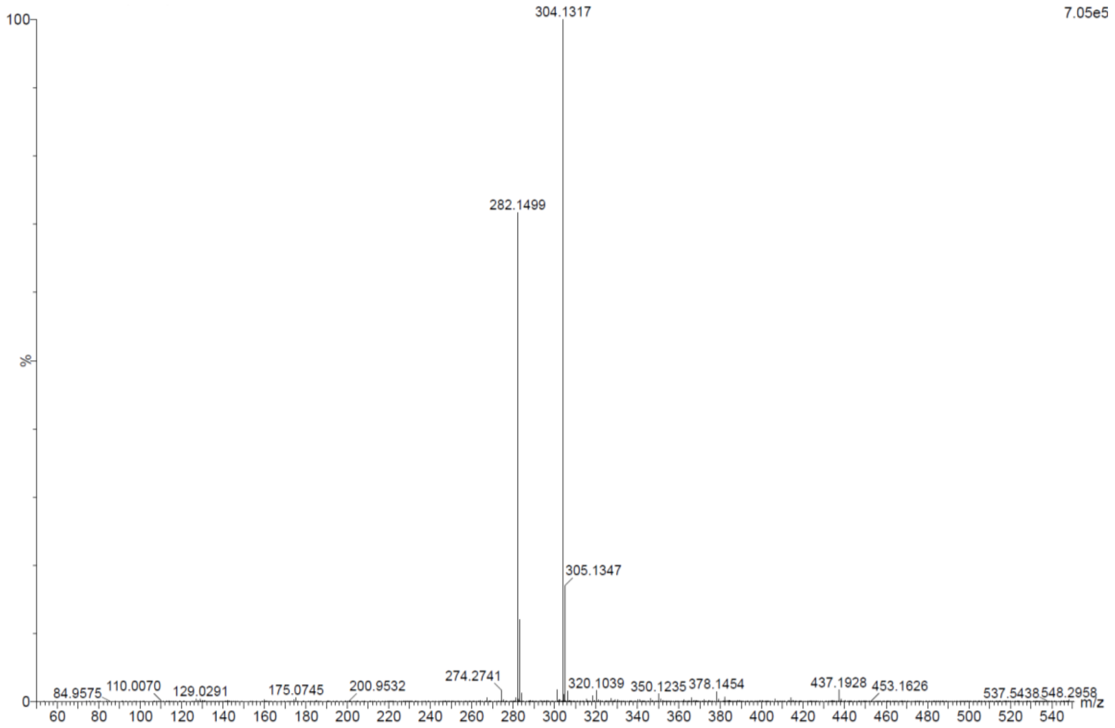

HRMS spectra of **F23**

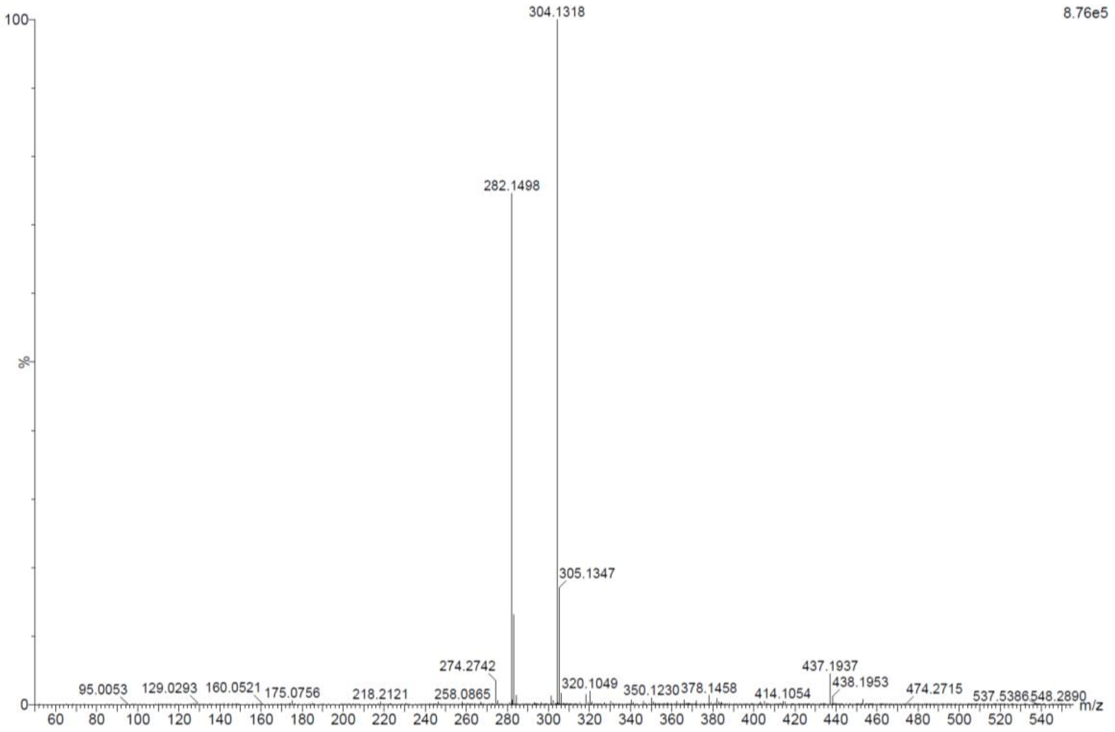

HRMS spectra of **F24**

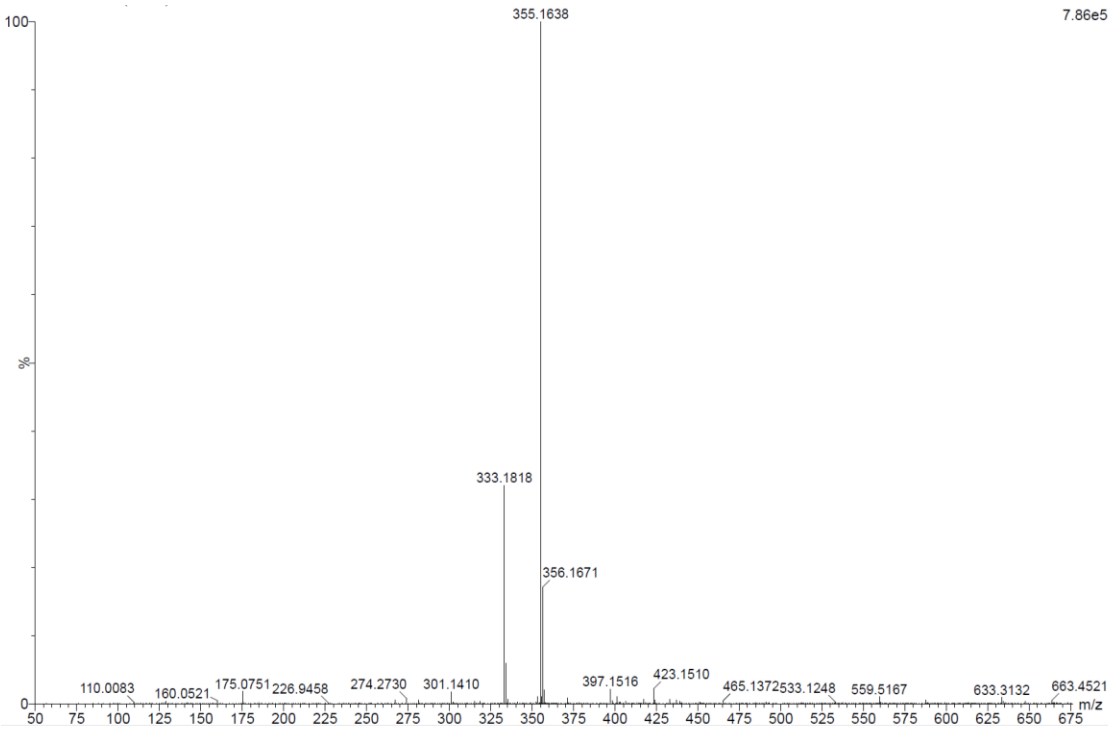

HRMS spectra of **F25**

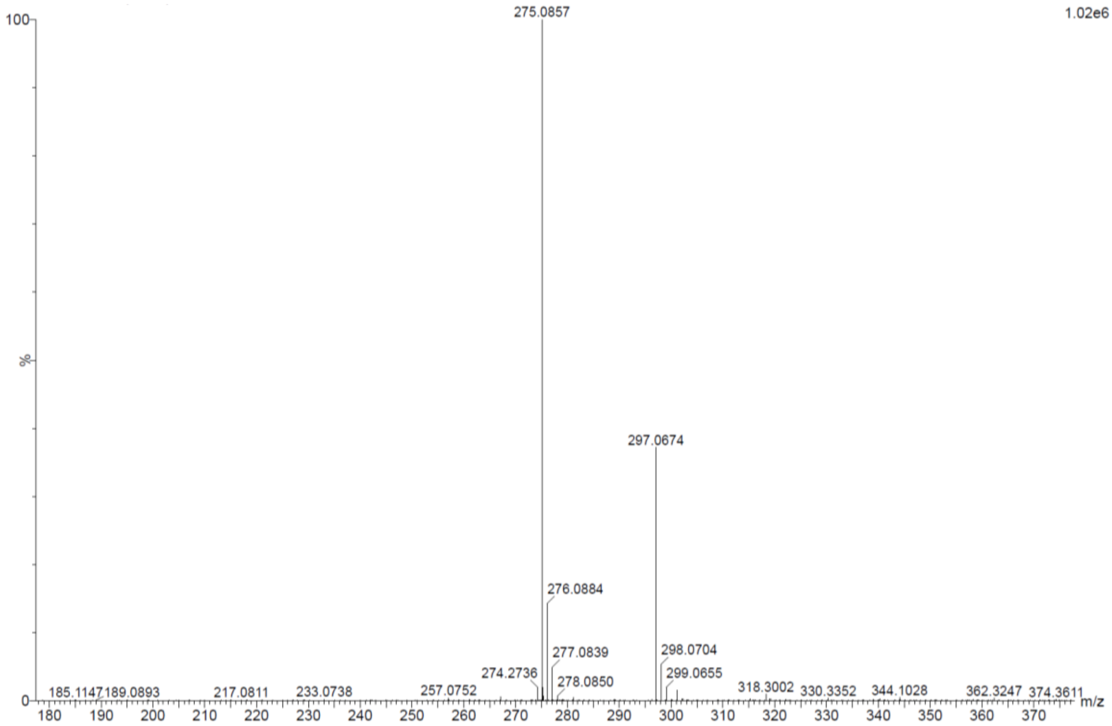

HRMS spectra of **F26**

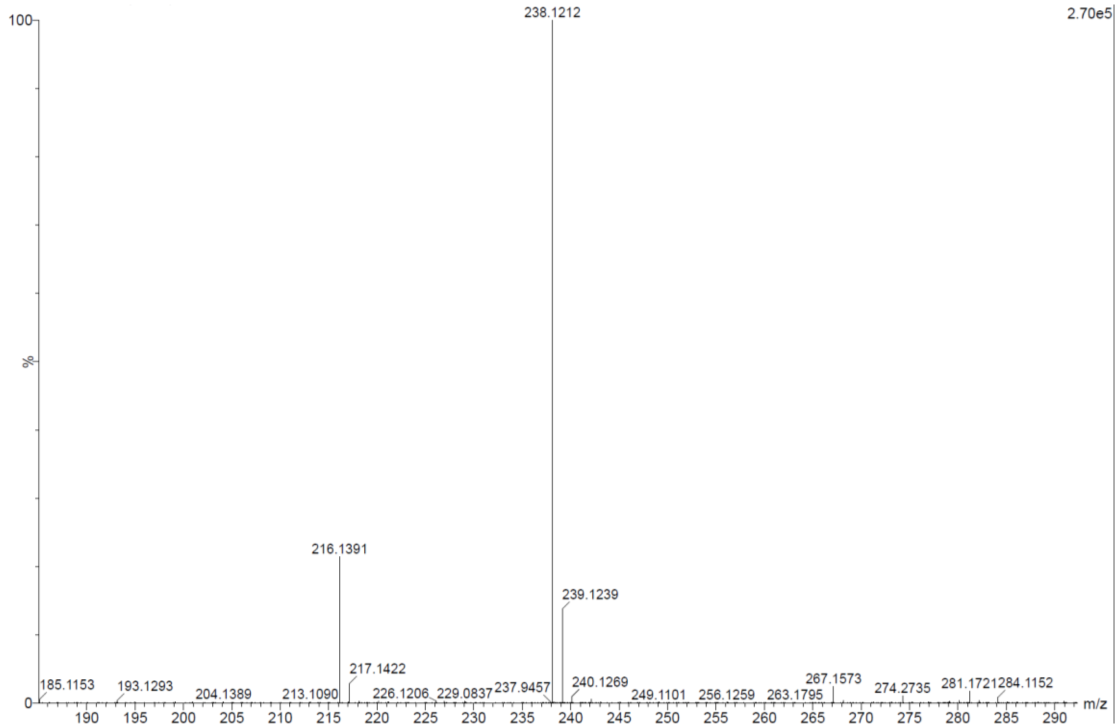

HRMS spectra of **F27**

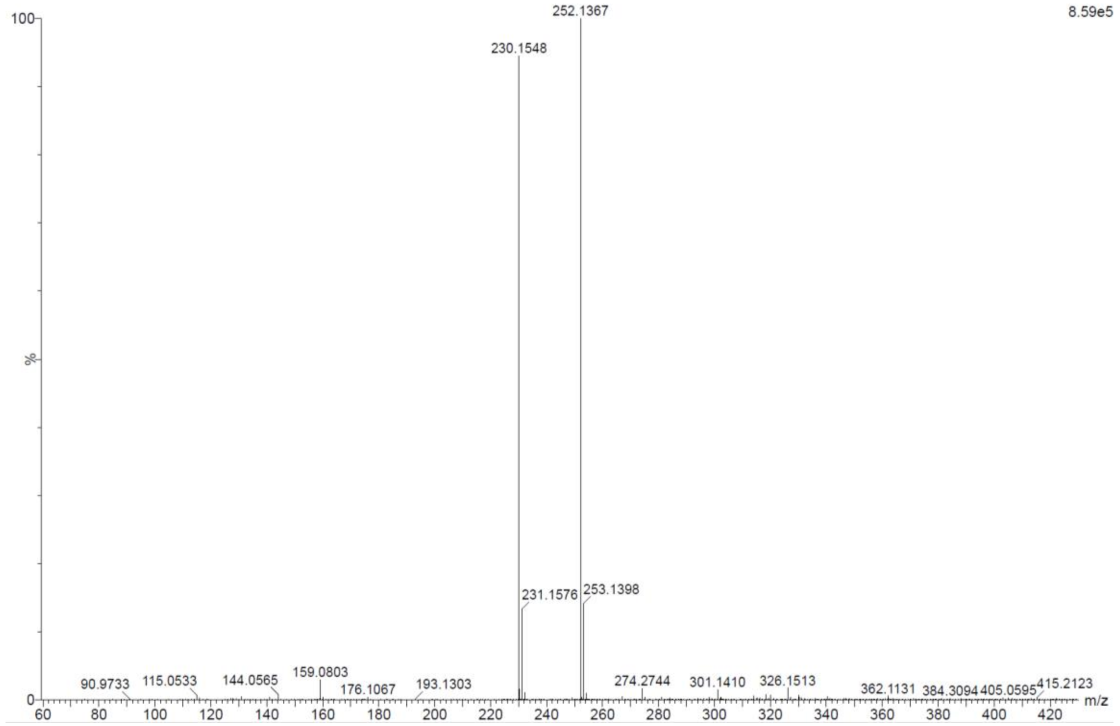

HRMS spectra of **F28**

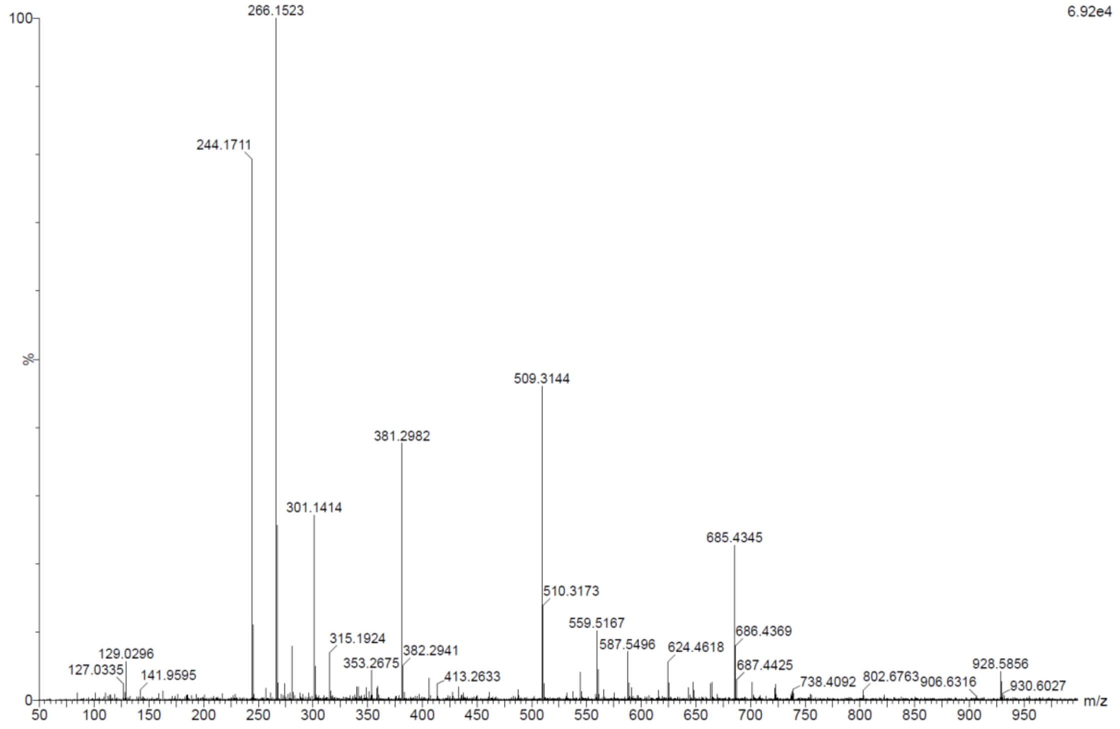

HRMS spectra of **F29**

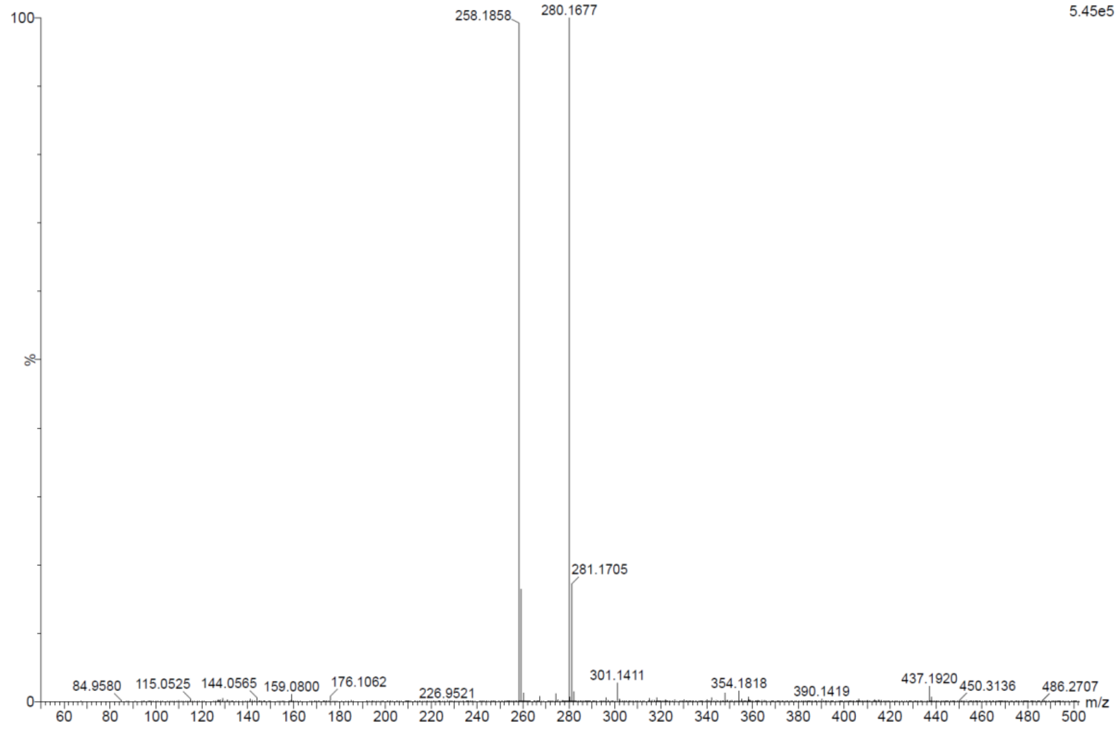

HRMS spectra of **F30**

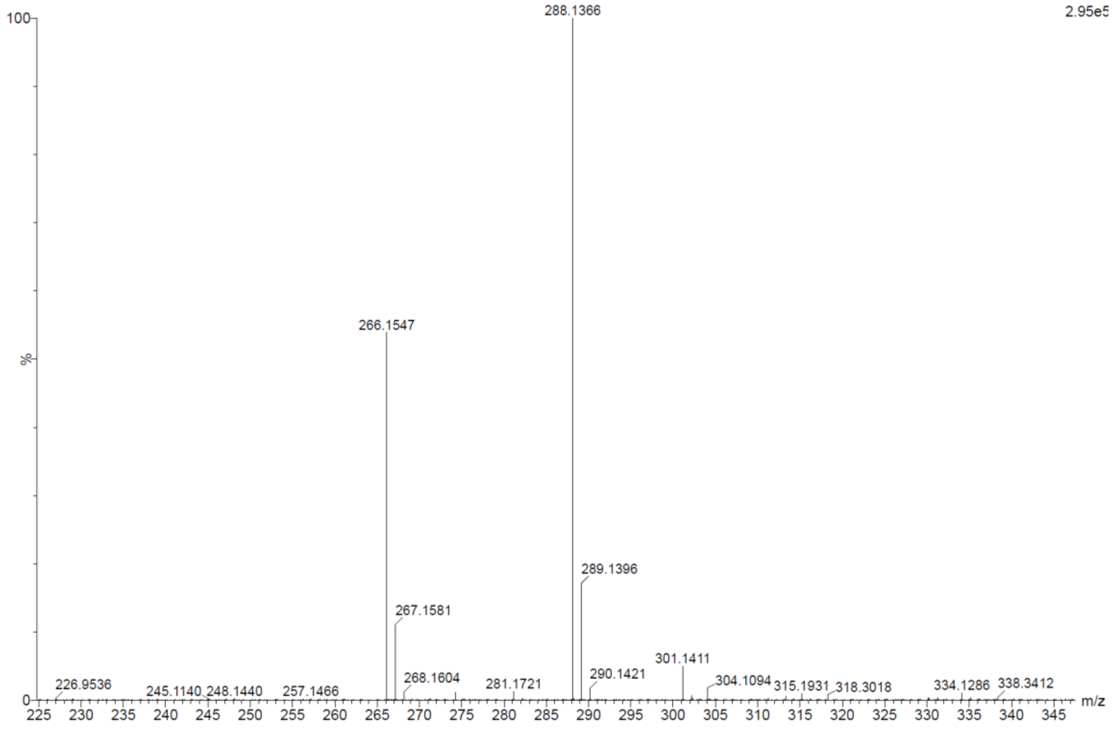

HRMS spectra of **F31**

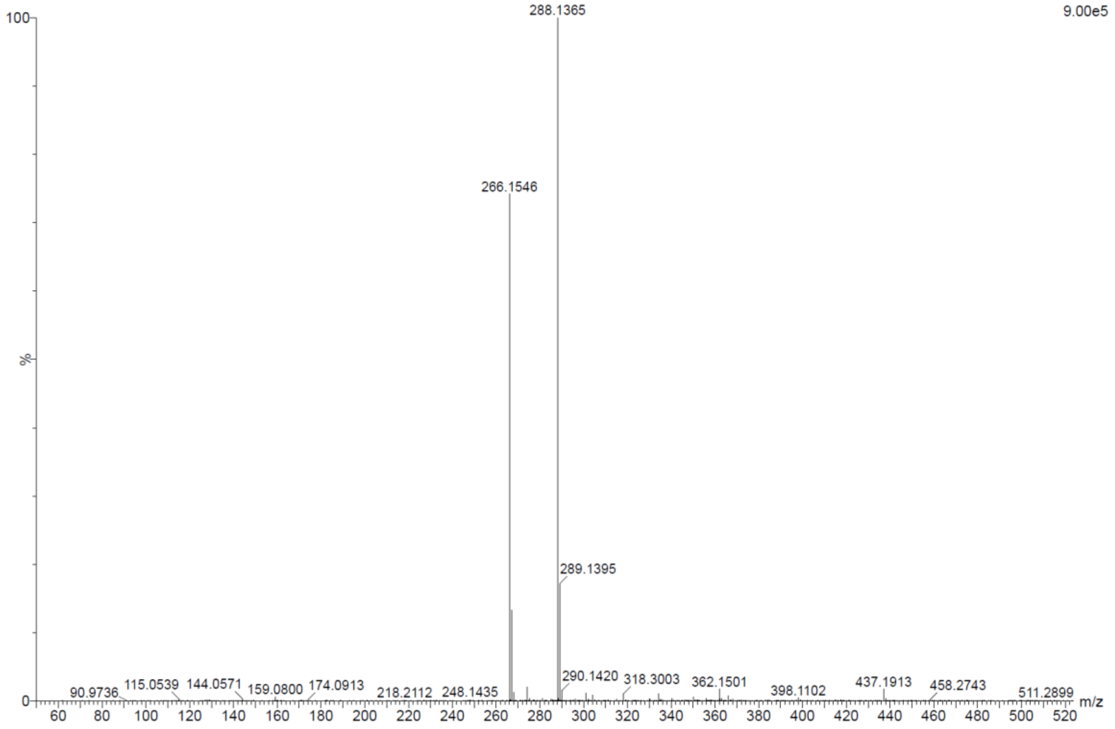

HRMS spectra of **F32**

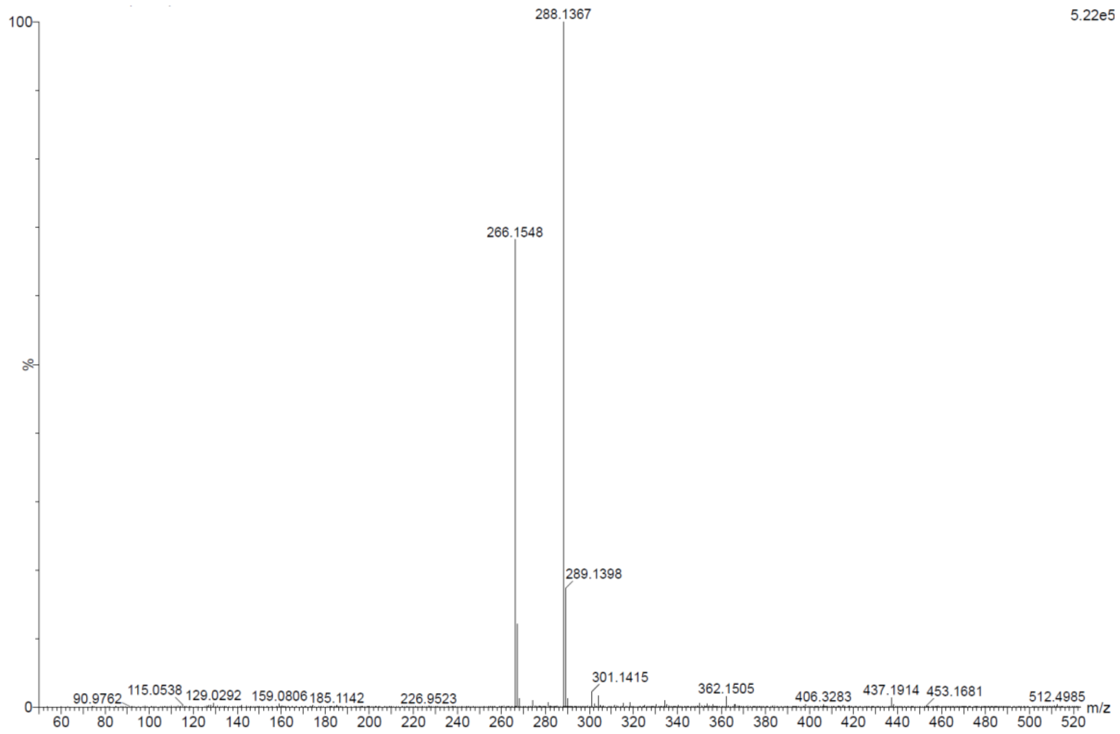

HRMS spectra of **F33**

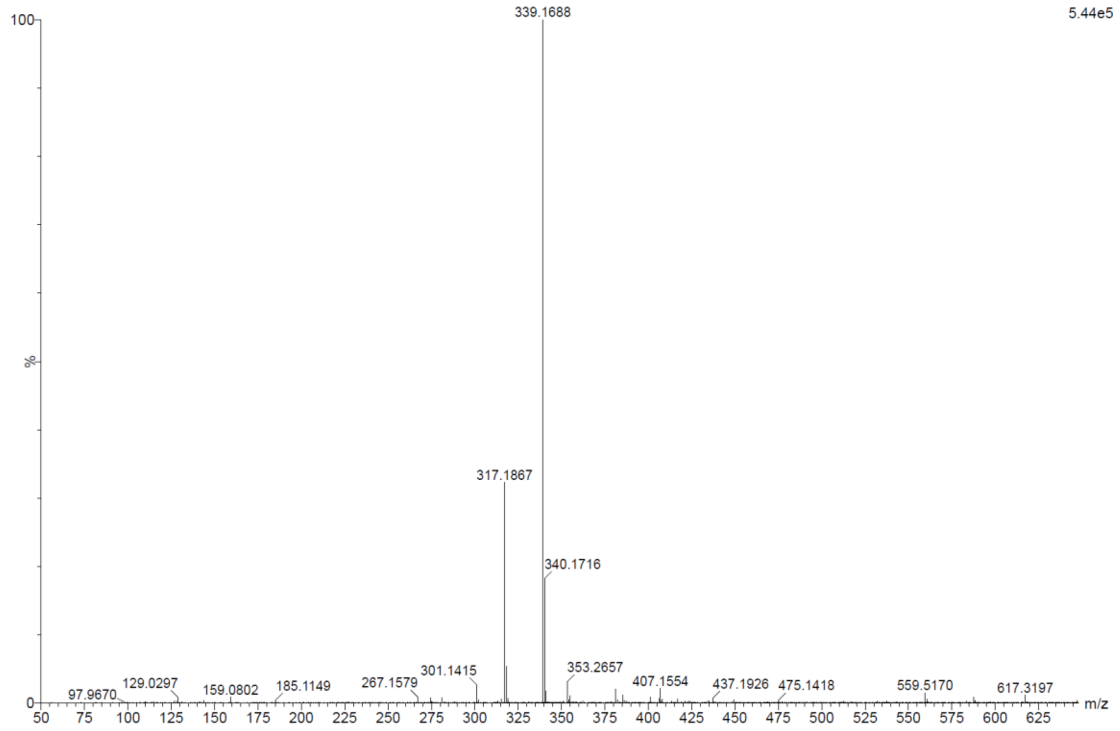

HRMS spectra of **F34**

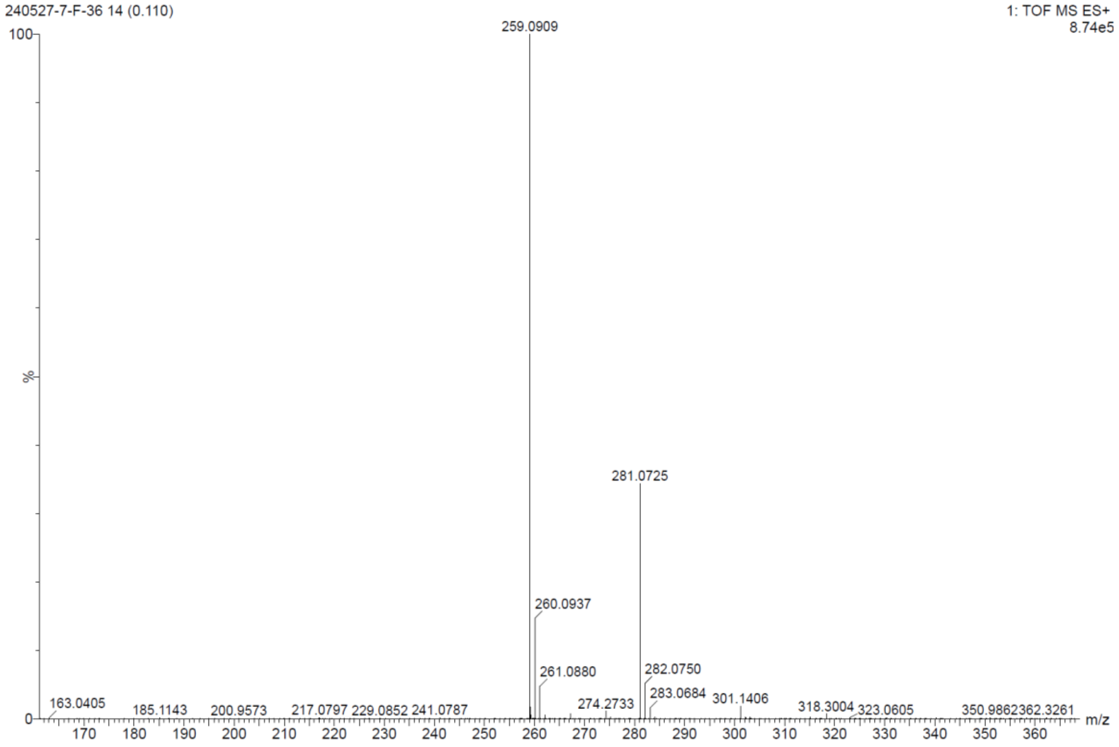

HRMS spectra of **F35**

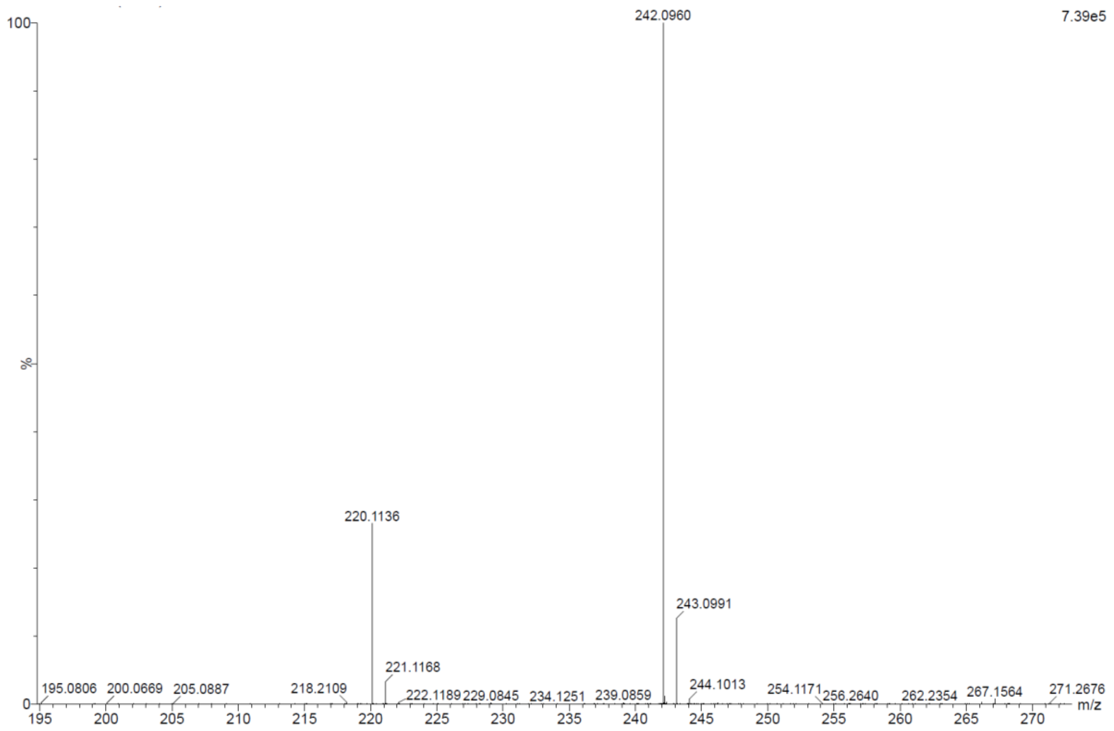

HRMS spectra of **F36**

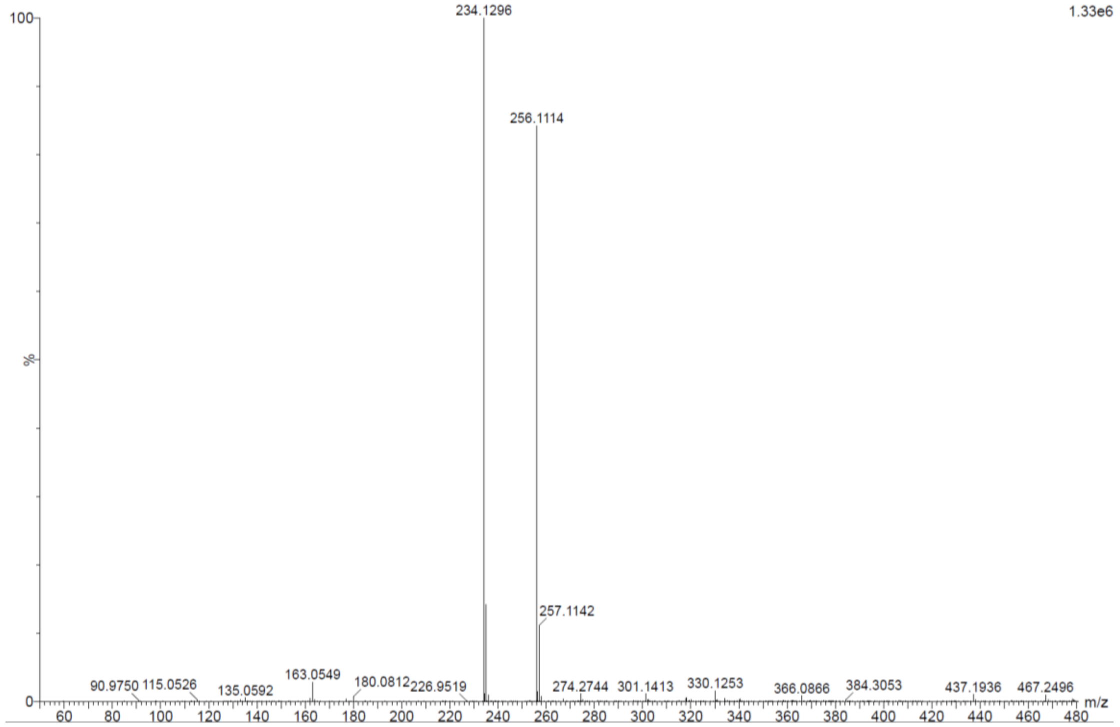

HRMS spectra of **F37**

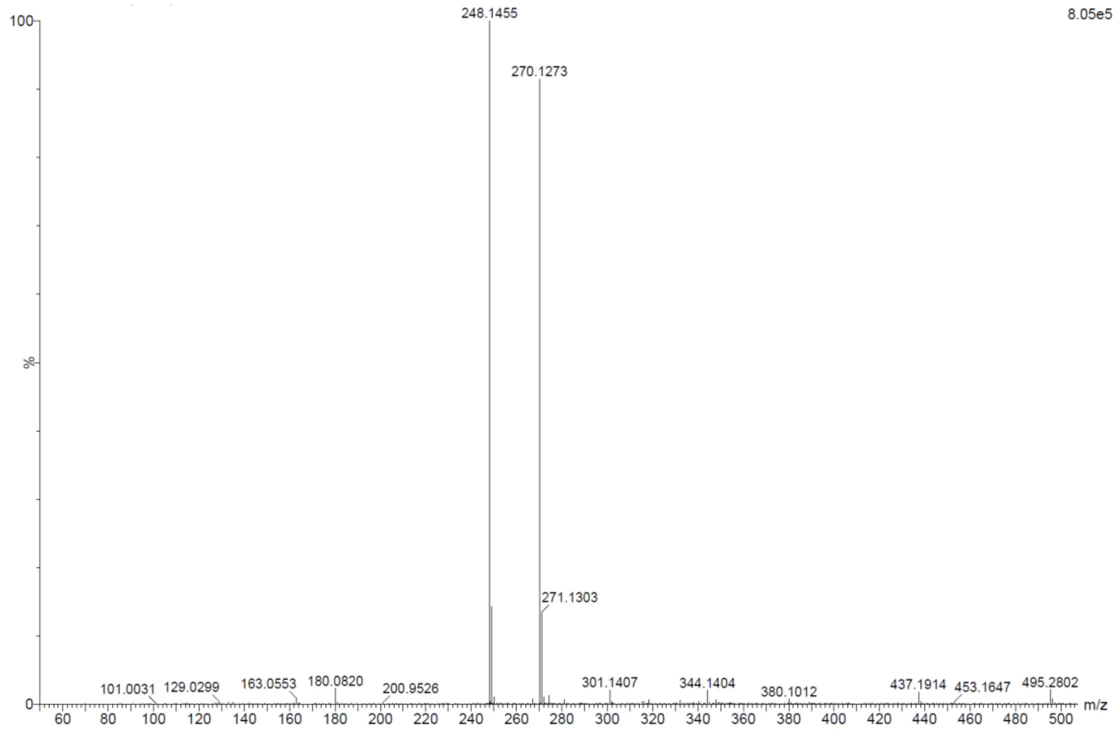

HRMS spectra of **F38**

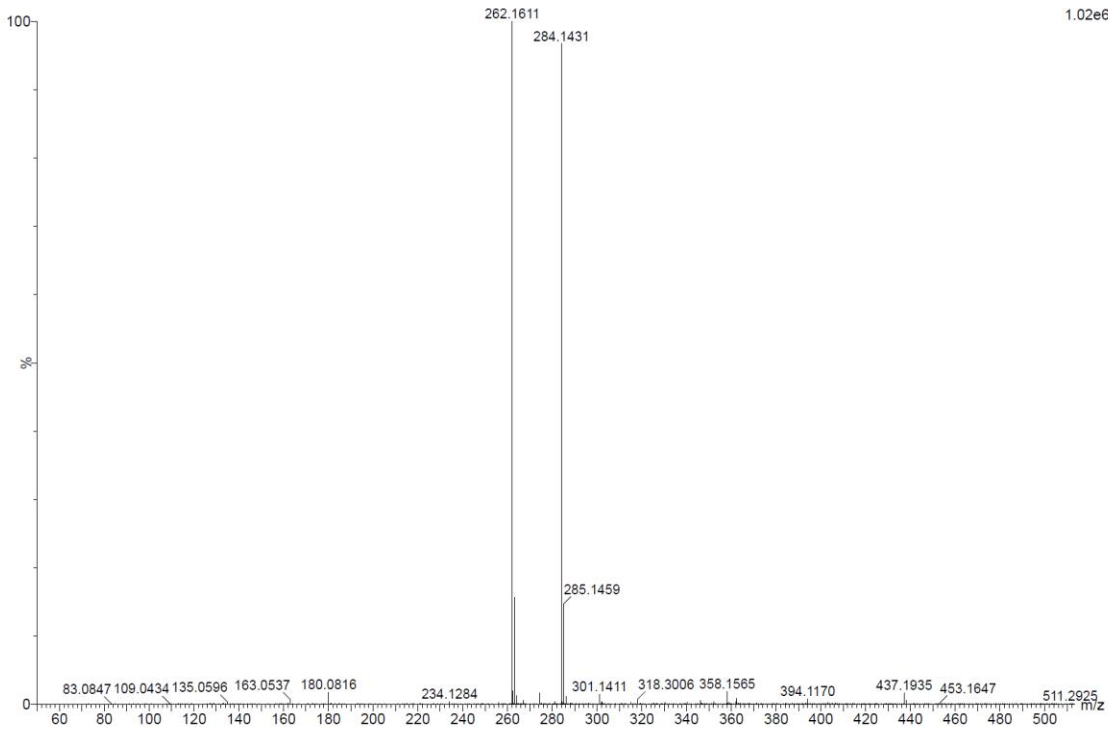

HRMS spectra of **F39**

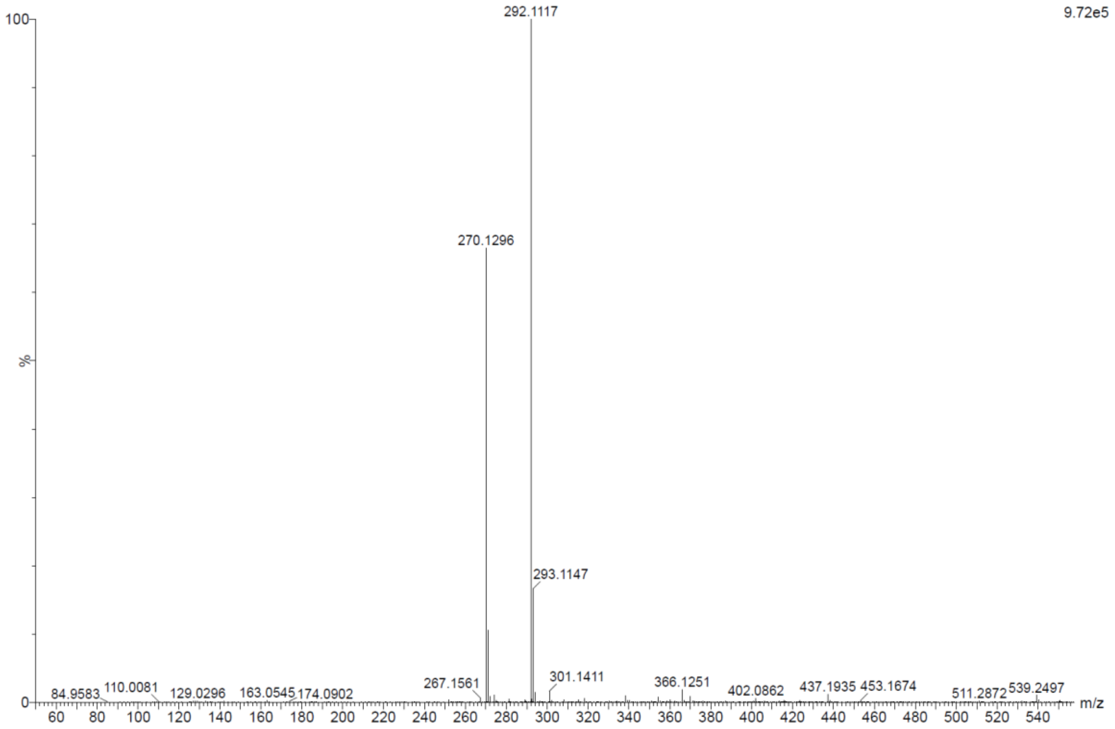

HRMS spectra of **F40**

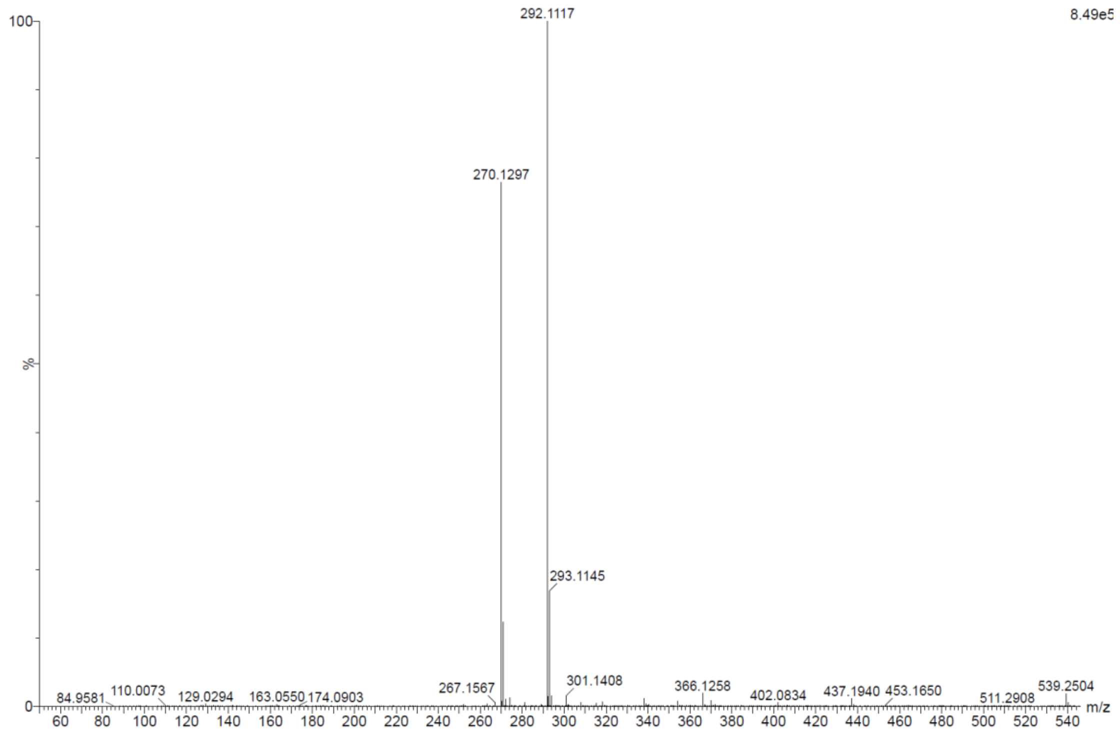

HRMS spectra of **F41**

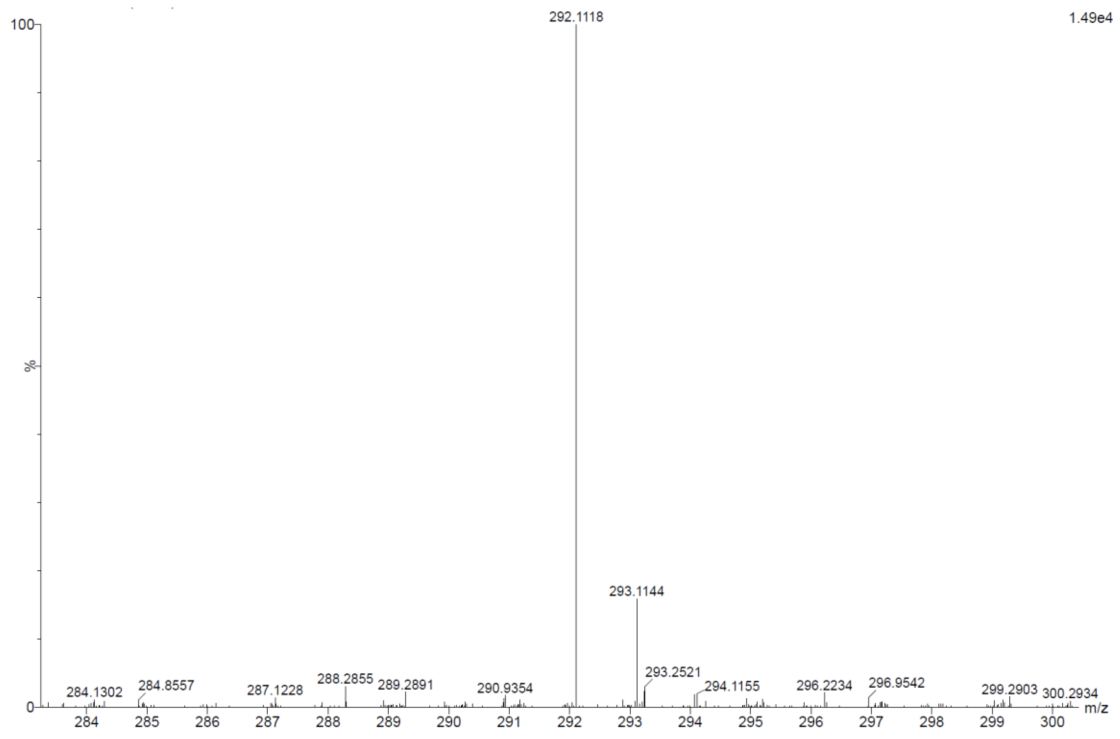

HRMS spectra of **F42**

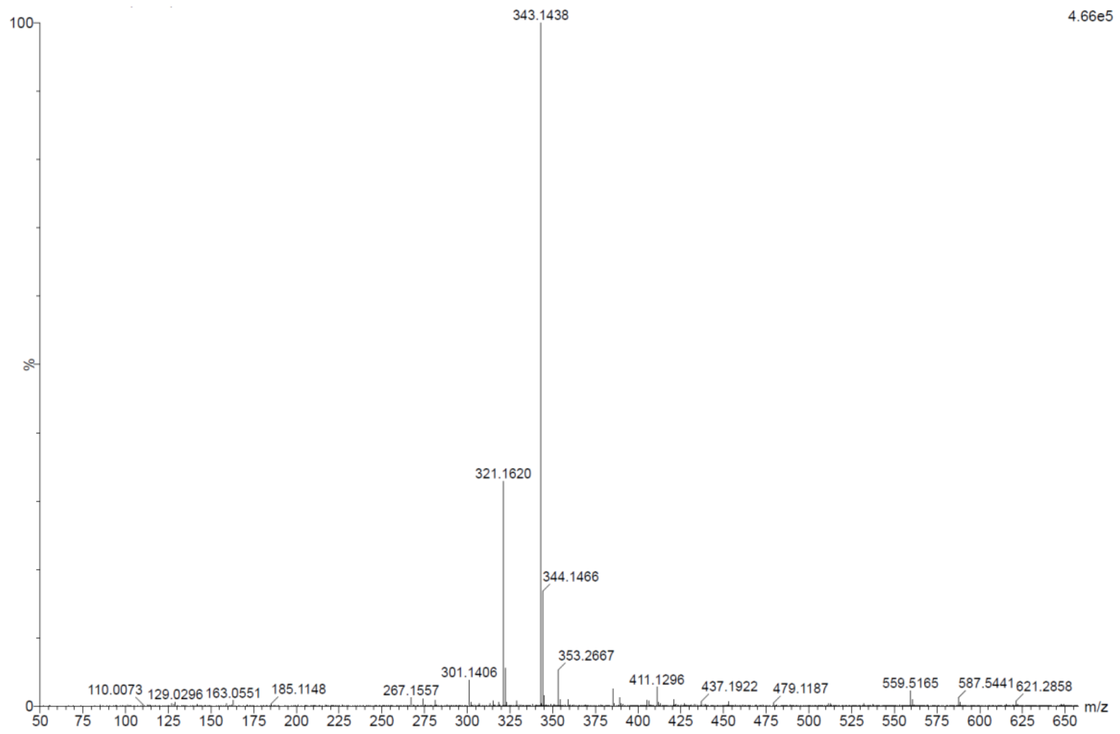

HRMS spectra of **F43**

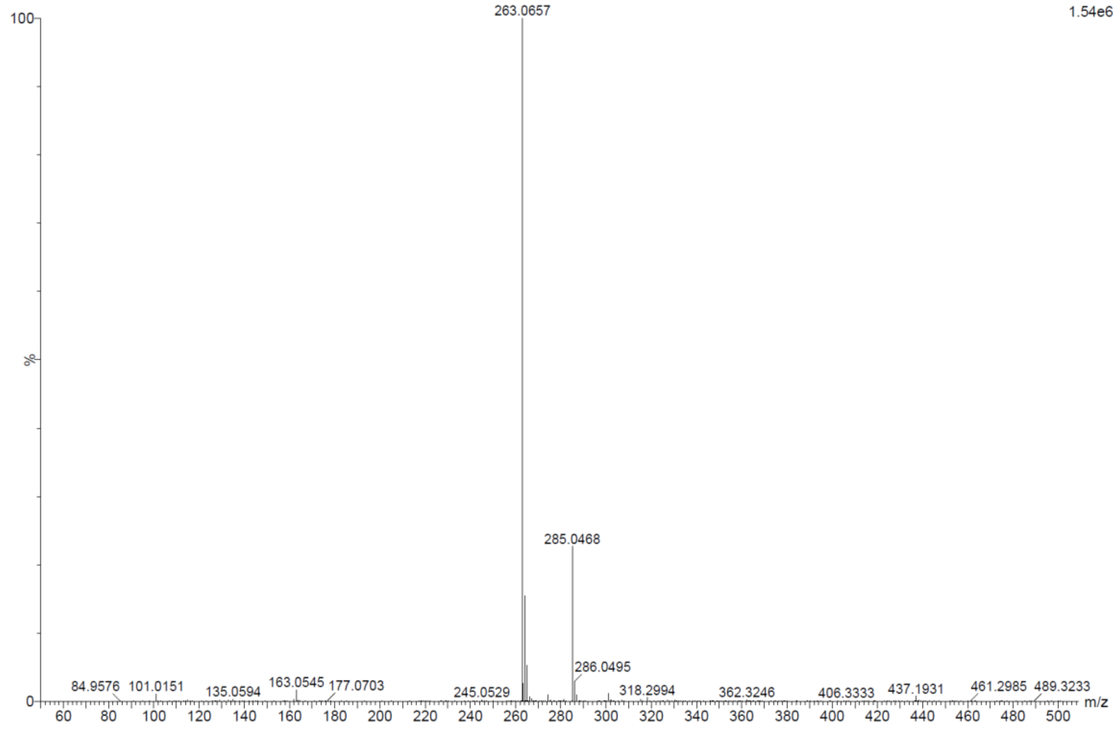

HRMS spectra of **F44**

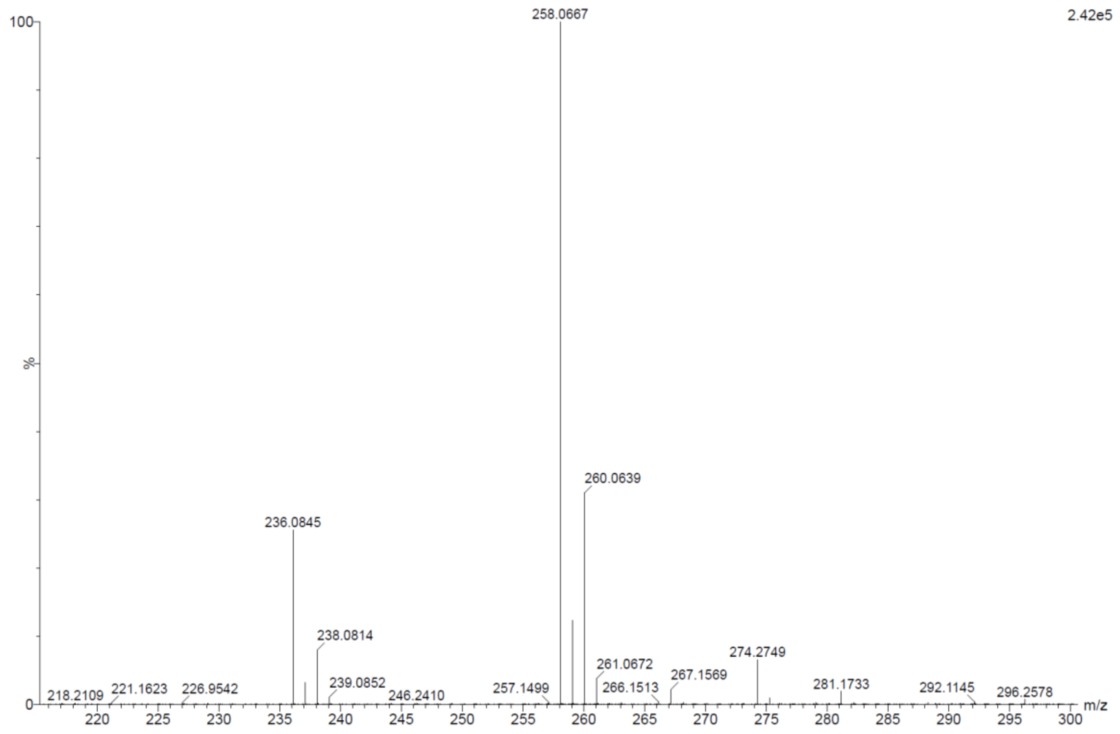

HRMS spectra of **F45**

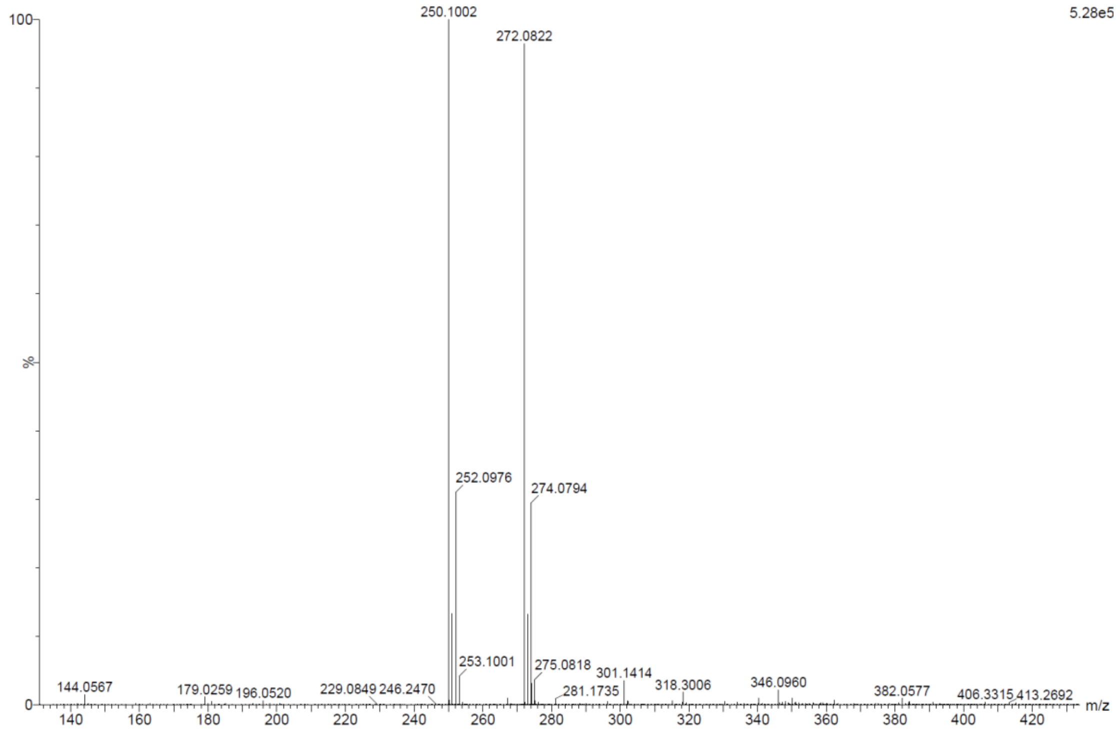

HRMS spectra of **F46**

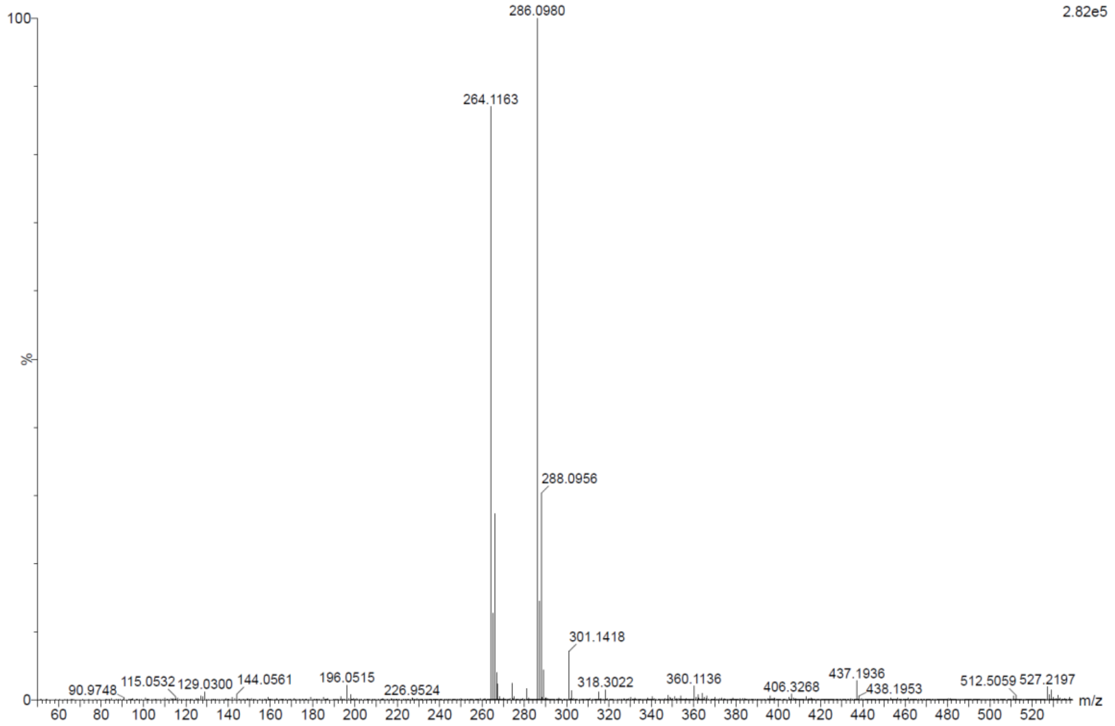

HRMS spectra of **F47**

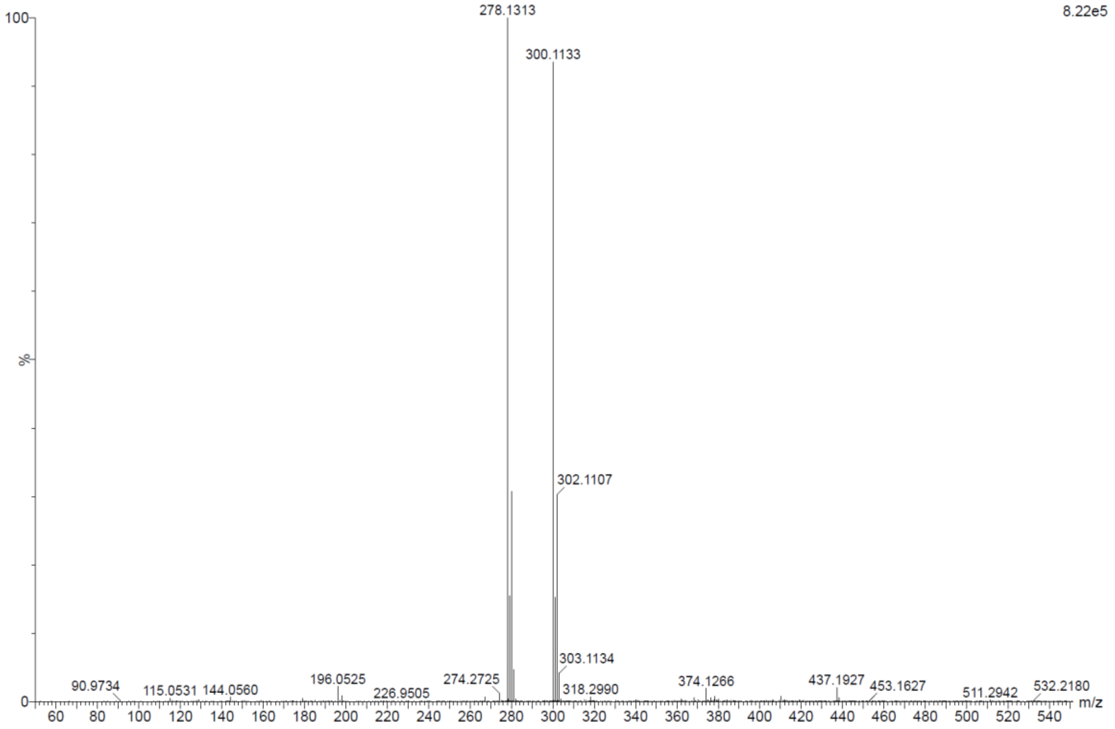

HRMS spectra of **F48**

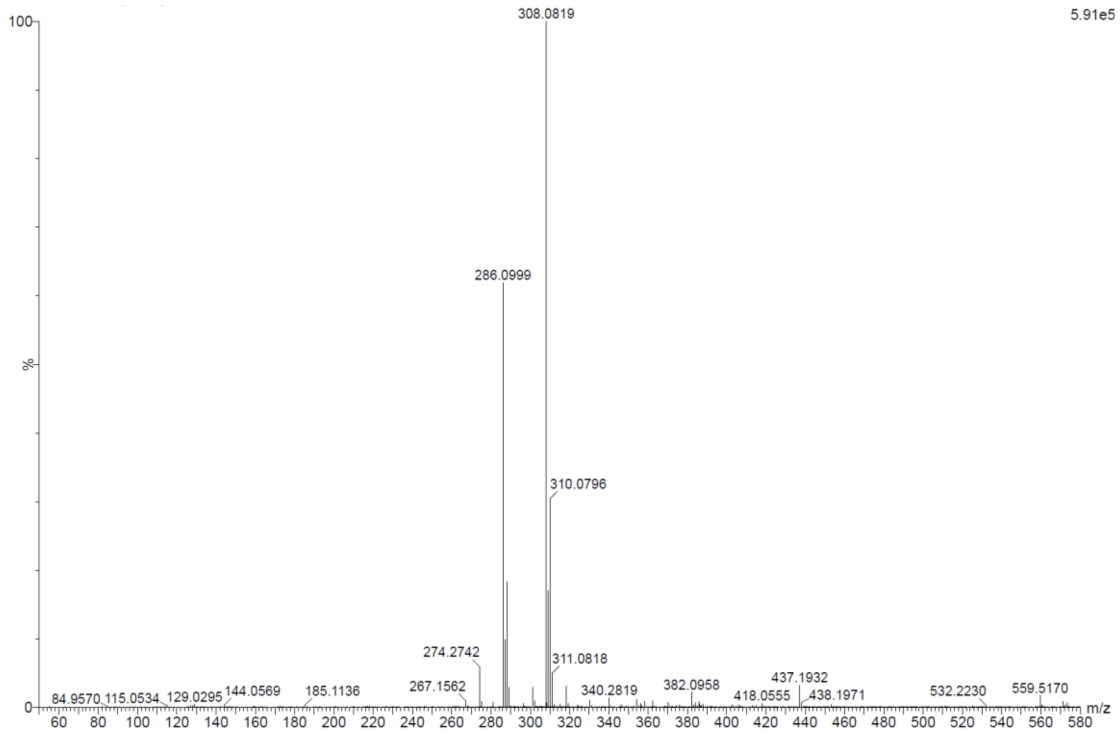

HRMS spectra of **F49**

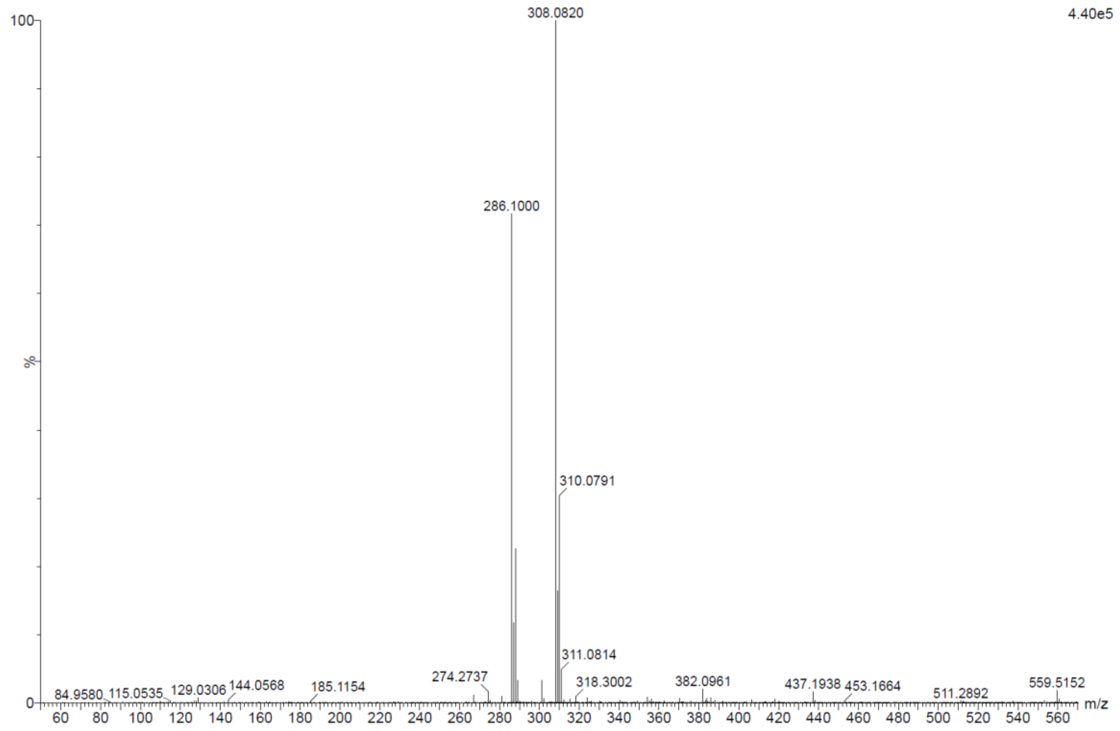

HRMS spectra of **F50**

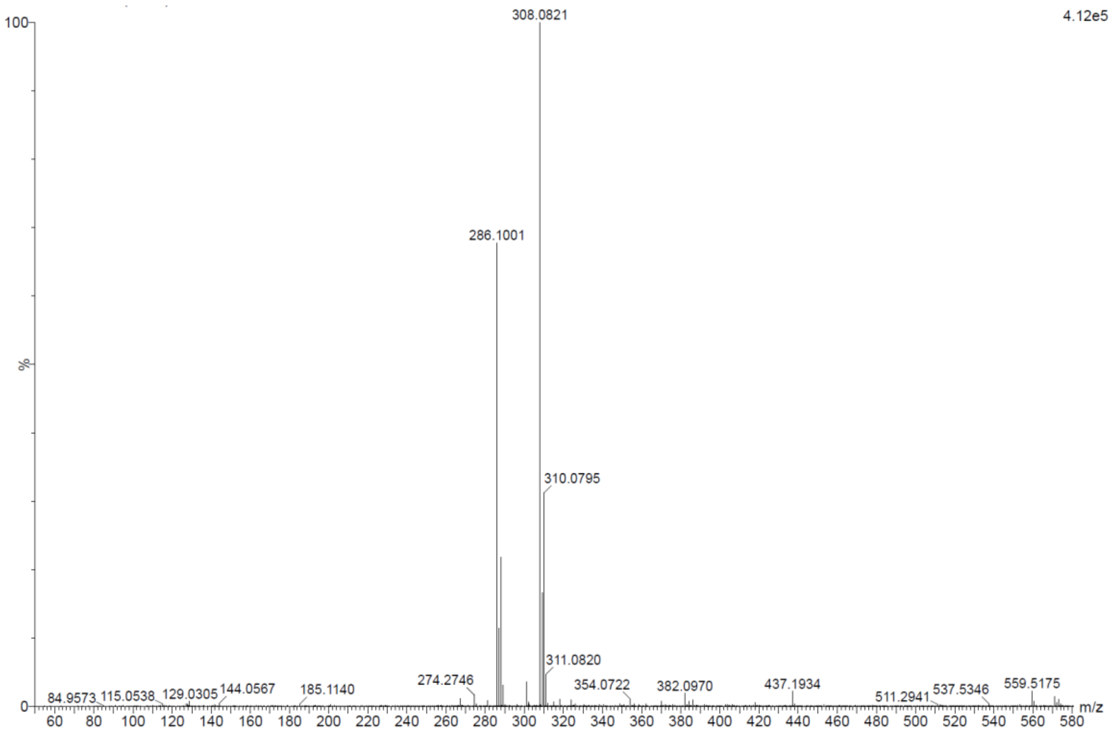

HRMS spectra of **F51**

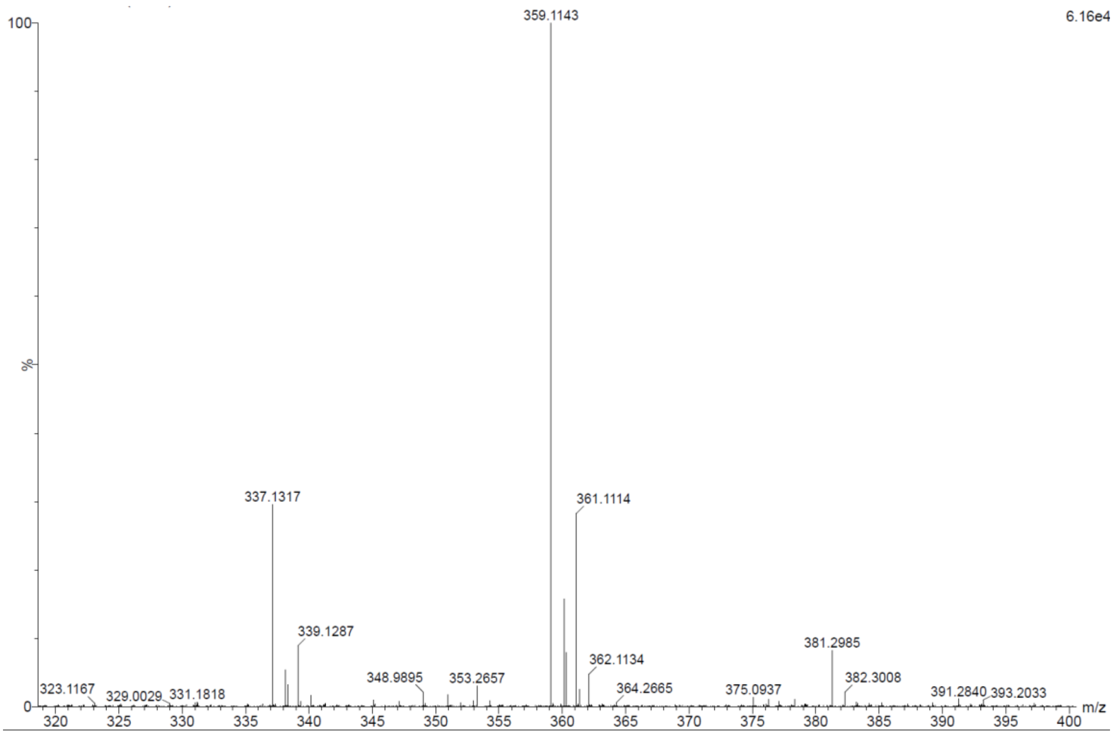

HRMS spectra of **F52**

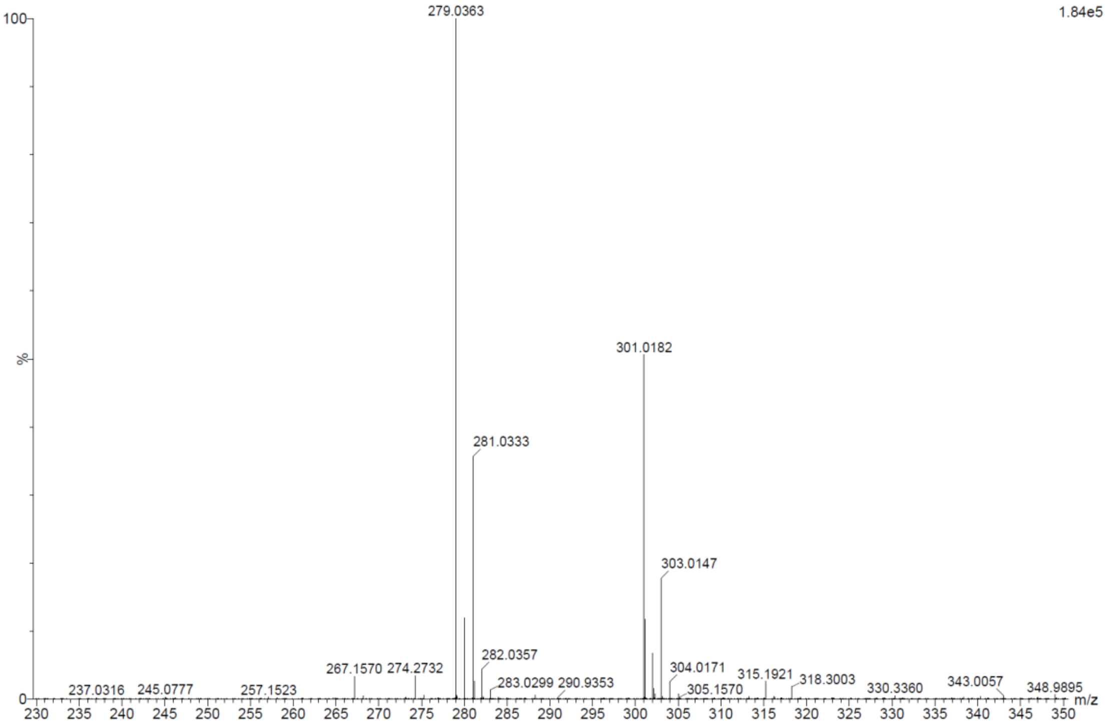

HRMS spectra of **F53**
